# α-Branched amines through radical coupling with 2-azaallyl anions, redox active esters and alkenes†

**DOI:** 10.1039/d2sc00500j

**Published:** 2022-03-03

**Authors:** Shengzu Duan, Yujin Zi, Lingling Wang, Jielun Cong, Wen Chen, Minyan Li, Hongbin Zhang, Xiaodong Yang, Patrick J. Walsh

**Affiliations:** Key Laboratory of Medicinal Chemistry for Natural Resources, Ministry of Education, Yunnan Province Center for Research & Development of Natural Products, School of Chemical Science and Technology, Yunnan University Kunming 650091 P. R. China xdyang@ynu.edu.cn zhanghb@ynu.edu.cn; Roy and Diana Vagelos Laboratories Penn/Merck Laboratory for High-Throughput Experimentation Department of Chemistry, University of Pennsylvania 231 South 34th Street Philadelphia PA USA pwalsh@sas.upenn.edu liminyan@sas.upenn.edu

## Abstract

α-Branched amines are fundamental building blocks in a variety of natural products and pharmaceuticals. Herein is reported a unique cascade reaction that enables the preparation of α-branched amines bearing aryl or alkyl groups at the β- or γ-positions. The cascade is initiated by reduction of redox active esters to alkyl radicals. The resulting alkyl radicals are trapped by styrene derivatives, leading to benzylic radicals. The persistent 2-azaallyl radicals and benzylic radicals are proposed to undergo a radical–radical coupling leading to functionalized amine products. Evidence is provided that the role of the nickel catalyst is to promote formation of the alkyl radical from the redox active ester and not promote the C–C bond formation. The synthetic method introduced herein tolerates a variety of imines and redox active esters, allowing for efficient construction of amine building blocks.

## Abstract 2

A mild method for the construction of α-branched amine derivatives is presented. SET processes between the Ni catalyst, redox active esters and 2-azaallyl anions generate azaallyl radicals and alkyl radicals that functionalize the alkenes.

## Graphical abstract

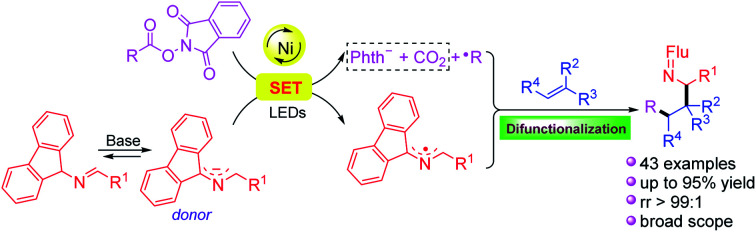

## Introduction

**Scheme 1:**
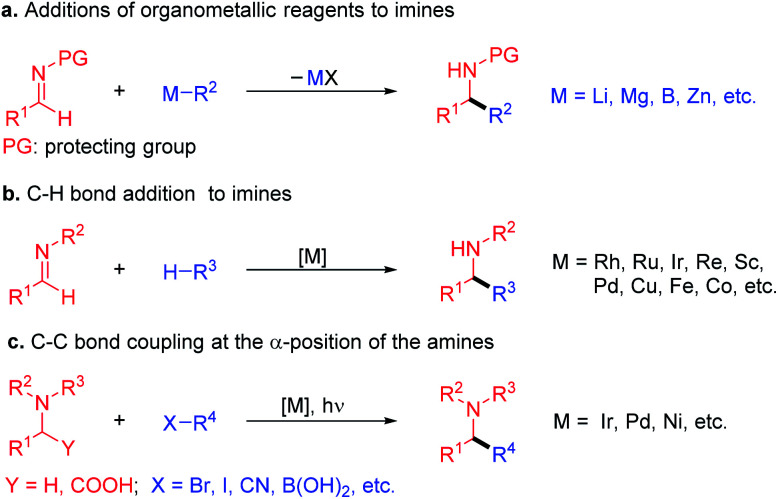
Approaches to α-branched amines. (a) α-Branched amine synthesis *via* addition of organometallic reagents to imines. (b) Transition-metal-catalyzed C–H activation followed by addition to imines. (c) Transition-metal-catalyzed α-functionalization of amines with C–C bond-formation.

Amines with α-branching are important functional groups in bioactive compounds,^1^ natural products,^2,3^ and medications.^4^ As such, their synthesis continues to attract attention, with emphasis on rapid access to new chemical space. The traditional approach to α-branched amines involves the addition of organometallic reagents,^5,6^ such as Grignard reagents and organolithiums, to imines (Scheme 1a).^7–10^ Recent years have witnessed remarkable progress in alternative syntheses of α-branched amines. A more atom economical method employs transition-metal-catalyzed C–H bond activation followed by addition of the resulting organometallic nucleophile to imines (Scheme 1b).^11–13^ The α-functionalization of amines toward the formation of α-branched derivatives has also been developed. Here, both transition-metal-catalyzed processes^14^ and photoredox catalyzed radical coupling approaches to form C–C bonds have been successfully introduced (Scheme 1c).^15–18^ Although the utility of these reactions is well appreciated, several employ the use of precious metal catalysts.

**Scheme 2:**
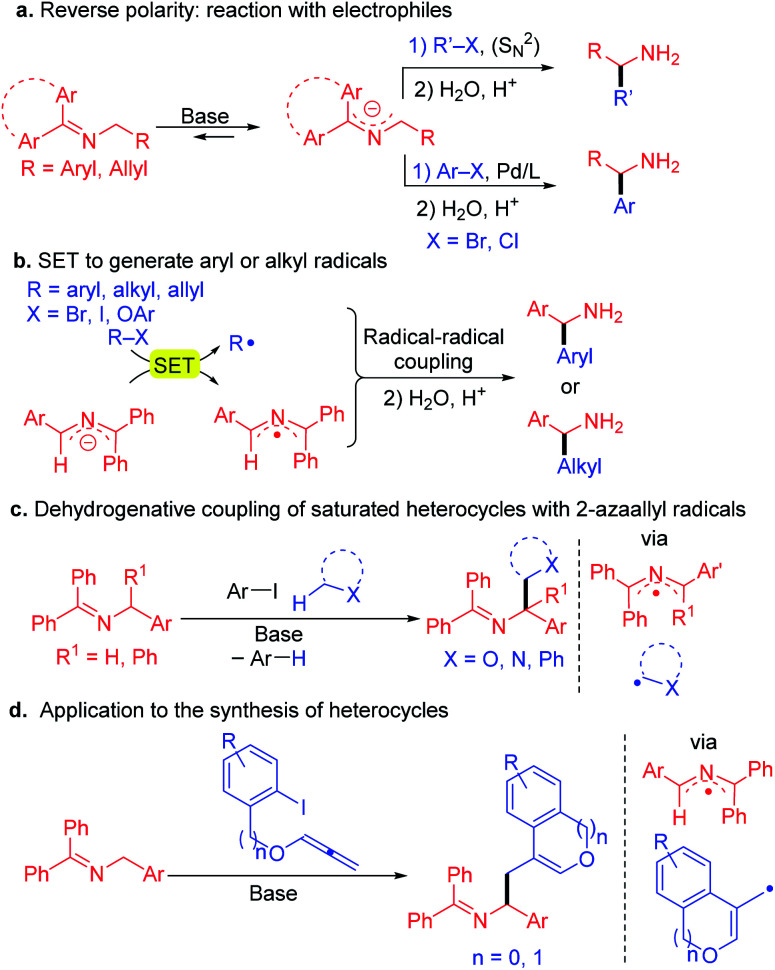
Reactivity of 2-azaallyl anions. (a) 2-electron processes such as S_N_2 and Pd-catalyzed coupling reactions of 2-azaallyl anions. (b) Single electron reactivity of 2-azaallyl anions with aryl halides and tertiary alkyl iodides *via* radical intermediates. (c) Dehydrogenative coupling of saturated heterocycles with 2-azaallyl radicals. (d) Applications to synthesis involving trapping of aryl radicals with allenes ultimately affording heterocyclic amines.

Alternatively, we and many others, have been interested in an approach involving the inversion of polarity of the imine by generation of 2-azaallyl anions.^19^ Recent studies have shown the utility of 2-azaallyl anions in a wide range of C(sp^2^)–C(sp^3^) and C(sp^3^)–C(sp^3^) coupling reactions *via* 2-electron processes. The 2-azaallyl anion is usually generated *in situ* by deprotonation of aldimines or ketimines under mild conditions and have the advantage of avoiding preformed organometallic reagents.^20–27^ The 2-azaallyl anions can react with unhindered alkyl halides *via* S_N_2, or with aryl halides in the presence of cross-coupling catalysts (Scheme 2a).^28–32^ A similar approach was use with aryl bromides and alkyl substituted 2-azaallyl anions in the presence of an enantioenriched Pd catalyst^24^ or with vinyl bromides and an enantioenriched nickel catalyst^33^ for the synthesis of highly enantioenriched benzylic and homoallylic amine derivatives, respectively. Azadiene precursors have also been hydrometallated to generate 2-azaallyl anions for enantioselective transformations.^20,34–36^ Hydrolysis of the alkylation, vinylation or arylation products affords α-branched amines with high ee values.

Recently we discovered that 2-azaallyl anions behave as super electron donors (SEDs)^37^ and undergo 1-electron processes with a variety of electrophiles, opening a new reactivity mode and enabling the synthesis of α-branched amines (Scheme 2b). Thus, reaction of 2-azaallyl anions with aryl iodides,^38^ bromides or even chlorides (under light irradiation)^39^ resulted in SET from the 2-azaallyl anion to the aryl halide to generate an aryl radical and the persistent^40^ 2-azaallyl radical, which then undergo a radical–radical coupling to afford the arylated product. With hindered alkyl iodides and bromides, such as 1-adamantyl iodide, the 2-azaallyl anion undergoes SET generating the alkyl radical, which again couples with the 2-azaallyl radical.^40^ Likewise, phenyl allyl ethers react to furnish homoallylic amines.^41^ The 2-azaallyl radical and anions related to the intermediates in these processes were recently isolated and fully characterized (including X-ray crystallographically).^42^

Inspired by the single electron transfer (SET) behavior of 2-azaallyl anions, we examined the applications of 2-azaallyl anions in the synthesis of amines. We discovered that using bulky aryl iodides, such as 2,6-dimethyl iodobenzene derivatives (Scheme 2c), the resulting sterically hindered aryl radical generated after SET and loss of iodide does not readily couple with the 2-azaallyl radical. Instead, the aryl radical undergoes HAT with nucleophilic C–H's (THF, toluene, α-amino C–H's) to generate stabilized radicals. These radicals then couple with the 2-azaallyl radical. The net result is a cross dehydrogenative coupling of two C–H bonds to form amine derivatives.^43^ In an application to the synthesis of heterocycles (Scheme 2d), treatment of aryl iodides possessing pendent allenyl ethers resulted in a tandem reduction to the aryl radical, cyclization onto the allene, and subsequent radical–radical coupling to lead to amino ethyl benzofurans and isochromene derivatives.^44,45^

Related radical processes have recently gained much attention in the area of amine synthesis, often under photolytic conditions.^46–48^ A long-standing goal in amine synthesis is to introduce functionality at different positions relative to the amino group. One strategy to accomplish this goal is to generate an α-amino radical that adds to a C=C π-bond. Attempts to achieve this mode of reactivity using 2-azaallyl species of the type described in Scheme 2a and b, however, were unsuccessful. To overcome this issue, we set out to identify (1) electron acceptors that can more easily undergo SET reductive activation by less reducing 2-azaallyl anions and (2) radical acceptors that would couple with the resulting more stabilized 2-azaallyl radicals.

**Scheme 3:**
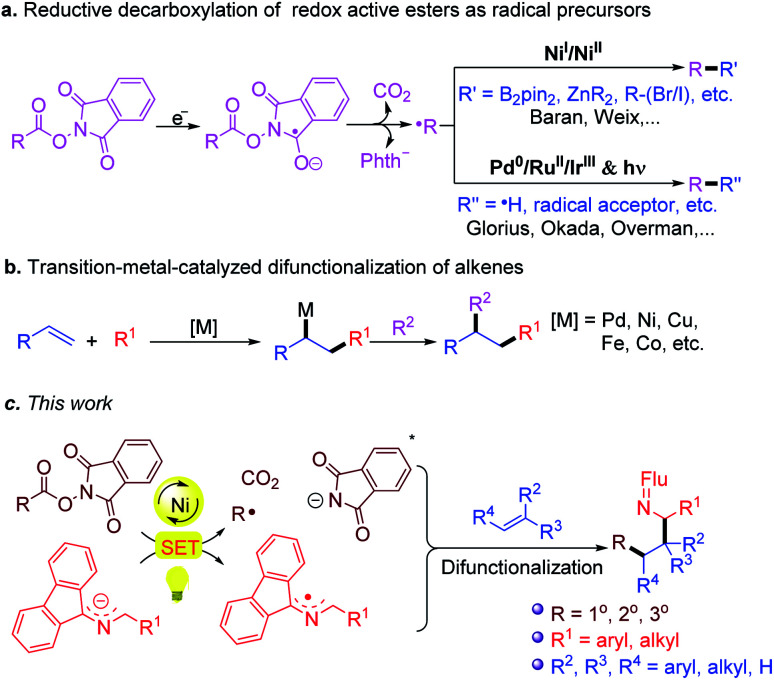
(a) Reductive decarboxylation of redox active esters as radical precursors. (b) Transition-metal-catalyzed difunctionalization of alkenes. (c) This work: a strategy towards the difunctionalization of alkenes with 2-azaallyl anions and redox active esters.

In recent years, the emergence of redox active esters (RAEs) as alkyl radical precursors has marked a milestone in the art of organic synthesis (Scheme 3a),^49–60^ particularly when combined with transition metal-catalyzed processes, such as alkene difunctionalization (Scheme 3b).^61–73^ In light of these advances, and our previous work on 2-azaallyl radical chemistry, we envisioned that even weakly reducing 2-azaallyl anions could match the redox properties of RAE to initiate the SET reduction and generate alkyl radicals and 2-azaallyl radicals. It was initially envisioned that the transient alkyl radical could be trapped by suitably activated alkenes in the presence of transition metal catalysts. Subsequent capture of the 2-azaallyl radical by the catalyst followed by reductive elimination was expected to form functionalized amines (Scheme 3c). Herein we report initial results from trapping 2-azaallyl radicals with alkenes under nickel catalysis in an alkene difunctionalization process (Scheme 3c). We also provide support for the notion that the nickel catalyst is *not involved* in the C–C bond formation, but is responsible for the generation of the alkyl radical from the redox active ester. We hypothesize that these results are relevant to other nickel catalyzed C(sp^3^)–C(sp^3^) cross-coupling reactions of persistent radicals.

## Results and discussion

### Reactivity concerns

At the outset of our study, we were concerned about several side reactions and challenges. These include: (1) dimerization^74^ or HAT quenching of 2-azaallyl radicals or alkyl radicals; (2) radical selectivity in the addition to the alkene acceptor; and (3) regioselectivity of alkene functionalization. We envisioned that the difference in reactivity between the alkyl radical and the persistent 2-azaallyl radical might work in our favor to address both the selectivity in which radical adds first to the alkene while simultaneously determining the regioselectivity.

### Reaction development and optimization

Before initiating our investigation, reaction conditions used in 2-azaallyl coupling reactions,^19,42^ alkene functionalization reactions^75,76^ and reductive activation of RAEs^60,77–82^ were studied. Based on these works, Ni(COD)_2_ was selected as the metal source,^33^ DIPEA as base,^31,59,69,83^ THF/DMF as solvent (4 : 1, based on literature precedence^50,51,84^). A series of ligands were screened in the cascade coupling between imine 1a, adamantyl RAE 2a and 1,1-diphenylethylene 3a. Blue LEDs were used to improve the reducing power of the 2-azaallyl anions, as demonstrated in previous research.^39^ Out of 3 bipyridyl type ligands (L1–L3, Table 1, entry 1), 3 phenanthroline type ligands (L4–L6, Table 1, entry 2) and 3 phosphine ligands (L7–L9, Table 1, entries 3–4), 1,3-bis(diphenylphosphino)propane (L9) provided the target product 4aa in 90% assay yield (AY, determined by ^1^H NMR integration against an internal standard) and 86% isolated yield (Table 1, entry 4). We were pleased to find that the alkene functionalization is chemo- and regioselective, likely because the transient alkyl radical is more reactive than the 2-azaallyl radical. Attack of the alkyl radical on the alkene at the terminal position affords a stabilized benzylic radical. The reducing feature of 2-azaallyl anions enabled the reaction without blue LED, albeit in a reduced 62% yield (Table 1, entry 5).

**Table 1:** Optimization of photoinduced/nickel-catalyzed difunctionalization of alkenes^aa^

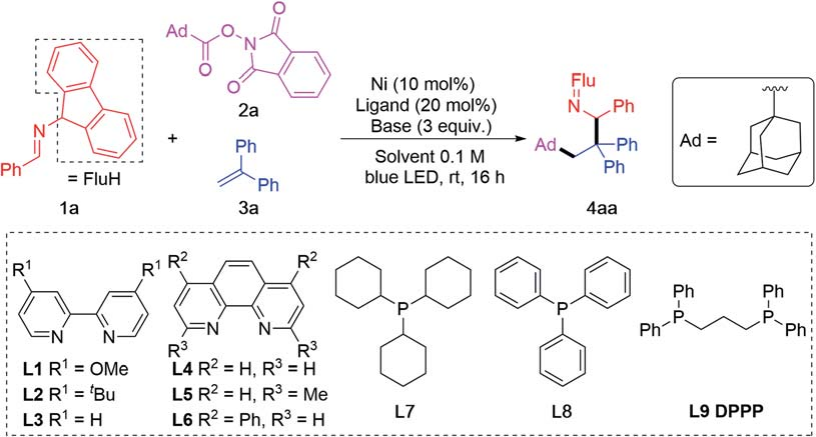					
Entry	L	Ni	Base	Solvent	4aa^b^ (%)
1	L1–L3	Ni(COD)_2_	DIPEA	THF/DMF = 4 : 1	82–88
2	L4–L6	Ni(COD)_2_	DIPEA	THF/DMF = 4 : 1	40–61
3	L7–L8	Ni(COD)_2_	DIPEA	THF/DMF = 4 : 1	25–36
4	L9	Ni(COD)_2_	DIPEA	THF/DMF = 4 : 1	90 (86)^c^
5	L9	Ni(COD)_2_	DIPEA	THF/DMF = 4 : 1	62^d^
6	L9	—	DIPEA	THF/DMF = 4 : 1	0
7	L9	NiCl_2_	DIPEA	THF/DMF = 4 : 1	0
8	L9	NiBr_2_	DIPEA	THF/DMF = 4 : 1	0
9	L9	Ni(COD)_2_	DBU	THF/DMF = 4 : 1	42
10	L9	Ni(COD)_2_	Et_3_N	THF/DMF = 4 : 1	61
11	L9	Ni(COD)_2_	LiO^*t*^Bu	THF/DMF = 4 : 1	15
12	L9	Ni(COD)_2_	DIPEA	DMF	75
13	L9	Ni(COD)_2_	DIPEA	DMA	85
14	L9	Ni(COD)_2_	DIPEA	THF/DMF = 4 : 1	87^e^
15	L9	Ni(COD)_2_	DIPEA	THF/DMF = 4 : 1	63^f^

a Reactions conducted on a 0.1 mmol scale using 1 equiv. of 1a, 1.5 equiv. of 2a, and 3 equiv. of 3a, with Ni(COD)_2_ (10 mol%), ligand (20 mol%), base (3.0 equiv.) and solvent (1.0 mL, 0.1 M).

b AY were determined by ^1^H NMR spectroscopy with C_2_H_2_Cl_4_ as internal standard. Flu = 9-fluorenyl.

c Isolated yield of 4aa after chromatographic purification.

d Without blue LED.

e 1 : 1 ratio of 1a and 2a.

f 1.5 : 1 ratio of 1a and 2a. Ad = 1-admantyl, Flu = 9-fluorenyl.

Although we had not previously used RAE substrates, we were guided by our past studies^38,43,44^ and assumed that the metal catalyst would not be needed in the SET between the 2-azaallyl anion and RAE. No product was observed, however, when the Ni catalyst was absent (Table 1, entry 6, about 40% of the RAE was consumed, 60% remained). This result caused us to question our original hypothesis that the 2-azaallyl anion was responsible for generation of the radical from the RAE. One possibility that is discussed later is that the Ni catalyst is involved in the SET to the RAE. Interestingly, switching Ni(0) to Ni(ii) led to no product generation (Table 1, entries 7–8). We next examined other bases on the reaction AY. Nitrogen bases, such as 1,8-diazabicyclo(5.4.0)undec-7-ene (DBU) or NEt_3_, afforded lower efficiency (61% and 42% AY, respectively. Table 1, entries 9–10). Stronger bases, such as LiO^*t*^Bu, shut down the reaction (Table 1, entry 11). Consistent with the literature reports on RAEs,^49,51^ the reaction can be performed with slightly lower yields in DMF and DMA (75–85% AY, Table 1, entries 12–13). The ratio of 1a to RAE 2a was also studied. While a 1 : 1 ratio of 1a and 2a afforded slightly diminished 87% AY (Table 1, entry 14), increasing the loading of imine to 1.5 equiv. caused a drop in the AY to 63% (Table 1, entry 15).

### Reaction scope of RAEs and alkenes

With the optimized conditions in hand (Table 1, entry 4), we began to evaluate diverse redox-active-esters as precursors of primary, secondary and tertiary alkyl radicals (Table 2). A broad array of alkyl radicals was shown to be tolerated in this process, enabling functionalization of the amine gamma to the nitrogen. Unactivated primary alkyl groups (CH_2_R) with R = *t*-Bu, cyclobutyl, cyclopentyl and cyclohexyl groups (2b–2e) gave products in 89–92% yields. When R = heterocyclic piperidine (2f) and tetrahydropyran (2g) the products were isolated in 84% and 72% yields, respectively.

**Table 2:** Scope of redox-active esters and alkenes^a^

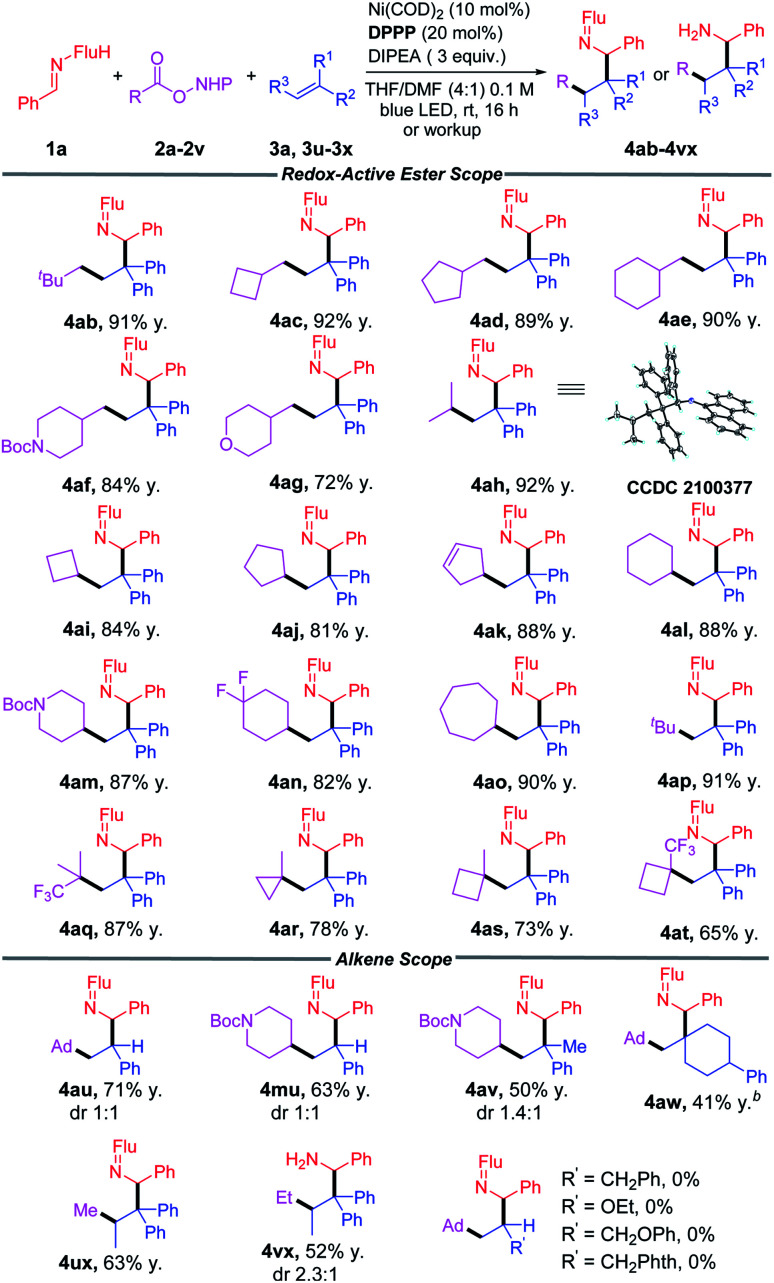

a Reactions conducted on a 1 mmol scale using 1 equiv. 1a, 1.5 equiv. 2 and 3 equiv. 3a, 3u–3x at 0.1 M. Isolated yields after chromatographic purification.

b 48 h reaction time. Ad = 1-admantyl, Flu = 9-fluorenyl.

Secondary aliphatic groups, including *i*-Pr and cyclic 4-, 5-, 6-, and 7-membered ring systems (2h–2o) all successfully coupled in this reaction and gave the desired products in 81–92% yields. Tertiary radicals were reported to exhibit low-yields in previously established methods.^51,85^ In our method, ^*t*^Bu- (2p) and CF_3_-dimethylpropyl (2q) RAEs were obtained with 91% and 87% yields, respectively. Cyclic tertiary groups (2r, 2s, 2t) were found to be suitable substrates, generating the amines in good yields (65–78%).

Substituents on the alkene serve to direct the alkyl radical addition and stabilize the newly formed radical. They are, therefore, a key parameter in this process. We next wanted to evaluate the impact of different alkene substituents. Switching from 1,1-diphenylethylene to styrene resulted in the formation of 4au and 4mu in 71% and 63% yield, respectively, as an equal mixture of diastereomers. Use of 2-phenylpropene cause the yield to fall to 50% (4av). The impact of radical stabilizing groups on the alkene was probed when the phenyl groups were completely removed. Thus, use of (4-methylenecyclohexyl)benzene resulted in only 41% yield of 4aw. While the yields with less stabilizing alkene acceptors were diminished, the regioselectivity in the difunctionalization reactions remained high. The sterically hindered trisubstituted alkene prop-1-ene-1,1-diyldibenzene was coupled with RAEs (2u and 2v) and afforded products 4ux and 4vx in 63% and 52% yields, respectively. However, other mono-alkyl-substituted alkenes and enol ethers, like allylbenzene, ethoxyethane, (allyloxy)benzene and 2-allylisoindoline-1,3-dione led to no reaction, despite increasing the catalyst loading to 20 mol% and extending the reaction time to 72 h.

### Reaction scope of the aldimine

We next studied the scope of aldimines, which are derived from commercially available benzaldehyde derivatives. Imines bearing neutral, electron-donating and electronegative substituents (1b–1g) coupled in this cascade reaction in 74–95% yields. Notably, sterically demanding fragments 2-tolyl (1h) and 1-napthyl (1i) were accommodated to give products in 62% and 70% yields, respectively. Heterocyclic imines (1j, 1k, 1l) furnished the corresponding coupling products in 85–91% yields. Alkyl imines could also be used as good coupling partners, and the products (4mm–4rm) were obtained in 60–76% yield (Table 3).

**Table 3:** Scope of aldimines^a^

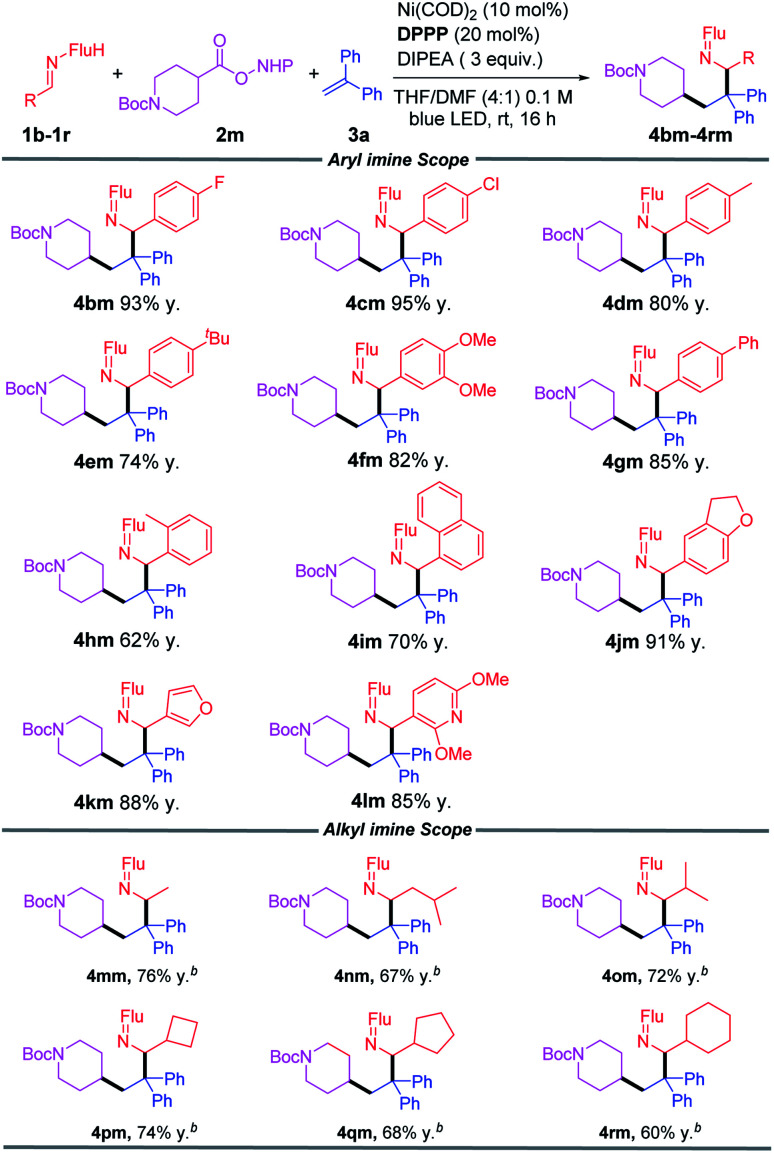

a Reactions conducted on a 1 mmol scale using 1 equiv. 1, 1.5 equiv. 2m and 3 equiv. 3a at 0.1 M. Isolated yields after chromatographic purification. Flu = 9-fluorenyl.

b 3 equiv. of DBU as base.

### Gram scale synthesis and ketimine hydrolysis

**Scheme 4:**
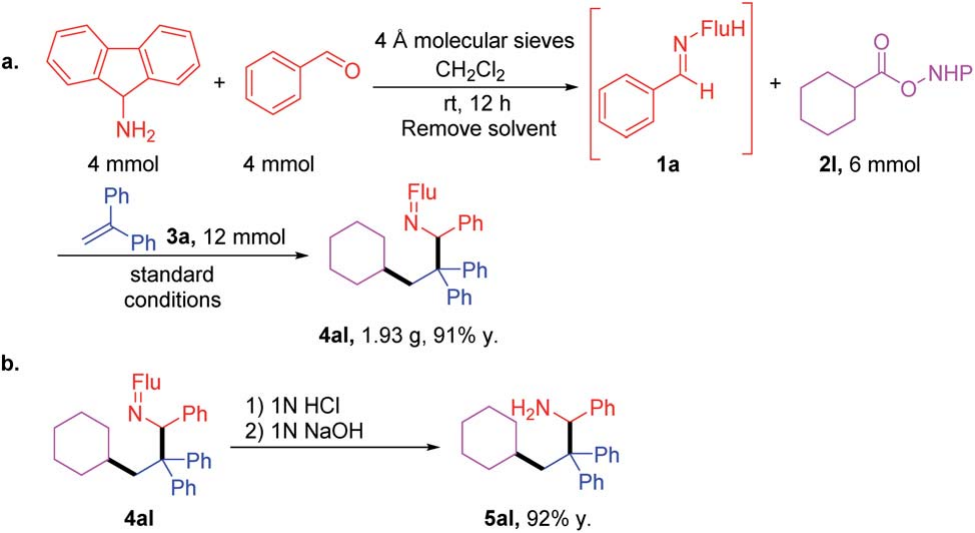
(a) Gram-scale one-pot synthesis of 4al through a telescoped imine synthesis and cascade coupling. (b) Ketimine hydrolysis.

After demonstrating the generality of this cascade amine synthesis, the scalability of this protocol was tested by a telescoped reaction. First, imine was synthesized using 4 mmol each of amine and aldehyde. After removal of the solvent, the crude imine was combined with RAE 2l and the cascade coupling was performed to give 1.93 g of 4al (91% over 2 steps). Hydrolysis with 1 M HCl in THF furnished amine 5al in 92% yield (Scheme 4).

### Attempts to develop an enantioselective version

**Scheme 5:**
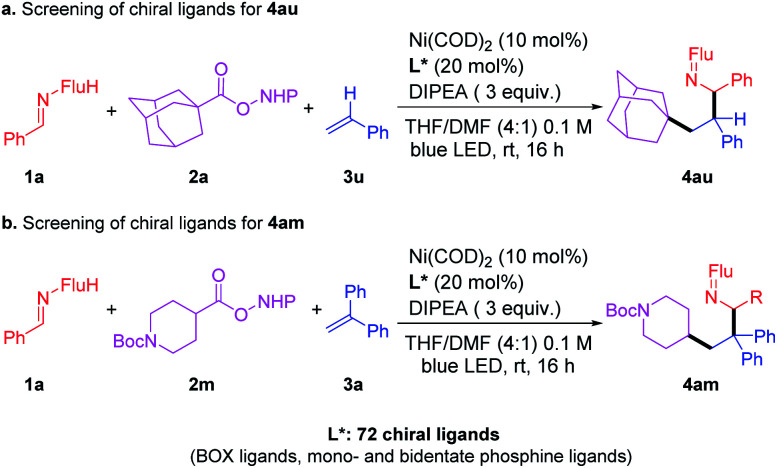
Attempts to develop an enantioselective catalyst.

Toward the identification of an enantioselective version of this three-component coupling reaction, we examined RAE 2a and 2m (Scheme 5) with a total of 72 enantioenriched ligands that are known to bind well to nickel (including BOX ligands and mono- and bidentate phosphine ligands, see ESI† for full details). Of these 72 reactions, 52 of them exhibited >10% isolated yields of the products under the standard (unoptimized) conditions. Analysis of the ee of the products of these 52 reactions led to a surprising result–*all of the products were racemic*. This observation suggests that the nickel catalyst is not involved in the stereochemistry determining step in these reactions. Based on this finding, we propose that this reaction *does not involve a reductive elimination from the Ni catalysts*. Furthermore, it is proposed that the nickel catalyst does not oxidatively trap intermediates in this process.

### Proposed mechanism

**Fig. 1:**
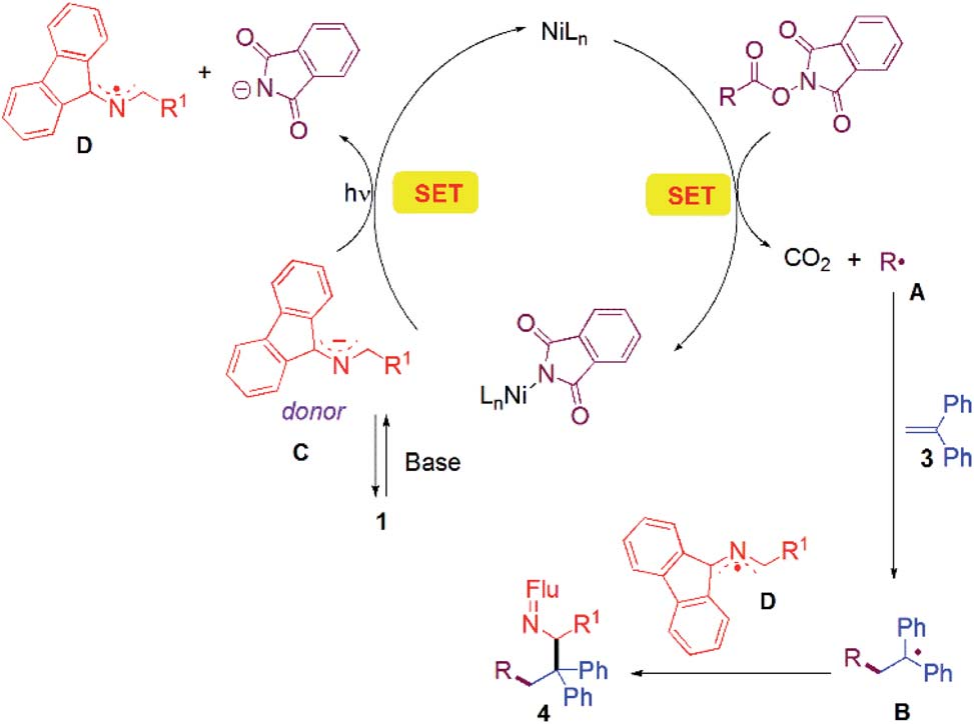
Proposed reaction pathway.

A working mechanism is provided based on literature precedence and observations made herein. A more detailed mechanistic study will be the subject of future work. In most investigations where redox-active-esters were used as alkyl coupling partners, alkyl radicals were proposed to be trapped by transition metal species. The second coupling partner generally joins the catalytic cycle through transmetallation from an organometallic reagent^51,86–88^ or through oxidative addition of aryl halide electrophiles.^52,89,90^ In contrast, in our cascade reaction (Fig. 1), we propose that the nickel catalyst is responsible for SET to the RAE to liberate CO_2_ and the alkyl radical A. Alkyl radical A adds to the alkene trap to form a benzylic radical B. Meanwhile, the aldimine 1 is deprotonated by the base (DIPEA) to give the 2-azaallyl anion C. Anion C reduces the Ni catalyst *via* SET with formation of the 2-azaallyl radical D. As noted earlier, excitation of the 2-azaallyl anion C with light increases its reducing power. The benzylic radical B and persistent 2-azaallyl radical D undergo a radical–radical coupling to generate product 4 without the participation of the nickel catalyst. Significantly, the relative reactivities of the three radical intermediates, the alkyl radical (A), the semi-stabilized benzylic radical (B) and the persistent 2-azaallyl radical (D), make possible the unique tandem reaction over undesired radical dimerizations or HAT quenching processes.

**Fig. 2:**
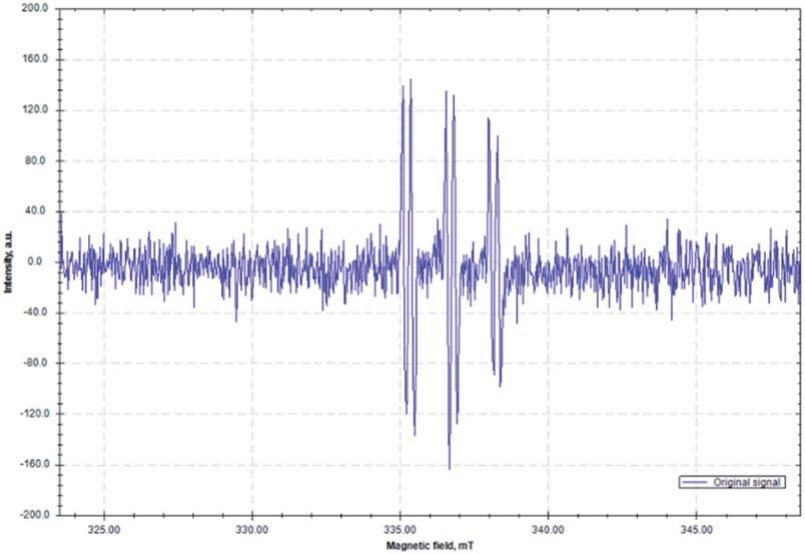
Isotropic X-band EPR spectrum of the PBN-trapped carbon centered radical (*T* = 298 K; microwave frequency: 9.440904 GHz; power: 0.2 mW; modulation amplitude: 100 μT).

To further support the proposed mechanistic pathway of this radical cascade reaction, an electron paramagnetic resonance (EPR) spectroscopy study was conducted (Fig. 2). A mixture of imine 1a, RAE 2m, 1,1-diphenylethylene 3a, Ni(COD)_2_/DPPP and DIPEA in degassed dry DMA in the presence of phenyl-*N-tert*-butylnitrone (PBN, a free-radical spin-trapping agent) was stirred under an argon atmosphere with irradiation by a commercially available blue LED for 10 min. A distinct signal of a trapped alkyl radical was observed (*g* = 2.0075, *A*_N_ = 14.51 G, *A*_H_ = 2.75 G). These results are similar to other reported PBN-trapped carbon centered radicals^91–93^ and support our contention that radical intermediates are involved in this process.

## Conclusions

In summary, we have developed a novel cascade strategy by merging nickel catalysis with RAEs in the presence of 2-azaallyl anions and alkenes. Alkene difunctionalization products are obtained with high regioselectivities. Despite generating three distinct radical species, excellent chemoselectivity is observed in bond formation processes leading to alkene difuctionalization products. The high functional group tolerance of *N*-benzyl imines and RAEs and their intermediates enables the synthesis of α-branched amines bearing aryl or alkyl groups at the β- or γ-positions. Mechanistically, we propose the intermediacy of three distinct radicals that undergo sequential radical trapping and radical–radical coupling processes. Evidence has been provided that the role of the nickel catalyst is to reduce the RAE. Importantly, the nickel catalyst does not appear to be active in mediating the radical–radical coupling reactions. Based on these results, we speculate that nickel catalysts may not be involved in mediating C(sp^3^)–C(sp^3^) bond-formations in cases where the radicals are sterically hindered or the radicals are relatively stable. Further expansion of the scope of these reactions and investigation of the reaction mechanism are underway in our labs.

## Data availability

All the relevant data (including NMR, EPR spectrum and X-ray crystal data) are contained within the ESI.†

## Author contributions

X. Y. and S. D. conceived of the project. M. L., H. Z. and P. W. designed the experiments. S. D., Y. Z., L. W., J. C. and W. C. performed the research. X. Y., M. L. and P. W. wrote the manuscript.

## Conflicts of interest

There are no conflicts to declare.

## Supplementary Material

SC-013-D2SC00500J-s001

SC-013-D2SC00500J-s002
