# Supplementary material for: α-Branched amines through radical coupling with 2-azaallyl anions, redox active esters and alkenes
Source: Chem Sci. 2022 Mar 3;13(13):3740–7. doi: 10.1039/d2sc00500j (PMC8966660; doi:10.1039/d2sc00500j)

Electronic Supplementary Material (ESI) for Chemical Science.
This journal is © The Royal Society of Chemistry 2022

Electronic supplementary information (ESI)

α-Branched Amines through Radical Coupling with 2-Azaallyl Anions, Redox Active Esters and Alkenes

Shengzu Duan[a], Yujin Zi[a], Lingling Wang[a], Jielun Cong[a], Wen Chen[a], Minyan Li*[,b], Hongbin Zhang*[,a], Xiaodong Yang*[, a] and Patrick J. Walsh*[,b]

[a]Key Laboratory of Medicinal Chemistry for Natural Resource, Ministry of Education; Yunnan Provincial Center for Research & Development of Natural Products; School of Chemical Science and Technology, Yunnan University, Kunming, 650091, P. R. China. *e-mail:xdyang@ynu.edu.cn; zhanghb@ynu. edu.cn

[b]Roy and Diana Vagelos Laboratories, Penn/Merck Laboratory for High-Throughput Experimentation, Department of Chemistry, University of Pennsylvania, 231 South 34th Street, Philadelphia, Pennsylvania, 19104, USA. *e-mail: pwalsh@sas.upenn.edu; liminyan@sas.upenn.edu

TABLE OF CONTENT

1. General methods……………………………………………………………………..…....………..S2
2. Preparation of *N*-fluorenyl imines …………………………....……………………………...……....S2
3. Preparation of redox active esters……………………………………………………………….....S4
4. Preparation of alkenes………………………………………………………………......................S5
5. Photoinduced/Ni-catalyzed functionalization of alkenes with imines and redox active esters (Table 1): *Lab scale reaction optimization of ligands, Ni source, bases, solvents, ratio of* ***1a*** *with* ***2a*** *and reaction condition control*……………………………………………………………………………………S6
6. General procedure and characterization of photoinduced/Nickel-catalyzed functionalization of alkenes with imines and redox active esters (Tables 2 and 3) ……………………………………...….......S9
7. Gram-scale sequential one-pot imine synthesis/cascade coupling...S28
8. Hydrolysis of compound **4al**………………………………………...……………………..........S28
9. EPR experiments……………………………………………………………………………………..S29
10. Attempts to enantioselectivity experiments…………………………………………………......S30
11. X-ray crystal structure of compound **4ah**………………….………...S34
12. Supplementary references………………………………………………………………......……........S36
13. NMR spectra of the products………….…...…………………………………………………...S37

1. General methods

All air- and moisture-sensitive solutions and chemicals were handled under a nitrogen atmosphere of a glovebox and solutions were transferred via “Titan” brand pipettor. Anhydrous solvents, including THF (Tetrahydrofuran), DMF (*N*,*N*-Dimethylformamide) and DMA (*N*,*N*-Dimethylacetamide) were purchased from Sigma-Aldrich and used without purification. Unless otherwise stated, all reagents were commercially available and used as received without purification. Ni(COD)$_2$ was purchased from Sigma-Aldrich and used as received. Ligands were purchased from TCI and J&K. Other chemicals were obtained from Sigma-Aldrich, Acros, TCI and Alfa-Aesar. TLC was performed with Merck TLC Silica gel60 F$_{254}$ plates with detection under UV light at 254 nm. Silica gel (200-300 mesh, Qingdao) was used for flash chromatography. Deactivated silica gel was prepared by addition of 15 mL Et$_3$N to 1 L of silica gel. The products were purified with XDB-C$_{18}$ (9.4 × 250 mm, 5 μm) column on an Agilent HPLC 1260 system. ^{1}H and ^{13}C{^{1}H} NMR spectra were obtained using a Brüker DRX 400 or 600 spectrometer at 400 MHz and 100 MHz or 600 MHz and 150 MHz, respectively. Chemical shifts were reported in units of parts per million (ppm) downfield from tetramethylsilane (TMS), and all coupling constants were reported in hertz. The infrared (IR) spectra were measured on a Nicolet iS10 FTIR spectrometer with 4 cm^{-1} resolution and 32 scans between wavenumber of 4000 cm^{-1} and 400 cm^{-1}. High Resolution Mass spectra were taken on AB QSTAR Pulsar mass spectrometer. Melting points were obtained on a XT-4 melting-point apparatus and were uncorrected.

2 Preparation of *N*-fluorenyl imines

(1) *Preparation of N-fluorenyl arylimine*：

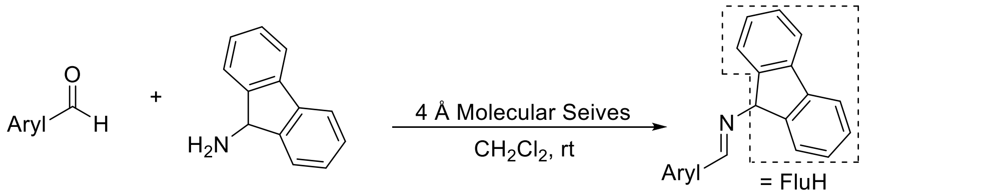

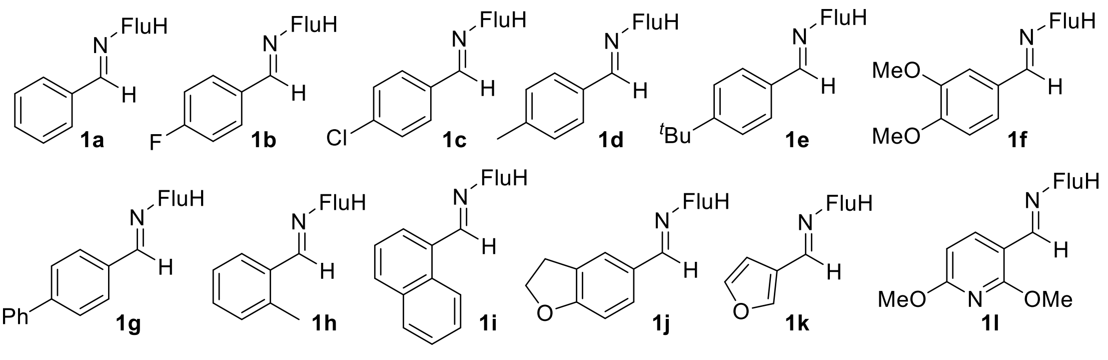

N-Fluorenyl arylimines(**1a**,[1] **1b**,[2] **1c**,[1] **1d**,[1] **1e**,[2] **1f**,[2] **1g**,[2] **1h**,[2] **1i**,[2] **1j**,[2] **1k**,[1] **1l**,[2]) were prepared following reported procedures.

To a solution of an aldehyde and 9*H*-fluoren-9-amine (1:1 mixture) in CH$_2$Cl$_2$ (0.2 M) at room temperature was added 4 Å molecular sieves (0.3 g/mmol). The mixture was stirred at room temperature until completion (ca. 12 h), as indicated by ^{1}H NMR, and filtered. The filtrate was concentrated and triturated with hexanes to give the corresponding *N*-fluorenyl imines as a solid that is sufficiently pure for further reactions.

(2) ***Preparation of N-fluorenyl alkylimines*：**

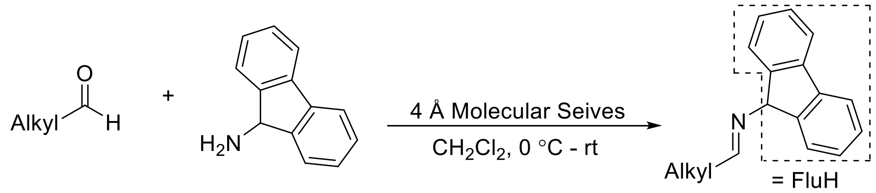

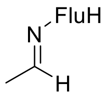
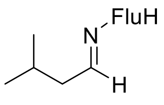
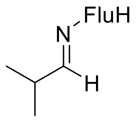
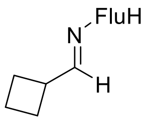
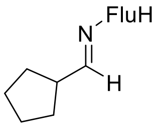
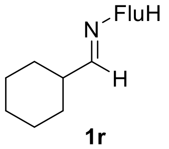

1m **1n** **1o** **1p** **1q** **1r**

N-Fluorenyl alkylimines(**1m-1o**) were prepared following reported procedures.[3]

Into an oven-dried reaction flask equipped with a magnetic stirring bar was added 9*H*-fluoren-9-amine (10 mmol) and 4 Å molecular sieves (0.3g/mmol). The flask was sealed with a rubber stopper, and connected to a Schlenk line through a needle. The flask was evacuated, and then refilled with nitrogen. This process was repeated twice, and the reaction flask was kept under a nitrogen atmosphere during the course of the reaction. CH_2Cl_2 (0.2 M) was added via syringe, and the resulting mixture was stirred at room temperature for 5 min before cooled in an ice-water bath. A solution of an aldehydes (10 mmol) in CH_2Cl_2 (2 mL) were added as a neat liquid. The reaction mixture was stirred in the ice-water bath for 0.5-1 h, then at room temperature for 1-4 h. As indicated by ^{1}H NMR, and filtered. The filtrate was concentrated and triturated with hexanes to give the corresponding *N*-fluorenyl imines are pure enough for further reactions.

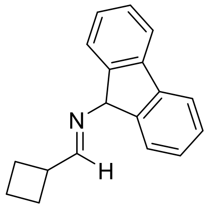

(*E*)-1-Cyclobutyl-*N*-(9*H*-fluoren-9-yl)methanimine (1p). ^{1}H NMR (400 MHz, Chloroform-*d*) δ 8.25 (d, *J* = 5.2 Hz, 1H), 7.74 (d, *J* = 7.6 Hz, 2H), 7.42 – 7.30 (m, 6H), 5.19 (s, 1H), 3.33 – 3.23 (m, 1H), 2.31 – 2.18 (m, 4H), 2.11 – 2.02 (m, 1H), 1.99 – 1.89 (m, 1H) ppm; **^{13}C{^{1}H} NMR** (100 MHz, Chloroform-*d*) δ 170.8, 145.0, 141.1, 128.4, 127.5, 125.1, 120.1, 74.3, 40.3, 25.8, 19.0 ppm; **IR** (thin film): 3039, 2951, 2864, 1623, 1488, 1435, 1302, 771, 697 cm^{-1}; **HRMS** calc'd for $C_{18}H_{18}N^+$ 248.1434, found 248.1438 [M+H]$^+$. **Mp**: 68 – 70 °C.

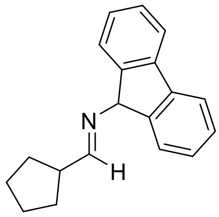

(*E*)-1-Cyclopentyl-*N*-(9*H*-fluoren-9-yl)methanimine (1q). ^{1}H NMR (400 MHz, Chloroform-*d*) δ 8.10 (d, *J* = 6.0 Hz, 1H), 7.74 (d, *J* = 7.6 Hz, 2H), 7.43 – 7.26 (m, 6H), 5.15 (s, 1H), 2.87 – 2.78 (m, 1H), 1.97 – 1.92 (m, 2H), 1.76 – 1.63 (m, 6H) ppm; **^{13}C{^{1}H} NMR** (100 MHz, Chloroform-*d*) δ 172.0, 145.0, 141.1, 128.4, 127.5, 125.1, 120.1, 74.4, 45.7, 30.6, 25.8 ppm; **IR** (thin film): 3060, 2925, 2850, 1623, 1482, 1438, 1310, 1025, 772, 696 cm^{-1}; **HRMS** calc'd for $C_{19}H_{20}N^+$ 262.1590, found 262.1588 [M+H]$^+$. **Mp**: 83 – 85 °C.

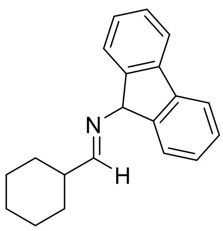

(*E*)-1-Cyclohexyl-*N*-(9*H*-fluoren-9-yl)methanimine (1r). ^{1}H NMR (400 MHz, Chloroform-*d*) δ 7.98 (d, *J* = 5.6 Hz, 1H), 7.66 (d, *J* = 7.6 Hz, 2H), 7.34 – 7.30 (m, 2H), 7.29 – 7.22 (m, 4H), 5.05 (s, 1H), 2.32 – 2.30 (m, 1H), 1.87 – 1.84 (m, 2H), 1.76 – 1.73 (m, 2H), 1.65 – 1.63 (m, 1H), 1.36 – 1.17 (m, 5H) ppm; **^{13}C{^{1}H} NMR** (100 MHz, Chloroform-*d*) δ 172.1, 144.9, 141.0, 128.2, 127.3, 124.9, 119.9,

74.4, 43.7, 29.9, 26.0, 25.4 ppm; **IR** (thin film): 3054, 2933, 2867, 1622, 1473, 1442, 1314, 1029, 700 cm^{-1}; **HRMS** calc'd for $C_{20}H_{22}N^+$ 276.1747, found 276.1747 $[M+H]^+$. **Mp**: 90 – 92 °C.

3 Preparation of redox active esters

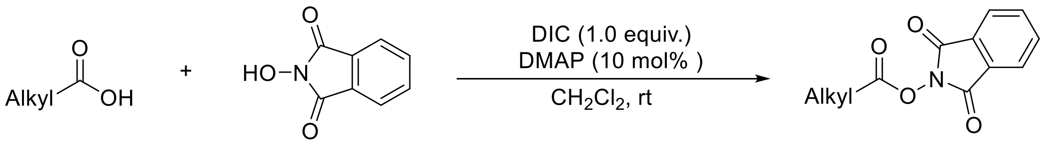

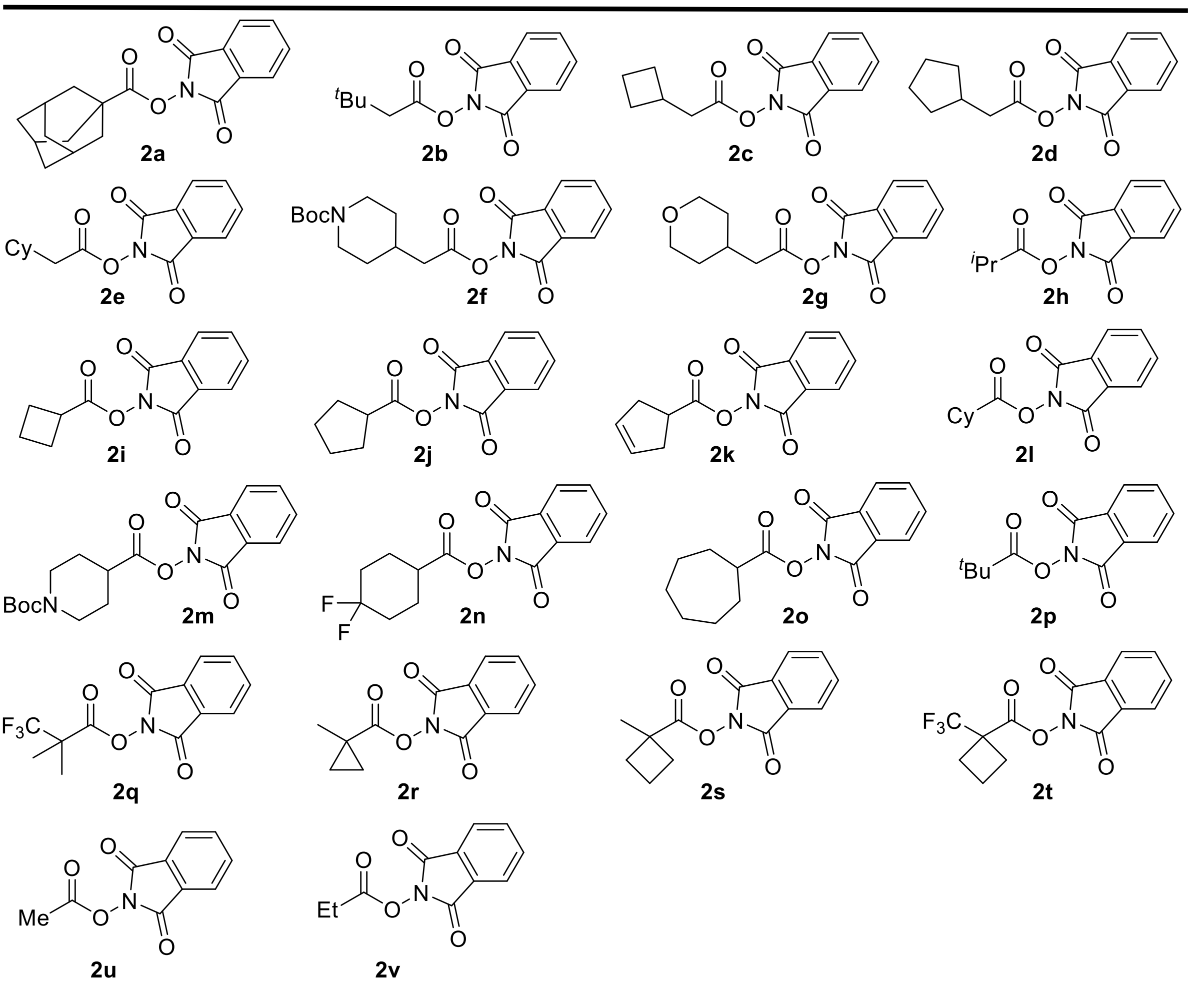

Redox active esters (**2a**,[4] **2b**,[5] **2c**,[6] **2d**,[6] **2e**,[5] **2f**,[7] **2g**,[8] **2h**,[4] **2i**,[4] **2j**,[4] **2k**,[9] **2l**,[4] **2m**,[10] **2n**,[4] **2o**,[4] **2p**,[9] **2q**,[11] **2r**,[9] **2s**,[12] **2t**,[12] **2u**,[13] **2v**,[13]) were prepared following reported procedures.

To a solution of 4-dimethylaminopyridine (DMAP, 122 mg, 1.0 mmol, 10 mol%) in CH_2Cl_2 (30 mL), *N*-hydroxyphthalimide (NHPI, 1.63 g, 10 mmol, 1.0 equiv.) and the corresponding acids (10 mmol, 1.0 equiv) were successively added. The solution was then allowed to stir vigorously at room temperature, and *N,N'*-diisopropylcarbodiimide (DIC, 1.26 g, 10 mmol, 1.0 equiv.) was added over 5 minutes by syringe. The reaction mixture was allowed to stir until the acid was consumed (monitored by TLC). The reaction mixture was then filtered, and the residue was washed by CH_2Cl_2. The organic phase was dried over anhydrous $NaSO_4$ and filtered. Silica gel was added, and the mixture was concentrated under reduced pressure. Purification of the residue by flash chromatography on silica gel using hexanes / ethyl acetate as eluent afforded the desired esters.

4. Preparation of alkenes

Alkenes (**3a, 3u** and **3v**) were purchased from Sigma-Aldrich and directly used. Alkenes (**3w** and **3x**) were prepared according to literature procedures.[14, 15]

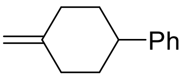

(4-Methylenecyclohexyl)benzene (3w). The reaction was performed following the literature procedures[14] with KO*t*Bu (2.53 g, 22.5 mmol) in dry Et_2O (30 mL) was added Methyltriphenylphosphonium bromide (8.04 g, 22.5 mmol) under N_2 at room temperature. The solution turned to yellow. The reaction mixture was stirred 45 min. 4-Phenylcyclohexanone (2.62 g, 15 mmol) in dry Et_2O (10 mL) was then canulated into the reaction mixture. It was then stirred 2 h at reflux. The reaction mixture was then diluted into Et_2O and was washed with water. The organic layer was then dried over $NaSO_4$. Filtered and concentrated under reduced pressure to afford a yellow oil. The crude mixture was then purified by flash chromatography on silica gel (Only hexanes) to give **3w** (2.32 g, 90% yield) as a colorless oil. The ^{1}H and ^{13}C{^{1}H} data for this compound match the literature data.[14]

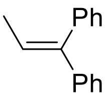

Prop-1-ene-1,1-diyldibenzene (3x). The reaction was performed following the literature procedures[15] with KO*t*Bu (3.45 g, 30 mmol) in dry THF (30 mL) was added Ethyltriphenylphosphonium bromide (11.17 g, 30 mmol) under N_2 at room temperature. The solution turned to yellow. The reaction mixture was stirred 45 min. Benzophenone (3.65 g, 20 mmol) in dry THF (10 mL) was then canulated into the reaction mixture. It was then stirred 8 h at 50 °C. The reaction mixture was then diluted into EtOAc and was washed with water. The organic layer was then dried over $NaSO_4$. Filtered and concentrated under reduced pressure to afford a white solid. The crude mixture was then purified by flash chromatography on silica gel (Only hexanes) to give **3x** (2.44 g, 63% yield) as a white solid. The ^{1}H and ^{13}C{^{1}H} data for this compound match the literature data.[15]

5. Photoinduced/Ni-catalyzed functionalization of alkenes with imines and redox active esters (Table 1): ***Lab scale reaction optimization of ligands, Ni source, bases, solvents,*** **ratio of 1a and 2a** ***and reaction condition control***

Table S1. Screening of ligands for photoinduced/Nickel-catalyzed functionalization of alkenes [a]

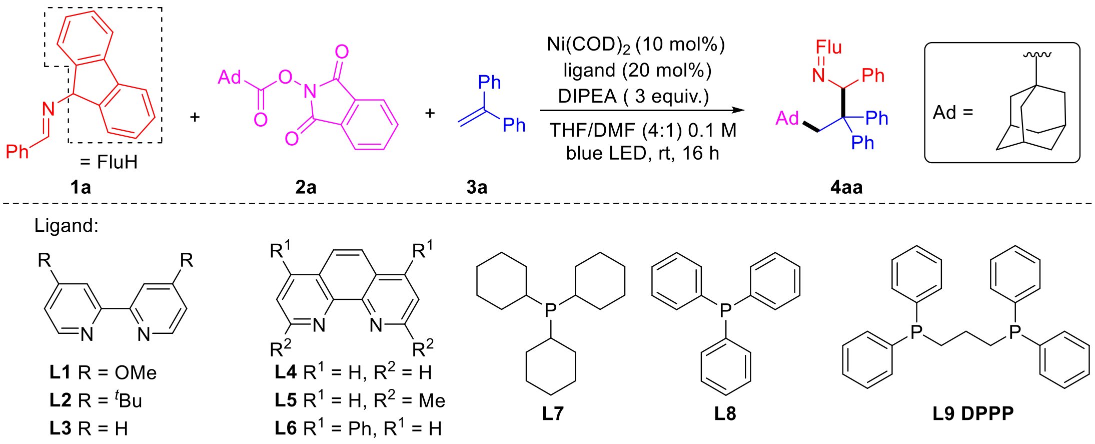

Entry	Ligand (20 mol%)	Yield (%) [b]
1	**L1**	88
2	**L2**	83
3	**L3**	82
4	**L4**	47
5	**L5**	40
6	**L6**	61
7	**L7**	36
8	**L8**	25
9	**L9**	90

[a]Reactions conducted on a 0.1 mmol scale using 1 equiv. of **1a**, 1.5 equiv. of **2a**, and 3 equiv. of **3a** $Ni(COD)_2$ (10 mol %), ligands (20 mol %) and DIPEA (3.0 equiv.) in 1 mL of THF/DMF = 4:1 at 25 °C for 16 h. [b]Yields were determined by ^1H NMR spectroscopy with $C_2H_2Cl_4$ as internal standard.

Table S2. Screening of Ni source for Photoinduced/Nickel-catalyzed functionalization of alkenes [a]

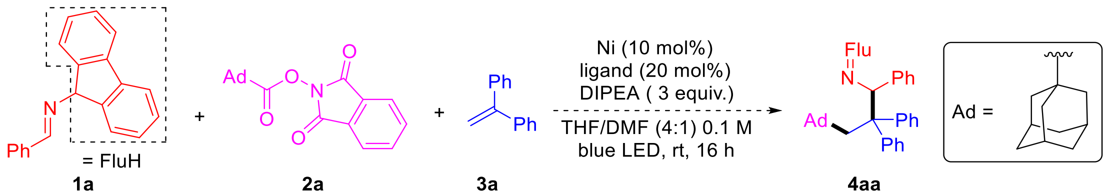

Entry	Ni source (10 mol%)	Ligand (20%)	Yield (%)[b]
1	$Ni(COD)_2$	DPPP	90
2	$NiCl_2$	DPPP	0
3	$NiBr_2$	DPPP	0
4	$NiBr_2$	bppy	0
5	$NiBr_2$·bppy (CAS:46389-47-3)	-	7

[a]Reactions conducted on a 0.1 mmol scale using 1 equiv. of **1a**, 1.5 equiv. of **2a**, and 3 equiv. of **3a** Ni (10 mol %), **DPPP** or **bppy** (20 mol %) and DIPEA (3.0 equiv.) in 1 mL of THF/DMF = 4:1 at 25 °C for

16 h. [b]Yields were determined by ^{1}H NMR spectroscopy with $C_2H_2Cl_4$ as internal standard.

Table S3. Screening of bases for photoinduced/Nickel-catalyzed functionalization of alkenes [a]

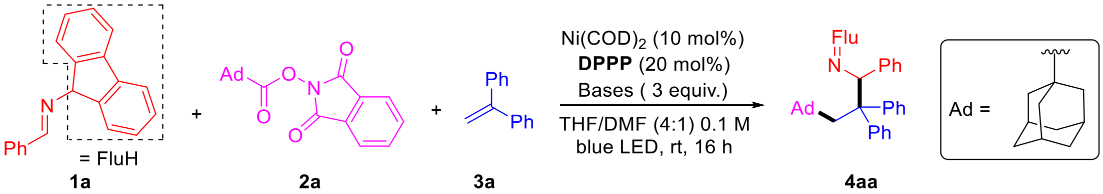

Entry	Bases (3 equiv.)	Yield (%) [b]
1	Et_3N	61
2	DIPEA	90
3	TMG	<5
4	DBU	42
5	Cs_2CO_3	0
6	LiO^{t}Bu	15

[a]Reactions conducted on a 0.1 mmol scale using 1 equiv. of **1a**, 1.5 equiv. of **2a**, and 3 equiv. of **3a** $Ni(COD)_2$ (10 mol %), **DPPP** (20 mol %) and Bases (3.0 equiv.) in 1 mL of THF/DMF = 4:1 at 27 °C for 16 h. [b]Yields were determined by ^{1}H NMR spectroscopy with $C_2H_2Cl_4$ as internal standard.

Table S4. Screening of solvents for photoinduced/Nickel-catalyzed functionalization of alkenes [a]

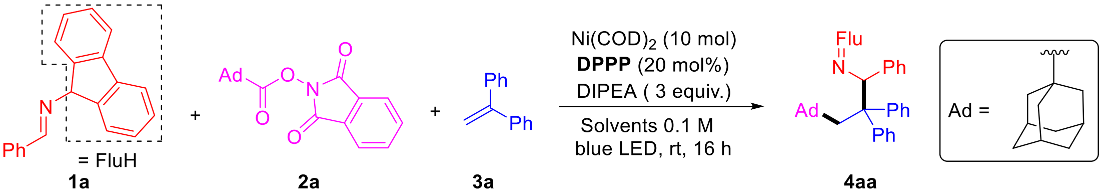

Entry	Solvent (0.1 M)	Yield (%) [b]
1	DMF	75
2	THF/DMF = 4:1	90
3	DMA	85

[a]Reactions conducted on a 0.1 mmol scale using 1 equiv. of **1a**, 1.5 equiv. of **2a**, and 3 equiv. of **3a** $Ni(COD)_2$ (10 mol %), **DPPP** (20 mol %) and DIPEA (3.0 equiv.) in 1 mL of THF/DMF = 4:1 at 27 °C for 16 h. [b]Yields were determined by ^{1}H NMR spectroscopy with $C_2H_2Cl_4$ as internal standard.

Table S5. Screening of ratio of 1a with 2a for photoinduced/Nickel-catalyzed functionalization of alkenes[a]

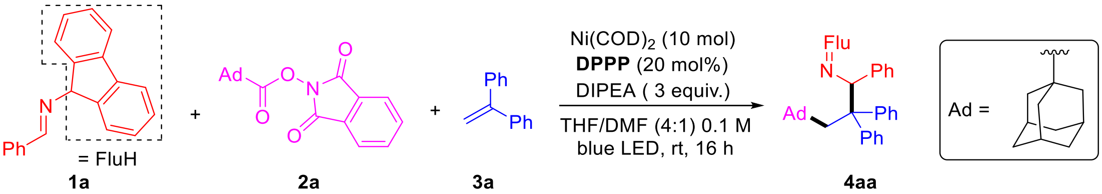

Entry	1a/2a	Yield (%) [b]
1	1:1	87
2	1.5:1	63
3	1:1.5	90

[a]Reactions conducted on a 0.1 mmol scale and 3 equiv. of **3a** Ni(COD)$_2$ (10 mol %), **DPPP** (20 mol %) and DIPEA (3.0 equiv.) in 1 mL of THF/DMF = 4:1 at 27 °C for 16 h. [b]Yields were determined by ^{1}H NMR spectroscopy with $C_2H_2Cl_4$ as internal standard.

Table S6. Reaction condition control for photoinduced/Nickel-catalyzed functionalization of alkenes[a]

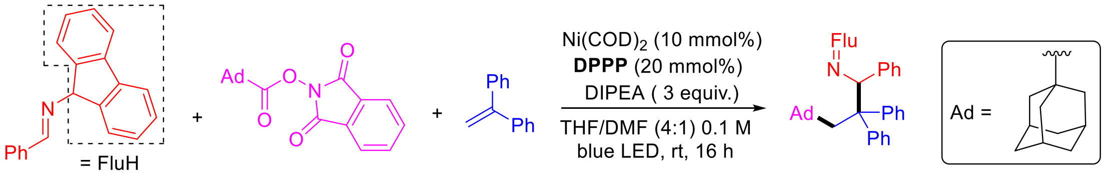

1a **2a** **3a** **4aa**

Entry	variation from the optimal conditions	Yield (%) [b]
1	none	90(86) [c]
2	no light	67
3	no Ni(COD)$_2$	0

[a]Reactions conducted on a 0.1 mmol scale using 1 equiv. of **1a**, 1.5 equiv. of **2a**, and 3 equiv. of **3a** Ni(COD)$_2$ (10 mol %), **DPPP** (20 mol %) and DIPEA (3.0 equiv.) in 1 mL of THF/DMF = 4:1 at 27 °C for 16 h. [b]Yields were determined by ^{1}H NMR spectroscopy with $C_2H_2Cl_4$ as internal standard. [c]Isolated yield of **4aa** after chromatographic purification.

6. General procedure and characterization of photoinduced/Nickel-catalyzed functionalization of alkenes with imines and redox active esters (Tables 2 and 3)

General procedure for the Photoinduced/Ni-catalyzed functionalization of alkenes with imines and redox active esters:

An oven-dried 30 mL reaction vial equipped with a stir bar was charged with Ni(COD)$_2$ (27.5 mg, 0.1 mmol, 10 mol %), **DPPP** (82.5 mg, 0.2 mmol, 20 mol %), imines (**1**, 1.0 mmol, 1.0 equiv) and redox active esters (**2**, 1.5 mmol, 1.5 equiv) in a glove box under a nitrogen atmosphere at room temperature, degassed THF (8 mL), DMF (2 mL) and alkenes (**3**, 3.0 mmol, 3.0 equiv) were sequentially added via syringe, the mixture was stirred and until all the solids were dissolved. Then, DIPEA (387.7 mg, 3.0 mmol, 3.0 equiv) was added to the reaction mixture. The vial was capped, removed from the glove box, and stirred for 16 h with blue LEDs whereby the temperature was maintained at approximately 27 ^{o}C via cooling with a fan. The lid of the reaction vial was opened, and quenched with 4 mL of H_2O. The reaction mixture was diluted with CH_2Cl_2 (50 mL). The organic layer was washed with water (3×50 mL) and dried over anhydrous $MgSO_4$. After filtration and concentration, the crude material was loaded onto a silica gel column and purified by flash chromatography.

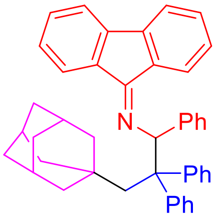

N*-(3-((3r,5r,7r)-Adamantan-1-yl)-1,2,2-triphenylpropyl)-9*H*-fluoren-9-imine (4aa):** The reaction was performed following the general procedure with (*E*)-*N*-(9*H*-fluoren-9-yl)-1-phenylmethanimine **1a** (269.1 mg, 1.0 mmol), 1,3-dioxoisoindolin-2-yl (3r,5r,7r)-adamantane-1-carboxylate **2a** (487.7 mg, 1.5 mmol) and ethene-1,1-diyldibenzene **3a** (540.8 mg, 3.0 mmol). The crude product was separated by flash chromatography on deactivated silica gel (hexanes:ethyl acetate = 200:1). Further purification by recrystallization (hexanes:MeOH = 10:1) give the product **4aa** (501.7 mg, 86% yield) as a yellow solid. **Mp**: 136 – 138 ^{o}C. **R*f = 0.42 (hexanes:ethyl acetate = 80:1). **^{1}H NMR** (400 MHz, Chloroform-*d*) 7.88 (d, *J* = 7.6 Hz, 1H), 7.61 (d, *J* = 7.6 Hz, 1H), 7.62 – 7.38 (m, 4H), 7.27 – 7.11 (m, 9H), 7.01 – 6.92 (m, 4H), 6.86 (t, *J* = 7.6 Hz, 2H), 6.69 – 6.56 (m, 2H), 6.48 (s, 1H), 2.07 (s, 2H), 1.64 – 1.57 (m, 3H), 1.43 – 1.40 (m, 3H), 1.34 – 1.31 (m, 3H), 1.22 – 1.12 (m, 6H) ppm; **^{13}C{^{1}H} NMR** (100 MHz, Chloroform-*d*) δ 161.4, 145.2, 144.1, 143.5, 141.3, 140.7, 139.3, 132.2, 131.6, 131.0, 130.5, 130.0, 128.2, 128.0, 127.3, 126.8, 126.61, 126.56, 126.0, 125.6, 122.9, 120.3, 119.1, 70.6, 57.0, 52.5, 43.9, 36.9, 34.7, 29.0 ppm, two resonances were not observed due to overlapping peaks; **IR** (thin film): 2901, 2847, 1646, 1449, 1275, 1102, 913, 748, 654 cm^{-1}; **HRMS** calc'd for $C_{44}H_{42}N^{+}$ 584.3312, found 584.3317 [M+H]$^{+}$.

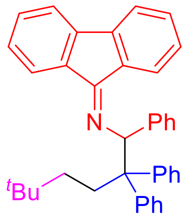

N*-(5,5-Dimethyl-1,2,2-triphenylhexyl)-9*H*-fluoren-9-imine (4ab):** The reaction was performed following the general procedure with (*E*)-*N*-(9*H*-fluoren-9-yl)-1-phenylmethanimine **1a** (269.1 mg, 1.0 mmol), 1,3-dioxoisoindolin-2-yl 3,3-dimethylbutanoate **2b** (391.7 mg, 1.5 mmol) and ethene-1,1-diyldibenzene **3a** (540.8 mg, 3.0 mmol). The crude product was separated by flash chromatography on deactivated silica gel (hexanes:ethyl acetate = 200:1). Further purification by recrystallization (hexanes:MeOH = 10:1) give the product **4ab** (472.6 mg, 91% yield) as a yellow solid. **Mp**: 195 – 197 ^{o}C. **R*f = 0.41 (hexanes:ethyl acetate = 80:1). **^{1}H NMR** (400 MHz, Chloroform-*d*) 7.88 (d, *J* = 8.0 Hz, 1H), 7.63 (d, *J* = 7.2 Hz, 1H), 7.50 (d, *J* = 7.6 Hz, 1H), 7.40 (d, *J* = 7.6 Hz, 1H), 7.30 – 7.12 (m, 9H), 7.02 – 6.95 (m, 6H), 6.92 (t, *J* = 7.6 Hz, 2H), 6.69 (d, *J* = 7.2 Hz, 2H), 6.35 (s, 1H), 2.11 – 1.95 (m, 2H), 1.05 (td, *J* = 12.8, 4.8 Hz, 1H), 0.89 (td, *J* = 12.8, 4.8 Hz, 1H), 0.72 (s, 9H) ppm; **^{13}C{^{1}H}**

NMR (100 MHz, Chloroform-*d*) δ 160.6, 145.5, 143.1, 142.3, 140.0, 139.7, 138.3, 130.9, 130.5, 130.0, 129.5, 128.8, 128.6, 127.2, 127.0, 126.4, 126.1, 125.8, 125.7, 125.0, 124.9, 124.2, 121.9, 119.2, 118.0, 67.8, 55.9, 37.1, 32.7, 29.4, 28.4 ppm; **IR** (thin film): 2956, 1646, 1449, 1275, 1081, 913, 748, 689 cm^{-1}; **HRMS** calc'd for $C_{39}H_{38}N^+$ 520.2999, found 520.3002 $[M+H]^+$.

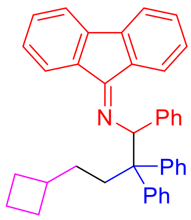

***N*-(4-Cyclobutyl-1,2,2-triphenylbutyl)-9*H*-fluoren-9-imine (4ac):** The reaction was performed following the general procedure with (*E*)-*N*-(9*H*-fluoren-9-yl)-1-phenylmethanimine **1a** (269.1 mg, 1.0 mmol), 1,3-dioxoisoindolin-2-yl 2-cyclobutylacetate **2c** (388.6 mg, 1.5 mmol) and ethene-1,1-diyldibenzene **3a** (540.8 mg, 3.0 mmol). The crude product was separated by flash chromatography on deactivated silica gel (hexanes:ethyl acetate = 200:1). Further purification by recrystallization (hexanes:MeOH = 10:1) give the product **4ac** (475.9 mg, 92% yield) as a yellow solid. **Mp**: 171 – 173 °C. $\mathbf{R}_f$ = 0.35 (hexanes:ethyl acetate = 80:1). **^{1}H NMR** (400 MHz, Chloroform-*d*) 7.88 (d, *J* = 7.6 Hz, 1H), 7.62 (d, *J* = 7.2 Hz, 1H), 7.46 (d, *J* = 7.2 Hz, 1H), 7.37 (d, *J* = 7.6 Hz, 1H), 7.26 – 7.25 (m, 2H), 7.17 – 7.20 (m, 2H), 7.17 – 7.09 (m, 5H), 7.01 – 6.91 (m, 8H), 6.88 (d, *J* = 7.6 Hz, 2H), 6.36 (s, 1H), 2.09 – 2.02 (m, 1H), 1.93 – 1.87 (m, 4H), 1.70 – 1.59 (m, 2H), 1.44 – 1.26 (m, 3H), 1.05 – 0.96 (m, 1H) ppm; **^{13}C{^{1}H} NMR** (100 MHz, Chloroform-*d*) δ 160.6, 145.6, 143.0, 142.4, 139.9, 139.7, 138.3, 130.8, 130.5, 130.0, 129.5, 128.64, 128.57, 127.2, 126.9, 126.4, 126.2, 125.7, 124.9, 124.2, 121.9, 119.2, 118.0, 67.4, 55.7, 35.6, 35.4, 31.0, 27.2, 27.1, 17.5 ppm, two resonances were not observed due to overlapping peaks; **IR** (thin film): 3058, 2930, 2853, 1645, 1449, 1275, 1093, 913, 748, 653 cm^{-1}; **HRMS** calc'd for $C_{39}H_{36}N^+$ 518.2842, found 518.2847 $[M+H]^+$.

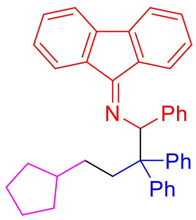

***N*-(4-Cyclopentyl-1,2,2-triphenylbutyl)-9*H*-fluoren-9-imine (4ad):** The reaction was performed following the general procedure with (*E*)-*N*-(9*H*-fluoren-9-yl)-1-phenylmethanimine **1a** (269.1 mg, 1.0 mmol), 1,3-dioxoisoindolin-2-yl 2-cyclopentylacetate **2d** (409.7 mg, 1.5 mmol) and ethene-1,1-diyldibenzene **3a** (540.8 mg, 3.0 mmol). The crude product was separated by flash chromatography on deactivated silica gel (hexanes:ethyl acetate = 200:1). Further purification by recrystallization (hexanes:MeOH = 10:1) give the product **4ad** (472.8 mg, 89% yield) as a yellow solid. **Mp**: 171 – 173 °C. $\mathbf{R}_f$ = 0.36 (hexanes:ethyl acetate = 80:1). **^{1}H NMR** (400 MHz, Chloroform-*d*) 7.87 (d, *J* = 7.6 Hz, 1H), 7.62 (d, *J* = 7.2 Hz, 1H), 7.43 (d, *J* = 7.2 Hz, 1H), 7.34 (d, *J* = 7.6 Hz, 1H), 7.29 – 7.27 (m, 2H), 7.22 – 7.08 (m, 7H), 7.01 – 6.89 (m, 8H), 6.88 (d, *J* = 7.6 Hz, 2H), 6.36 (s, 1H), 2.14 – 1.99 (m, 2H), 1.66 – 1.52 (m, 3H), 1.42 – 1.32 (m, 4H), 1.19 – 1.16 (m, 1H), 0.99 – 0.81 (m, 3H) ppm; **^{13}C{^{1}H} NMR** (100 MHz, Chloroform-*d*) δ 161.8, 146.7, 144.2, 143.5, 141.1, 140.8, 139.4, 132.0, 131.6, 131.1, 130.6, 129.84, 129.75, 128.3, 128.1, 127.5, 127.3, 126.9, 126.13, 126.10, 125.3, 123.0, 120.4, 119.2, 68.8, 57.2, 41.1, 38.4, 32.9, 32.7, 31.1, 25.2, 25.1 ppm, one resonance was not observed due to overlapping peaks; **IR** (thin film): 3058, 2947, 2847, 1646, 1449, 1310, 1101, 907, 731, 653, 607 cm^{-1}; **HRMS** calc'd for $C_{40}H_{38}N^+$ 532.2999, found 532.2998 $[M+H]^+$.

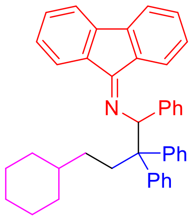

***N*-(4-Cyclohexyl-1,2,2-triphenylbutyl)-9*H*-fluoren-9-imine (4ae):** The reaction was performed following the general procedure with (*E*)-*N*-(9*H*-fluoren-9-yl)-1-phenylmethanimine **1a** (269.1 mg, 1.0 mmol), 1,3-dioxoisoindolin-2-yl 2-cyclohexylacetate **2e** (430.7 mg, 1.5 mmol) and ethene-1,1-diyldibenzene **3a** (540.8 mg, 3.0 mmol). The crude product was separated by flash chromatography on

deactivated silica gel (hexanes:ethyl acetate = 200:1). Further purification by recrystallization (hexanes:MeOH = 10:1) give the product **4ae** (490.8 mg, 90% yield) as a yellow solid. **Mp**: 171 – 173 ^{o}C. **R$_f$** = 0.36 (hexanes:ethyl acetate = 80:1). **^{1}H NMR** (400 MHz, Chloroform-*d*) 8.05 (d, *J* = 7.6 Hz, 1H), 7.84 (d, *J* = 7.2 Hz, 1H), 7.66 (d, *J* = 7.2 Hz, 1H), 7.57 (d, *J* = 7.6 Hz, 1H), 7.51 – 7.49 (m, 2H), 7.45 – 7.30 (m, 7H), 7.24 – 7.11 (m, 8H), 6.90 (d, *J* = 8.8 Hz, 2H), 6.59 (s, 1H), 2.32 – 2.21 (m, 2H), 1.83 – 1.69 (m, 5H), 1.38 – 1.18 (m, 5H), 1.11 – 1.06 (m, 1H), 1.00 – 0.84 (m, 2H) ppm; **^{13}C{^{1}H} NMR** (100 MHz, Chloroform-*d*) δ 161.8, 146.7, 144.2, 143.5, 141.1, 140.8, 139.4, 132.0, 131.6, 131.1, 130.6, 129.83, 129.75, 128.3, 128.1, 127.5, 127.3, 126.9, 126.11, 126.09, 125.3, 123.0, 120.4, 119.2, 68.7, 57.1, 38.6, 36.5, 33.6, 33.5, 32.4, 26.8, 26.6, 26.5 ppm, one resonance was not observed due to overlapping peaks; **IR** (thin film): 3023, 2923, 1645, 1448, 1275, 1101, 913, 749, 653 cm^{-1}; **HRMS** calc'd for $C_{41}H_{40}N^{+}$ 546.3155, found 546.3156 [M+H]$^{+}$.

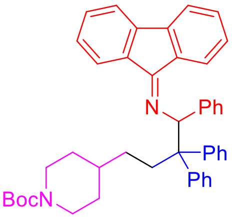

***tert*-Butyl 4-(4-((9*H*-fluoren-9-ylidene)amino)-3,3,4-triphenylbutyl)piperidine-1-carboxylate (4af):** The reaction was performed following the general procedure with (*E*)-*N*-(9*H*-fluoren-9-yl)-1-phenylmethanimine **1a** (269.1 mg, 1.0 mmol), *tert*-butyl 4-(2-((1,3-dioxoisoindolin-2-yl)oxy)-2-oxoethyl)piperidine-1-carboxylate **2f** (582.2 mg, 1.5 mmol) and ethene-1,1-diyldibenzene **3a** (540.8 mg, 3.0 mmol). The crude product was separated by flash chromatography on deactivated silica gel (hexanes:ethyl acetate = 10:1). Further purification by recrystallization (hexanes:MeCN = 10:1) give the product **4af** (542.9 mg, 84% yield) as a yellow solid. **Mp**: 180 – 182 ^{o}C. **R$_f$** = 0.36 (hexanes:ethyl acetate = 10:1). **^{1}H NMR** (400 MHz, Chloroform-*d*) 7.85 (d, *J* = 7.6 Hz, 1H), 7.61 (d, *J* = 7.6 Hz, 1H), 7.44 (d, *J* = 7.2 Hz, 1H), 7.36 (d, *J* = 7.2 Hz, 1H), 7.28 – 7.24 (m, 2H), 7.22 – 7.19 (m, 1H), 7.17 – 7.09 (m, 6H), 7.00 – 6.89 (m, 8H), 6.67 (d, *J* = 7.6 Hz, 2H), 6.34 (s, 1H), 4.03 – 3.80 (m, 2H), 2.55 – 2.47 (m, 2H), 2.09 – 2.01 (m, 2H), 1.54 – 1.50 (m, 1H), 1.45 – 1.42 (m, 1H), 1.34 (s, 9H), 1.18 – 0.99 (m, 2H), 0.91 – 0.83 (m, 3H) ppm; **^{13}C{^{1}H} NMR** (100 MHz, Chloroform-*d*) δ 160.7, 153.8, 145.2, 143.0, 142.0, 139.8, 139.7, 138.2, 130.7, 130.4, 130.0, 129.5, 128.6, 128.5, 127.2, 126.9, 126.3, 126.2, 125.81, 125.78, 125.1, 125.0, 124.3, 121.8, 119.2, 118.1, 78.1, 67.7, 55.9, 42.9, 35.7, 35.0, 31.2, 30.4, 27.4 ppm, one resonance was not observed due to overlapping peaks; **IR** (thin film): 2929, 1685, 1449, 1276, 1163, 913, 769, 653 cm^{-1}; **HRMS** calc'd for $C_{45}H_{47}N_2O_2^{+}$ 647.3632, found 647.3630 [M+H]$^{+}$.

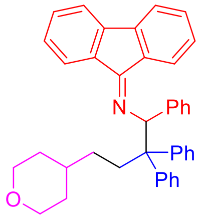

***N*-(1,2,2-Triphenyl-4-(tetrahydro-2*H*-pyran-4-yl)butyl)-9*H*-fluoren-9-imine (4ag):** The reaction was performed following the general procedure with (*E*)-*N*-(9*H*-fluoren-9-yl)-1-phenylmethanimine **1a** (269.1 mg, 1.0 mmol), 1,3-dioxoisoindolin-2-yl 2-(tetrahydro-2*H*-pyran-4-yl)acetate **2g** (433.7 mg, 1.5 mmol) and ethene-1,1-diyldibenzene **3a** (540.8 mg, 3.0 mmol). The crude product was separated by flash chromatography on deactivated silica gel (hexanes:ethyl acetate = 20:1). Further purification by recrystallization (hexanes:MeOH = 10:1) give the product **4ag** (394.0 mg, 72% yield) as a yellow solid. **Mp**: 163 – 165 ^{o}C. **R$_f$** = 0.36 (hexanes:ethyl acetate = 10:1). **^{1}H NMR** (400 MHz, Chloroform-*d*) 7.85 (d, *J* = 7.6 Hz, 1H), 7.61 (d, *J* = 7.2 Hz, 1H), 7.41 (d, *J* = 7.2 Hz, 1H), 7.32 (d, *J* = 7.2 Hz, 1H), 7.28 – 7.25 (m, 2H), 7.21 – 7.06 (m, 7H), 7.02 – 6.88 (m, 8H), 6.67 (d, *J* = 7.2 Hz, 2H), 6.35 (s, 1H), 3.79 – 3.74 (m, 2H), 3.17 – 3.14 (m, 2H), 2.12 – 1.99 (m, 2H), 1.46 – 1.42 (m, 1H), 1.38 – 1.33 (m, 1H), 1.22 – 1.19 (m, 1H), 1.15 – 1.02 (m, 3H), 1.00 – 0.92 (m, 1H) ppm; **^{13}C{^{1}H} NMR** (100 MHz, Chloroform-*d*) δ 160.7, 145.2, 143.0, 142.0, 139.8, 139.7, 138.2, 130.7, 130.4, 130.0,

129.5, 128.6, 128.5, 127.2, 126.9, 126.3, 126.2, 125.80, 125.77, 125.1, 125.0, 124.3, 121.8, 119.2, 118.0, 67.8, 67.0, 55.9, 34.7, 34.6, 32.14, 32.07, 30.8 ppm, one resonance was not observed due to overlapping peaks; **IR** (thin film): 3057, 2930, 2844, 1645, 1449, 1261, 1092, 913, 748, 653 cm^{-1}; **HRMS** calc'd for $C_{40}H_{38}N^+$ 548.2948, found 548.2949 $[M+H]^+$.

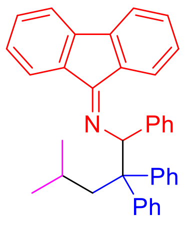

N*-(4-Methyl-1,2,2-triphenylpentyl)-9*H*-fluoren-9-imine (4ah):** The reaction was performed following the general procedure with (*E*)-*N*-(9*H*-fluoren-9-yl)-1-phenylmethanimine **1a** (269.1 mg, 1.0 mmol), 1,3-dioxoisoindolin-2-yl isobutyrate **2h** (349.6 mg, 1.5 mmol) and ethene-1,1-diyldibenzene **3a** (540.8 mg, 3.0 mmol). The crude product was separated by flash chromatography on deactivated silica gel (hexanes:ethyl acetate = 200:1). Further purification by recrystallization (hexanes:MeOH = 10:1) give the product **4ah** (452.0 mg, 92% yield) as a yellow solid. **Mp**: 164 – 166 °C. **R*f = 0.43 (hexanes:ethyl acetate = 80:1). **^{1}H NMR** (400 MHz, Chloroform-*d*) 7.89 (d, *J* = 7.6 Hz, 1H), 7.61 (d, *J* = 7.2 Hz, 1H), 7.41 – 7.30 (m, 4H), 7.20 – 7.06 (m, 9H), 7.00 – 6.86 (m, 6H), 6.66 (d, *J* = 7.2 Hz, 2H), 6.38 (s, 1H), 2.05 (dd, *J* = 14.0, 5.2 Hz, 1H), 1.89 (dd, *J* = 14.0, 5.2 Hz, 1H), 1.66 – 1.57 (m, 1H), 0.43 (d, *J* = 6.8 Hz, 3H), 0.41 (d, *J* = 6.8 Hz, 3H) ppm; **^{13}C{^{1}H} NMR** (100 MHz, Chloroform-*d*) δ 160.5, 145.0, 143.0, 142.2, 139.9, 139.6, 138.2, 130.8, 130.5, 129.9, 129.4, 128.9, 128.8, 127.1, 126.9, 126.3, 126.2, 125.7, 125.0, 124.4, 121.8, 119.2, 118.0, 68.8, 56.6, 46.5, 23.7, 23.6, 23.4 ppm, one resonance was not observed due to overlapping peaks; **IR** (thin film): 3023, 2953, 1644, 1449, 1276, 1101, 913, 749, 652 cm^{-1}; **HRMS** calc'd for $C_{37}H_{34}N^+$ 492.2686, found 492.2690 $[M+H]^+$.

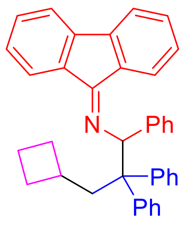

N*-(3-Cyclobutyl-1,2,2-triphenylpropyl)-9*H*-fluoren-9-imine (4ai):** The reaction was performed following the general procedure with (*E*)-*N*-(9*H*-fluoren-9-yl)-1-phenylmethanimine **1a** (269.1 mg, 1.0 mmol), 1,3-dioxoisoindolin-2-yl cyclobutanecarboxylate **2i** (367.6 mg, 1.5 mmol) and ethene-1,1-diyldibenzene **3a** (540.8 mg, 3.0 mmol). The crude product was separated by flash chromatography on deactivated silica gel (hexanes:ethyl acetate = 200:1). Further purification by recrystallization (hexanes:MeOH = 10:1) give the product **4ai** (422.7 mg, 84% yield) as a yellow solid. **Mp**: 178 – 180 °C. **R*f = 0.41 (hexanes:ethyl acetate = 80:1). **^{1}H NMR** (400 MHz, Chloroform-*d*) 7.89 (d, *J* = 7.6 Hz, 1H), 7.62 (d, *J* = 7.2 Hz, 1H), 7.43 (d, *J* = 7.2 Hz, 1H), 7.34 (d, *J* = 7.2 Hz, 1H), 7.29 – 7.26 (m, 2H), 7.23 – 7.06 (m, 7H), 7.03 – 7.01 (m, 2H), 6.98 – 6.87 (m, 6H), 6.65 (d, *J* = 7.2 Hz, 2H), 6.40 (s, 1H), 2.41 – 2.34 (m, 1H), 2.26 – 2.12 (m, 2H), 1.62 – 1.37 (m, 3H), 1.28 – 1.13 (m, 2H), 1.11 – 1.01 (m, 1H) ppm; **^{13}C{^{1}H} NMR** (100 MHz, Chloroform-*d*) δ 160.7, 144.8, 143.0, 142.3, 139.9, 139.6, 138.2, 130.8, 130.5, 130.0, 129.4, 128.73, 128.67, 127.1, 126.9, 126.3, 126.2, 125.71, 125.65, 125.0, 124.9, 124.2, 121.8, 119.2, 118.0, 67.5, 55.6, 45.6, 32.0, 29.0, 28.8, 18.0 ppm; **IR** (thin film): 3005, 2988, 1645, 1449, 1276, 1079, 913, 749, 653 cm^{-1}; **HRMS** calc'd for $C_{38}H_{34}N^+$ 504.2686, found 504.2690 $[M+H]^+$.

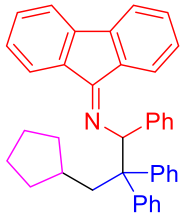

***N*-(3-Cyclopentyl-1,2,2-triphenylpropyl)-9*H*-fluoren-9-imine (4aj):** The reaction was performed following the general procedure with (*E*)-*N*-(9*H*-fluoren-9-yl)-1-phenylmethanimine **1a** (269.1 mg, 1.0 mmol), 1,3-dioxoisoindolin-2-yl cyclopentanecarboxylate **2j** (388.6 mg, 1.5 mmol) and ethene-1,1-diyldibenzene **3a** (540.8 mg, 3.0 mmol). The crude product was separated by flash chromatography on deactivated silica gel (hexanes:ethyl acetate = 200:1). Further purification by

recrystallization (hexanes:MeOH = 10:1) give the product **4aj** (419.0 mg, 81% yield) as a yellow solid. **Mp**: 161 – 163 °C. **R*f*** = 0.40 (hexanes:ethyl acetate = 80:1). **^{1}H NMR** (400 MHz, Chloroform-*d*) 8.06 (d, *J* = 7.6 Hz, 1H), 7.79 (d, *J* = 7.2 Hz, 1H), 7.64 (d, *J* = 7.6 Hz, 1H), 7.55 (d, *J* = 7.6 Hz, 1H), 7.52 – 7.49 (m, 2H), 7.44 – 7.36 (m, 2H), 7.35 – 7.27 (m, 5H), 7.25 – 7.22 (m, 2H), 7.18 – 7.11 (m, 4H), 7.07 (t, *J* = 7.2 Hz, 2H), 6.84 (d, *J* = 7.2 Hz, 2H), 6.57 (s, 1H), 2.32 (d, *J* = 5.2 Hz, 2H), 1.97 – 1.89 (m, 1H), 1.56 – 1.26 (m, 5H), 1.15 – 1.08 (m, 1H), 0.90 – 0.86 (m, 1H), 0.80 - 0.70 (m, 1H) ppm; **^{13}C{^{1}H} NMR** (100 MHz, Chloroform-*d*) δ 160.6, 145.3, 143.1, 142.4, 139.9, 139.7, 138.3, 130.9, 130.5, 130.0, 129.5, 129.0, 128.8, 127.2, 126.9, 126.4, 126.3, 125.7, 125.0, 124.3, 121.9, 119.2, 118.0, 68.2, 56.3, 44.6, 35.7, 33.5, 33.1, 23.9, 23.7 ppm, two resonances were not observed due to overlapping peaks; **IR** (thin film): 3006, 2989, 1648, 1449, 1275, 1080, 897, 749, 701 cm^{-1}; **HRMS** calc'd for $C_{39}H_{36}N^+$ 518.2842, found 518.2838 [M+H]$^+$.

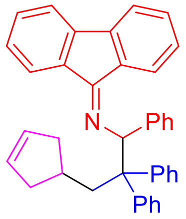

N*-(3-(Cyclopent-3-en-1-yl)-1,2,2-triphenylpropyl)-9*H*-fluoren-9-imine (4ak):** The reaction was performed following the general procedure with (*E*)-*N*-(9*H*-fluoren-9-yl)-1-phenylmethanimine **1a** (269.1 mg, 1.0 mmol), 1,3-dioxoisoindolin-2-yl cyclopent-3-ene-1-carboxylate **2k** (385.6 mg, 1.5 mmol) and ethene-1,1-diyldibenzene **3a** (540.8 mg, 3.0 mmol). The crude product was separated by flash chromatography on deactivated silica gel (hexanes:ethyl acetate = 200:1). Further purification by recrystallization (hexanes:MeOH = 10:1) give the product **4ak** (453.4 mg, 88% yield) as a yellow solid. **Mp**: 171 – 173 °C. **R*f = 0.34 (hexanes:ethyl acetate = 80:1). **^{1}H NMR** (400 MHz, Chloroform-*d*) 7.90 (d, *J* = 7.6 Hz, 1H), 7.63 (d, *J* = 7.2 Hz, 1H), 7.46 (d, *J* = 7.6 Hz, 1H), 7.37 (d, *J* = 7.6 Hz, 1H), 7.35 – 7.32 (m, 2H), 7.26 – 7.12 (m, 7H), 7.08 – 7.06 (m, 2H), 7.02 – 6.95 (m, 4H), 6.91 (t, *J* = 7.6 Hz, 2H), 6.68 (d, *J* = 7.6 Hz, 2H), 6.44 (s, 1H), 5.42 – 5.36 (m, 2H), 2.37 – 2.24 (m, 3H), 2.08 – 2.02 (m, 1H), 1.67 – 1.52 (m, 2H), 1.45 – 1.38 (m, 1H) ppm; **^{13}C{^{1}H} NMR** (100 MHz, Chloroform-*d*) δ 160.7, 145.1, 143.0, 142.3, 139.8, 139.7, 138.2, 130.9, 130.5, 130.0, 129.5, 129.3, 129.0, 128.7, 128.6, 127.2, 127.0, 126.38, 126.36, 125.7, 125.1, 125.0, 124.4, 121.9, 119.2, 118.0, 67.8, 56.2, 45.3, 39.6, 39.2, 33.9 ppm, one resonance was not observed due to overlapping peaks; **IR** (thin film): 3057, 2927, 1644, 1449, 1275, 1102, 913, 749, 652 cm^{-1}; **HRMS** calc'd for $C_{39}H_{34}N^+$ 516.2686, found 516.2685 [M+H]$^+$.

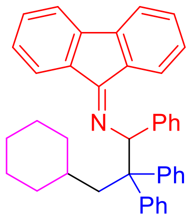

N*-(3-Cyclohexyl-1,2,2-triphenylpropyl)-9*H*-fluoren-9-imine (4al):** The reaction was performed following the general procedure with (*E*)-*N*-(9*H*-fluoren-9-yl)-1-phenylmethanimine **1a** (269.1 mg, 1.0 mmol), 1,3-dioxoisoindolin-2-yl cyclohexanecarboxylate **2l** (409.7 mg, 1.5 mmol) and ethene-1,1-diyldibenzene **3a** (540.8 mg, 3.0 mmol). The crude product was separated by flash chromatography on deactivated silica gel (hexanes:ethyl acetate = 200:1). Further purification by recrystallization (hexanes:MeOH = 10:1) give the product **4al** (467.5 mg, 88% yield) as a yellow solid. **Mp**: 158 – 160 °C. **R*f = 0.41 (hexanes:ethyl acetate = 80:1). **^{1}H NMR** (400 MHz, Chloroform-*d*) 7.90 (d, *J* = 7.6 Hz, 1H), 7.63 (d, *J* = 7.6 Hz, 1H), 7.46 (d, *J* = 7.6 Hz, 1H), 7.38 – 7.35 (m, 3H), 7.26 – 7.09 (m, 9H), 7.02 – 6.93 (m, 4H), 6.88 (t, *J* = 7.2 Hz, 2H), 6.66 (d, *J* = 7.2 Hz, 2H), 6.36 (s, 1H), 2.07 (dd, *J* = 16.0, 4.0 Hz, 1H), 1.81 (dd, *J* = 16.0, 4.0 Hz, 1H), 1.38 – 1.31 (m, 3H), 1.27 – 1.20 (m, 1H), 1.02 – 0.81 (m, 4H), 0.77 – 0.53 (m, 3H) ppm; **^{13}C{^{1}H} NMR** (100 MHz, Chloroform-*d*) δ 160.5, 145.1, 143.0, 142.0, 139.9, 139.7, 138.3, 130.8, 130.6, 129.9, 129.4, 128.9, 128.8, 127.2, 126.9, 126.3, 126.2, 125.7, 125.1, 125.0, 124.4, 121.8, 119.2, 118.0, 69.3, 56.9, 45.3, 34.2, 34.1, 33.0, 25.6, 25.5, 25.4 ppm, one resonance was not observed due to overlapping peaks; **IR** (thin film): 2923, 1686, 1449, 1276, 1165, 913,

748, 653 cm^{-1}; **HRMS** calc'd for $C_{40}H_{38}N^{+}$ 532.2999, found 532.2994 $[M+H]^{+}$.

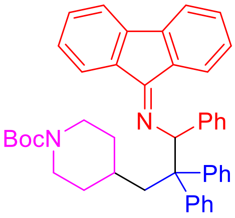

tert*-Butyl4-(3-((9*H*-fluoren-9-ylidene)amino)-2,2,3-triphenylpropyl)piperidine-1-carboxylate (4am):** The reaction was performed following the general procedure with (*E*)-*N*-(9*H*-fluoren-9-yl)-1-phenylmethanimine **1a** (269.1 mg, 1.0 mmol), 1-(*tert*-butyl) 4-(1,3-dioxoisoindolin-2-yl) piperidine-1,4-dicarboxylate **2m** (561.2 mg, 1.5 mmol) and ethene-1,1-diyldibenzene **3a** (540.8 mg, 3.0 mmol). The crude product was separated by flash chromatography on deactivated silica gel (hexanes:ethyl acetate = 20:1). Further purification by recrystallization (hexanes:MeOH = 10:1) give the product **4am** (550.1 mg, 87% yield) as a yellow solid. **Mp**: 165 – 167 °C. **R*f = 0.36 (hexanes:ethyl acetate = 10:1). **^{1}H NMR** (400 MHz, Chloroform-*d*) 7.87 (d, *J* = 7.6 Hz, 1H), 7.63 (d, *J* = 7.6 Hz, 1H), 7.48 (d, *J* = 7.2 Hz, 1H), 7.39 (d, *J* = 7.2 Hz, 1H), 7.35 – 7.32 (m, 2H), 7.28 – 7.10 (m, 9H), 7.04 – 6.94 (m, 4H), 6.89 (t, *J* = 7.2 Hz, 2H), 6.65 (d, *J* = 7.2 Hz, 2H), 6.34 (s, 1H), 3.79 – 3.57 (m, 2H), 2.40 – 2.27 (m, 2H), 2.20 – 2.15 (m, 1H), 1.89 – 1.82 (m, 1H), 1.43 – 1.37 (m, 1H), 1.31 (s, 9H), 0.94 – 0.73 (m, 4H) ppm; **^{13}C{^{1}H} NMR** (100 MHz, Chloroform-*d*) δ 160.7, 153.8, 144.6, 143.1, 141.6, 139.7, 139.6, 138.2, 130.6, 130.5, 130.1, 129.6, 128.8, 128.7, 127.2, 127.0, 126.4, 126.3, 125.8, 125.3, 125.2, 124.7, 121.8, 119.3, 118.1, 78.0, 69.5, 56.8, 44.2, 43.0, 32.9, 32.7, 31.5, 27.4 ppm, two resonances were not observed due to overlapping peaks; **IR** (thin film): 3058, 2927, 1683, 1449, 1287, 1168, 912, 743, 652 cm^{-1}; **HRMS** calc'd for $C_{44}H_{45}N_2O_2^{+}$ 633.3476, found 633.3477 $[M+H]^{+}$.

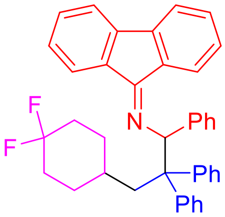

N*-(3-(4,4-Difluorocyclohexyl)-1,2,2-triphenylpropyl)-9*H*-fluoren-9-imine (4an):** The reaction was performed following the general procedure with (*E*)-*N*-(9*H*-fluoren-9-yl)-1-phenylmethanimine **1a** (269.1 mg, 1.0 mmol), 1,3-dioxoisoindolin-2-yl 4,4-difluorocyclohexane-1-carboxylate **2n** (463.6 mg, 1.5 mmol) and ethene-1,1-diyldibenzene **3a** (540.8 mg, 3.0 mmol). The crude product was separated by flash chromatography on deactivated silica gel (hexanes:ethyl acetate = 100:1). Further purification by recrystallization (hexanes:MeOH = 10:1) give the product **4ab** (465.2 mg, 82% yield) as a yellow solid. **Mp**: 175 – 177 °C. **R*f = 0.56 (hexanes:ethyl acetate = 20:1). **^{1}H NMR** (400 MHz, Chloroform-*d*) 7.87 (d, *J* = 7.6 Hz, 1H), 7.61 (d, *J* = 7.2 Hz, 1H), 7.46 (d, *J* = 7.2 Hz, 1H), 7.38 – 7.32 (m, 3H), 7.26 – 7.10 (m, 9H), 7.03 – 6.93 (m, 4H), 6.89 (t, *J* = 7.6 Hz, 2H), 6.64 (d, *J* = 7.6 Hz, 2H), 6.32 (s, 1H), 2.18 (dd, *J* = 14.4, 4.0 Hz, 1H), 1.83 (dd, *J* = 14.4, 4.0 Hz, 1H), 1.79 – 1.62 (m, 2H), 1.44 – 1.00 (m, 5H), 0.91 – 0.81 (m, 1H), 0.68 – 0.63 (m, 1H) ppm; **^{13}C{^{1}H} NMR** (100 MHz, Chloroform-*d*) δ 160.7, 144.4, 143.0, 141.3, 139.7, 139.6, 138.1, 130.6, 130.5, 130.1, 129.6, 128.7, 128.6, 127.2, 127.0, 126.4, 126.3, 125.8, 125.31, 125.29, 124.7, 122.3 (t, $J_{C\text{-}F}$ = 239.8 Hz), 121.7, 119.3, 118.1, 69.6, 56.9, 43.3, 32.9 (d, $J_{C\text{-}F}$ = 11.6 Hz), 32.6 (dd, $J_{C\text{-}F}$ = 11.6, 2.5 Hz) 32.4 (d, $J_{C\text{-}F}$ = 11.5 Hz), 31.3, 29.7 (d, $J_{C\text{-}F}$ = 9.0 Hz), 29.2 (d, $J_{C\text{-}F}$ = 8.9 Hz) ppm; **^{19}F NMR** (376 MHz, Chloroform-*d*) δ= -91.70 (d, *J* = 235.4 Hz, 1F), -101.47 (d, *J* = 233.12 Hz, 1F) ppm; **IR** (thin film): 2935, 1646, 1449, 1275, 1112, 913, 748, 702 cm^{-1}; **HRMS** calc'd for $C_{40}H_{36}F_2N^{+}$ 568.2810, found 568.2812 $[M+H]^{+}$.

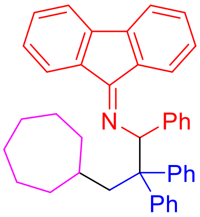

***N*-(3-Cycloheptyl-1,2,2-triphenylpropyl)-9*H*-fluoren-9-imine (4ao):** The reaction was performed following the general procedure with (*E*)-*N*-(9*H*-fluoren-9-yl)-1-phenylmethanimine **1a** (269.1 mg, 1.0 mmol), 1,3-dioxoisoindolin-2-yl cycloheptanecarboxylate **2o** (430.7 mg, 1.5 mmol) and ethene-1,1-diyldibenzene **3a** (540.8 mg, 3.0 mmol). The crude product was separated by flash

chromatography on deactivated silica gel (hexanes:ethyl acetate = 200:1). Further purification by recrystallization (hexanes:MeOH = 10:1) give the product **4ao** (490.8 mg, 90% yield) as a yellow solid. **Mp**: 168 – 170 °C. $\mathbf{R}_{\boldsymbol{f}}$ = 0.41 (hexanes:ethyl acetate = 80:1). **^{1}H NMR** (400 MHz, Chloroform-*d*) 7.92 (d, *J* = 7.6 Hz, 1H), 7.63 (d, *J* = 7.6 Hz, 1H), 7.47 (d, *J* = 7.6 Hz, 1H), 7.39 – 7.34 (m, 3H), 7.25 (t, *J* = 7.2 Hz, 1H), 7.21 – 7.14 (m, 5H), 7.12 – 7.10 (m, 3H), 7.02 – 6.93 (m, 4H), 6.88 (t, *J* = 7.6 Hz, 2H), 6.66 (d, *J* = 7.6 Hz, 2H), 6.35 (s, 1H), 2.15 (dd, *J* = 14.0, 4.0 Hz, 1H), 1.83 (dd, *J* = 14.0, 4.0 Hz, 1H), 1.50 – 1.44 (m, 1H), 1.32 – 1.16 (m, 7H), 1.09 – 0.88 (m, 3H), 0.77 – 0.72 (m, 2H) ppm; **^{13}C{^{1}H} NMR** (100 MHz, Chloroform-*d*) δ 160.4, 144.9, 143.0, 141.8, 139.9, 139.6, 138.2, 130.9, 130.6, 130.0, 129.4, 128.9, 128.8, 127.1, 126.9, 126.3, 126.2, 125.6, 125.1, 125.0, 124.3, 121.8, 119.2, 118.0, 69.4, 57.3, 46.1, 36.0, 34.8, 33.9, 27.3, 26.9, 25.7, 25.6 ppm, one resonance was not observed due to overlapping peaks; **IR** (thin film): 3057, 2923, 1646, 1449, 1275, 1102, 912, 749, 652 cm^{-1}; **HRMS** calc'd for $C_{41}H_{40}N^{+}$ 546.3155, found 546.3154 $[M+H]^{+}$.

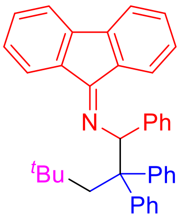

***N*-(4,4-Dimethyl-1,2,2-triphenylpentyl)-9*H*-fluoren-9-imine (4ap):** The reaction was performed following the general procedure with (*E*)-*N*-(9*H*-fluoren-9-yl)-1-phenylmethanimine **1a** (269.1 mg, 1.0 mmol), 1,3-dioxoisoindolin-2-yl pivalate **2p** (370.6 mg, 1.5 mmol) and ethene-1,1-diyldibenzene **3a** (540.8 mg, 3.0 mmol). The crude product was separated by flash chromatography on deactivated silica gel (hexanes:ethyl acetate = 200:1). Further purification by recrystallization (hexanes:MeOH = 10:1) give the product **4ap** (458.8 mg, 91% yield) as a yellow solid. **Mp**: 134 – 136 °C. $\mathbf{R}_{\boldsymbol{f}}$ = 0.41 (hexanes:ethyl acetate = 80:1). **^{1}H NMR** (400 MHz, Chloroform-*d*) 7.88 (d, *J* = 7.6 Hz, 1H), 7.62 (d, *J* = 7.2 Hz, 1H), 7.48 – 7.37 (m, 4H), 7.26 – 7.20 (m, 4H), 7.16 – 7.11 (m, 5H), 7.02 – 6.92 (m, 4H), 6.87 (t, *J* = 7.6 Hz, 2H), 6.70 – 6.57 (m, 2H), 6.50 (s, 1H), 2.29 (d, *J* = 14.8 Hz, 1H), 2.22 (d, *J* = 14.8 Hz, 1H), 0.56 (s, 9H) ppm; **^{13}C{^{1}H} NMR** (100 MHz, Chloroform-*d*) δ 160.4, 143.6, 143.0, 142.0, 140.2, 139.6, 138.2, 131.1, 130.5, 129.9, 129.4, 128.8, 127.2, 126.9, 126.2, 125.8, 125.6, 125.5, 125.0, 124.6, 124.5, 121.8, 119.2, 118.0, 69.5, 56.3, 49.1, 31.1, 30.8 ppm, one resonance was not observed due to overlapping peaks; **IR** (thin film): 2988, 1646, 1495, 1275, 1078, 913, 764, 749 cm^{-1}; **HRMS** calc'd for $C_{38}H_{36}N^{+}$ 506.2842, found 506.2843$[M+H]^{+}$.

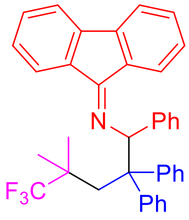

***N*-(5,5,5-Trifluoro-4,4-dimethyl-1,2,2-triphenylpentyl)-9*H*-fluoren-9-imine (4aq):** The reaction was performed following the general procedure with (*E*)-*N*-(9*H*-fluoren-9-yl)-1-phenylmethanimine **1a** (269.1 mg, 1.0 mmol), 1,3-dioxoisoindolin-2-yl 3,3,3-trifluoro-2,2-dimethylpropanoate **2q** (451.6 mg, 1.5 mmol) and ethene-1,1-diyldibenzene **3a** (540.8 mg, 3.0 mmol). The crude product was separated by flash chromatography on deactivated silica gel (hexanes:ethyl acetate = 200:1). Further purification by recrystallization (hexanes:MeOH = 10:1) give the product **4aq** (486.5 mg, 87% yield) as a yellow solid. **Mp**: 137 – 139 °C. $\mathbf{R}_{\boldsymbol{f}}$ = 0.40 (hexanes:ethyl acetate = 80:1). **^{1}H NMR** (400 MHz, Chloroform-*d*) 7.80 (d, *J* = 7.6 Hz, 1H), 7.60 (d, *J* = 7.6 Hz, 1H), 7.48 – 7.46 (m, 2H), 7.39 (d, *J* = 7.2 Hz, 1H), 7.32 – 7.27 (m, 2H), 7.26 – 7.13 (m, 8H), 7.08 – 7.02 (m, 3H), 6.96 (t, *J* = 7.2 Hz, 1H), 6.87 (t, *J* = 7.6 Hz, 2H), 6.63 – 6.51 (m, 2H), 6.37 (s, 1H), 2.74 (d, *J* = 14.8 Hz, 1H), 2.41 (d, *J* = 14.8 Hz, 1H), 0.56 (s, 3H), 0.49 (s, 3H) ppm; **^{13}C{^{1}H} NMR** (100 MHz, Chloroform-*d*) δ 160.8, 143.0, 142.2, 139.8, 139.7, 139.4, 138.1, 130.9, 130.4, 130.1, 129.6, 128.9 (q, $J_{C\text{-}F}$ = 282.2 Hz), 128.5, 127.2, 127.0, 126.22, 126.17, 125.9, 125.8, 125.5, 125.1, 125.0, 121.7, 119.2, 118.1, 71.2, 55.6, 40.8 (q, $J_{C\text{-}F}$ = 22.3 Hz), 39.9, 20.2, 19.4 ppm; **^{19}F NMR** (376 MHz, Chloroform-*d*) δ -80.80 (s, 3F) ppm; **IR** (thin film):

2989, 2875, 1622, 1476, 1275, 1122, 913, 764, 688 cm^{-1}; **HRMS** calc'd for $C_{38}H_{33}F_3N^+$ 560.2560, found 560.2563 $[M+H]^+$.

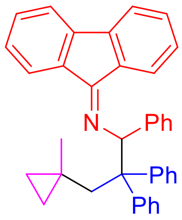

N*-(3-(1-Methylcyclopropyl)-1,2,2-triphenylpropyl)-9*H*-fluoren-9-imine (4ar):** The reaction was performed following the general procedure with (*E*)-*N*-(9*H*-fluoren-9-yl)-1-phenylmethanimine **1a** (269.1 mg, 1.0 mmol), 1,3-dioxoisoindolin-2-yl 1-methylcyclopropane-1-carboxylate **2r** (367.6 mg, 1.5 mmol) and ethene-1,1-diyldibenzene **3a** (540.8 mg, 3.0 mmol). The crude product was separated by flash chromatography on deactivated silica gel (hexanes:ethyl acetate = 200:1). Further purification by recrystallization (hexanes:MeOH = 10:1) give the product **4ar** (392.5 mg, 78% yield) as a yellow solid. **Mp**: 169 – 171 °C. **R*f = 0.36 (hexanes:ethyl acetate = 80:1). **^{1}H NMR** (400 MHz, Chloroform-*d*) 7.84 (d, *J* = 7.6 Hz, 1H), 7.61 (d, *J* = 7.2 Hz, 1H), 7.42 (d, *J* = 7.6 Hz, 1H), 7.36 – 7.33 (m, 3H), 7.21 – 7.08 (m, 9H), 7.03 – 6.96 (m, 3H), 6.94 – 6.92 (m, 1H), 6.87 (t, *J* = 7.6 Hz, 2H), 6.63 (d, *J* = 7.6 Hz, 2H), 6.38 (s, 1H), 2.47 (d, *J* = 14.8 Hz, 1H), 1.85 (d, *J* = 14.8 Hz, 1H), 0.66 (s, 3H), -0.11 – -0.15 (m, 1H), -0.23 – -0.33 (m, 2H), -0.41 – -0.45 (m, 1H) ppm; **^{13}C{^{1}H} NMR** (100 MHz, Chloroform-*d*) δ 161.6, 145.1, 144.1, 142.3, 141.1, 140.8, 139.3, 132.3, 131.6, 131.1, 130.7, 130.6, 129.9, 128.3, 128.1, 127.3, 127.0, 126.82, 126.79, 126.2, 125.8, 125.6, 123.0, 120.4, 119.2, 70.5, 58.4, 47.5, 26.2, 14.8, 14.2, 13.7 ppm; **IR** (thin film): 3005, 2918, 1645, 1449, 1275, 912, 748, 652 cm^{-1}; **HRMS** calc'd for $C_{38}H_{34}N^+$ 504.2686, found 504.2688 $[M+H]^+$.

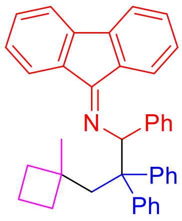

N*-(3-(1-Methylcyclobutyl)-1,2,2-triphenylpropyl)-9*H*-fluoren-9-imine (4as):** The reaction was performed following the general procedure with (*E*)-*N*-(9*H*-fluoren-9-yl)-1-phenylmethanimine **1a** (269.1 mg, 1.0 mmol), 1,3-dioxoisoindolin-2-yl 1-methylcyclobutane-1-carboxylate **2s** (388.6 mg, 1.5 mmol) and ethene-1,1-diyldibenzene **3a** (540.8 mg, 3.0 mmol). The crude product was separated by flash chromatography on deactivated silica gel (hexanes:ethyl acetate = 200:1). Further purification by recrystallization (hexanes:MeOH = 10:1) give the product **4as** (377.6 mg, 73% yield) as a yellow solid. **Mp**: 150 – 152 °C. **R*f = 0.37 (hexanes:ethyl acetate = 80:1). **^{1}H NMR** (400 MHz, Chloroform-*d*) 7.89 (d, *J* = 7.6 Hz, 1H), 7.60 (d, *J* = 7.2 Hz, 1H), 7.46 (d, *J* = 7.6 Hz, 1H), 7.39 – 7.36 (m, 3H), 7.24 – 7.10 (m, 9H), 7.02 – 6.92 (m, 4H), 6.87 (t, *J* = 7.6 Hz, 2H), 6.63 (d, *J* = 7.6 Hz, 2H), 6.45 (s, 1H), 2.44 (d, *J* = 4.8 Hz, 1H), 2.25 (d, *J* = 4.8 Hz, 1H), 1.62 – 1.53 (m, 1H), 1.44 – 1.32 (m, 2H), 1.24 – 1.17 (m, 1H), 1.02 (s, 3H), 1.00 – 0.90 (m, 2H) ppm; **^{13}C{^{1}H} NMR** (100 MHz, Chloroform-*d*) δ 161.5, 144.2, 144.1, 142.2, 141.1, 140.8, 139.3, 132.3, 131.7, 131.0, 130.8, 130.5, 129.9, 128.2, 128.0, 127.3, 127.0, 126.7, 126.1, 125.7, 125.5, 122.9, 120.3, 119.1, 71.1, 57.5, 50.9, 39.0, 35.4, 34.9, 27.2, 16.1 ppm, one resonance was not observed due to overlapping peaks; **IR** (thin film): 3058, 2929, 1685, 1449, 1275, 1167, 912, 749, 653 cm^{-1}; **HRMS** calc'd for $C_{39}H_{36}N^+$ 518.2842, found 518.2842 $[M+H]^+$.

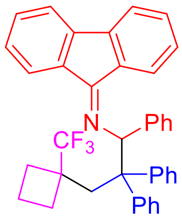

***N*-(1,2,2-Triphenyl-3-(1-(trifluoromethyl)cyclobutyl)propyl)-9*H*-fluoren-9-imine (4at):** The reaction was performed following the general procedure with (*E*)-*N*-(9*H*-fluoren-9-yl)-1-phenylmethanimine **1a** (269.1 mg, 1.0 mmol), 1,3-dioxoisoindolin-2-yl 1-(trifluoromethyl)cyclobutane-1-carboxylate **2t** (469.6 mg, 1.5 mmol) and ethene-1,1-diyldibenzene **3a** (540.8 mg, 3.0 mmol). The crude product was separated by flash chromatography on deactivated silica gel (hexanes:ethyl acetate = 200:1). Further purification by recrystallization (hexanes:MeOH = 10:1) give the product **4at**

(371.3 mg, 65% yield) as a yellow solid. **Mp**: 163 – 165 °C. R_f = 0.34 (hexanes:ethyl acetate = 80:1). **^{1}H NMR** (400 MHz, Chloroform-*d*) 7.80 (d, *J* = 7.6 Hz, 1H), 7.60 (d, *J* = 7.2 Hz, 1H), 7.51 – 7.36 (m, 6H), 7.24 – 7.18 (m, 5H), 7.14 – 7.07 (m, 4H), 7.04 – 7.00 (m, 1H), 6.95 – 6.91 (m, 1H), 6.86 (t, *J* = 7.2 Hz, 2H), 6.61 (d, *J* = 7.6 Hz, 2H), 6.30 (s, 1H), 2.74 (d, *J* = 15.2 Hz, 1H), 2.18 (d, *J* = 15.2 Hz, 1H), 1.84 – 1.78 (m, 1H), 1.63 – 1.51 (m, 3H), 0.92 – 0.80 (m, 2H) ppm; **^{13}C{^{1}H} NMR** (100 MHz, Chloroform-*d*) δ 160.8, 143.0, 142.2, 139.7, 139.2, 138.9, 138.0, 130.6, 130.4, 130.0, 129.7 (q, $J_{C\text{-}F}$ = 279.4 Hz), 129.5, 129.2, 128.4, 127.2, 126.9, 126.5, 126.3, 125.9, 125.8, 125.5, 125.4, 125.0, 121.6, 119.2, 118.1, 72.6, 55.8, 44.2 (q, *J* = 23.9 Hz), 38.7, 24.7, 24.3, 15.3 ppm; **^{19}F NMR** (376 MHz, Chloroform-*d*) δ -80.96 (s, 3F) ppm; **IR** (thin film): 3056, 2927, 1678, 1447, 1275, 1167, 912, 738, 654 cm^{-1}; **HRMS** calc'd for $C_{39}H_{33}F_3N^+$ 572.2560, found 572.2559 [M+H]$^+$.

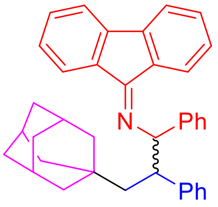

***N*-(3-((3r,5r,7r)-Adamantan-1-yl)-1,2-diphenylpropyl)-9*H*-fluoren-9-imine (4au):** The reaction was performed following the general procedure with (*E*)-*N*-(9*H*-fluoren-9-yl)-1-phenylmethanimine **1a** (269.1 mg, 1.0 mmol), 1,3-dioxoisoindolin-2-yl (3r,5r,7r)-adamantane-1-carboxylate **2a** (487.7 mg, 1.5 mmol) and styrene **3u** (312.2 mg, 3.0 mmol). The crude product was separated by flash chromatography on deactivated silica gel (hexanes:ethyl acetate = 200:1). Further purification was performed on an Agilent HPLC 1260 system using acetonitrile:H_2O (98:2 vol./vol.) as mobile phase and flow rate of 5 mL/min with monitoring at 254 nm to give the product **4au** in 71% overall yield (**4au'**, 180.1 mg, 36% yield, **4au''**, 180.1 mg, 36% yield, dr = 1:1).

4au': yellow oil. R_f = 0.54 (hexanes:ethyl acetate = 20:1). **^{1}H NMR** (400 MHz, Chloroform-*d*) 7.93 (d, *J* = 7.2 Hz, 1H), 7.75 (d, *J* = 8.0 Hz, 1H), 7.58 (d, *J* = 7.2 Hz, 1H), 7.53 (d, *J* = 7.2 Hz, 1H), 7.41 – 7.31 (m, 3H), 7.21 – 7.06 (m, 11H), 5.64 (d, *J* = 4.8 Hz, 1H), 3.42 – 3.39 (m, 1H), 1.82 – 1.78 (m, 4H), 1.68 – 1.49 (m, 7H), 1.40 – 1.35 (m, 3H), 1.29 – 1.25 (m, 3H) ppm; **^{13}C{^{1}H} NMR** (100 MHz, Chloroform-*d*) δ 161.5, 143.9, 143.6, 142.8, 140.8, 139.2, 131.6, 130.8, 130.6, 130.1, 128.2, 127.9, 127.8, 127.7, 127.4, 127.1, 126.6, 125.7, 122.7, 120.1, 119.1, 71.0, 49.4, 46.5, 43.1, 37.1, 33.4, 28.7 ppm; **IR** (thin film): 2916, 2827, 1652, 1456, 1265, 1108, 916, 745, 651 cm^{-1}; **HRMS** calc'd for $C_{38}H_{38}N^+$ 508.2999, found 508.2996 [M+H]$^+$.

4au'': yellow oil. R_f = 0.54 (hexanes:ethyl acetate = 20:1). **^{1}H NMR** (400 MHz, Chloroform-*d*) 8.00 (d, *J* = 6.4 Hz, 1H), 7.64 (d, *J* = 7.6 Hz, 1H), 7.59 – 7.54 (m, 2H), 7.43 – 7.34 (m, 2H), 7.30 (td, *J* = 8, 0.8 Hz, 1H), 7.20 – 7.17 (m, 2H), 7.15 – 7.03 (m, 9H), 5.47 (d, *J* = 6.4 Hz, 1H), 3.49 – 3.44 (m, 1H), 1.85 – 1.72 (m, 5H), 1.60 – 1.56 (m, 3H), 1.51 – 1.48 (m, 3H), 1.37 – 1.33 (m, 3H), 1.25 – 1.21 (m, 3H) ppm; **^{13}C{^{1}H} NMR** (100 MHz, Chloroform-*d*) δ 162.0, 145.3, 143.8, 143.3, 140.9, 139.2, 131.8, 130.8, 130.6, 129.3, 128.3, 127.8, 127.7, 127.5, 126.4, 125.7, 122.8, 120.1, 119.2, 71.2, 50.5, 45.6, 43.1, 37.1, 33.2, 28.8 ppm, two resonances were not observed due to overlapping peaks; **IR** (thin film): 2906, 2852, 1644, 1438, 1268, 1102, 917, 748, 654 cm^{-1}; **HRMS** calc'd for $C_{38}H_{38}N^+$ 508.2999, found 508.2999 [M+H]$^+$.

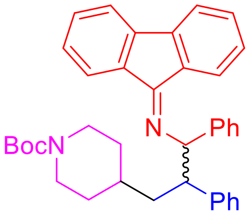

***tert*-Butyl 4-(3-((9*H*-fluoren-9-ylidene)amino)-2,3-diphenylpropyl)piperidine-1-carboxylate (4mu):** The reaction was performed following the general procedure with (*E*)-*N*-(9*H*-fluoren-9-yl)-1-phenylmethanimine **1a** (269.1 mg, 1.0 mmol), 1-(*tert*-butyl) 4-(1,3-dioxoisoindolin-2-yl)piperidine-1,4-dicarboxylate **2m** (561.2 mg, 1.5 mmol) and styrene **3u** (312.2 mg, 3.0 mmol). The crude product was separated by flash chromatography on deactivated silica gel (hexanes:ethyl acetate = 20:1). Further purification was performed on an Agilent HPLC 1260 system

using acetonitrile:H_2O (98:2 vol./vol.) as mobile phase and flow rate of 5 mL/min with monitoring at 254 nm to give the product **4mu** in 63% overall yield (**4mu'**, 178.0 mg, 32% yield, **4mu''**, 178.0 mg, 32% yield, dr = 1:1).

4mu': yellow oil. **R*f*** = 0.31 (hexanes:ethyl acetate = 10:1). **^{1}H NMR** (400 MHz, Chloroform-*d*) 7.82 (d, *J* = 7.2 Hz, 1H), 7.68 (d, *J* = 8.0 Hz, 1H), 7.49 (d, *J* = 7.2 Hz, 1H), 7.44 (d, *J* = 7.2 Hz, 1H), 7.31 (td, *J* = 7.6, 1.2 Hz, 1H), 7.25 (t, *J* = 7.2 Hz, 2H), 7.13 – 7.03 (m, 11H), 5.60 (d, *J* = 5.2 Hz, 1H), 3.98 – 3.74 (m, 2H), 3.30 – 3.27 (m, 1H), 2.47 – 2.35 (m, 2H), 1.83 – 1.76 (m, 1H), 1.58 – 1.54 (m, 3H), 1.34 (s, 9H), 1.18 – 1.13 (m, 1H), 1.00 – 0.89 (m, 2H) ppm; **^{13}C{^{1}H} NMR** (100 MHz, Chloroform-*d*) δ 160.6, 153.8, 142.9, 141.5, 140.2, 139.8, 138.0, 130.5, 129.9, 129.6, 128.6, 127.5, 127.2, 126.9, 126.7, 126.5. 126.4, 125.7, 125.1, 121.6, 119.2, 118.1, 78.0, 68.8, 50.5, 42.7, 37.9, 32.2, 27.4 ppm, one resonance was not observed due to overlapping peaks; **IR** (thin film): 2988, 1686, 1449, 1275, 1167, 913, 749, 701 cm^{-1}; **HRMS** calc'd for $C_{38}H_{41}N_2O_2^+$ 557.3163, found 557.3166 [M+H]$^+$.

4mu'': yellow oil. **R*f*** = 0.31 (hexanes:ethyl acetate = 10:1). **^{1}H NMR** (400 MHz, Chloroform-*d*) 7.91 (d, *J* = 7.2 Hz, 1H), 7.61 (d, *J* = 7.6 Hz, 1H), 7.51 (d, *J* = 7.6 Hz, 1H), 7.48 (d, *J* = 7.2 Hz, 1H), 7.36 – 7.23 (m, 3H), 7.12 – 7.01 (m, 11H), 5.47 (d, *J* = 6.8 Hz, 1H), 3.95 – 3.73 (m, 2H), 3.36 – 3.30 (m, 1H), 2.47 – 2.35 (m, 2H), 1.88 – 1.81 (m, 1H), 1.70 – 1.56 (m, 3H), 1.34 (s, 9H), 1.13 – 1.08 (m, 1H), 1.00 – 0.90 (m, 2H) ppm; **^{13}C{^{1}H} NMR** (100 MHz, Chloroform-*d*) δ 161.1, 153.8, 142.9, 141.9, 141.5, 139.9, 138.0, 130.7, 129.9, 129.8, 127.9, 127.3, 127.0, 126.8, 126.6, 126.4. 125.5, 125.2, 121.7, 119.2, 118.2, 78.0, 69.5, 51.6, 42.8, 37.0, 32.4, 27.4 ppm, two resonances were not observed due to overlapping peaks; **IR** (thin film): 2978, 1656, 1448, 1270, 1157, 910, 750, 680 cm^{-1}; **HRMS** calc'd for $C_{38}H_{41}N_2O_2^+$ 557.3163, found 557.3158 [M+H]$^+$.

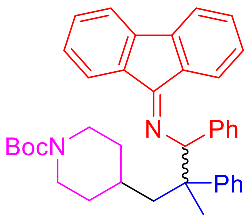

***tert*-Butyl 4-(3-((9*H*-fluoren-9-ylidene)amino)-2-methyl-2,3-diphenylpropyl)piperidine-1-carboxylate (4mv):** The reaction was performed following the general procedure with (*E*)-*N*-(9*H*-fluoren-9-yl)-1-phenylmethanimine **1a** (269.1 mg, 1.0 mmol), 1-(*tert*-butyl) 4-(1,3-dioxoisoindolin-2-yl) piperidine-1,4-dicarboxylate **2m** (561.2 mg, 1.5 mmol) and prop-1-en-2-ylbenzene **3v** (354.2 mg, 3.0 mmol). The crude product was separated by flash chromatography on deactivated silica gel (hexanes:ethyl acetate = 200:1). Further purification by recrystallization (hexanes:MeOH = 10:1) give the product **4mv** in 50% overall yield (**4mv'**(major), 165.4 mg, 29% yield, **4mv''** (minor), 119.8 mg, 21% yield, dr = 1.4:1).

4mv' (major): yellow solid. **Mp**: 189 – 191 °C. **R*f*** = 0.44 (hexanes:ethyl acetate = 10:1). **^{1}H NMR** (400 MHz, Chloroform-*d*) 7.90 (d, *J* = 6.8 Hz, 1H), 7.55 – 7.46 (m, 3H), 7.35 – 7.18 (m, 7H), 7.14 – 7.11 (m, 1H), 7.39 (td, *J* = 7.6, 1.2 Hz, 1H), 6.95 – 6.88 (m, 3H), 6.71 – 6.68 (m, 2H), 5.44 (s, 1H), 3.92 – 3.58 (m, 2H), 2.46 (t, *J* = 12.8 Hz, 1H), 3.31 (t, *J* = 12.8 Hz, 1H), 1.85 – 1.75 (m, 2H), 1.56 – 1.53 (m, 4H), 1.31 (s, 9H), 1.27 – 1.21 (m, 1H), 1.11 – 0.99 (m, 2H), 0.83 – 0.73 (m, 1H) ppm; **^{13}C{^{1}H} NMR** (100 MHz, Chloroform-*d*) δ 160.8, 153.7, 144.5, 142.8, 139.9, 139.8, 138.0, 130.4, 129.9, 129.7, 127.8, 127.2, 127.0, 126.9, 126.6, 126.5, 125.9, 125.4, 125.0, 121.5, 119.2, 118.2, 78.0, 73.7, 47.0, 44.5, 43.0, 33.6, 32.8, 31.8, 27.4, 17.1 ppm; **IR** (thin film): 2931, 2852, 1673, 1440, 1282, 1105, 916, 759, 632 cm^{-1}; **HRMS** calc'd for $C_{39}H_{43}N_2O_2^+$ 571.3319, found 571.3324 [M+H]$^+$.

4mv'' (minor): yellow solid. **Mp**: 188 – 190 °C. **R*f*** = 0.45 (hexanes:ethyl acetate = 10:1). **^{1}H NMR** (400 MHz, Chloroform-*d*) 7.81 (d, *J* = 6.8 Hz, 1H), 7.67 (d, *J* = 8 Hz, 1H), 7.47 (d, *J* = 7.2 Hz, 1H), 7.42 (d, *J* = 7.2 Hz, 1H), 7.31 – 7.20 (m, 5H), 7.15 – 7.01 (m, 7H), 6.91 – 6.89 (m, 2H), 5.34 (s, 1H), 3.92 – 3.66 (m, 2H), 2.50 – 2.44 (m, 1H), 2.39 – 2.32 (m, 1H), 2.08 (dd, *J* = 14, 5.6 Hz, 1H), 1.68 (dd, *J* = 14, 5.6

Hz, 1H), 1.57 – 1.51 (m, 1H), 1.46 (s, 3H), 1.32 (s, 9H), 1.31 – 1.26 (m, 1H), 1.16 – 1.10 (m, 2H), 0.91 – 0.85 (m, 1H) ppm; **^{13}C{^{1}H} NMR** (100 MHz, Chloroform-*d*) δ 160.0, 153.8, 143.5, 142.9, 139.8, 139.7, 138.1, 130.5, 129.8, 129.6, 128.4, 127.4, 127.2, 126.7, 126.3, 126.1, 126.0, 125.8, 124.8, 121.5, 119.1, 118.1, 78.0, 74.1, 46.5, 42.9, 33.7, 32.9, 31.7, 27.4, 21.1 ppm, one resonance was not observed due to overlapping peaks;; **IR** (thin film): 2955, 2838, 1640, 1463, 1270, 1104, 908, 748, 653 cm^{-1}; **HRMS** calc'd for $C_{39}H_{43}N_2O_2^+$ 571.3319, found 571.3323 [M+H]$^+$.

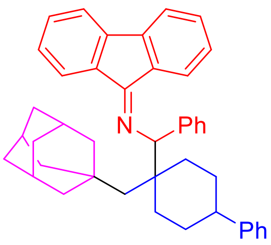

N*-((1-(((3r,5r,7r)-Adamantan-1-yl)methyl)-4-phenylcyclohexyl)(phenyl)methyl)-9*H*-fluoren-9-imine (4aw):** The reaction was performed following the general procedure with (*E*)-*N*-(9*H*-fluoren-9-yl)-1-phenylmethanimine **1a** (269.1 mg, 1.0 mmol), 1,3-dioxoisoindolin-2-yl (3r,5r,7r)-adamantane-1-carboxylate **2a** (487.7 mg, 1.5 mmol) and (4-methylenecyclohexyl)benzene **3w** (516.4 mg, 3.0 mmol). The crude product was separated by flash chromatography on deactivated silica gel (hexanes:ethyl acetate = 200:1). Further purification was performed on an Agilent HPLC 1260 system using acetonitrile:H_2O (98:2 vol./vol.) as mobile phase and flow rate of 5 mL/min with monitoring at 254 nm to give the product **4aw** (235.9 mg, 41% yield) as a yellow oil. **R*f = 0.50 (hexanes:ethyl acetate = 50:1). **^{1}H NMR** (600 MHz, Chloroform-*d*) 7.93 (d, *J* = 7.8 Hz, 1H), 7.64 (d, *J* = 7.8 Hz, 1H), 7.50 (dd, *J* = 10.2, 2.4 Hz, 2H), 7.35 – 7.32 (m, 3H), 7.28 (t, *J* = 7.8 Hz, 1H), 7.24 – 7.19 (m, 4H), 7.16 – 7.14 (m, 3H), 7.11 – 7.06 (m, 3H), 5.76 (s, 1H), 2.25 – 2.15 (m, 2H), 1.84 – 1.82 (m, 1H), 1.76 – 1.72 (m, 5H), 1.68 – 1.64 (m, 6H), 1.63 – 1.60 (m, 3H), 1.53 – 1.46 (m, 9H) ppm; **^{13}C{^{1}H} NMR** (150 MHz, Chloroform-*d*) δ 160.8, 145.9, 142.7, 140.8, 140.0, 138.3, 130.6, 129.7, 129.5, 129.4, 127.23, 127.16, 126.8, 126.5, 126.1, 125.8, 125.4, 124.7, 121.5, 119.0, 118.1, 68.7, 44.0, 43.4, 42.9, 42.0, 36.0, 33.8, 31.2, 30.8, 29.2, 29.0, 28.7, 28.1 ppm; **IR** (thin film): 2958, 2788, 1589, 1438, 1243, 989, 757, 655 cm^{-1}; **HRMS** calc'd for $C_{43}H_{46}N^+$ 576.3625, found 576.3620 [M+H]$^+$.

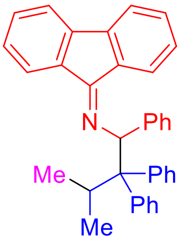

N*-(3-Methyl-1,2,2-triphenylbutyl)-9H-fluoren-9-imine (4ux):** The reaction was performed following the general procedure with (*E*)-*N*-(9*H*-fluoren-9-yl)-1-phenylmethanimine **1a** (269.1 mg, 1.0 mmol), 1,3-dioxoisoindolin-2-yl acetate **2u** (307.6 mg, 1.5 mmol) and prop-1-ene-1,1-diyldibenzene **3x** (582.3 mg, 3.0 mmol). The crude product was separated by flash chromatography on deactivated silica gel (hexanes:ethyl acetate = 100:1). Further purification by recrystallization (hexanes:MeCN = 10:1) give the product **4ux** (300.7 mg, 63% yield) as a yellow solid. **Mp**: 188 – 200 °C. **R*f = 0.32 (hexanes:ethyl acetate = 80:1). **^{1}H NMR** (400 MHz, Chloroform-*d*) 7.86 (d, *J* = 7.6 Hz, 1H), 7.67 – 7.38 (m, 4H), 7.27 – 7.13 (m, 7H), 7.01 – 6.88 (m, 9H), 6.68 (s, 1H), 6.64 – 6.59 (m, 2H), 2.86 – 2.57 (m, 1H), 0.96 – 0.68 (m, 6H) ppm; **^{13}C{^{1}H} NMR** (100 MHz, Chloroform-*d*) δ 160.6, 143.0, 140.3, 139.7, 138.2, 130.4, 130.0, 129.4, 128.4, 127.1, 127.0, 126.1, 125.64, 125.59, 125.0, 124.8, 124.4, 124.3, 121.8, 119.2, 118.0, 64.3, 60.8, 28.5, 18.0 ppm; **IR** (thin film): 2968, 1646, 1599, 1448, 1260, 1036, 913, 744, 705, 652 cm^{-1}; **HRMS** calc'd for $C_{36}H_{32}N^+$ 478.2529, found 478.2526 [M+H]$^+$.

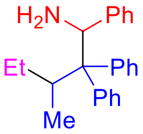

3-Methyl-1,2,2-triphenylpentan-1-amine (4vx): The reaction was performed following the general procedure with (*E*)-*N*-(9*H*-fluoren-9-yl)-1-phenylmethanimine **1a** (269.1 mg, 1.0 mmol), 1,3-dioxoisoindolin-2-yl propionate **2v** (328.6 mg, 1.5 mmol) and prop-1-ene-1,1-diyldibenzene **3x** (582.3 mg, 3.0 mmol). After the reaction was complete, 1 M aq. HCl (5 mL) was added to the reaction mixture at 0 °C. The resulting mixture was left at room temperature until the imine products were fully consumed, and then diluted with H_2O (20 mL). The mixture was concentrated to remove THF and extracted with Et_2O (50 mL) to remove fluorenone. The aqueous layer was basified with NaOH (1 M) to pH > 9 and extracted with CH_2Cl_2 (30 ml). The combined organics were dried over Na_2SO_4, filtered, and concentrated under vacuum. The crude product was separated by flash chromatography on deactivated silica gel (hexanes:ethyl acetate = 5:1) to give the product **4vx** (171.2 mg, 52% yield over 2 steps, dr = 2.3:1) as a yellow oil. **R*f*** = 0.30 (hexanes:ethyl acetate = 3:1). Diastereomeric ratio was determined based on H* (α-amino C–H's) (1H, ~ 5.2 – 5.1 ppm), see ^{1}H spectra (PageS67) for determination of diastereomeric ratio; **IR** (thin film): 3005, 1496, 1452, 1260, 1036, 913, 749, 706 cm^{-1}; **HRMS** calc'd for $C_{24}H_{28}N^+$ 330.2216, found 330.2213 [M+H]$^+$.

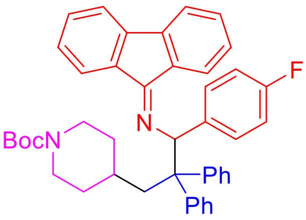

tert*-Butyl 4-(3-((9*H*-fluoren-9-ylidene)amino)-3-(4-fluorophenyl)-2,2-diphenylpropyl)piperidine-1-carboxylate (4bm):** The reaction was performed following the general procedure with (*E*)-*N*-(9*H*-fluoren-9-yl)-1-(4-fluorophenyl)methanimine **1b** (287.1 mg, 1.0 mmol), 1-(*tert*-butyl) 4-(1,3-dioxoisoindolin-2-yl) piperidine-1,4-dicarboxylate **2m** (561.2 mg, 1.5 mmol) and ethene-1,1-diyldibenzene **3a** (540.8 mg, 3.0 mmol). The crude product was separated by flash chromatography on deactivated silica gel (hexanes:ethyl acetate = 10:1). Further purification by recrystallization (hexanes:MeOH = 10:1) give the product **4bm** (604.8 mg, 93% yield) as a yellow solid. **Mp**: 176 – 178 °C. **R*f = 0.35 (hexanes:ethyl acetate = 10:1). **^{1}H NMR** (400 MHz, Chloroform-*d*) 7.82 (d, *J* = 7.6 Hz, 1H), 7.60 (d, *J* = 7.2 Hz, 1H), 7.47 (d, *J* = 6.8 Hz, 1H), 7.39 – 7.28 (m, 3H), 7.26 – 7.24 (m, 1H), 7.21 – 7.14 (m, 5H), 7.12 – 7.10 (m, 3H), 7.03 – 6.95 (m, 3H), 6.63 – 6.56 (m, 4H), 6.32 (s, 1H), 3.89 – 3.51 (m, 2H), 2.39 – 2.25 (m, 2H), 2.14 – 2.10 (m, 1H), 1.88 – 1.83 (m, 1H), 1.40 – 1.34 (m, 1H), 1.30 (s, 9H), 0.90 – 0.49 (m, 4H) ppm; **^{13}C{^{1}H} NMR** (100 MHz, Chloroform-*d*) δ 160.1, 159.2, 158.5 (d, $J_{C\text{-}F}$ = 140.7 Hz), 152.4, 143.4, 142.3, 140.6, 139.1, 137.5, 135.0 (d, $J_{C\text{-}F}$ = 3.1 Hz), 130.4, 130.2, 130.1, 129.8 (d, $J_{C\text{-}F}$ = 7.4 Hz), 129.5, 128.7, 127.2, 127.0, 126.4, 126.3, 125.49, 125.46, 124.9, 122.0, 119.7, 118.6, 113.4 (d, $J_{C\text{-}F}$ = 20.0 Hz), 80.4, 71.6, 60.2, 48.2, 47.0, 37.4, 37.2, 36.0, 32.2 ppm; **^{19}F NMR** (376 MHz, Chloroform-*d*) δ -115.17 (s, 1F) ppm; **IR** (thin film): 3058, 2925, 1682, 1448, 1276, 1167, 911, 731, 651 cm^{-1}; **HRMS** calc'd for $C_{44}H_{44}FN_2O_2^+$ 651.3381, found 651.3383 [M+H]$^+$.

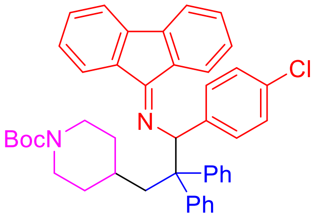

tert*-Butyl 4-(3-((9*H*-fluoren-9-ylidene)amino)-3-(4-chlorophenyl)-2,2-diphenylpropyl)piperidine-1-carboxylate (4cm):** The reaction was performed following the general procedure with (*E*)-1-(4-chlorophenyl)-*N*-(9*H*-fluoren-9-yl)methanimine **1c** (303.1 mg, 1.0 mmol), 1-(*tert*-butyl) 4-(1,3-dioxoisoindolin-2-yl) piperidine-1,4-dicarboxylate **2m** (561.2 mg, 1.5 mmol) and ethene-1,1-diyldibenzene **3a** (540.8 mg, 3.0 mmol). The crude product was separated by flash chromatography on deactivated silica gel (hexanes:ethyl acetate = 10:1). Further purification by recrystallization (hexanes:MeOH = 10:1) give the product **4cm** (633.0 mg, 95% yield) as a yellow solid. **Mp**: 165 – 167 °C. **R*f = 0.36 (hexanes:ethyl

acetate = 10:1). **^{1}H NMR** (400 MHz, Chloroform-*d*) 7.80 (d, *J* = 7.6 Hz, 1H), 7.61 (d, *J* = 7.6 Hz, 1H), 7.51 (d, *J* = 7.6 Hz, 1H), 7.41 (d, *J* = 7.2 Hz, 1H), 7.35 – 7.23 (m, 4H), 7.21 – 7.10 (m, 7H), 7.05 – 6.98 (m, 3H), 6.88 (d, *J* = 8.0 Hz, 2H), 6.57 (d, *J* = 8.0 Hz, 2H), 6.31 (s, 1H), 3.80 – 3.59 (m, 2H), 2.40 – 2.26 (m, 2H), 2.15 – 2.10 (m, 1H), 1.85 – 1.81 (m, 1H), 1.40 – 1.35 (m, 1H), 1.31 (s, 9H), 1.07 – 0.48 (m, 4H) ppm; **^{13}C{^{1}H} NMR** (100 MHz, Chloroform-*d*) δ 153.3, 146.4, 137.2, 136.3, 134.3, 133.0, 131.5, 131.4, 125.3, 124.3, 124.1, 124.0, 123.6, 123.5, 122.6, 121.1, 120.9, 120.4, 120.1, 120.0, 119.44, 119.41, 118.8, 115.9, 113.7, 112.5, 74.3, 65.6, 54.0, 42.1, 40.9, 31.3, 31.1, 29.9, 26.1 ppm, one resonance was not observed due to overlapping peaks; **IR** (thin film): 3058, 2925, 1682, 1448, 1276, 1167, 911, 731, 651 cm^{-1}; **HRMS** calc'd for $C_{44}H_{44}ClN_2O_2^+$ 667.3086, found 667.3083 [M+H]$^+$.

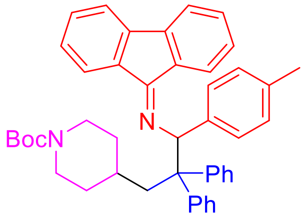

***tert*-Butyl 4-(3-((9*H*-fluoren-9-ylidene)amino)-2,2-diphenyl-3-(*p*-tolyl) propyl)piperidine-1-carbox-ylate (4dm):** The reaction was performed following the general procedure with (*E*)-*N*-(9*H*-fluoren-9-yl)-1-(*m*-tolyl)methanimine **1d** (283.1 mg, 1.0 mmol), 1-(*tert*-butyl) 4-(1,3-dioxoisoindolin-2-yl) piperidine-1,4- dicarboxylate **2m** (561.2 mg, 1.5 mmol) and ethene-1,1-diyldibenzene **3a** (540.8 mg, 3.0 mmol). The crude product was separated by flash chromatography on deactivated silica gel (hexanes:ethyl acetate = 8:1). Further purification was performed on an Agilent HPLC 1260 system using acetonitrile:H_2O (98:2 vol./vol.) as mobile phase and flow rate of 5 mL/min with monitoring at 254 nm to give the product **4dm** (517.1 mg, 80% yield) as a yellow oil. **R$_f$** = 0.32 (hexanes:ethyl acetate = 10:1). **^{1}H NMR** (400 MHz, Chloroform-*d*) 7.88 (d, *J* = 7.6 Hz, 1H), 7.61 (d, *J* = 7.2 Hz, 1H), 7.47 (d, *J* = 7.2 Hz, 1H), 7.39 – 7.35 (m, 3H), 7.25 (t, *J* = 7.6 Hz, 1H), 7.21 – 7.16 (m, 4H), 7.14 – 7.10 (m, 4H), 7.03 – 7.00 (m, 3H), 6.70 (d, *J* = 8.0 Hz, 2H), 6.52 (d, *J* = 7.6 Hz, 2H), 6.31 (s, 1H), 3.92 – 3.53 (m, 2H), 2.40 – 2.28 (m, 2H), 2.23 – 2.14 (m, 1H), 2.08 (s, 3H), 1.92 – 1.78 (m, 1H), 1.45 – 1.35 (m, 1H), 1.31 (s, 9H), 1.00 – 0.42 (m, 4H) ppm; **^{13}C{^{1}H} NMR** (100 MHz, Chloroform-*d*) δ 160.5, 153.8, 144.8, 143.0, 141.7, 139.7, 138.2, 136.5, 135.3, 130.7, 130.5, 130.0, 129.5, 128.8, 128.6, 127.1, 127.0, 126.5, 126.4, 126.3, 125.22, 125.16, 124.6, 121.8, 119.2, 118.1, 78.0, 69.6, 56.7, 44.2, 43.1, 32.9, 32.7, 31.4, 27.4, 20.0 ppm, one resonance was not observed due to overlapping peaks; **IR** (thin film): 2921, 1686, 1449, 1275, 1168, 913, 748, 653 cm^{-1}; **HRMS** calc'd for $C_{45}H_{47}N_2O_2^+$ 647.3632, found 647.3630 [M+H]$^+$.

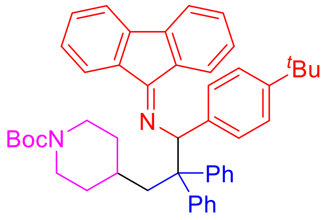

***tert*-Butyl 4-(3-((9*H*-fluoren-9-ylidene)amino)-3-(4-(*tert*-butyl) phenyl)-2,2-diphenylpropyl)piperi-dine-1-carboxylate (4em):** The reaction was performed following the general procedure with (*E*)-1-(3-(*tert*-butyl)phenyl)-*N*-(9*H*-fluoren-9-yl)methanimine **1e** (325.2 mg, 1.0 mmol), 1-(*tert*-butyl) 4-(1,3-dioxoisoindolin-2-yl) piperidine-1,4-dicarboxylate **2m** (561.2 mg, 1.5 mmol) and ethene-1,1-diyldibenzene **3a** (540.8 mg, 3.0 mmol). The crude product was separated by flash chromatography on deactivated silica gel (hexanes:ethyl acetate = 20:1). Further purification by recrystallization (hexanes:MeOH = 10:1) give the product **4em** (509.4 mg, 74% yield) as a yellow solid. **Mp**: 170 – 172 °C. **R$_f$** = 0.34 (hexanes:ethyl acetate = 10:1). **^{1}H NMR** (400 MHz, Chloroform-*d*) 7.89 (d, *J* = 7.6 Hz, 1H), 7.60 (d, *J* = 7.2 Hz, 1H), 7.34 – 7.27 (m, 4H), 7.18 – 7.06 (m, 9H), 6.99 – 6.93 (m, 3H), 6.89 (d, *J* = 8.0 Hz, 2H), 6.56 (d, *J* = 8.0 Hz, 2H), 6.32 (s, 1H), 3.91 – 3.50 (m, 2H), 2.38 – 2.27 (m, 2H), 2.18 – 2.05 (m, 1H), 1.90 – 1.78 (m, 1H), 1.39 – 1.35 (m, 1H), 1.29 (s, 9H), 1.08 (s, 9H), 0.92 – 0.44 (m, 4H) ppm; **^{13}C{^{1}H} NMR** (100 MHz,

Chloroform-*d*) δ 161.5, 154.9, 149.6, 145.9, 144.1, 143.0, 140.8, 139.3, 137.6, 131.8, 131.7, 131.1, 130.6, 130.0, 129.3, 128.3, 128.1, 127.5, 127.4, 126.4, 126.3, 125.7, 123.7, 122.9, 120.4, 119.2, 79.1, 70.3, 57.9, 45.4, 44.2, 34.4, 34.0, 33.9, 32.6, 31.4, 28.6 ppm, one resonance was not observed due to overlapping peaks; **IR** (thin film): 3005, 2966, 1687, 1449, 1276, 1169, 1018, 913, 749, 652 cm^{-1}; **HRMS** calc'd for $C_{48}H_{53}N_2O_2^+$ 689.4102, found 689.4105 $[M+H]^+$.

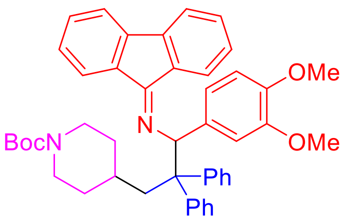

***tert*-Butyl 4-(3-((9*H*-fluoren-9-ylidene)amino)-3-(3,4-dimethoxy-phenyl)-2,2-diphenylpropyl)piperi-dine-1-carboxylate (4fm):** The reaction was performed following the general procedure with (*E*)-1-(3,4-dimethoxyphenyl)-*N*-(9*H*-fluoren-9-yl)methanimine **1f** (329.1 mg, 1.0 mmol), 1-(*tert*-butyl) 4-(1,3-dioxoisoindolin- 2-yl) piperidine-1,4-dicarboxylate **2m** (561.2 mg, 1.5 mmol) and ethene-1,1-diyldibenzene **3a** (540.8 mg, 3.0 mmol). The crude product was separated by flash chromatography on deactivated silica gel (hexanes:ethyl acetate = 8:1). Further purification was performed on an Agilent HPLC 1260 system using acetonitrile:H_2O (98:2 vol./vol.) as mobile phase and flow rate of 5 mL/min with monitoring at 254 nm to give the product **4fm** (567.7 mg, 82% yield) as a yellow oil. **R_f** = 0.31 (hexanes:ethyl acetate = 5:1). **^{1}H NMR** (400 MHz, Chloroform-*d*) 7.92 (d, *J* = 7.6 Hz, 1H), 7.58 (d, *J* = 7.2 Hz, 1H), 7.42 – 7.31 (m, 3H), 7.22 – 7.12 (m, 6H), 7.11 – 7.05 (m, 4H), 6.99 – 6.90 (m, 3H), 6.70 (d, *J* = 8.0 Hz, 1H), 6.53 (d, *J* = 8.0 Hz, 1H), 6.30 (s, 1H), 5.67 (s, 1H), 3.82 – 3.57 (m, 5H), 3.18 (s, 3H), 2.39 – 2.26 (m, 2H), 2.20 – 2.06 (m, 1H), 1.90 – 1.76 (m, 1H), 1.39 – 1.31 (m, 1H), 1.28 (s, 9H), 0.88 – 0.73 (m, 4H) ppm; **^{13}C{^{1}H} NMR** (100 MHz, Chloroform-*d*) δ 161.7, 154.9, 147.8, 147.6, 145.8, 144.2, 142.8, 140.8, 139.3, 133.1, 132.0, 131.7, 131.2, 130.7, 129.9, 128.3, 128.1, 127.6, 127.5, 126.5, 126.4, 125.8, 122.7, 122.0, 120.5, 119.3, 113.0, 109.1, 79.1, 69.9, 57.9, 55.6, 55.2, 45.5, 44.1, 34.0, 33.8, 32.5, 28.5 ppm, one resonance was not observed due to overlapping peaks; **IR** (thin film): 3005, 2978, 1686, 1449, 1275, 1167, 1029, 913, 749, 653 cm^{-1}; **HRMS** calc'd for $C_{46}H_{49}N_2O_4^+$ 693.3687, found 693.3689 $[M+H]^+$.

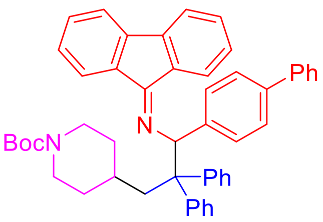

***tert*-Butyl 4-(3-((9*H*-fluoren-9-ylidene)amino)-3-([1,1'-biphenyl]-4-yl)-2,2-diphenylpropyl)piperidine-1-carboxylate (4gm):** The reaction was performed following the general procedure with (*E*)-1-([1,1'-biphenyl]-3-yl)-*N*-(9*H*-fluoren-9-yl)methanimine **1g** (345.2 mg, 1.0 mmol), 1-(*tert*-butyl) 4-(1,3-dioxoisoindolin-2-yl) piperidine-1,4-dicarboxylate **2m** (561.2 mg, 1.5 mmol) and ethene-1,1-diyldibenzene **3a** (540.8 mg, 3.0 mmol). The crude product was separated by flash chromatography on deactivated silica gel (hexanes:ethyl acetate = 10:1). Further purification by recrystallization (hexanes:MeOH = 10:1) give the product **4gm** (602.1 mg, 85% yield) as a yellow solid. **Mp**: 182 – 184 °C. **R_f** = 0.31 (hexanes:ethyl acetate = 10:1). **^{1}H NMR** (400 MHz, Chloroform-*d*) 7.89 (d, *J* = 7.2 Hz, 1H), 7.62 (d, *J* = 7.6 Hz, 1H), 7.40 – 7.30 (m, 6H), 7.21 – 7.07 (m, 14H), 7.01 – 6.93 (m, 3H), 7.00 (d, *J* = 8.0 Hz, 2H), 6.38 (s, 1H), 3.91 – 3.52 (m, 2H), 2.39 – 2.27 (m, 2H), 2.22 – 2.09 (m, 1H), 1.96 – 1.79 (m, 1H), 1.46 – 1.36 (m, 1H), 1.28 (s, 9H), 1.04 – 0.45 (m, 4H) ppm; **^{13}C{^{1}H} NMR** (100 MHz, Chloroform-*d*) δ 161.9, 154.9, 145.8, 144.2, 142.7, 140.9, 140.7, 139.9, 139.5, 139.3, 131.9, 131.6, 131.3, 130.8, 130.2, 130.0, 128.8, 128.4, 128.2, 127.6, 127.5, 127.2, 127.0, 126.52, 126.48, 125.9, 125.6, 123.0, 120.5, 119.3, 79.1, 70.4, 58.0, 45.5, 44.7, 34.1, 33.9, 32.6, 28.6 ppm, one resonance was not observed due to overlapping peaks; **IR**

(thin film): 3029, 2976, 1686, 1449, 1275, 1168, 1008, 913, 748, 653 cm^{-1}; **HRMS** calc'd for $C_{50}H_{49}N_2O_2^+$ 709.3789, found 709.3791 [M+H]$^+$.

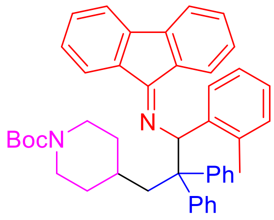

***tert*-Butyl 4-(3-((9*H*-fluoren-9-ylidene)amino)-2,2-diphenyl-3-(*o*-tolyl) propyl)piperidine-1-carboxylate (4hm):** The reaction was performed following the general procedure with (*E*)-(2-methylbenzyl)-9*H*-fluoren-9-imine **1h** (283.1 mg, 1.0 mmol), 1-(*tert*-butyl) 4-(1,3-dioxoisoindolin-2-yl) piperidine-1,4-dicarboxylate **2m** (561.2 mg, 1.5 mmol) and ethene-1,1-diyldibenzene **3a** (540.8 mg, 3.0 mmol). The crude product was separated by flash chromatography on deactivated silica gel (hexanes:ethyl acetate = 10:1). Further purification by recrystallization (hexanes:MeOH = 10:1) give the product **4hm** (400.8 mg, 62% yield) as a yellow solid. **Mp**: 137 – 139 °C. $\mathbf{R_f}$ = 0.32 (hexanes:ethyl acetate = 10:1). **^{1}H NMR** (400 MHz, Chloroform-*d*) 7.87 (d, *J* = 7.6 Hz, 1H), 7.58 (d, *J* = 7.2 Hz, 1H), 7.43 (d, *J* = 7.6 Hz, 1H), 7.35 – 7.08 (m, 13H), 7.03 – 6.84 (m, 5H), 6.58 – 6.19 (m, 2H), 3.80 – 3.51 (m, 2H), 2.46 – 2.14 (m, 5H), 1.81 – 1.76 (m, 1H), 1.34 – 1.05 (m, 13H), 0.50 – 0.14 (m, 2H) ppm; **^{13}C{^{1}H} NMR** (100 MHz, Chloroform-*d*) δ 159.5, 153.7, 144.5, 143.0, 140.0, 139.5, 138.3, 137.7, 131.3, 130.4, 130.3, 129.9, 129.4, 129.1, 128.6, 127.1, 126.7, 126.5, 125.5, 125.4, 125.3, 124.8, 123.3, 121.8, 119.3, 118.0, 77.9, 66.8, 59.3, 44.5, 42.9, 33.3, 32.4, 31.3, 27.4, 20.0 ppm, three resonances were not observed due to overlapping peaks; **IR** (thin film): 3057, 2929, 1685, 1448, 1287, 1168, 911, 731, 652 cm^{-1}; **HRMS** calc'd for $C_{45}H_{47}N_2O_2^+$ 647.3632, found 647.3634 [M+H]$^+$.

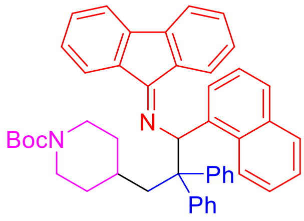

***tert*-Butyl 4-(3-((9*H*-fluoren-9-ylidene)amino)-3-(naphthalen-1-yl)-2,2-diphenylpropyl)piperidine-1-carboxylate (4im):** The reaction was performed following the general procedure with (*E*)-*N*-(9*H*-fluoren-9-yl)-1-(naphthalen-1-yl)methanimine **1i** (319.1 mg, 1.0 mmol), 1-(*tert*-butyl) 4-(1,3-dioxoisoindolin-2-yl) piperidine-1,4-dicarboxylate **2m** (561.2 mg, 1.5 mmol) and ethene-1,1-diyldibenzene **3a** (540.8 mg, 3.0 mmol). The crude product was separated by flash chromatography on deactivated silica gel (hexanes:ethyl acetate = 10:1). Further purification by recrystallization (hexanes:MeOH = 10:1) give the product **4im** (477.7 mg, 70% yield) as a yellow solid. **Mp**: 173 – 175 °C. $\mathbf{R_f}$ = 0.40 (hexanes:ethyl acetate = 10:1). **^{1}H NMR** (400 MHz, Chloroform-*d*) 8.51 (d, *J* = 8.4 Hz, 1H), 7.83 (d, *J* = 7.6 Hz, 1H), 7.70 (d, *J* = 8.4 Hz, 1H), 7.61 – 7.54 (m, 2H), 7.45 (d, *J* = 8.0 Hz, 1H), 7.39 – 7.32 (m, 4H), 7.27 – 7.19 (m, 7H), 7.14 – 7.12 (m, 2H), 7.06 – 6.92 (m, 4H), 6.84 (t, *J* = 7.6 Hz, 1H), 6.76 (t, *J* = 7.6 Hz, 1H), 6.15 (d, *J* = 7.2 Hz, 1H), 3.78 – 3.38 (m, 2H), 2.41 – 2.34 (m, 1H), 2.19 – 2.07 (m, 2H), 1.87 – 1.81 (m, 1H), 1.35 – 1.13 (m, 12H), 0.93 – 0.82 (m, 1H), 0.49 – 0.38 (m, 1H) ppm; **^{13}C{^{1}H} NMR** (100 MHz, Chloroform-*d*) δ 160.2, 153.6, 145.0, 142.8, 139.7, 139.6, 138.4, 135.8, 132.0, 131.6, 131.0, 130.2, 129.8, 129.4, 128.6, 128.5, 127.1, 126.8, 126.5, 126.4, 125.6, 125.4, 125.0, 124.7, 123.7, 122.6, 121.7, 119.1, 118.0, 77.9, 64.1, 59.9, 45.4, 42.8, 33.1, 32.4, 31.4, 27.4 ppm, three resonances were not observed due to overlapping peaks; **IR** (thin film): 3057, 2926, 2246, 1686, 1449, 1288, 1168, 910, 731, 654 cm^{-1}; **HRMS** calc'd for $C_{48}H_{47}N_2O_2^+$ 683.3632, found 683.3634 [M+H]$^+$.

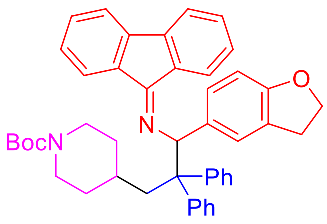

tert*-Butyl 4-(3-((9*H*-fluoren-9-ylidene)amino)-3-(2,3-dihydrobenzo-furan-6-yl)-2,2-diphenylpropyl)piperidine-1-carboxylate (4jm):** The reaction was performed following the general procedure with (*E*)-1-(2,3-dihydrobenzofuran-6-yl)-*N*-(9*H*-fluoren-9-yl)methanimine **1j** (311.1 mg, 1.0 mmol), 1-(*tert*-butyl) 4-(1,3-dioxoisoindolin-2-yl) piperidine-1,4-dicarboxylate **2m** (561.2 mg, 1.5 mmol) and ethene-1,1-diyldibenzene **3a** (540.8 mg, 3.0 mmol). The crude product was separated by flash chromatography on deactivated silica gel (hexanes:ethyl acetate = 8:1). Further purification by recrystallization (hexanes:MeOH = 10:1) give the product **4jm** (613.7 mg, 91% yield) as a yellow solid. **Mp**: 218 – 220 °C. **R*f = 0.31 (hexanes:ethyl acetate = 10:1). **^{1}H NMR** (400 MHz, Chloroform-*d*) 7.90 (d, *J* = 7.6 Hz, 1H), 7.60 (d, *J* = 7.2 Hz, 1H), 7.47 (d, *J* = 7.6 Hz, 1H), 7.38 – 7.35 (m, 3H), 7.25 (t, *J* = 7.6 Hz, 1H), 7.22 – 7.16 (m, 5H), 7.13 – 7.09 (m, 3H), 7.02 – 6.96 (m, 3H), 6.65 (d, *J* = 8.4 Hz, 1H), 6.41 (d, *J* = 8.0 Hz, 1H), 6.27 (s, 1H), 6.10 (s, 1H), 4.33 – 4.22 (m, 2H), 3.88 – 3.56 (m, 2H), 2.74 (t, *J* = 8.4 Hz, 2H), 2.40 – 2.27 (m, 2H), 2.16 – 2.04 (m, 1H), 1.87 – 1.75 (m, 1H), 1.42 – 1.35 (m, 1H), 1.30 (s, 9H), 1.03 – 0.47 (m, 4H) ppm; **^{13}C{^{1}H} NMR** (100 MHz, Chloroform-*d*) δ 161.4, 158.8, 154.9, 145.9, 144.1, 142.8, 140.8, 139.3, 132.6, 132.0, 131.6, 131.1, 130.6, 129.9, 129.4, 128.2, 128.1, 127.5, 127.4, 126.4, 126.2, 125.70, 125.66, 122.9, 120.4, 119.2, 107.2, 79.1, 71.1, 70.1, 58.0, 45.4, 44.1, 34.0, 33.8, 32.5, 29.6, 28.5 ppm, two resonances were not observed due to overlapping peaks; **IR** (thin film): 3006, 2989, 1685, 1490, 1276, 1168, 913, 749, 655 cm^{-1}; **HRMS** calc'd for $C_{46}H_{47}N_2O_3^+$ 675.3581, found 675.3583 [M+H]$^+$.

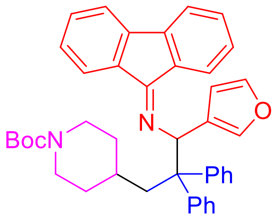

tert*-Butyl 4-(3-((9*H*-fluoren-9-ylidene)amino)-3-(furan-3-yl)-2,2-di-phenylpropyl)piperidine-1-car-boxylate (4km):** The reaction was performed following the general procedure with (*E*)-*N*-(9*H*-fluoren-9-yl)-1-(furan-3-yl)methanimine **1k** (259.1 mg, 1.0 mmol), 1-(*tert*-butyl) 4-(1,3-dioxoisoindolin-2-yl)piperidine-1,4- dicarboxylate **2m** (561.2 mg, 1.5 mmol) and ethene-1,1-diyldibenzene **3a** (540.8 mg, 3.0 mmol). The crude product was separated by flash chromatography on deactivated silica gel (hexanes:ethyl acetate = 20:1). Further purification was performed on an Agilent HPLC 1260 system using acetonitrile:H_2O (98:2 vol./vol.) as mobile phase and flow rate of 5 mL/min with monitoring at 254 nm to give the product **4km** (547.6 mg, 88% yield) as a yellow oil. **R*f = 0.44 (hexanes:ethyl acetate = 10:1). **^{1}H NMR** (400 MHz, Chloroform-*d*) 7.77 (d, *J* = 7.6 Hz, 1H), 7.62 (d, *J* = 7.2 Hz, 1H), 7.53 (d, *J* = 7.6 Hz, 1H), 7.45 – 7.43 (m, 3H), 7.32 – 7.25 (m, 2H), 7.23 – 7.14 (m, 5H), 7.11 – 7.04 (m, 5H), 6.94 (s, 1H), 6.73 (s, 1H), 6.29 (s, 1H), 5.51 (s, 1H), 3.87 – 3.57 (m, 2H), 2.40 – 2.32 (m, 2H), 2.22 – 2.12 (m, 1H), 2.04 – 1.88 (m, 1H), 1.47 – 1.37 (m, 1H), 1.30 (s, 9H), 1.06 – 0.56 (m, 4H) ppm, three resonances were not observed due to overlapping peaks; **^{13}C{^{1}H} NMR** (100 MHz, Chloroform-*d*) δ 161.2, 153.8, 144.0, 143.0, 140.1, 139.8, 139.7, 138.0, 130.4, 130.2, 129.9, 129.7, 129.1, 127.3, 127.0, 126.6, 126.1, 125.8, 125.2, 124.8, 123.2, 121.8, 119.4, 118.2, 110.1, 78.0, 62.7, 56.0, 44.1, 43.0, 32.9, 31.4, 27.4 ppm; **IR** (thin film): 3409, 2976, 1682, 1449, 1276, 1167, 1076, 913, 748, 654 cm^{-1}; **HRMS** calc'd for $C_{42}H_{43}N_2O_3^+$ 623.3268, found 623.3266 [M+H]$^+$.

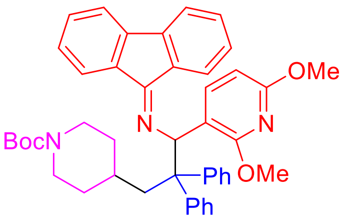

tert*-Butyl 4-(3-((9*H*-fluoren-9-ylidene)amino)-3-(2,6-dimethoxypyridin-3-yl)-2,2-diphenylpropyl) piperidine-1-carboxylate (4lm):** The reaction was performed following the general procedure with (*E*)-1-(2,6-dimethoxypyridin-3-yl)-*N*-(9*H*-fluoren-9-yl)methanimine **1l** (330.4 mg, 1.0 mmol), 1-(*tert*-butyl) 4-(1,3-dioxoisoindolin-2-yl) piperidine-1,4-dicarboxylate **2m** (561.2 mg, 1.5 mmol) and ethene-1,1-diyldibenzene **3a** (540.8 mg, 3.0 mmol). The crude product was separated by flash chromatography on deactivated silica gel (hexanes:ethyl acetate = 8:1). Further purification by recrystallization (hexanes:MeOH = 10:1) give the product **4lm** (589.4 mg, 85% yield) as a white solid. **Mp**: 189 – 191 °C. **R*f = 0.38 (hexanes:ethyl acetate = 10:1). **^{1}H NMR** (400 MHz, Chloroform-*d*) 8.23 (d, *J* = 7.2 Hz, 1H), 7.53 (d, *J* = 7.6 Hz, 1H), 7.43 – 7.39 (m, 2H), 7.32 (d, *J* = 7.2 Hz, 1H), 7.25 – 7.12 (m, 8H), 7.09 – 7.03 (m, 2H), 6.99 – 6.90 (m, 3H), 6.74 (s, 1H), 5.89 (d, *J* = 8.4 Hz, 1H), 5.64 (d, *J* = 8.4 Hz, 1H), 4.01 (s, 3H), 3.86 – 3.53 (m, 5H), 2.44 – 2.37 (m, 1H), 2.32 – 2.28 (m, 1H), 2.03 – 1.86 (m, 1H), 1.86 – 1.77 (m, 1H), 1.44 – 1.38 (m, 1H), 1.29 (s, 9H), 0.87 – 0.21 (m, 4H) ppm; **^{13}C{^{1}H} NMR** (100 MHz, Chloroform-*d*) δ 160.5, 160.4, 157.8, 153.7, 145.1, 142.9, 141.8, 141.0, 139.5, 138.2, 131.3, 130.5, 130.0, 129.4, 128.6, 127.1, 127.0, 126.4, 125.7, 125.3, 125.1, 124.6, 121.7, 119.1, 118.0, 113.2, 99.5, 77.9, 60.6, 57.7, 52.2, 43.9, 42.9, 32.9, 32.7, 31.3, 27.4 ppm, two resonances were not observed due to overlapping peaks; **IR** (thin film): 3060, 2938, 1687, 1400, 1356, 1280, 1100, 919, 753, 653 cm^{-1};**HRMS** calc'd for $C_{45}H_{48}N_3O_4^+$ 694.3639, found 694.3644 $[M+H]^+$.

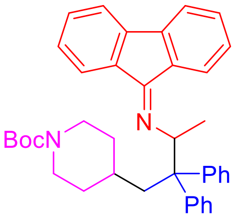

tert*-Butyl 4-(3-((9*H*-fluoren-9-ylidene)amino)-2,2-diphenylbutyl)piperidine-1-carboxylate (4mm):** The reaction was performed following the general procedure with *N*-(9*H*-fluoren-9-yl)ethanimine **1m** (207.1 mg, 1.0 mmol), 1-(*tert*-butyl) 4-(1,3-dioxoisoindolin-2-yl)piperidine-1,4- dicarboxylate **2m** (561.2 mg, 1.5 mmol) and ethene-1,1-diyldibenzene **3a** (540.8 mg, 3.0 mmol). The crude product was separated by flash chromatography on deactivated silica gel (hexanes:ethyl acetate = 20:1). Further purification was performed on an Agilent HPLC 1260 system using acetonitrile:H_2O (95:5 vol./vol.) as mobile phase and flow rate of 5 mL/min with monitoring at 254 nm to give the product **4mm** (433.4 mg, 76% yield) as a yellow oil. **R*f = 0.37 (hexanes:ethyl acetate = 10:1). **^{1}H NMR** (400 MHz, Chloroform-*d*) 7.89 (d, *J* = 7.6 Hz, 1H), 7.61 (d, *J* = 7.6 Hz, 1H), 7.56 (d, *J* = 7.6 Hz, 1H), 7.45 – 7.40 (m, 3H), 7.32 (t, *J* = 7.6 Hz, 1H), 7.26 – 7.22 (m, 6H), 7.17 – 7.12 (m, 4H), 7.09 – 7.05 (m, 1H), 5.46 (q, *J* = 6.4 Hz, 1H), 3.80 – 3.44 (m, 2H), 2.29 – 2.17 (m, 3H), 1.95 – 1.85 (m, 1H), 1.26 (s, 9H), 1.23 – 1.18 (m, 1H), 1.05 (d, *J* = 6.4 Hz, 3H), 0.88 – 0.57 (m, 4H) ppm; **^{13}C{^{1}H} NMR** (100 MHz, Chloroform-*d*) δ 159.6, 153.7, 144.3, 144.1, 142.9, 139.4, 137.9, 130.4, 130.1, 129.5, 129.4, 129.1, 127.2, 127.1, 126.3, 125.9, 125.8, 125.1, 124.8, 121.7, 119.5, 118.1, 77.9, 58.4, 54.8, 44.8, 42.9, 32.9, 32.8, 31.1, 27.4, 16.2 ppm; **IR** (thin film): 2976, 1686, 1599, 1448, 1365, 1261, 1168, 1076, 912, 764, 702, 654 cm^{-1}; **HRMS** calc'd for $C_{39}H_{43}N_2O_2^+$ 571.3315, found 571.3319 $[M+H]^+$.

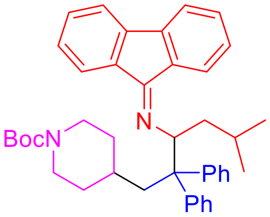

***tert*-Butyl 4-(3-((9*H*-fluoren-9-ylidene)amino)-5-methyl-2,2-diphenylhexyl)piperidine-1-carboxylate (4nm):** The reaction was performed following the general procedure with *N*-(9*H*-fluoren-9-yl)-3-methylbutan-1-imine **1n** (249.2 mg, 1.0 mmol), 1-(*tert*-butyl) 4-(1,3-dioxoisoindolin-2-yl)piperidine-1,4- dicarboxylate **2m** (561.2 mg, 1.5 mmol) and ethene-1,1-diyldibenzene **3a** (540.8 mg, 3.0 mmol). The crude product was separated by

flash chromatography on deactivated silica gel (hexanes:ethyl acetate = 20:1). Further purification was performed on an Agilent HPLC 1260 system using acetonitrile:H_2O (95:5 vol./vol.) as mobile phase and flow rate of 5 mL/min with monitoring at 254 nm to give the product **4nm** (410.3 mg, 67% yield) as a yellow oil. **R*f*** = 0.36 (hexanes:ethyl acetate = 10:1). **^{1}H NMR** (400 MHz, Chloroform-*d*) 8.23 (d, *J* = 7.6 Hz, 1H), 7.71 (d, *J* = 7.2 Hz, 1H), 7.62 (d, *J* = 7.6 Hz, 1H), 7.53 (d, *J* = 7.2 Hz, 1H), 7.39 – 7.14 (m, 14H), 5.47 (s, 1H), 3.57 – 3.37 (m, 2H), 2.17 – 1.90 (m, 4H), 1.63 – 1.50 (m, 2H), 1.31 – 1.06 (m, 15H), 0.71 – 0.63 (m, 4H), 0.42 – 0.26 (m, 2H) ppm; **^{13}C{^{1}H} NMR** (100 MHz, Chloroform-*d*) δ 158.8, 153.5, 144.6, 144.2, 143.2, 139.3, 137.9, 131.4, 130.0, 129.53, 129.49, 128.9, 127.3, 126.8, 126.6, 125.7, 125.2, 125.0, 121.8, 119.5, 118.2, 77.8, 65.5, 55.7, 45.7, 43.1, 42.1, 34.4, 33.3, 32.2, 30.9, 27.3, 26.7, 26.2, 25.3, 25.2 ppm; **IR** (thin film): 2924, 1687, 1448, 1275, 1171, 913, 764, 701 cm^{-1}; **HRMS** calc'd for $C_{42}H_{49}N_2O_2^+$ 613.3788, found 613.3789 [M+H]$^+$.

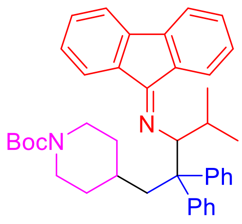

tert*-Butyl 4-(3-((9*H*-fluoren-9-ylidene)amino)-4-methyl-2,2-diphenyl-pentyl)piperidine-1-carboxylate (4om):** The reaction was performed following the general procedure with *N*-(9*H*-fluoren-9-yl)-2-methylpropan-1-imine **1o** (235.1 mg, 1.0 mmol), 1-(*tert*-butyl) 4-(1,3-dioxoisoindolin-2-yl)piperidine-1,4- dicarboxylate **2m** (561.2 mg, 1.5 mmol) and ethene-1,1-diyldibenzene **3a** (540.8 mg, 3.0 mmol). The crude product was separated by flash chromatography on deactivated silica gel (hexanes:ethyl acetate = 20:1). Further purification was performed on an Agilent HPLC 1260 system using acetonitrile:H_2O (95:5 vol./vol.) as mobile phase and flow rate of 5 mL/min with monitoring at 254 nm to give the product **4om** (430.8.6 mg, 72% yield) as a yellow oil. **R*f = 0.37 (hexanes:ethyl acetate = 10:1). **^{1}H NMR** (400 MHz, Chloroform-*d*) 8.22 (d, *J* = 7.6 Hz, 1H), 7.71 (d, *J* = 7.6 Hz, 1H), 7.60 (d, *J* = 7.6 Hz, 1H), 7.51 (d, *J* = 7.2 Hz, 1H), 7.41 (d, *J* = 8.0 Hz, 2H), 7.35 – 7.16 (m, 12H), 5.54 (s, 1H), 3.55 – 3.45 (m, 2H), 2.34 – 2.30 (m, 1H), 2.19 – 2.14 (m, 1H), 2.06 – 1.98 (m, 1H), 1.83 – 1.54 (m, 2H), 1.23 – 1.14 (m, 10H), 1.01 (d, *J* = 6.0 Hz, 3H), 0.76 – 0.08 (m, 4H), -0.18 (d, *J* = 6.0 Hz, 3H) ppm; **^{13}C{^{1}H} NMR** (100 MHz, Chloroform-*d*) δ 159.2, 153.5, 144.4, 144.1, 143.3, 139.3, 137.8, 131.4, 130.0, 129.6, 129.5, 128.9, 127.3, 126.8, 126.6, 125.8, 125.7, 125.2, 124.9, 121.8, 119.5, 118.1, 77.8, 64.7, 55.6, 45.8, 42.8, 33.3, 32.2, 31.1, 30.9, 27.3, 23.8, 15.6 ppm; **IR** (thin film): 2960, 1685, 1496, 1365, 1275, 1166, 913, 764, 701 cm^{-1}; **HRMS** calc'd for $C_{41}H_{47}N_2O_2^+$ 599.3635, found 599.3632 [M+H]$^+$.

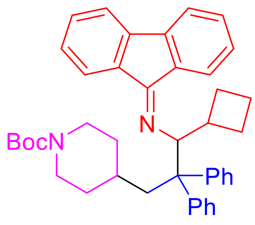

tert*-Butyl 4-(3-((9*H*-fluoren-9-ylidene)amino)-3-cyclobutyl-2,2-diphenylpropyl)piperidine-1-carboxylate (4pm):** The reaction was performed following the general procedure with 1-cyclobutyl-*N*-(9*H*-fluoren-9-yl)methanimine **1p** (247.1 mg, 1.0 mmol), 1-(*tert*-butyl) 4-(1,3-dioxoisoindolin-2-yl)piperidine-1,4- dicarboxylate **2m** (561.2 mg, 1.5 mmol) and ethene-1,1-diyldibenzene **3a** (540.8 mg, 3.0 mmol). The crude product was separated by flash chromatography on deactivated silica gel (hexanes:ethyl acetate = 20:1). Further purification was performed on an Agilent HPLC 1260 system using acetonitrile:H_2O (95:5 vol./vol.) as mobile phase and flow rate of 5 mL/min with monitoring at 254 nm to give the product **4pm** (451.7 mg, 74% yield) as a yellow oil. **R*f = 0.36 (hexanes:ethyl acetate = 10:1). **^{1}H NMR** (400 MHz, Chloroform-*d*) 8.10 (d, *J* = 7.6 Hz, 1H), 7.70 (d, *J* = 7.2 Hz, 1H), 7.59 (d, *J* = 7.2 Hz, 1H), 7.48 (d, *J* = 7.6 Hz, 1H), 7.42 (d, *J* = 8.0 Hz, 2H), 7.34 – 7.15 (m, 9H), 7.07 – 6.99 (m, 3H), 5.49 (d, *J* = 3.2 Hz, 1H), 3.74 – 3.37 (m, 2H), 2.80 – 2.73 (m, 1H), 2.25 – 2.21 (m, 1H), 2.08 – 1.93 (m, 2H), 1.79 – 1.60 (m, 3H), 1.46 – 1.35

(m, 2H), 1.24 (s, 9H), 1.19 – 1.16 (m, 2H), 0.84 – 0.15 (m, 5H) ppm; **$^{13}C\{^1H\}$ NMR** (100 MHz, Chloroform-*d*) δ 161.0, 154.7, 145.5, 144.9, 144.4, 140.5, 139.0, 132.8, 131.1, 130.7, 130.3, 130.2, 128.4, 128.0, 127.5, 127.2, 127.0, 126.3, 125.8, 122.9, 120.7, 119.3, 79.0, 66.1, 56.1, 46.4, 44.1, 39.2, 34.3, 33.5, 32.3, 28.4, 27.0, 23.7, 19.2 ppm; **IR** (thin film): 3057, 2930, 1685, 1476, 1365, 1251, 1169, 912, 932, 701, 649 cm^{-1}; **HRMS** calc'd for $C_{42}H_{47}N_2O_2^+$ 611.3629, found 611.3632 $[M+H]^+$.

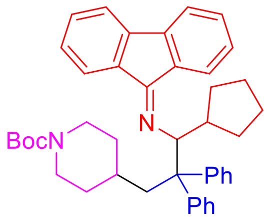

***tert*-Butyl 4-(3-((9*H*-fluoren-9-ylidene)amino)-3-cyclopentyl-2,2-diphenyl-propyl)piperidine-1-carboxylate (4qm):** The reaction was performed following the general procedure with 1-cyclopentyl-*N*-(9*H*-fluoren-9-yl)methanimine **1q** (261.2 mg, 1.0 mmol), 1-(*tert*-butyl) 4-(1,3-dioxoisoindolin-2-yl)piperidine-1,4- dicarboxylate **2m** (561.2 mg, 1.5 mmol) and ethene-1,1-diyldibenzene **3a** (540.8 mg, 3.0 mmol). The crude product was separated by flash chromatography on deactivated silica gel (hexanes:ethyl acetate = 20:1). Further purification was performed on an Agilent HPLC 1260 system using acetonitrile:H_2O (95:5 vol./vol.) as mobile phase and flow rate of 5 mL/min with monitoring at 254 nm to give the product **4qm** (434.1 mg, 68% yield) as a yellow oil. **R_f** = 0.36 (hexanes:ethyl acetate = 10:1). **^1H NMR** (400 MHz, Chloroform-*d*) 8.18 (d, *J* = 8.0 Hz, 1H), 7.71 (d, *J* = 7.2 Hz, 1H), 7.62 (d, *J* = 7.6 Hz, 1H), 7.51 (d, *J* = 7.2 Hz, 1H), 7.44 (d, *J* = 8.0 Hz, 2H), 7.36 – 7.23 (m, 9H), 7.18 – 7.09 (m, 3H), 5.76 (s, 1H), 3.61 – 3.35 (m, 2H), 2.41 – 2.33 (m, 1H), 2.20 – 2.16 (m, 1H), 2.04 – 1.95 (m, 1H), 1.86 – 1.65 (m, 3H), 1.32 – 1.07 (m, 16H), 0.81 – 0.70 (m, 3H), 0.43 – 0.32 (m, 1H), 0.16 – 0.06 (m, 1H) ppm; **$^{13}C\{^1H\}$ NMR** (100 MHz, Chloroform-*d*) δ 159.3, 153.5, 144.41, 144.35, 143.3, 139.3, 137.9, 131.5, 130.0, 129.6, 129.0, 127.3, 126.8, 126.6, 125.9, 125.8, 125.2, 124.9, 121.8, 119.6, 118.2, 77.8, 63.3, 55.4, 45.9, 43.0, 33.3, 32.2, 32.0, 31.0, 27.4, 23.9, 23.6, 23.2 ppm; **IR** (thin film): 2933, 1683, 1448, 1275, 1168, 913, 748, 701, 649 cm^{-1}; **HRMS** calc'd for $C_{43}H_{49}N_2O_2^+$ 625.3785, found 625.3789 $[M+H]^+$.

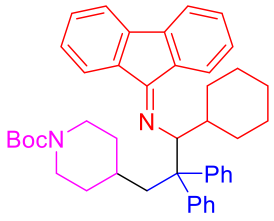

***tert*-Butyl 4-(3-((9*H*-fluoren-9-ylidene)amino)-3-cyclohexyl-2,2-diphenyl-propyl)piperidine-1-carboxylate (4rm):** The reaction was performed following the general procedure with 1-cyclohexyl-*N*-(9*H*-fluoren-9-yl)methanimine **1r** (275.2 mg, 1.0 mmol), 1-(*tert*-butyl) 4-(1,3-dioxoisoindolin-2-yl)piperidine-1,4- dicarboxylate **2m** (561.2 mg, 1.5 mmol) and ethene-1,1-diyldibenzene **3a** (540.8 mg, 3.0 mmol). The crude product was separated by flash chromatography on deactivated silica gel (hexanes:ethyl acetate = 20:1). Further purification was performed on an Agilent HPLC 1260 system using acetonitrile:H_2O (95:5 vol./vol.) as mobile phase and flow rate of 5 mL/min with monitoring at 254 nm to give the product **4rm** (374.6 mg, 60% yield) as a yellow oil. **R_f** = 0.35 (hexanes:ethyl acetate = 10:1). **^1H NMR** (400 MHz, Chloroform-*d*) 8.20 (d, *J* = 8.0 Hz, 1H), 7.64 (d, *J* = 7.2 Hz, 1H), 7.61 (d, *J* = 7.6 Hz, 1H), 7.52 (d, *J* = 7.2 Hz, 1H), 7.43 – 7.27 (m, 9H), 7.24 – 7.18 (m, 5H), 5.53 (d, *J* = 9.6 Hz, 1H), 3.61 – 3.43 (m, 2H), 2.14 – 1.93 (m, 3H), 1.81 – 1.58 (m, 3H), 1.30 – 1.17 (m, 12H), 1.09 – 1.03 (m, 1H), 0.92 – 0.91 (m, 3H), 0.69 – 0.41 (m, 7H) ppm; **$^{13}C\{^1H\}$ NMR** (100 MHz, Chloroform-*d*) δ 159.3, 153.6, 144.1, 143.3, 142.9, 139.3, 138.0, 131.6, 130.1, 129.9, 129.6, 128.9, 127.2, 126.8, 126.5, 125.6, 125.5, 125.2, 124.9, 122.1, 119.6, 118.1, 77.9, 61.9, 55.7, 45.7, 42.9, 40.8, 33.1, 32.5, 31.2, 27.4, 24.0, 23.2, 21.9 ppm; **IR** (thin film): 3005, 1686, 1448, 1275, 1169, 913, 764, 702 cm^{-1}; **HRMS** calc'd for $C_{44}H_{51}N_2O_2^+$ 639.3926, found 639.3945 $[M+H]^+$.

7. Gram-scale sequential one-pot imine synthesis/cascade coupling

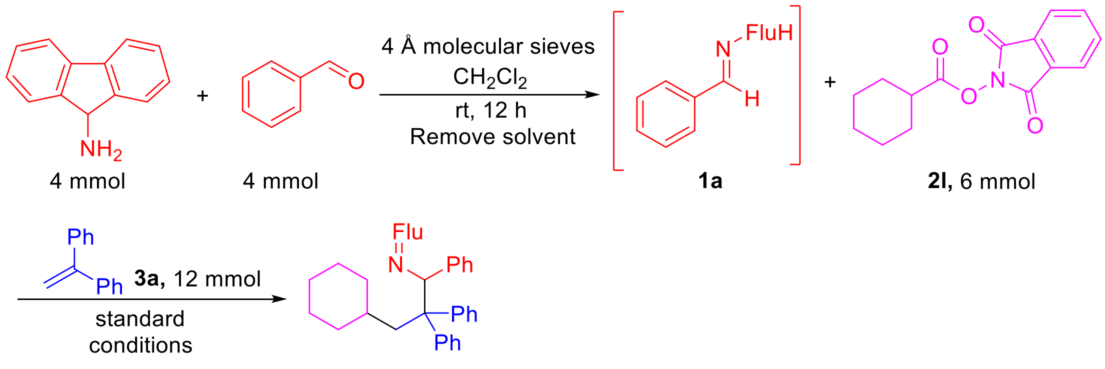

4al, 1.93g, 91% y.

Into an oven-dried reaction Schlenk tube equipped with a magnetic stirring bar was added 9*H*-fluoren-9-amine (724.9 mg, 4.0 mmol, 1 equiv.) and 4 Å molecular sieves (1.4 g, powder, <50 μM). The flask was sealed with a rubber stopper and connected to a Schlenk line through a needle. The flask was evacuated, and then refilled with nitrogen. This process was repeated twice, and the reaction flask was kept under a nitrogen atmosphere during the course of the reaction. CH_2Cl_2 (20 mL) was added under nitrogen via syringe through the rubber septum. The resulting mixture was stirred at room temperature for 10 min before the Benzaldehyde (424.5 mg, 4.0 mmol, 1 equiv.) was added under nitrogen via syringe through the rubber septum. The reaction was stirred at room temperature for 12 h, the solvent was completely removed in *vacuo* and the Schlenk tube was filled with nitrogen. A solution (prepared in the glove box) of $Ni(COD)_2$ (110.0 mg, 10 mol%) **DPPP** (330.0 mg, 20 mol%) and 1,3-dioxoisoindolin-2-yl cyclohexanecarboxylate **2l** (1229.1 mg, 6.0 mmol, 1.5 equiv) in DMF (8 mL) and THF (32 mL) was added to the Schlenk tube via syringe through the rubber septum. Then, ethene-1,1-diyldibenzene **3a** (1638.8 mg, 12.0 mmol, 3.0 equiv) were sequentially added via syringe, the mixture was stirred and until all the solids were dissolved. Then, DIPEA (1550.8 mg, 12.0 mmol, 3.0 equiv) was added to the reaction mixture. The vial was capped, removed from the glove box, and stirred for 16 h with blue LEDs whereby the temperature was maintained at approximately 27 °C via cooling with a fan. The lid of the reaction vial was opened, and quenched with 10 mL of H_2O. The layers were separated and the aqueous layer was extracted with CH_2Cl_2 (3×100 mL). The combined organic solution was washed by brine and dried over Na_2SO_4. The combined organic layers were concentrated in *vacuo*. The crude product was separated by flash chromatography on deactivated silica gel (hexanes:ethyl acetate = 200:1). Further purification by recrystallization (hexanes:MeOH = 10:1) give the product **4al** (1.93 g, 91% yield) as a yellow solid.

8. Hydrolysis of compound 4al

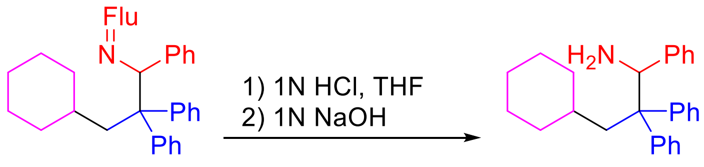

4al **5al,** 92% y.

An oven-dried microwave vial equipped with a stir bar was charged with **4al** (159.4 mg 0.3 mmol). THF (3 mL) was added to the reaction vial via syringe and the reaction was cooled at 0 ℃. HCl 1N (3 mL) was added to the reaction vial via syringe. The stirring solution was warmed to room temperature and

was monitored by TLC until **4al** was consumed (reaction completed in 3 h). The reaction mixture was basified with 1N NaOH until the pH reached 14, transferred to a 50 mL seperatory funnel via pipette and was extracted with dichloromethane (1×20 mL). The combined organic layers were concentrated in vacuo, loaded onto a deactivated silica gel column via pipette and purified by flash chromatography on deactivated silica gel (eluted with hexanes to ethyl acetate:hexanes = 1:5) to give the amine product **5al** (101.9 mg, 92% yield) as a white solid. **Mp**: 127 – 129 °C. **R$_f$** = 0.40 (hexanes:ethyl acetate = 1:1). **^{1}H NMR** (400 MHz, Chloroform-*d*) 7.34 (d, *J* = 8 Hz, 2H), 7.24 – 7.11 (m, 6H), 7.06 – 7.02 (m, 3H), 6.96 (t, *J* = 7.2 Hz, 2H), 6.42 (d, *J* = 7.2 Hz, 2H), 4.79 (s, 1H), 1.75 (dd, *J* = 14.0, 8.0 Hz, 1H), 1.49 – 1.44 (m, 2H), 1.34 – 1.23 (m, 3H), 1.10 – 0.73 (m, 6H), 0.64 – 0.56 (m, 1H), 0.50 – 0.36 (m, 2H) ppm; **^{13}C{^{1}H} NMR** (100 MHz, Chloroform-*d*) δ 145.4, 142.0, 141.3, 131.2, 129.5, 129.2, 128.0, 127.1, 126.7, 126.4, 126.2, 62.1, 57.6, 47.3, 35.3, 35.0, 34.0, 26.7, 26.5, 26.4 ppm; **IR** (thin film): 3060, 2975, 2866, 1600, 1492, 1455, 1188, 1060, 701 cm^{-1} ; **HRMS** calc'd for $C_{27}H_{32}N^+$ 370.2529, found 370.2526 [M+H]$^+$.

9. EPR experiments

X-band EPR spectrum of a carbon radical (probably alkyl radical intermediate) trapped with PBN;

(1) *Settings*: microwave frequency: 9.440904 GHz; power: 0.2 mW; center field: 336.00 mT; sweep width: 25.0 mT; modulation frequency: 93750 Hz; modulation amplitude: 100 µT.

(2) *Reaction conditions*: Ni(COD)$_2$ (2.8 mg, 0.01 mmol), **DPPP** (8.3 mg, 0.02 mmol), (*E*)-*N*-(9*H*-fluoren-9-yl)-1-phenylmethanimine **1a** (26.9 mg, 0.1 mmol), 1-(*tert*-butyl) 4-(1,3-dioxoisoindolin-2-yl)piperidine-1,4- dicarboxylate **2m** (56.1 mg, 0.15 mmol), ethene-1,1-diyldibenzene **3a** (54.1 mg, 0.3 mmol), PBN (44.3 mg, 0.25 mmol), DIPEA (38.8 mg, 0.3 mmol), DMA (1 mL), rt, blue LED, 10 min.

(3) *The resulting EPR signal:*

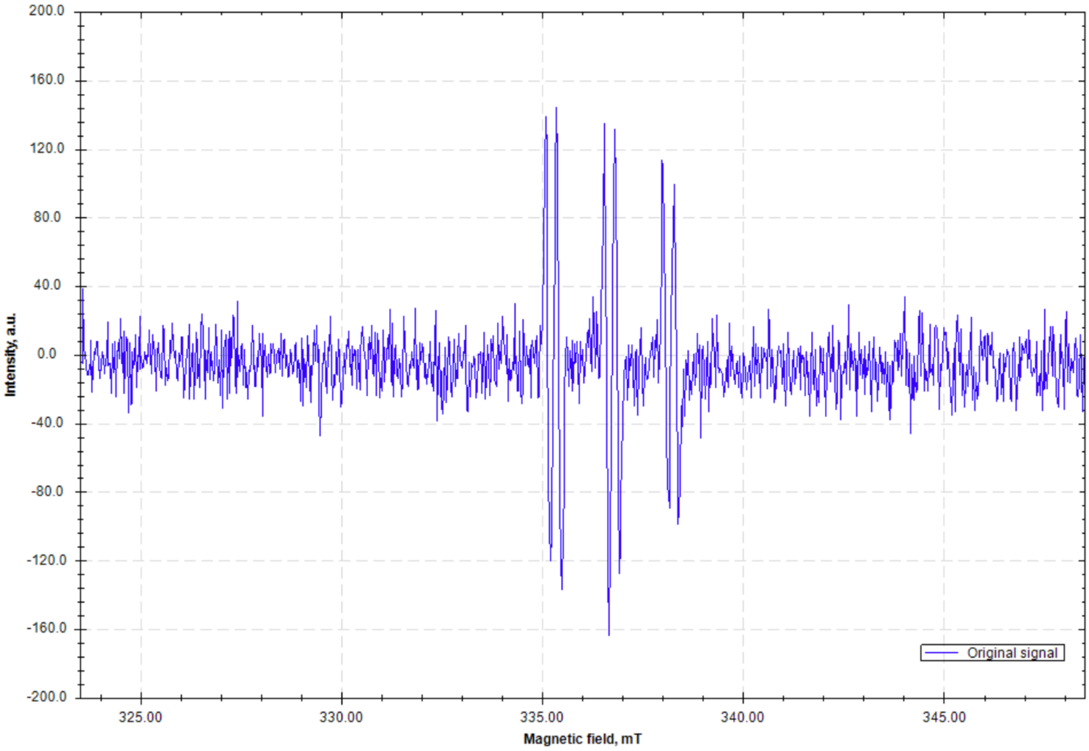

Figure 1. Isotropic X-band EPR spectrum of the PBN-trapped carbon centered radical (T = 298 K; microwave frequency: 9.440904 GHz; power: 0.2 mW; modulation amplitude: 100 µT).

10. Attempts to enantioselectivity experiments

(1) Screening of chiral ligands for the enantioselective synthesis of 4au[a]

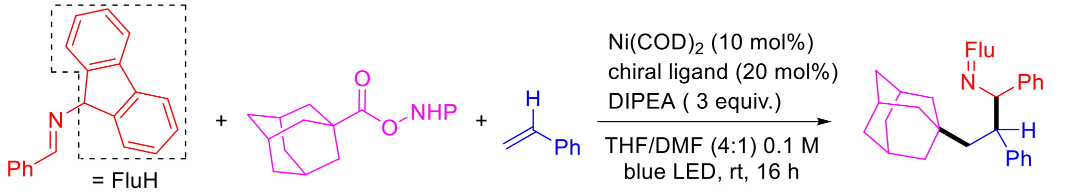

entry	ligand	CAS number	yield of **4au** (%)[b]	ee of **4au** (%)[c]	dr of **4au**[d]
1	**L1**	131833-93-7	trace	-	-
2	**L2**	131833-92-6	trace	-	-
3	**L3**	185346-17-2	8	0	1:1
4	**L4**	117408-98-7	trace	-	-
5	**L5**	64896-28-2	92	0	1.5:1
6	**L6**	157488-65-8	32	0	1:1
7	**L7**	76189-55-4	13	0	1:1
8	**L8**	99646-28-3	trace	-	-
9	**L9**	636559-55-2	0	-	-
10	**L10**	712352-08-4	trace	-	-
11	**L11**	185449-81-4	trace	-	-
12	**L12**	138517-61-0	0	-	-
13	**L13**	1439556-82-7	50	0	1:1
14	**L14**	244261-66-3	28	0	1:1
15	**L15**	37002-48-5	trace	-	-
16	**L16**	143668-57-9	0	-	-
17	**L17**	136705-65-2	12	0	1:1
18	**L18**	528565-79-9	63	0	1.2:1
19	**L19**	486429-99-6	trace	-	-
20	**L20**	885701-78-0	11	0	1:1
21	**L21**	503538-69-0	15	0	1:1
22	**L22**	252288-04-3	trace	-	-
23	**L23**	192463-40-4	32	0	1:1
24	**L24**	957782-11-5	21	0	1:1
25	**L25**	364732-86-5	25	0	1:1

[a]Reactions conducted on a 0.2 mmol scale using 1 equiv. of **1a**, 1.5 equiv. of **2a**, and 3 equiv. of **3u**, with $Ni(COD)_2$ (10 mol %), ligand (20 mol %), DIPEA (3.0 equiv.) in 2 mL of THF/DMF = 4:1 at 27 °C for 16 h. [b]Isolated yield of **4au** after chromatographic purification. [c]Ee of **4au** was determined by chiral phase HPLC. [c]Dr of **4au** was determined by HPLC.

(2) Screening of chiral ligands for the enantioselective synthesis of 4am[a]

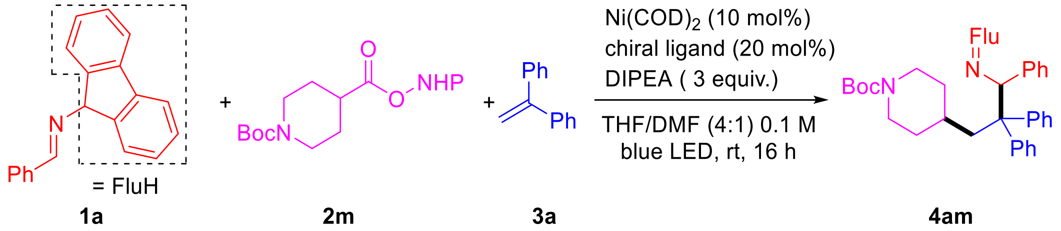

entry	ligand	CAS number	yield of **4am** (%)[b]	ee of **4am** (%)[c]
1	**L26**	1428537-19-2	48	0
2	**L27**	205647-96-7	33	0
3	**L28**	2097333-76-9	35	0
4	**L29**	280755-83-1	44	0
5	**L30**	1402851-53-9	47	0
6	**L31**	2055935-90-3	17	0
7	**L32**	135532-33-1	39	0
8	**L33**	444575-98-8	62	0
9	**L34**	195379-09-0	57	0
10	**L35**	150529-93-4	50	0
11	**L36**	1246401-48-8	44	0
12	**L37**	1246401-49-9	39	0
13	**L38**	298693-04-6	41	0
14	**L39**	1404433-37-9	51	0
15	**L40**	2271404-99-8	49	0
16	**L41**	529489-04-1	66	0
17	**L42**	229184-96-7	69	0
18	**L43**	2133827-34-4	70	0
19	**L44**	2634687-82-2	74	0
20	**L45**	185346-17-2	23	0
21	**L46**	147409-41-4	80	0
22	**L47**	180186-94-1	22	0
23	**L48**	175166-51-5	28	0
24	**L49**	1379763-05-9	<10	-
25	**L50**	394738-76-2	39	0
26	**L51**	182122-08-3	21	0
27	**L52**	2097145-90-7	40	0
28	**L53**	2005443-90-1	33	0
29	**L54**	182122-13-0	42	0
30	**L55**	NR	37	0
31	**L56**	1239015-11-2	46	0
32	**L57**	195433-00-2	51	0
33	**L58**	485394-21-6	<10	-
34	**L59**	1610785-35-7	68	0
35	**L60**	1884680-48-1	66	0
36	**L61**	1435940-19-4	75	0
37	**L62**	138517-61-0	0	-
38	**L63**	1355162-85-4	73	0

39	**L64**	2468233-72-7	66	0
40	**L65**	212312-33-9	62	0
41	**L66**	155806-35-2	22	0
42	**L67**	162291-01-2	35	0
43	**L68**	155806-35-2	33	0
44	**L69**	849923-15-5	41	0
45	**L70**	223121-07-1	25	0
46	**L71**	155830-69-6	33	0
47	**L72**	849924-43-2	19	0

[a]Reactions conducted on a 0.2 mmol scale using 1 equiv. of **1a**, 1.5 equiv. of **2m**, and 3 equiv. of **3a**, with Ni(COD)$_2$ (10 mol %), ligand (20 mol %), DIPEA (3.0 equiv.) in 2 mL of THF/DMF = 4:1 at 27 °C for 16 h. [b]Isolated yield of **4am** after chromatographic purification. [c]Ee(enantiomeric excess) of **4am** was determined by chiral phase HPLC.

72 chiral ligands:

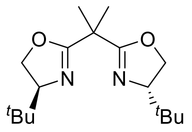
L1

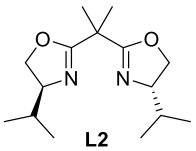
L2

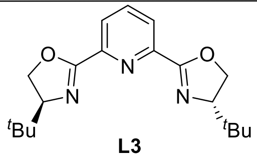
L3

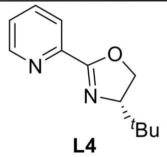
L4

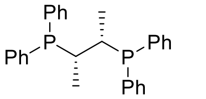
L5

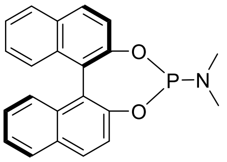
L6

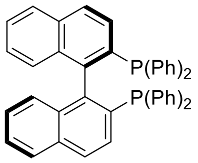
L7

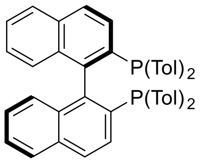
L8

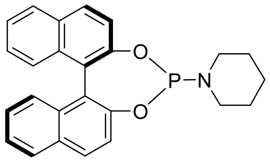
L9

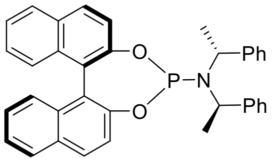
L10

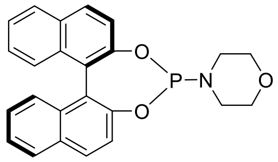
L11

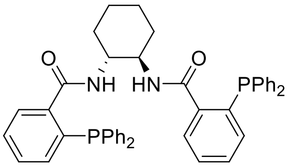
L12

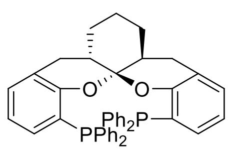
L13

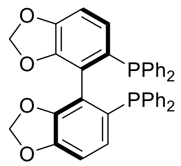
L14

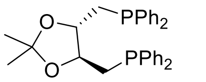
L15

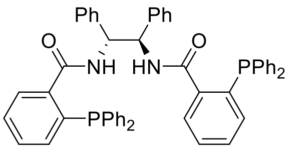
L16

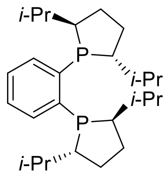
L17

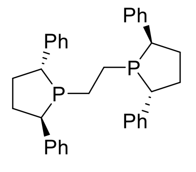
L18

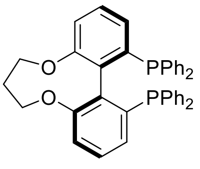
L19

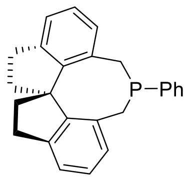
L20

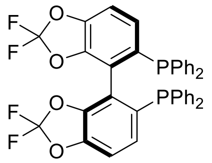
L21

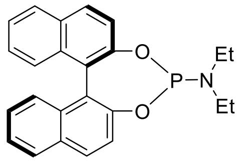
L22

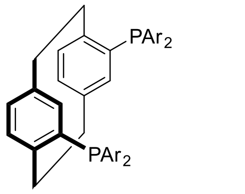
L23, Ar = Ph
L24, Ar = 3,5-(Me)$_2$-C$_6$H$_3$
L25, Ar = 4-OMe-C$_6$H$_4$

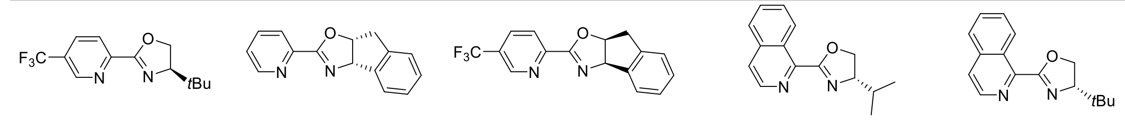

L26 L27 L28 L29 L30

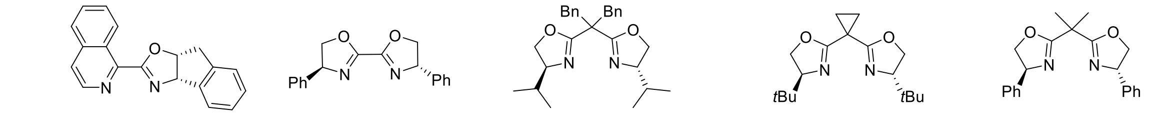

L31 L32 L33 L34 L35

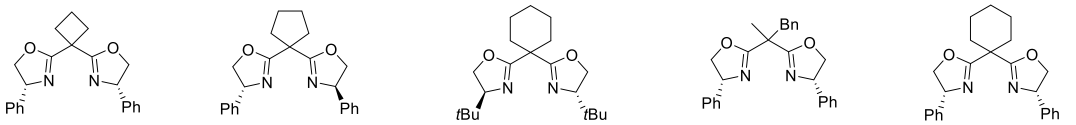

L36 L37 L38 L39 L40

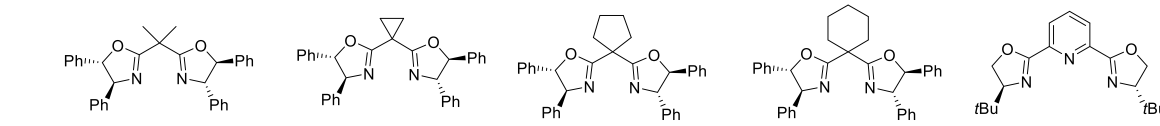

L41 L42 L43 L44 L45

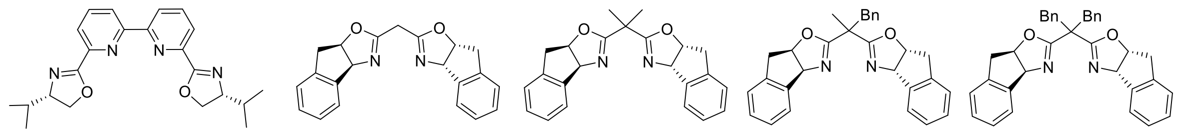

L46 L47 L48 L49 L50

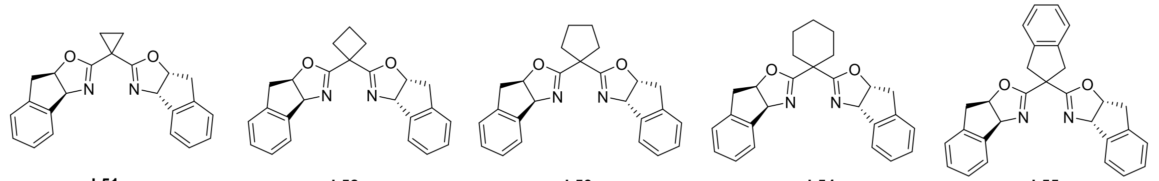

L51 L52 L53 L54 L55

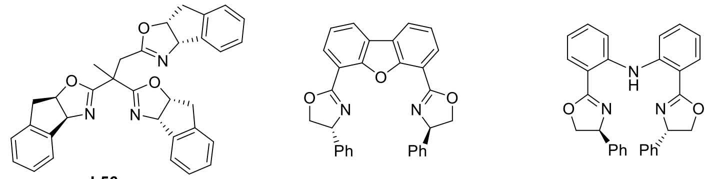

L56 L57 L58

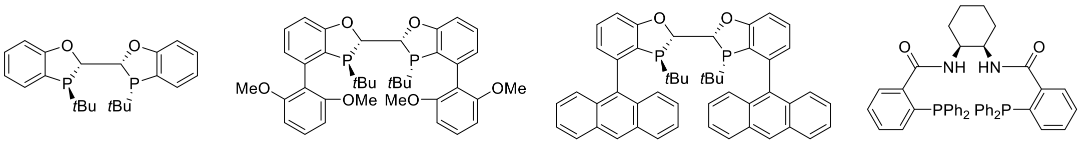

L59 L60 L61 L62

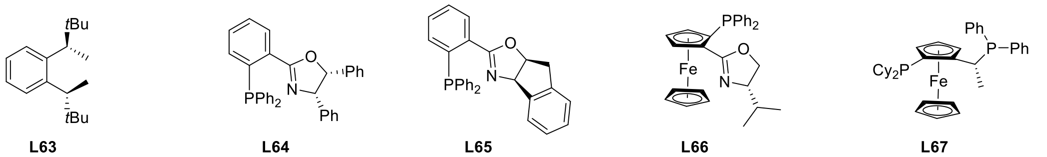

L63 L64 L65 L66 L67

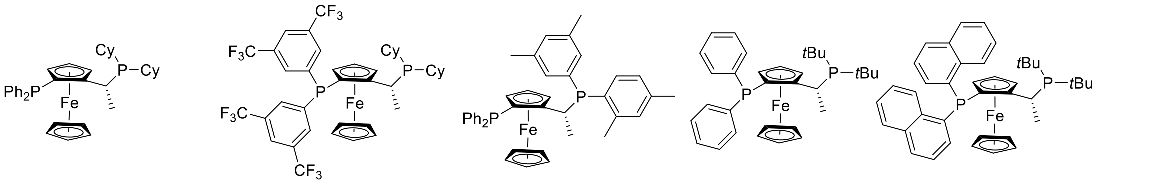

L68 L69 L70 L71 L72

11. X-ray crystal structure of compound 4ah

Crystal data for **4ah**: $C_{37}H_{33}N$, M = 491.64, a = 35.402(5) Å, b = 9.1778(13) Å, c = 17.961(3) Å, α = 90°, β = 110.844(6)°, γ = 90°, V = 5453.8(14) Å3, T = 100.(2) K, space group C12/c1, Z = 8, μ(Cu Kα) = 0.516 mm-1, 25990 reflections measured, 5178 independent reflections (Rint = 0.0857). The final R1 values were 0.0598 (I > 2σ(I)). The final wR(F2) values were 0.1647 (I > 2σ(I)). The final R1 values were 0.0730 (all data). The final wR(F2) values were 0.1790 (all data). The goodness of fit on F2 was 1.082.

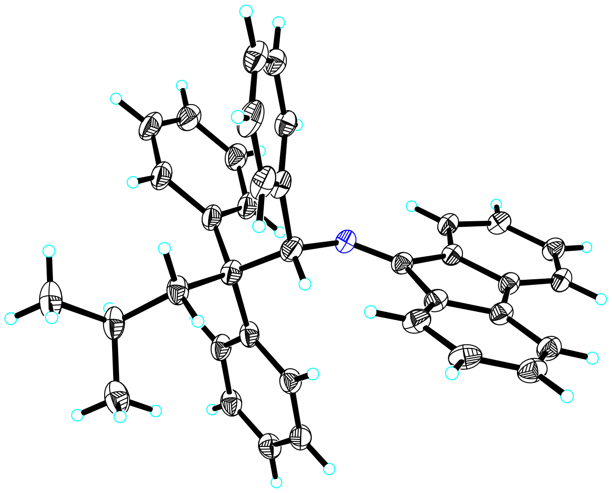

Figure S1. Crystal structure of **4ah** (**CCDC 2100377**)

Crystal data and structure refinement for 4ah.

Identification code	**4ah**	
Empirical formula	$C_{37}H_{33}N$	
Formula weight	491.64	
Temperature	100(2) K	
Wavelength	1.54178 Å	
Crystal system	Monoclinic	
Space group	C 1 2/c 1	
Unit cell dimensions	a = 35.402(5) Å	α= 90 °.
	b = 9.1778(13) Å	β= 110.844(6) °.
	c = 17.961(3) Å	γ = 90 °.
Volume	5453.8(14) $Å^3$	
Z	8	
Density (calculated)	1.198 Mg/m^3	
Absorption coefficient	0.516 mm^{-1}	
F(000)	2096	
Crystal size	0.330 x 0.320 x 0.140 mm^3	

Theta range for data collection	2.67 to 70.42 °.
Index ranges	-40<=h<=42, -11<=k<=11, -21<=l<=18
Reflections collected	25990
Independent reflections	5178 [R(int) = 0.0857]
Completeness to theta = 70.42 °	99.2 %
Absorption correction	Semi-empirical from equivalents
Max. and min. transmission	0.93 and 0.49
Refinement method	Full-matrix least-squares on F^2
Data / restraints / parameters	5178 / 399 / 448
Goodness-of-fit on F^2	1.082
Final R indices [I>2sigma(I)]	R1 = 0.0598, wR2 = 0.1647
R indices (all data)	R1 = 0.0730, wR2 = 0.1790
Largest diff. peak and hole	0.237 and -0.219 e.Å^{-3}

12. Supplementary references

[1] J. Liu, C.-G. Cao, H.-B. Sun, X. Zhang and D. Niu, *J. Am. Chem. Soc.* **2016**, *138*, 13103−13106.

[2] S. Duan, G. Deng, Y. Zi, X. Wu, X. Tian, Z. Liu, M. Li, H. Zhang, X. Yang and P. J. Walsh, *Chem. Sci.* **2021**, *12*, 6406–6412.

[3] Y. Zhu and S. L. Buchwald, *J. Am. Chem. Soc.* **2014**, *136*, 4500−4503.

[4] J. Cornella, J. T. Edward, T. Qin, S. Kawamura, J. Wang, C.-M. Pan, R. Gianatassio, M. Schmidt, M. D. Eastgate and P. S. Baran, *J. Am. Chem. Soc.* **2016**, *138*, 2174−2177.

[5] T. Qin, J. Cornella, C. Li, L. R. Malins, J. T. Edwards, S. Kawamura, B. D. Maxwell, M. D. Eastgate and P. S. Baran, *Science* **2016**, *352*, 797−800.

[6] Y. Jin, H. Yang and C. Wang, *Org. Lett.* **2019**, *21*, 7602−7608.

[7] Y. Gao, D. E. Hill, W. Hao, B. J. McNicholas, J. C. Vantourout, R. G. Hadt, S. E. Reisman, D. G. Blackmond and P. S. Baran, *J. Am. Chem. Soc.* **2021**, *143*, 9478−9488.

[8] P. P. Chandrachud, L. Wojtas and J. M. Lopchuk, *J. Am. Chem. Soc.* **2020**, *142*, 21743−21750.

[9] L. Yu, M. L. Tang, C. M. Si, Z. Meng, Y. Liang, J. Han and X. Sun, *Org. Lett.* **2018**, *20*, 4579−4583.

[10] W. Zhao, R. P. Wurz, J. C. Peters and G. C. Fu, *J. Am. Chem. Soc.* **2017**, *139*, 12153−12156.

[11] H.-M. Huang, M. Koy, E. Serrano, P. M. Pflüger, J. L. Schwarz and F. Glorius, *Nat. Catal.* **2020**, *3*, 393−400.

[12] R. Kobayashi, S. Shibutani, K. Nagao, Z. Ikeda, J. Wang, I. Ibáñez, M. Reynolds, Y. Sasaki and H. Ohmiya, *Org. Lett.* **2021**, *23*, 5415−5419.

[13] Y. Chen, J. wang and Y. Lu, *Chem. Sci.* **2021**, *12*, 11316–11321.

[14] V. Soulard, G. Villa, D. P Vollmar and P. Renaud, *J. Am. Chem. Soc.* **2018**, *140*, 155−158.

[15] B. Yang and Z. Lu, *Chem. Commun.* **2017**, *53*, 12634−12637.

13. NMR Spectra of the products

Figure S1. ^{1}H NMR spectra (400 MHz, Chloroform-*d*) of (*E*)-1-Cyclobutyl-*N*-(9*H*-fluoren-9-yl)methanimine (1p).

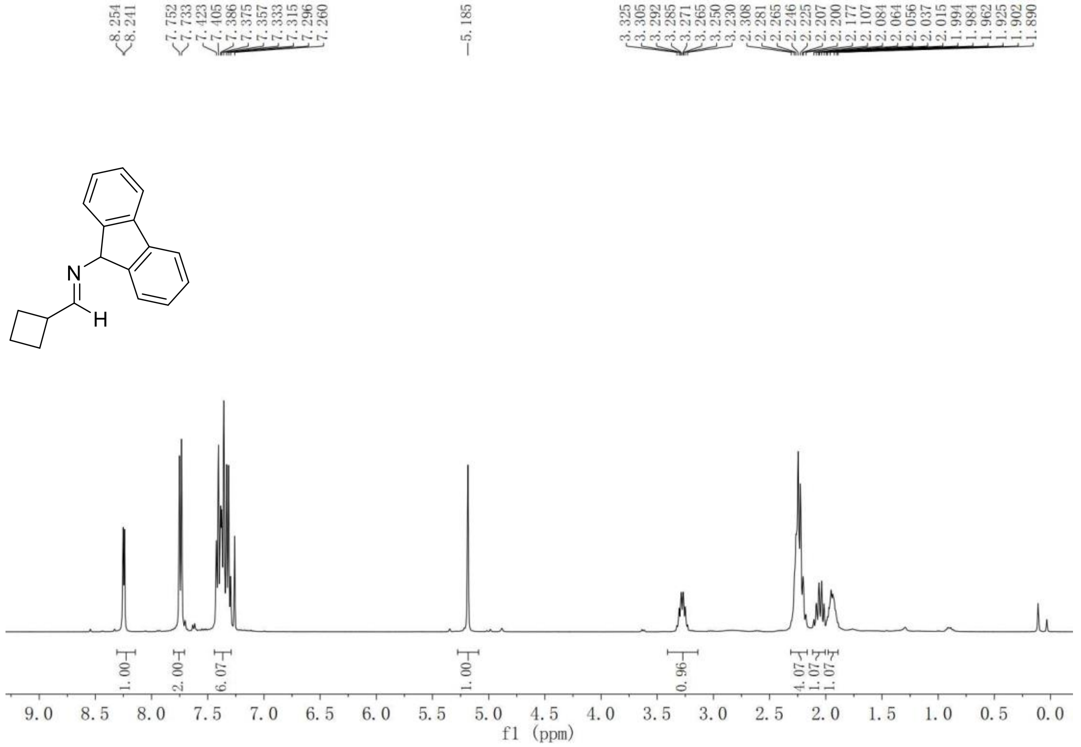

Figure S2. ^{13}C{^{1}H} NMR spectra (100 MHz, Chloroform-*d*) of (*E*)-1-Cyclobutyl-*N*-(9*H*-fluoren-9-yl)methanimine (1p).

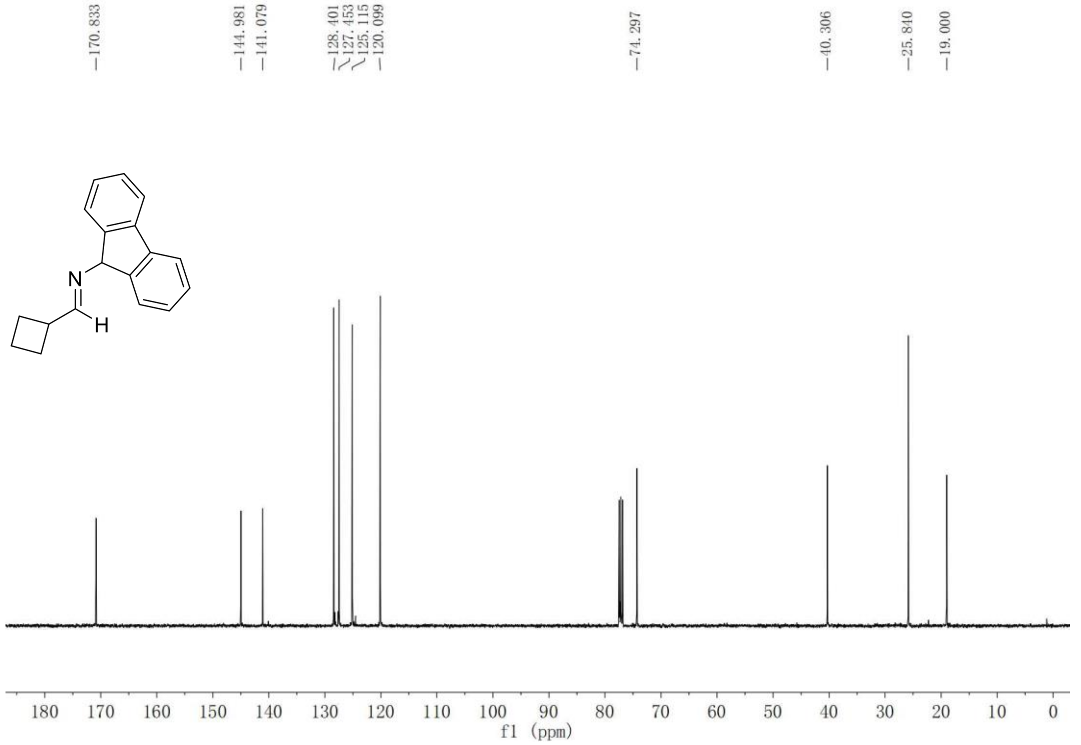

Figure S3. **^{1}H NMR spectra (400 MHz, Chloroform-*d*) of (*E*)-1-Cyclopentyl-*N*-(9*H*-fluoren-9-yl)methanimine (1q).**

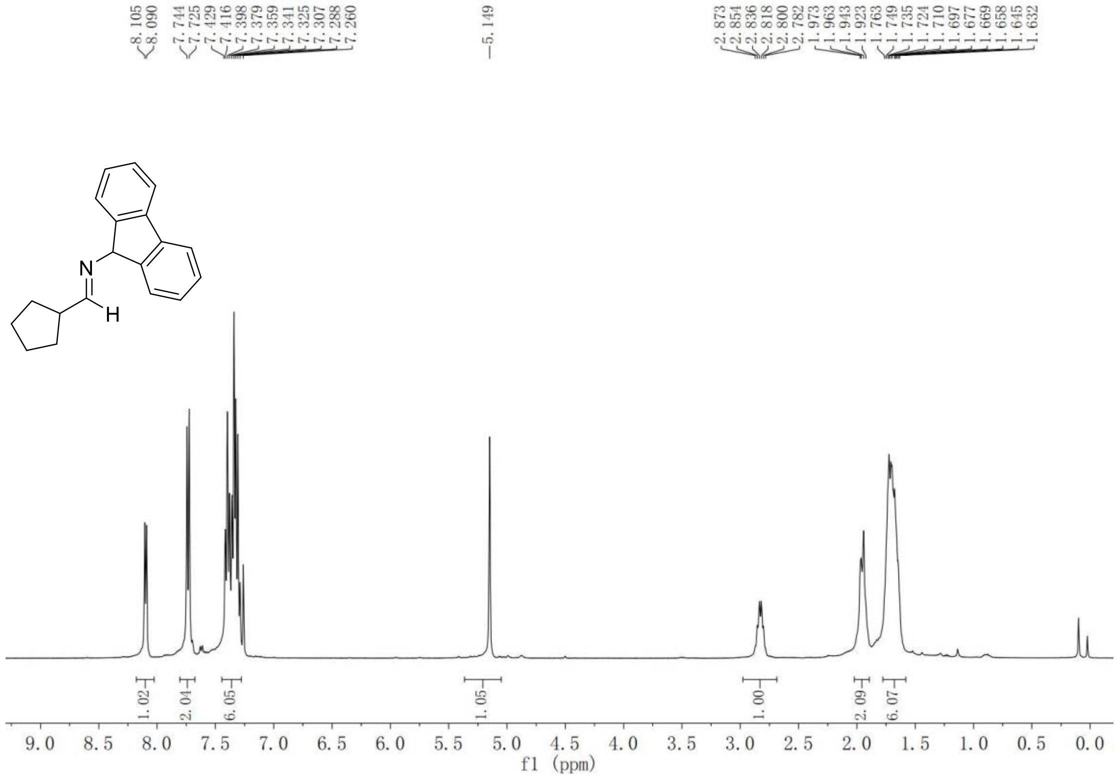

Figure S4. **^{13}C{^{1}H} NMR spectra (100 MHz, Chloroform-*d*) of (*E*)-1-Cyclopentyl-*N*-(9*H*-fluoren-9-yl)methanimine (1q).**

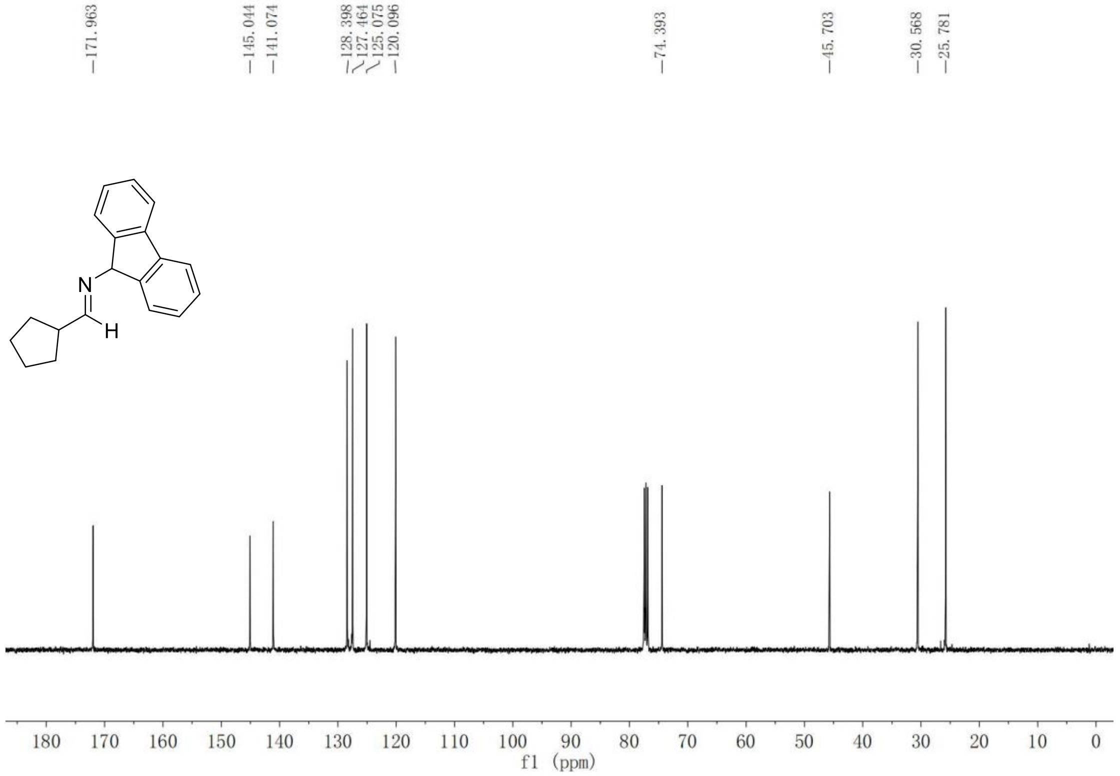

Figure S5. ^{1}**H NMR spectra (400 MHz, Chloroform-*d*) of (*E*)-1-Cyclohexyl-*N*-(9*H*-fluoren-9-yl)methanimine (1r).**

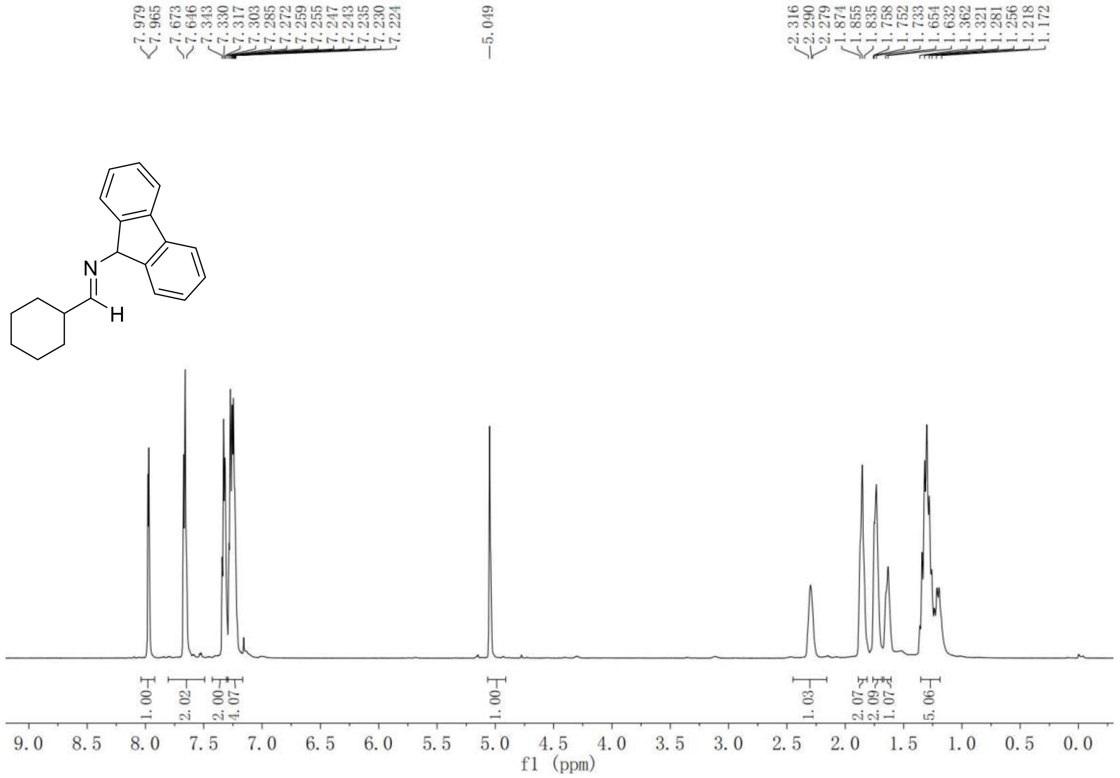

Figure S6. ^{13}**C{**^{1}**H} NMR spectra (100 MHz, Chloroform-*d*) of (*E*)-1-Cyclohexyl-*N*-(9*H*-fluoren-9-yl)methanimine (1r).**

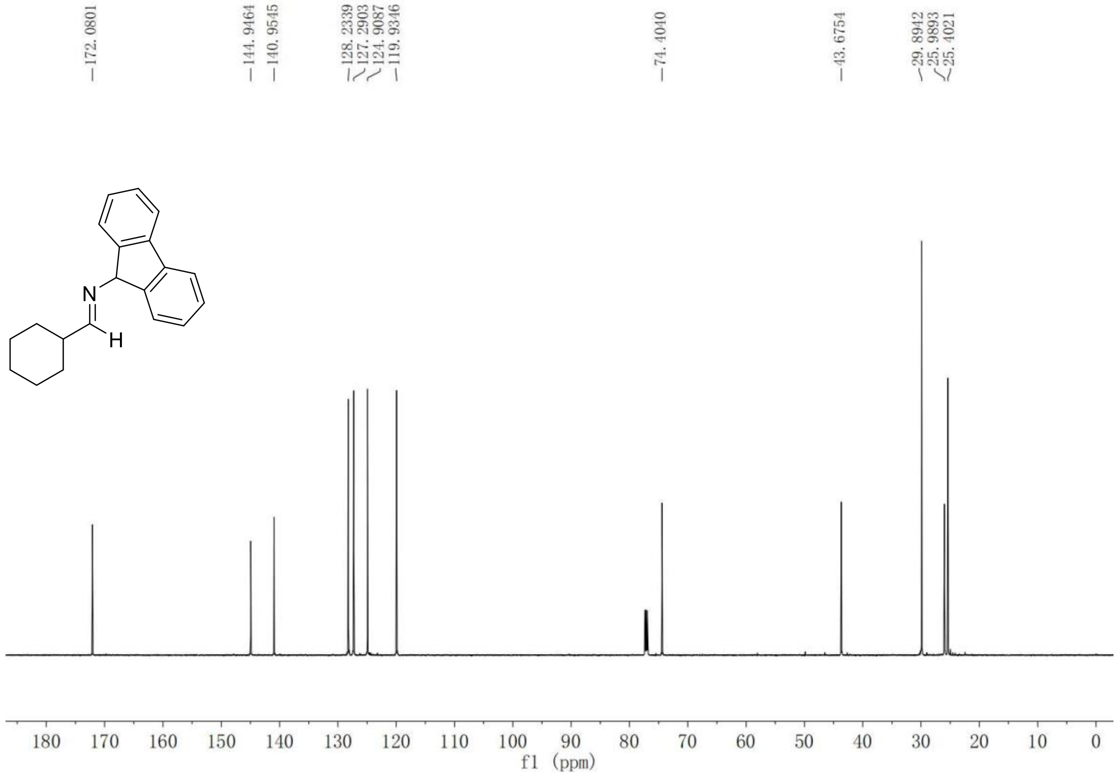

Figure S7. ^{1}**H NMR spectra (400 MHz, Chloroform-*d*) of *N*-(3-((3r,5r,7r)-Adamantan-1-yl)-1,2,2-triphenylpropyl)-9*H*-fluoren-9-imine (4aa).**

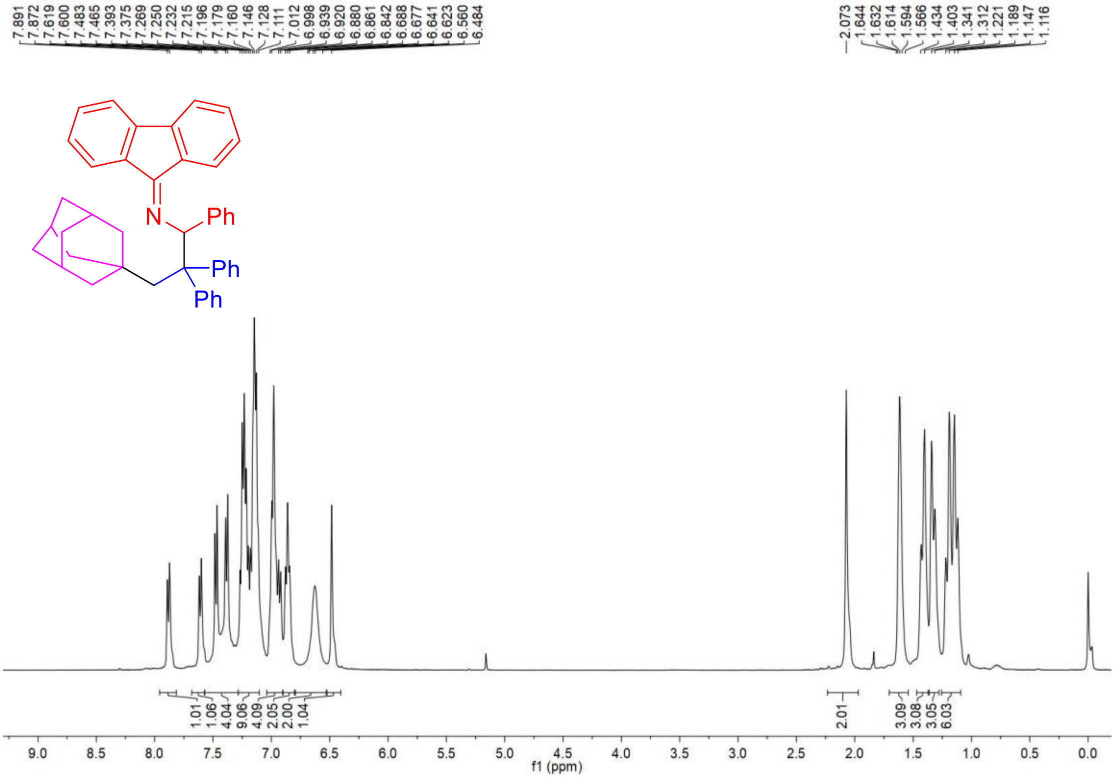

Figure S8. ^{13}**C{**^{1}**H} NMR spectra (100 MHz, Chloroform-*d*) of *N*-(3-((3r,5r,7r)-Adamantan-1-yl)-1,2,2-triphenylpropyl)-9*H*-fluoren-9-imine (4aa).**

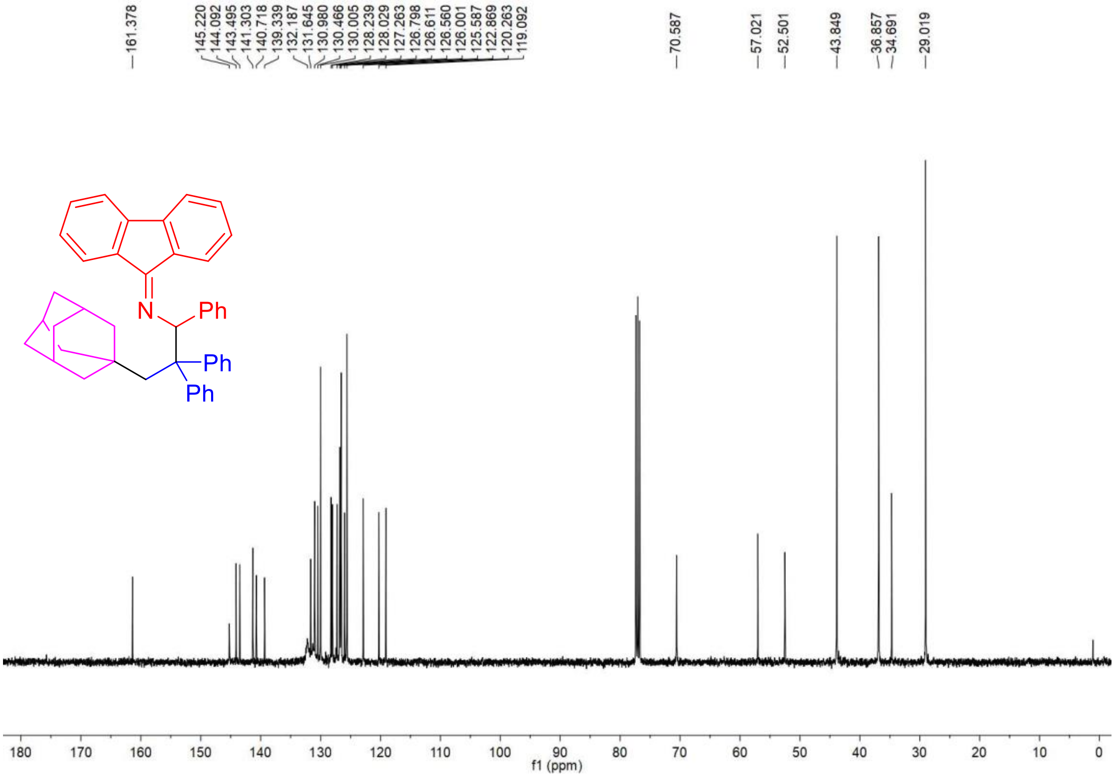

Figure S9. **^{1}H NMR spectra (400 MHz, Chloroform-*d*) of *N*-(5,5-Dimethyl-1,2,2-triphenylhexyl)-9*H*-fluoren-9-imine (4ab).**

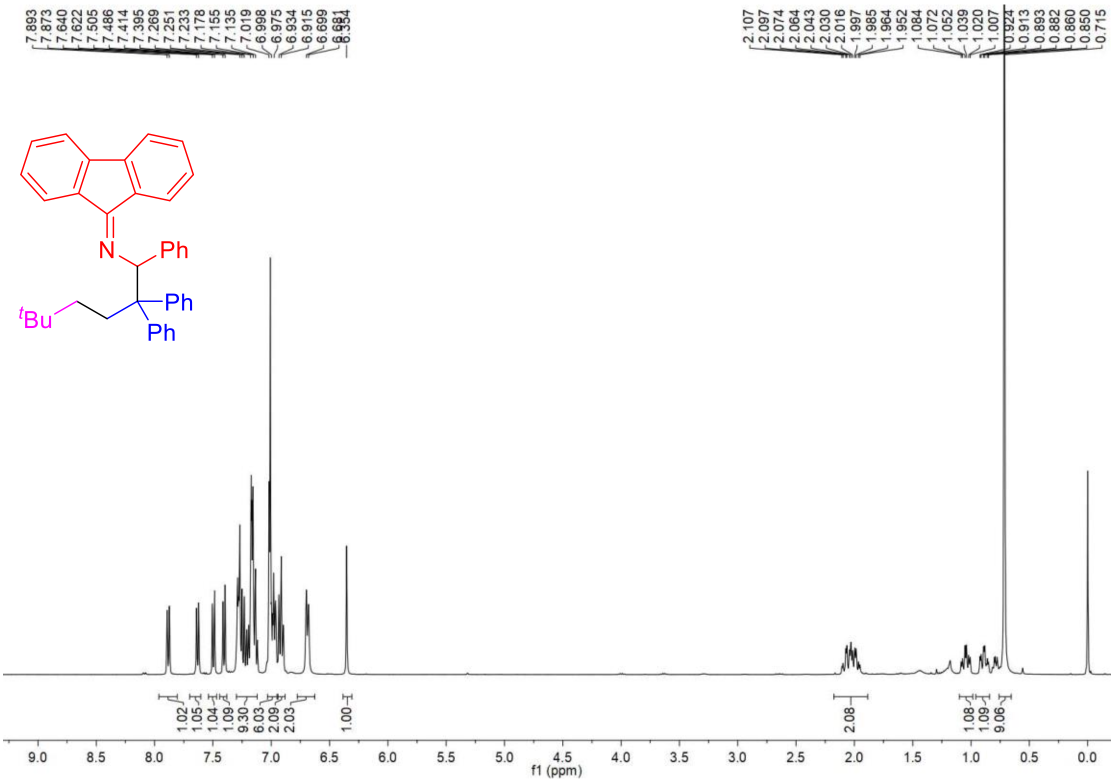

Figure S10. **^{13}C{^{1}H} NMR spectra (100 MHz, Chloroform-*d*) of *N*-(5,5-Dimethyl-1,2,2-triphenylhexyl)-9*H*-fluoren-9-imine (4ab).**

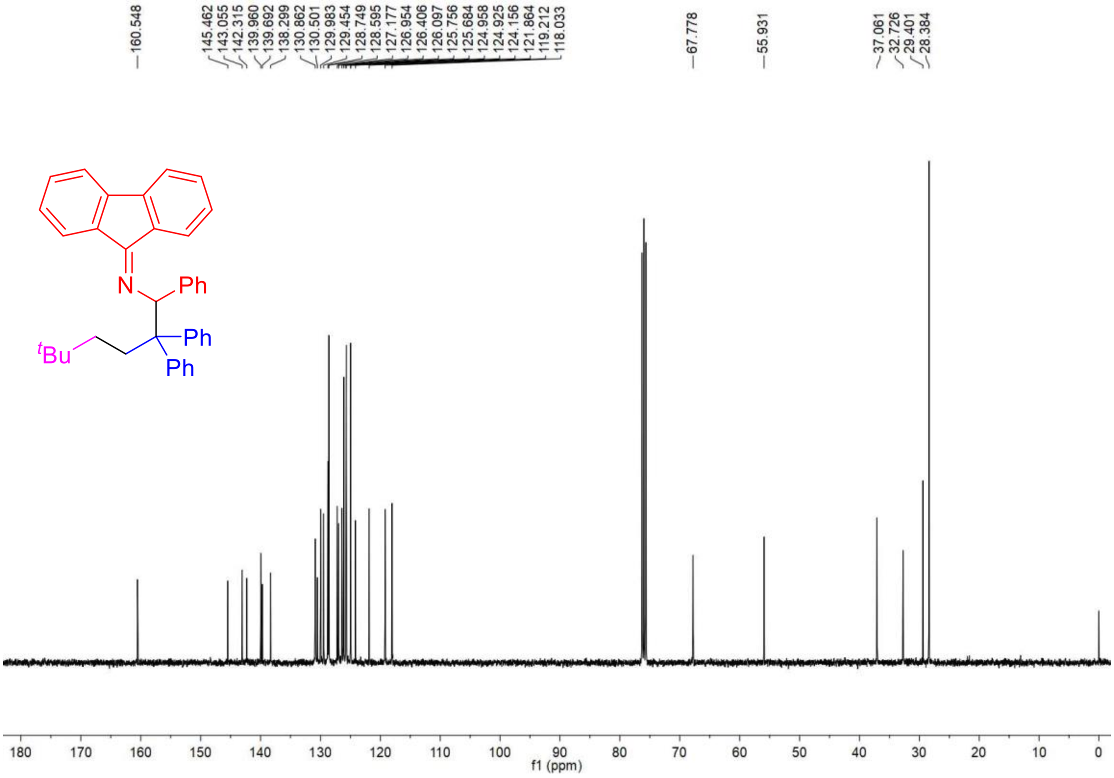

Figure S11. ^{1}H NMR spectra (400 MHz, Chloroform-*d*) of *N*-(4-Cyclobutyl-1,2,2-triphenylbutyl)-9*H*-fluoren-9-imine (4ac).

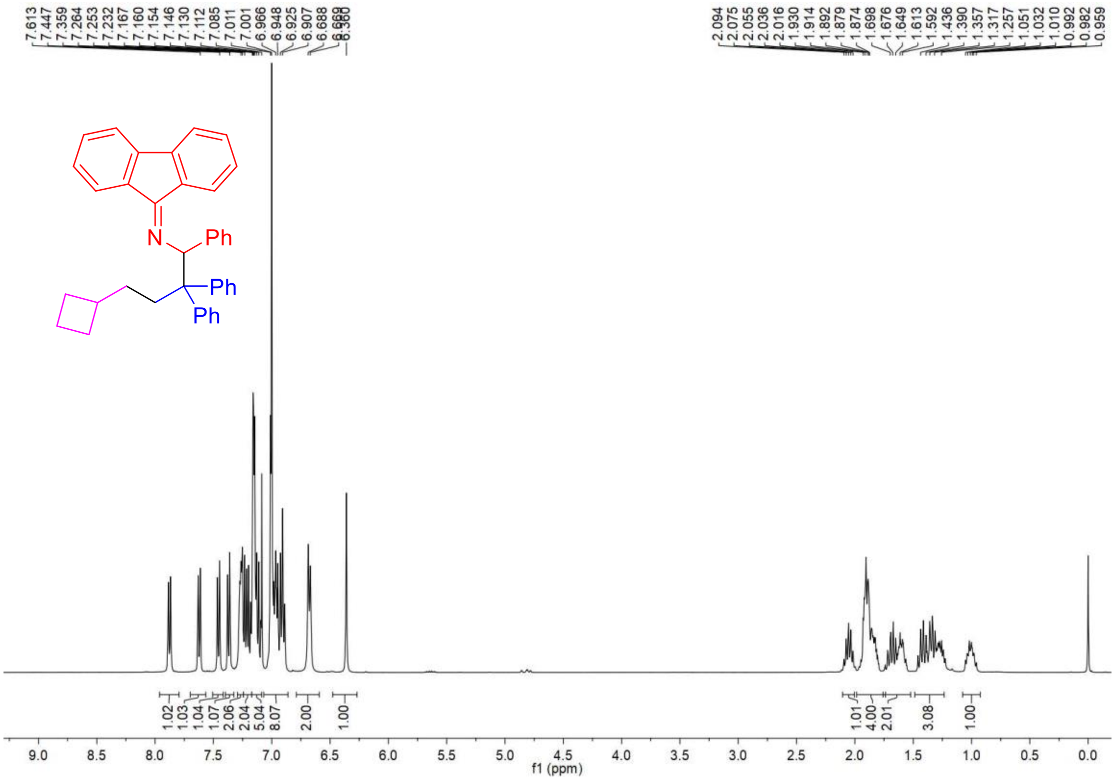

Figure S12. ^{13}C{^{1}H} NMR spectra (100 MHz, Chloroform-*d*) of *N*-(4-Cyclobutyl-1,2,2-triphenylbutyl)-9*H*-fluoren-9-imine (4ac).

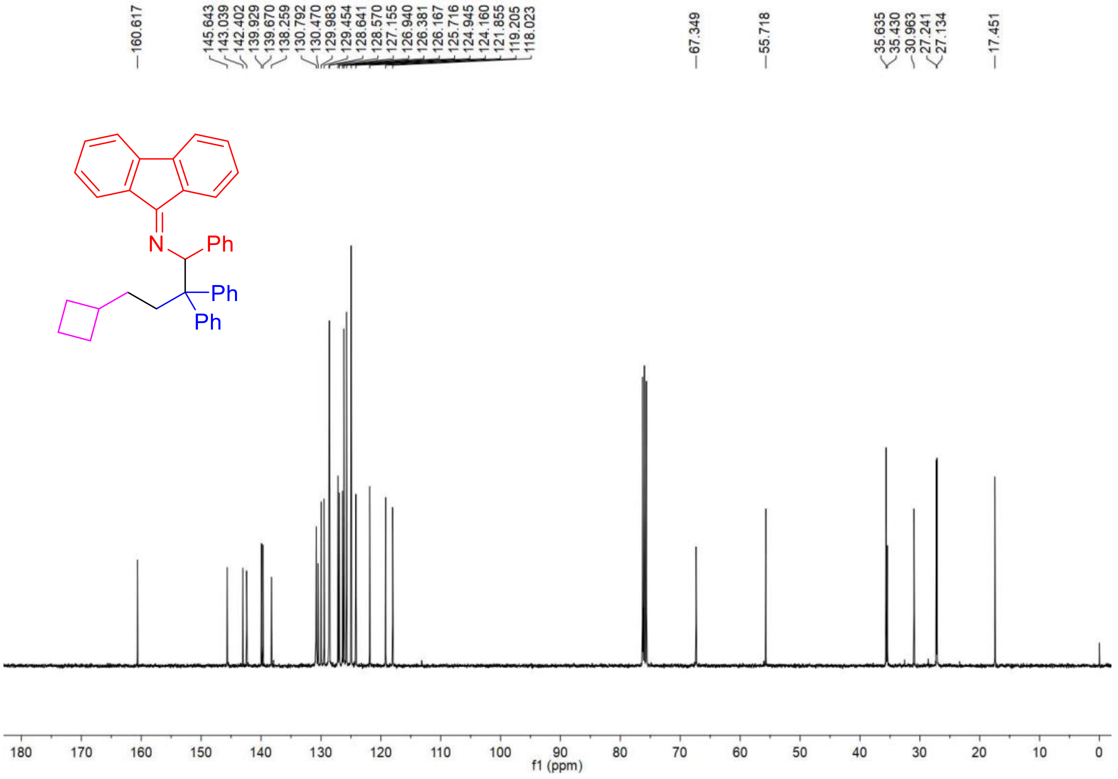

Figure S13. **^{1}H NMR spectra (400 MHz, Chloroform-*d*) of *N*-(4-Cyclopentyl-1,2,2-triphenylbutyl)-9*H*-fluoren-9-imine (4ad).**

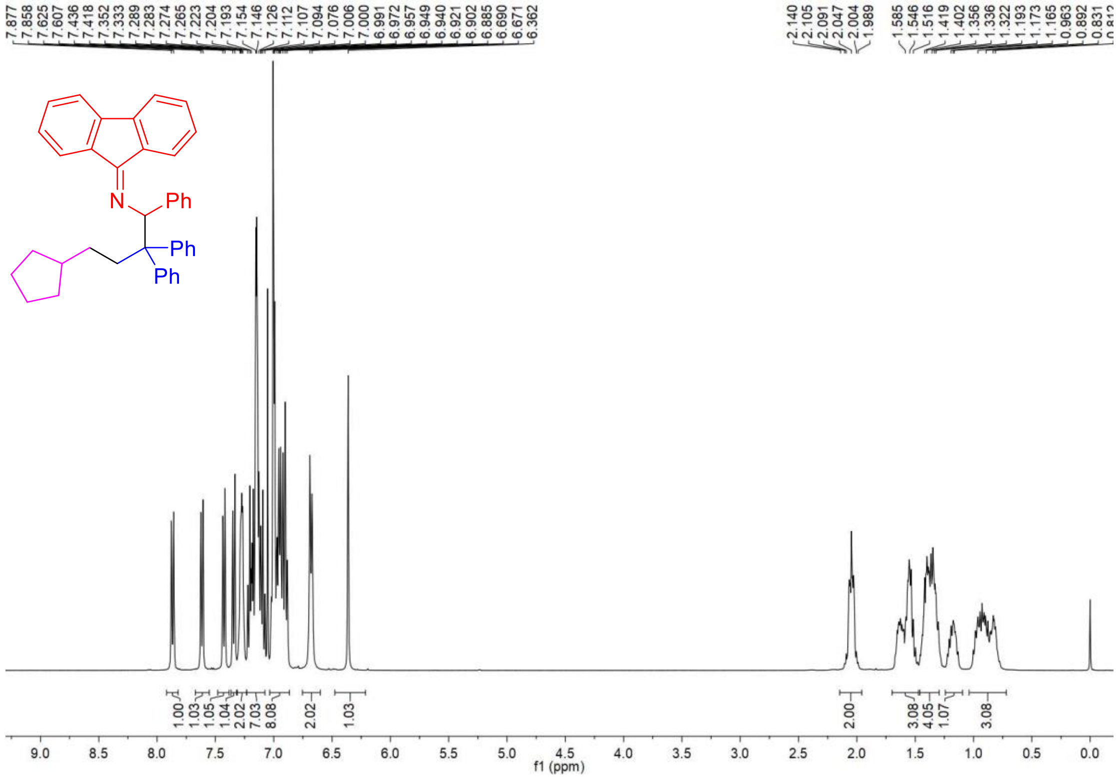

Figure S14. **^{13}C{^{1}H} NMR spectra (100 MHz, Chloroform-*d*) of *N*-(4-Cyclopentyl-1,2,2-triphenylbutyl)-9*H*-fluoren-9-imine (4ad).**

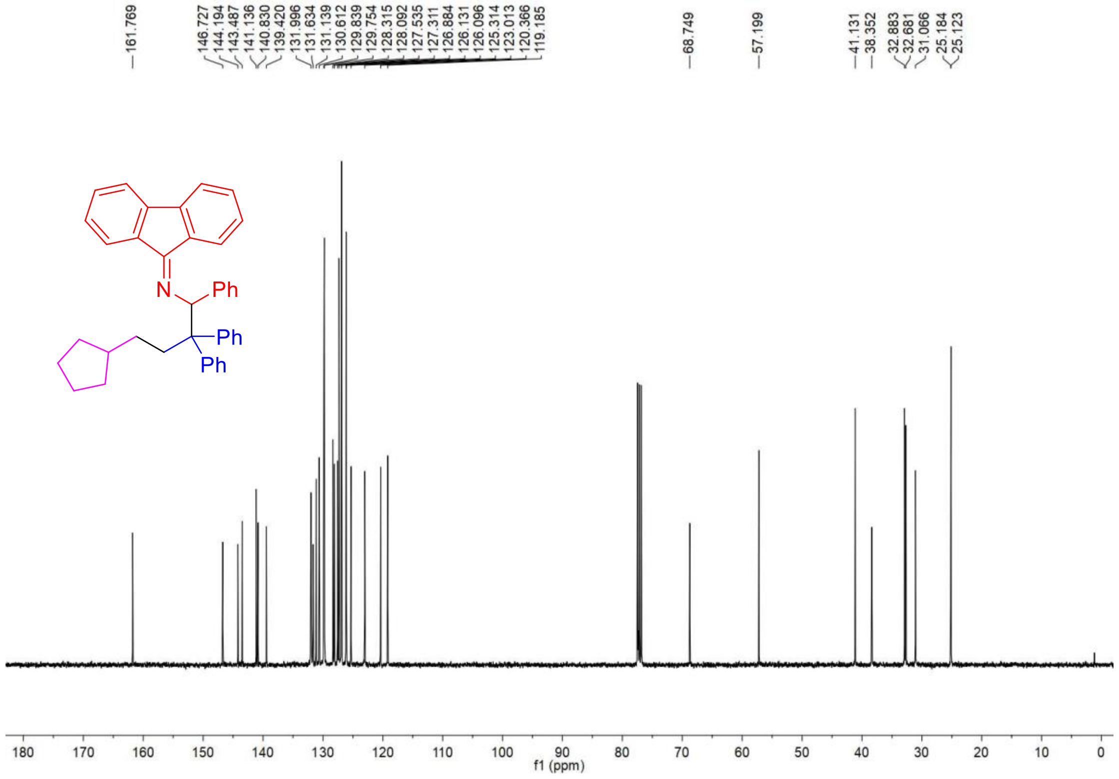

Figure S15. ^{1}H NMR spectra (400 MHz, Chloroform-*d*) of *N*-(4-Cyclohexyl-1,2,2-triphenylbutyl)-9*H*-fluoren-9-imine (4ae).

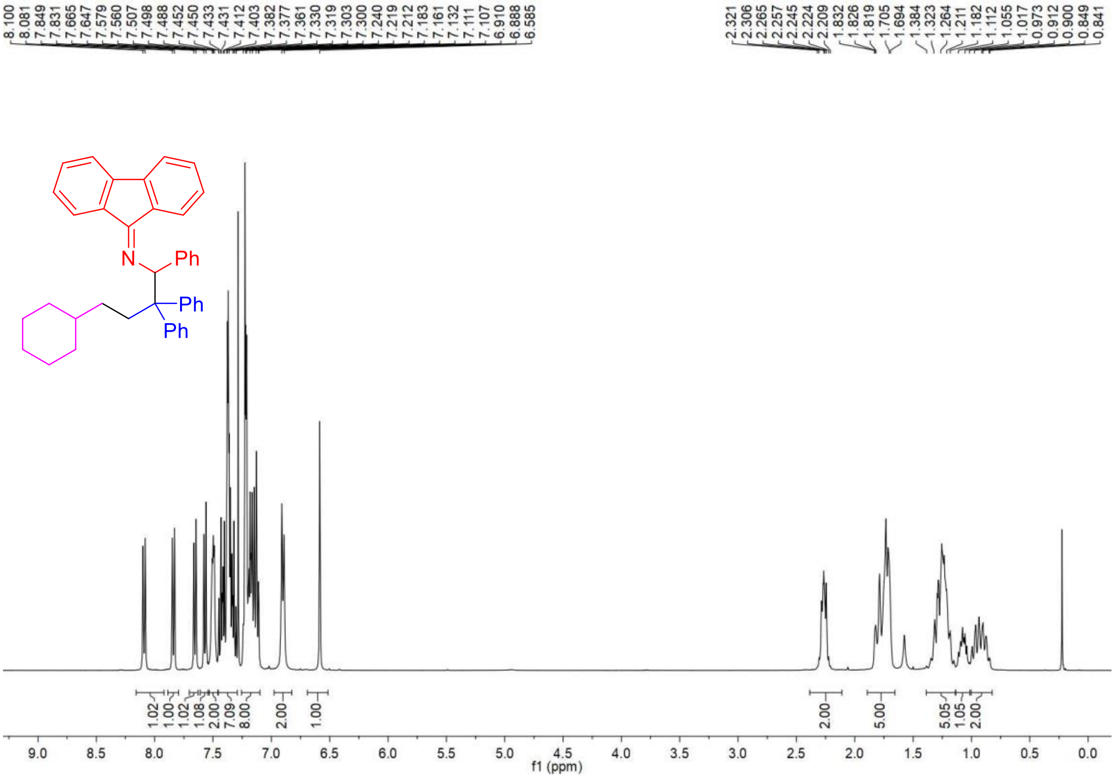

Figure S16. ^{13}C{^{1}H} NMR spectra (100 MHz, Chloroform-*d*) of *N*-(4-Cyclohexyl-1,2,2-triphenylbutyl)-9*H*-fluoren-9-imine (4ae).

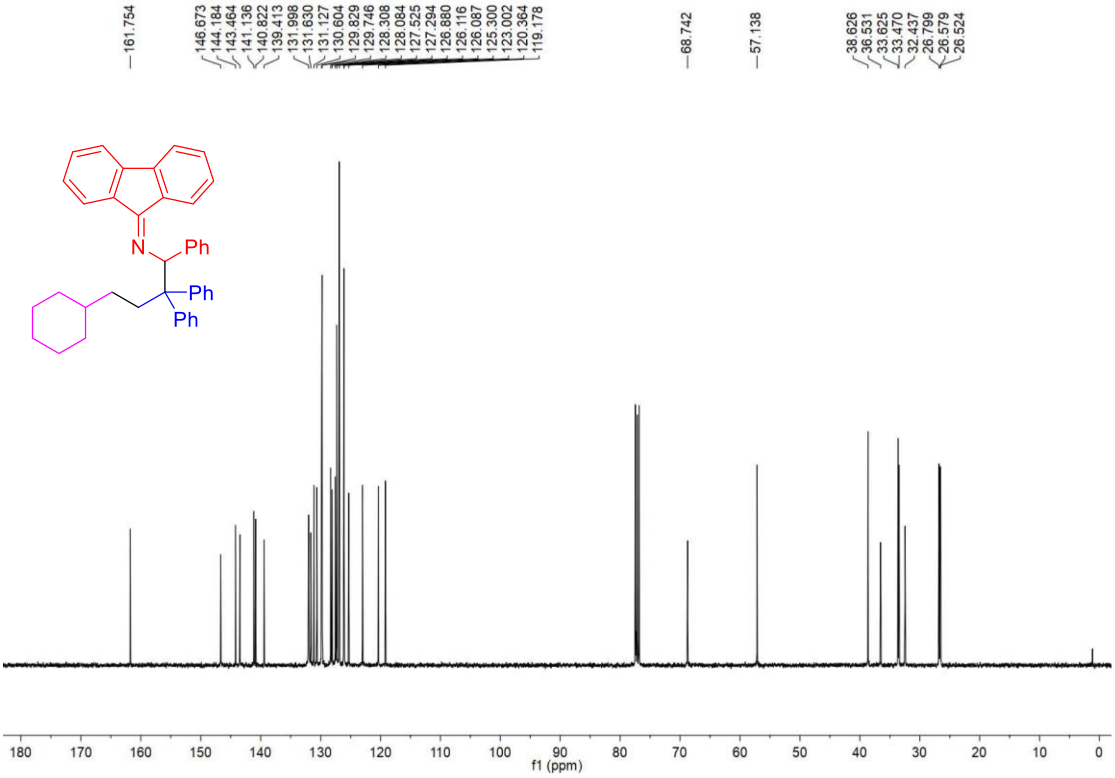

Figure S17. **^{1}H NMR spectra (400 MHz, Chloroform-*d*) of *tert*-Butyl 4-(4-((9*H*-fluoren-9-ylidene)amino)-3,3,4-triphenylbutyl)piperidine -1-carboxylate (4af).**

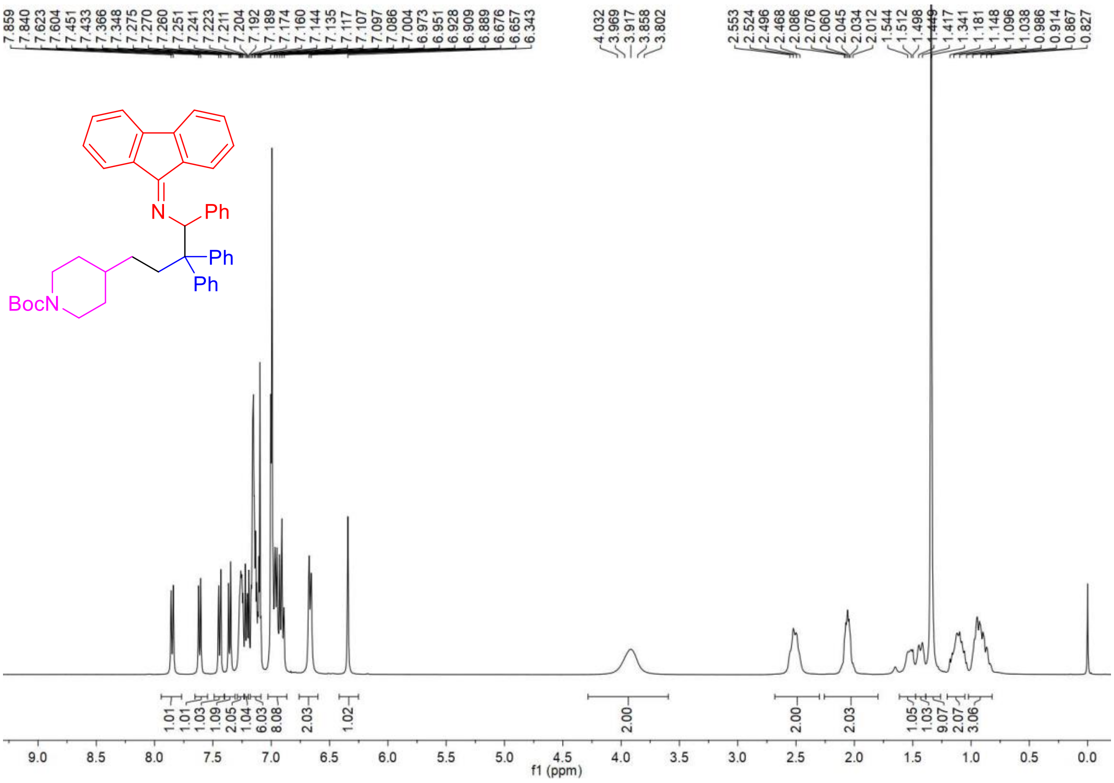

Figure S18. **^{13}C{^{1}H} NMR spectra (100 MHz, Chloroform-*d*) of *tert*-Butyl 4-(4-((9*H*-fluoren-9-ylidene)amino)-3,3,4-triphenylbutyl)piperidine -1-carboxylate (4af).**

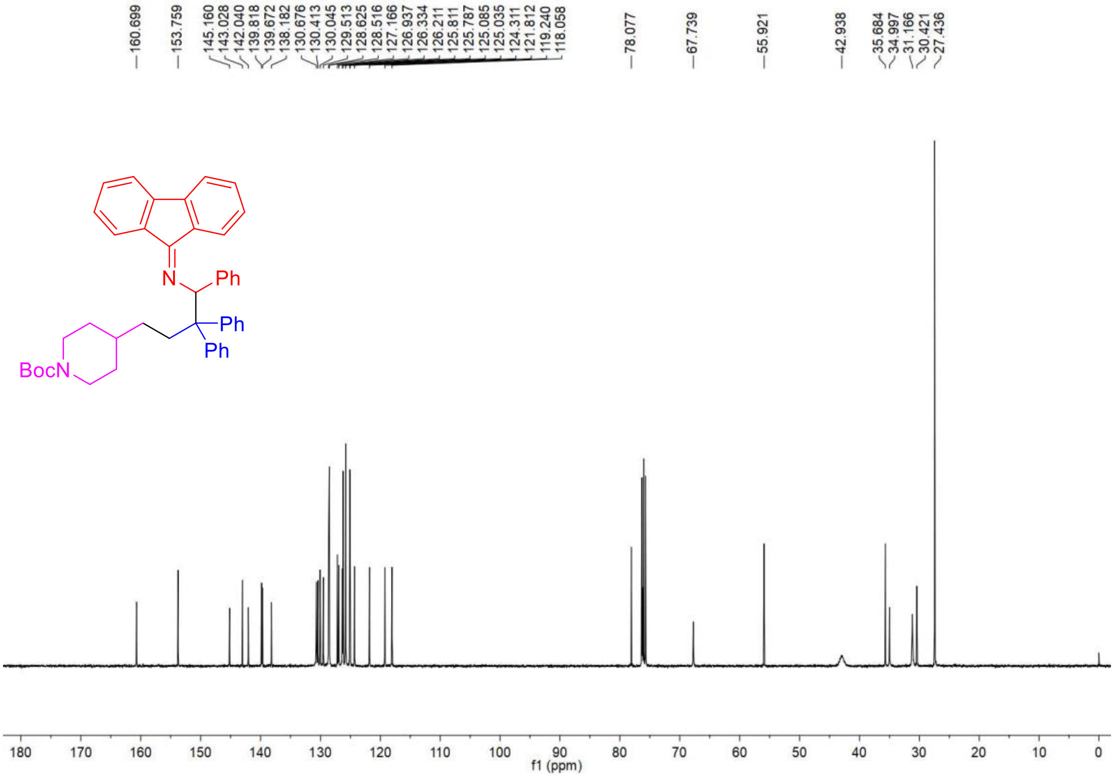

Figure S19. ^{1}**H NMR spectra (400 MHz, Chloroform-*d*) of *N*-(1,2,2-Triphenyl-4-(tetrahydro-2*H*-pyran-4-yl)butyl)-9*H*-fluoren-9-imine (4ag).**

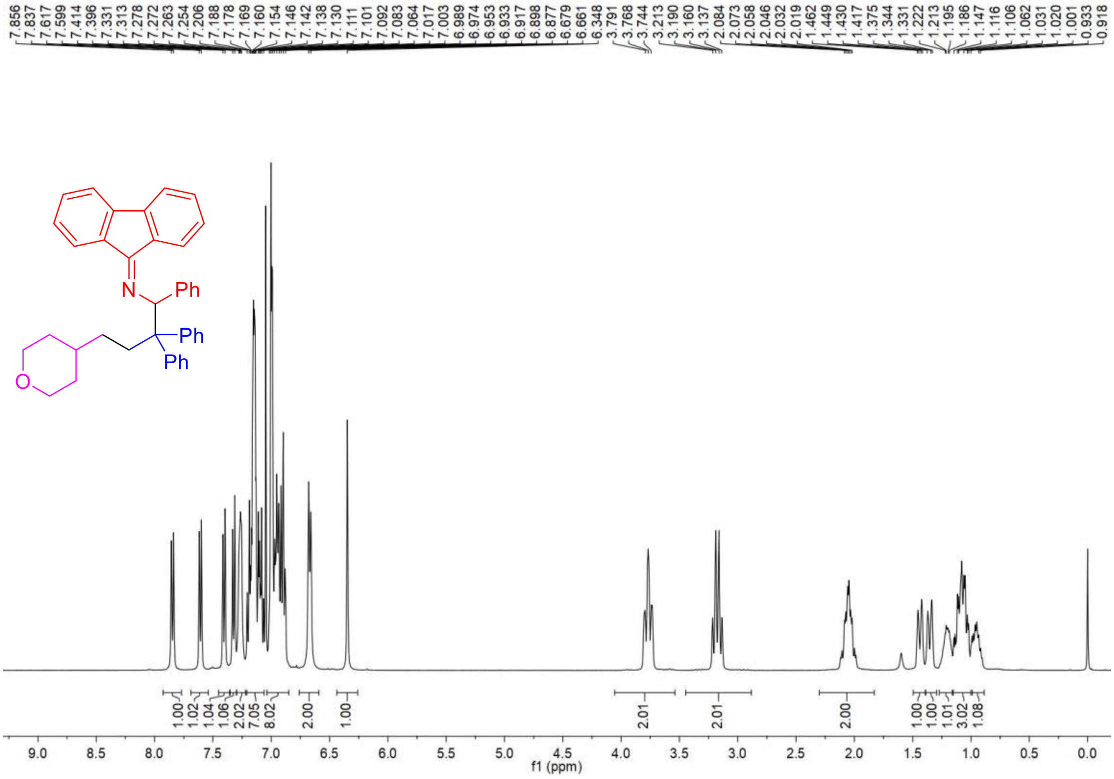

Figure S20. ^{13}**C{**^{1}**H} NMR spectra (100 MHz, Chloroform-*d*) of *N*-(1,2,2-Triphenyl-4-(tetrahydro-2*H*-pyran-4-yl)butyl)-9*H*-fluoren-9-imine (4ag).**

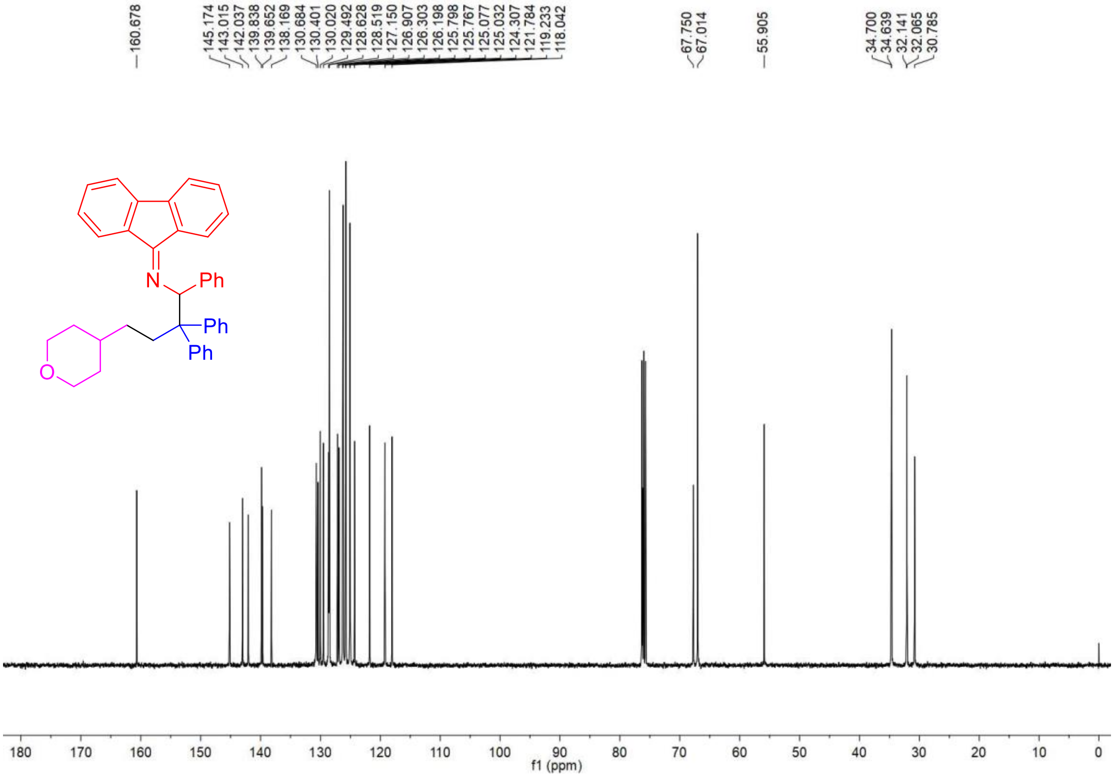

Figure S21. ^{1}**H NMR spectra (400 MHz, Chloroform-*d*) of *N*-(4-Methyl-1,2,2-triphenylpentyl)-9*H*-fluoren-9-imine (4ah).**

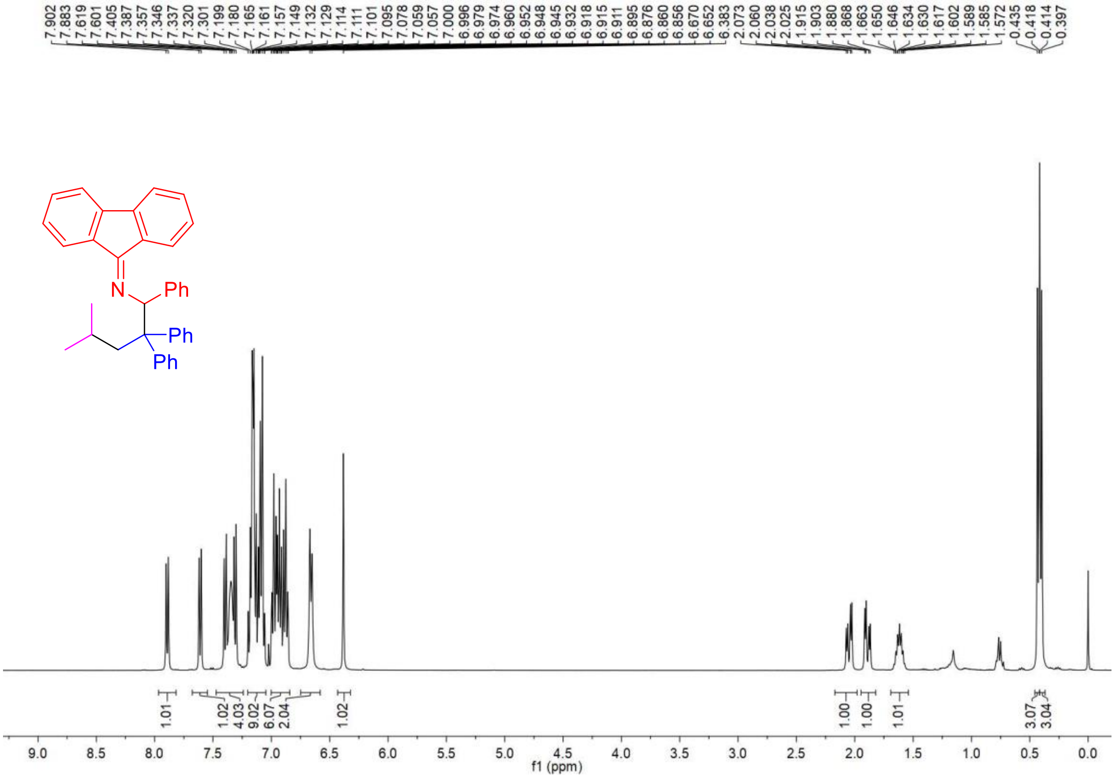

Figure S22. ^{13}**C{**^{1}**H} NMR spectra (100 MHz, Chloroform-*d*) of *N*-(4-Methyl-1,2,2-triphenylpentyl)-9*H*-fluoren-9-imine (4ah).**

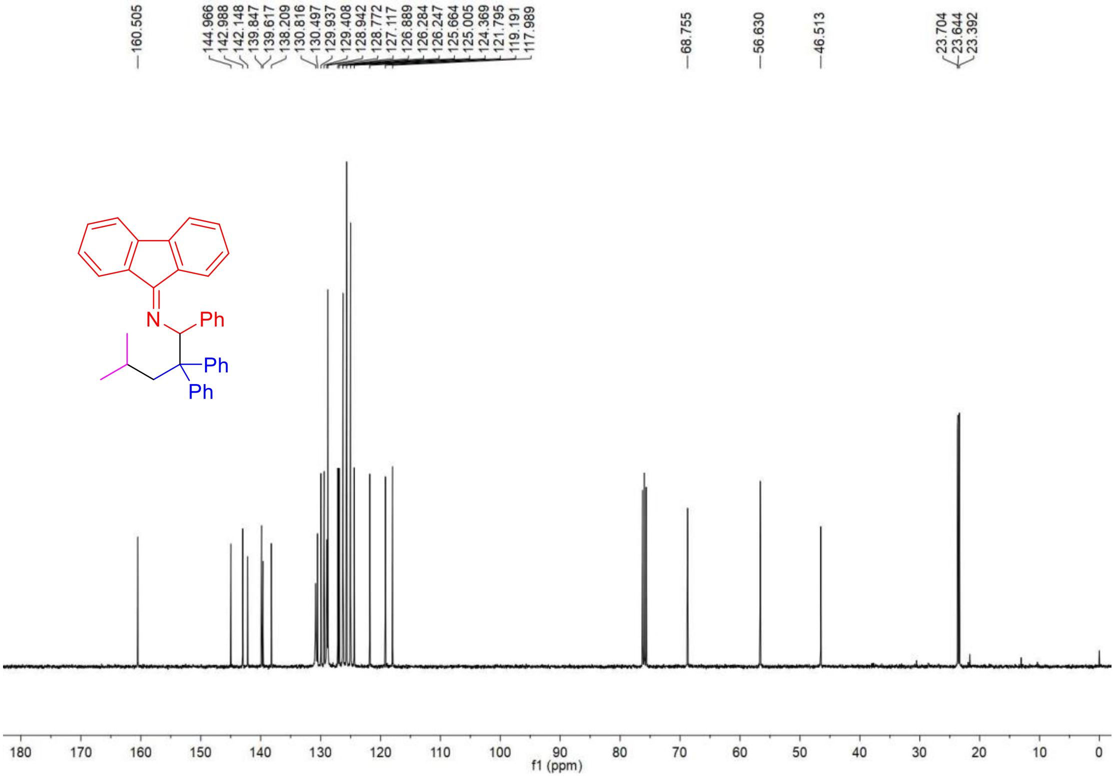

Figure S23. ^{1}**H NMR spectra (400 MHz, Chloroform-*d*) of *N*-(3-Cyclobutyl-1,2,2-triphenylpropyl)-9*H*-fluoren-9-imine (4ai).**

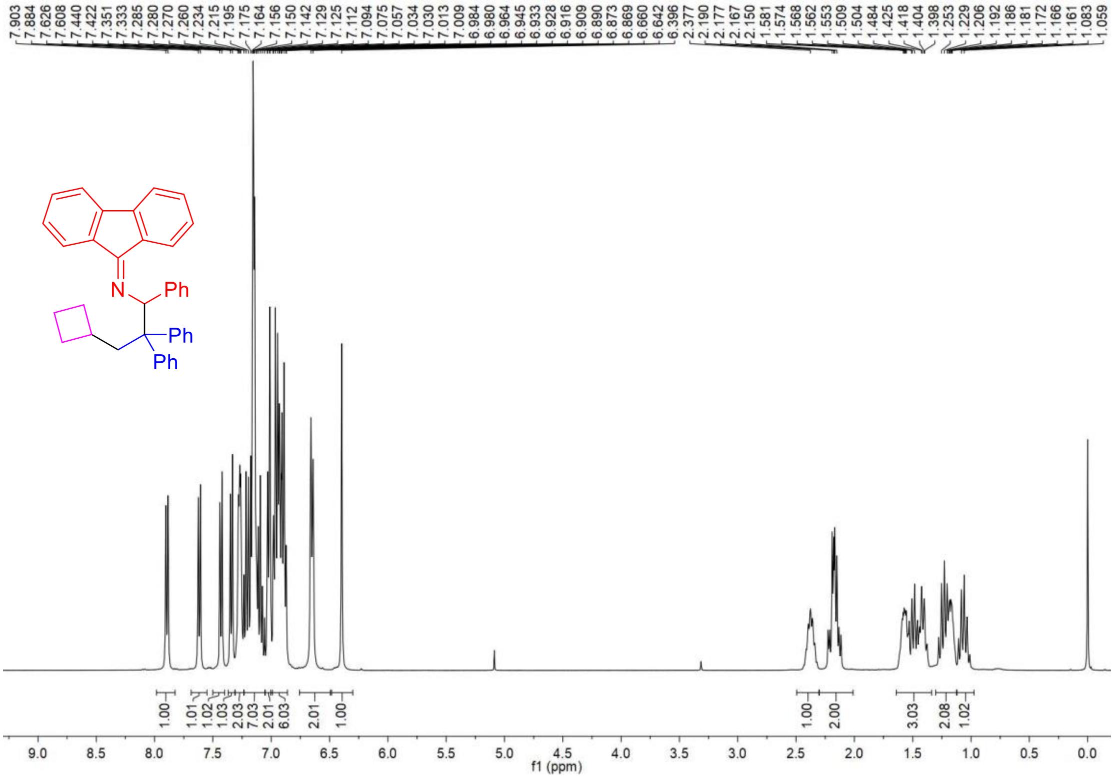

Figure S24. ^{13}**C{**^{1}**H} NMR spectra (100 MHz, Chloroform-*d*) of *N*-(3-Cyclobutyl-1,2,2-triphenylpropyl)-9*H*-fluoren-9-imine (4ai).**

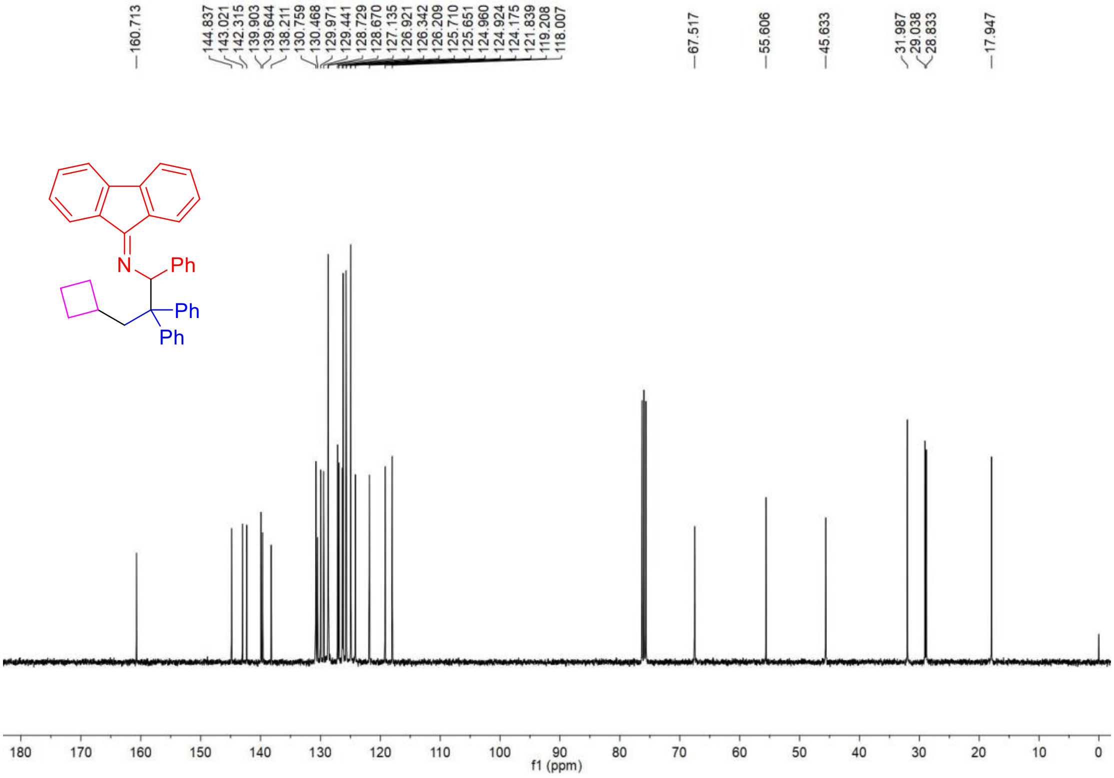

Figure S25. ^{1}H NMR spectra (400 MHz, Chloroform-*d*) of *N*-(3-Cyclopentyl-1,2,2-triphenylpropyl)-9*H*-fluoren-9-imine (4aj).

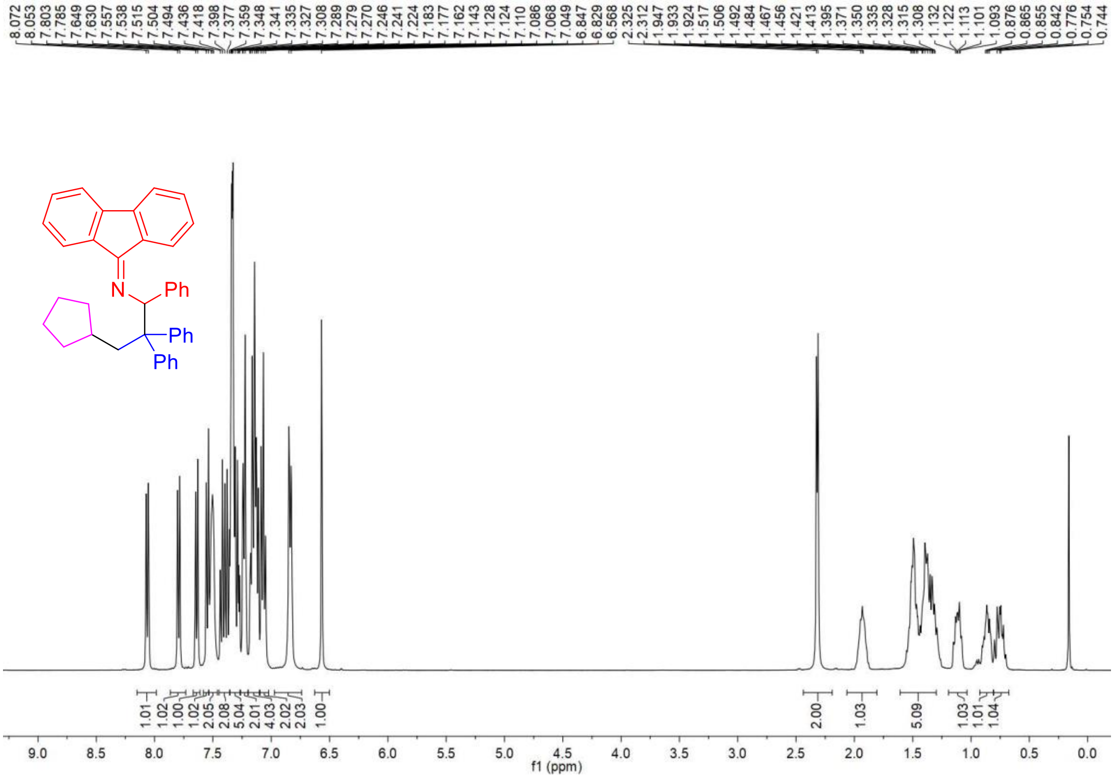

Figure S26. ^{13}C{^{1}H} NMR spectra (100 MHz, Chloroform-*d*) of *N*-(3-Cyclopentyl-1,2,2-triphenylpropyl)-9*H*-fluoren-9-imine (4aj).

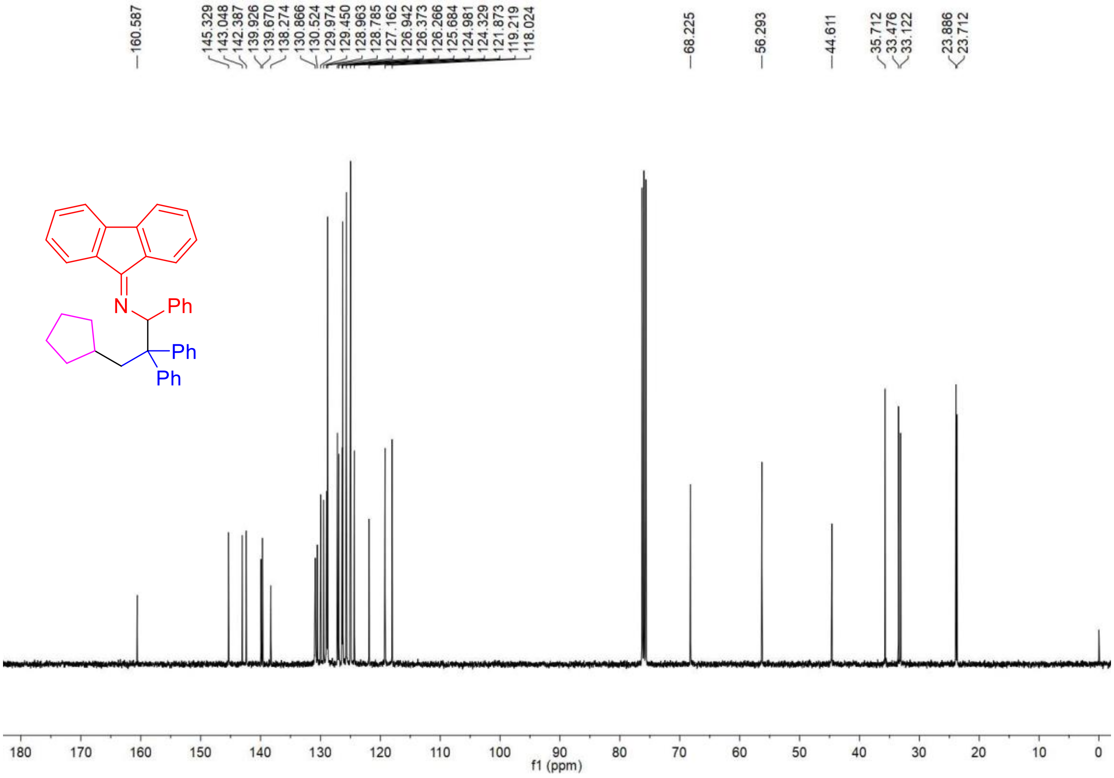

Figure S27. **^{1}H NMR spectra (400 MHz, Chloroform-*d*) of *N*-(3-(Cyclopent-3-en-1-yl)-1,2,2-triphenylpropyl)-9*H*-fluoren-9-imine (4ak).**

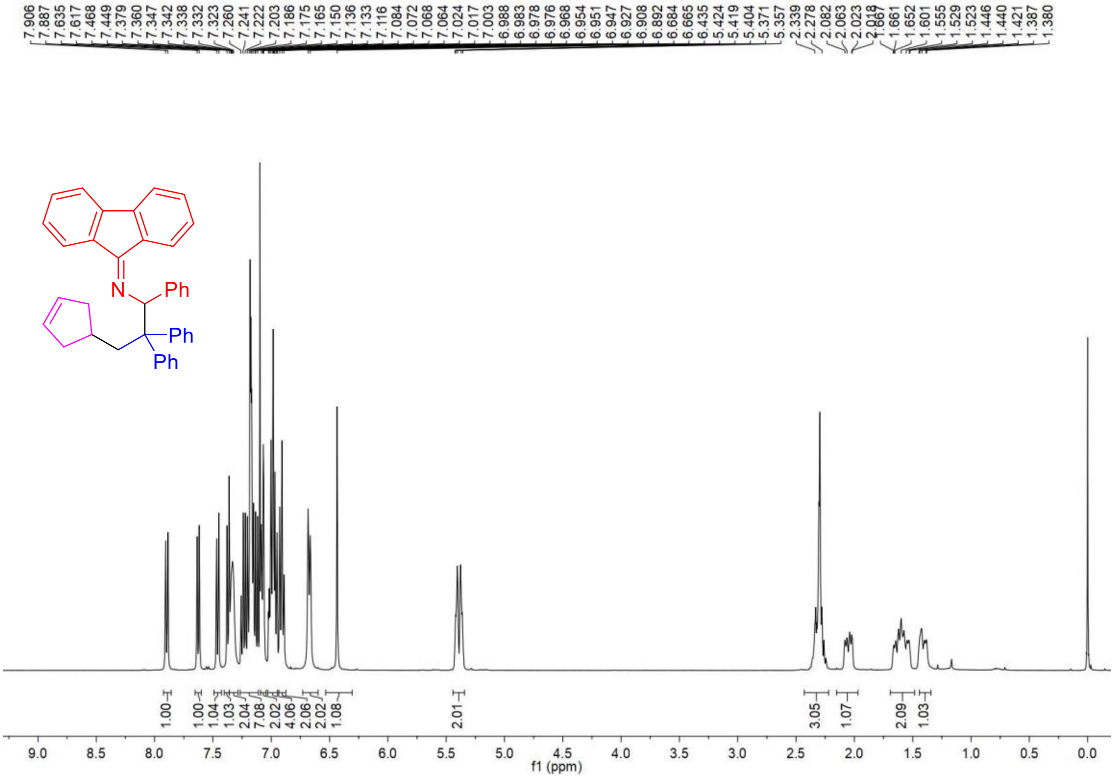

Figure S28. **^{13}C{^{1}H} NMR spectra (100 MHz, Chloroform-*d*) of *N*-(3-(Cyclopent-3-en-1-yl)-1,2,2-triphenylpropyl)-9*H*-fluoren-9-imine (4ak).**

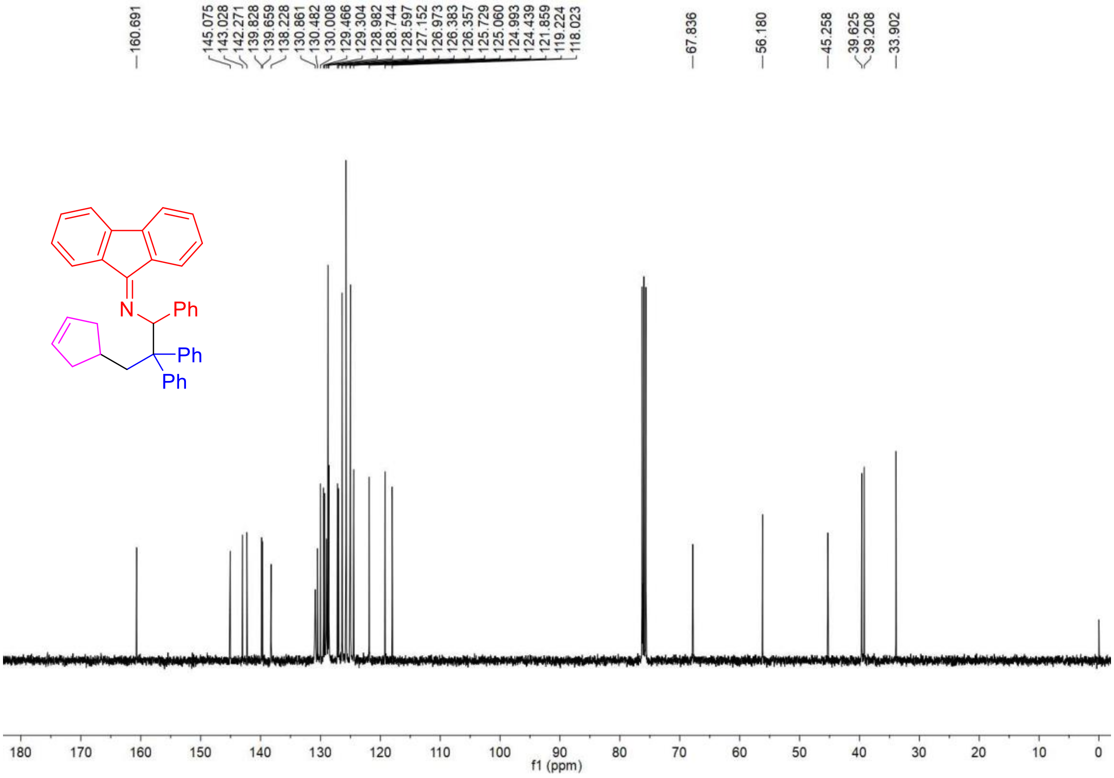

Figure S29. ^{1}**H NMR spectra (400 MHz, Chloroform-*d*) of *N*-(3-Cyclohexyl-1,2,2-triphenylpropyl)-9*H*-fluoren-9-imine (4al).**

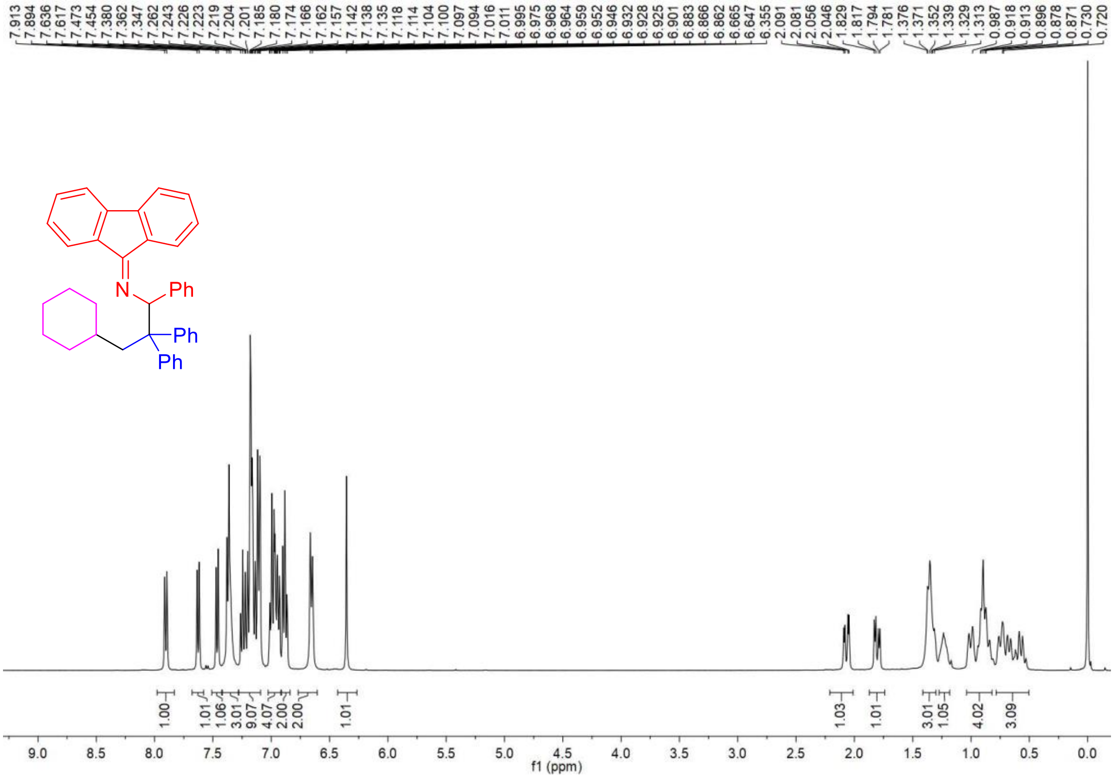

Figure S30. ^{13}**C{**^{1}**H} NMR spectra (100 MHz, Chloroform-*d*) of *N*-(3-Cyclohexyl-1,2,2-triphenylpropyl)-9*H*-fluoren-9-imine (4al).**

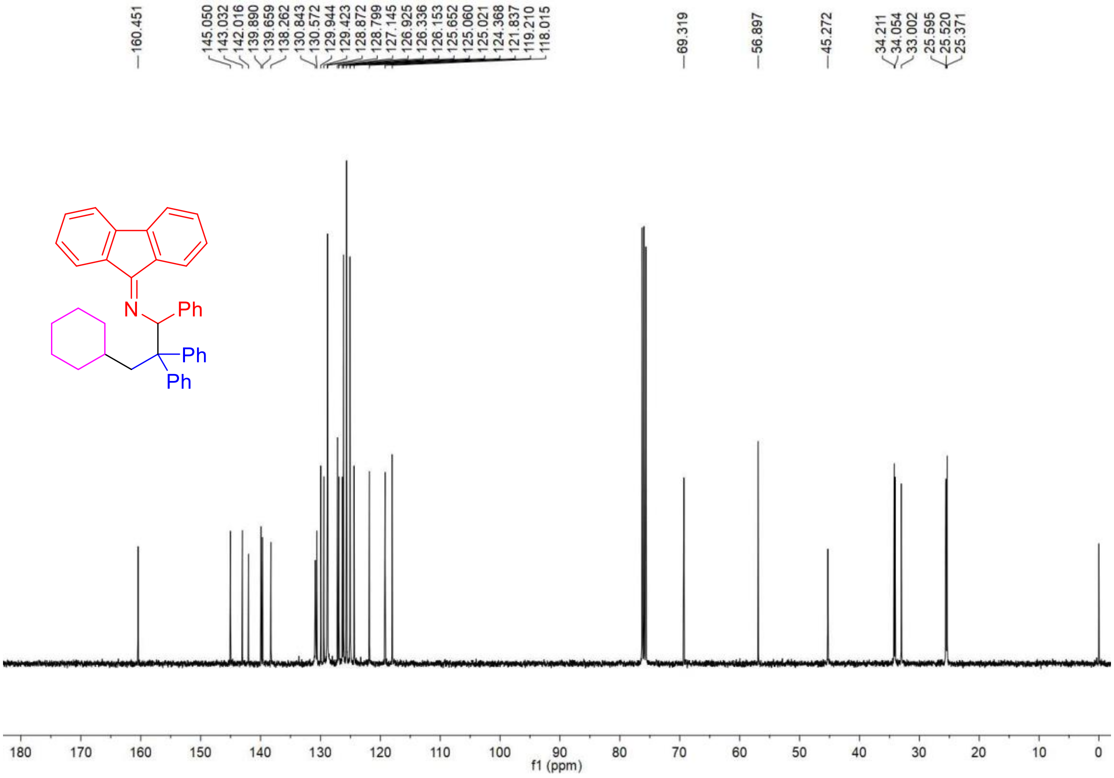

Figure S31. **^{1}H NMR spectra (400 MHz, Chloroform-*d*) of *tert*-Butyl 4-(3-((9*H*-fluoren-9-ylidene)amino)-2,2,3-triphenylpropyl)piperi-dine-1-carboxylate (4am).**

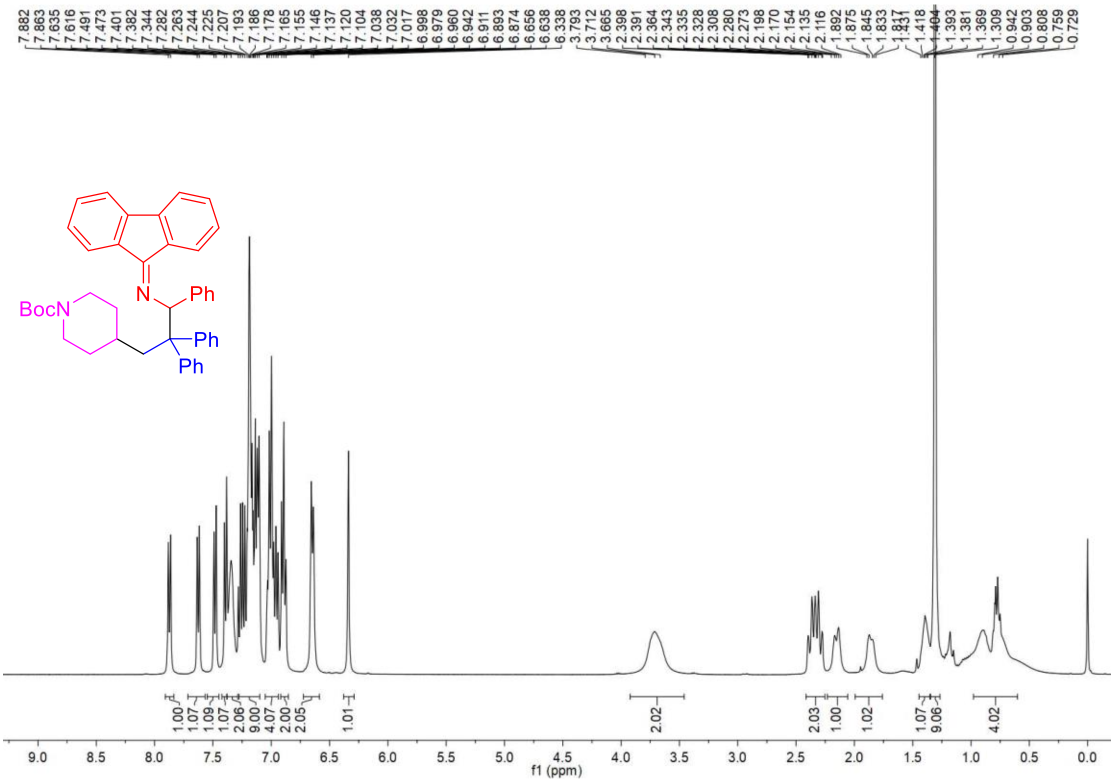

Figure S32. **^{13}C{^{1}H} NMR spectra (100 MHz, Chloroform-*d*) of *tert*-Butyl 4-(3-((9*H*-fluoren-9-ylidene)amino)-2,2,3-triphenylpropyl)piperi-dine-1-carboxylate (4am).**

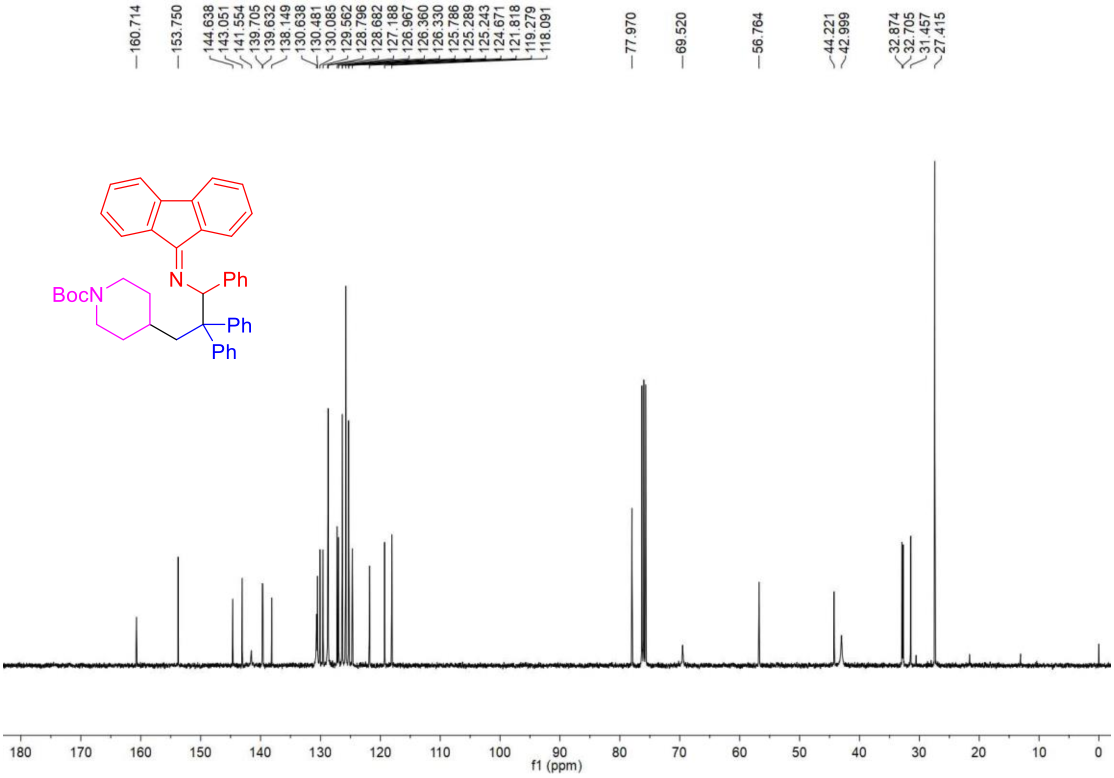

Figure S33. ^{1}**H NMR spectra (400 MHz, Chloroform-*d*) of *N*-(3-(4,4-Difluorocyclohexyl)-1,2,2-triphenylpropyl)-9*H*-fluoren-9-imine (4an).**

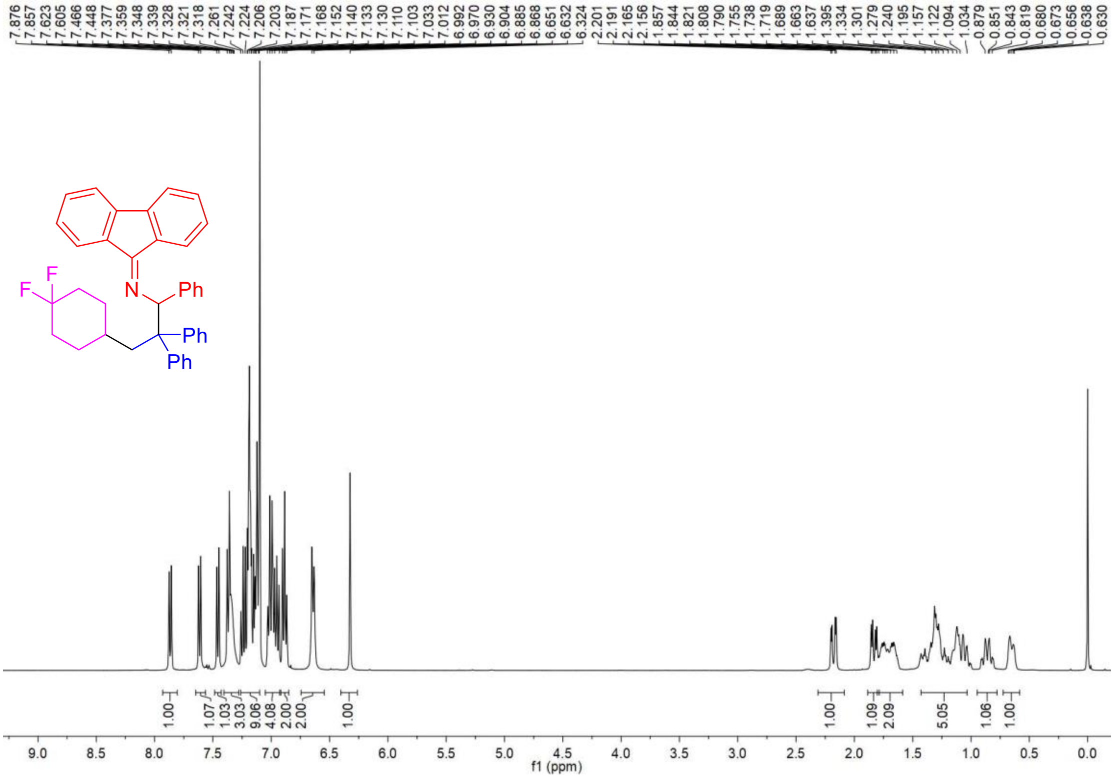

Figure S34. ^{13}**C{^{1}H} NMR spectra (100 MHz, Chloroform-*d*) of *N*-(3-(4,4-Difluorocyclohexyl)-1,2,2-triphenylpropyl)-9*H*-fluoren-9-imine (4an).**

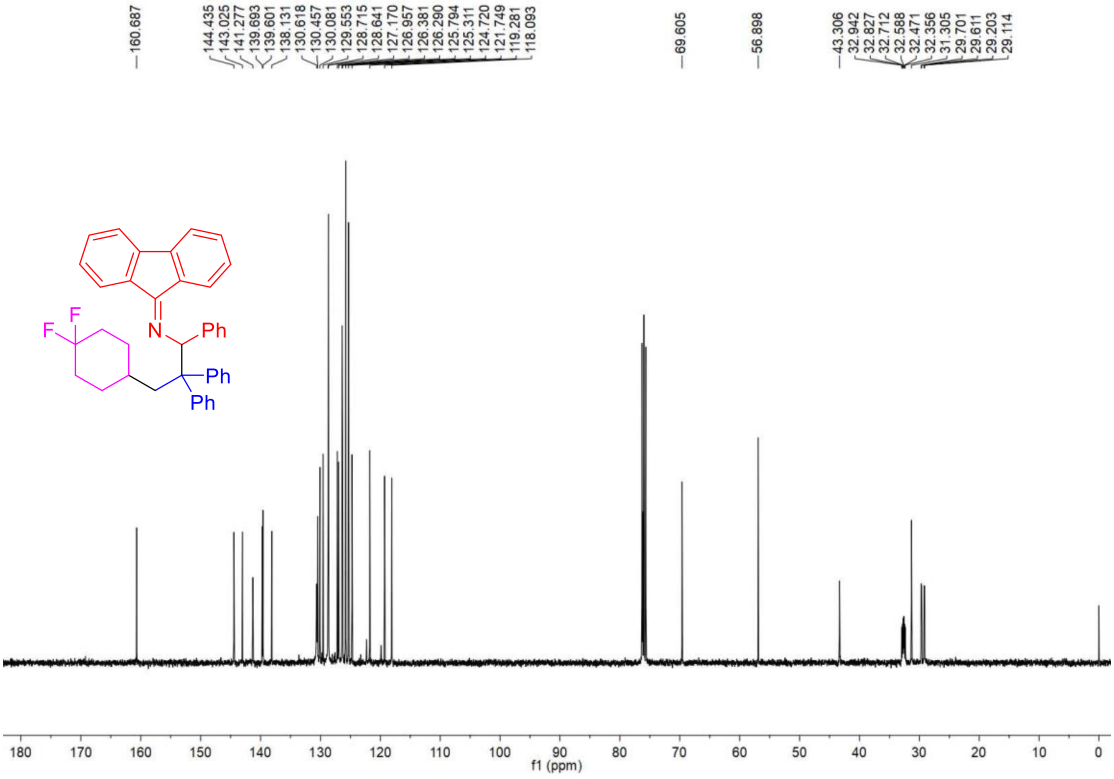

Figure S35. **^{19}F NMR spectra (376 MHz, Chloroform-*d*) of *N*-(3-(4,4-Difluorocyclohexyl)-1,2,2-triphenylpropyl)-9*H*-fluoren-9-imine (4an).**

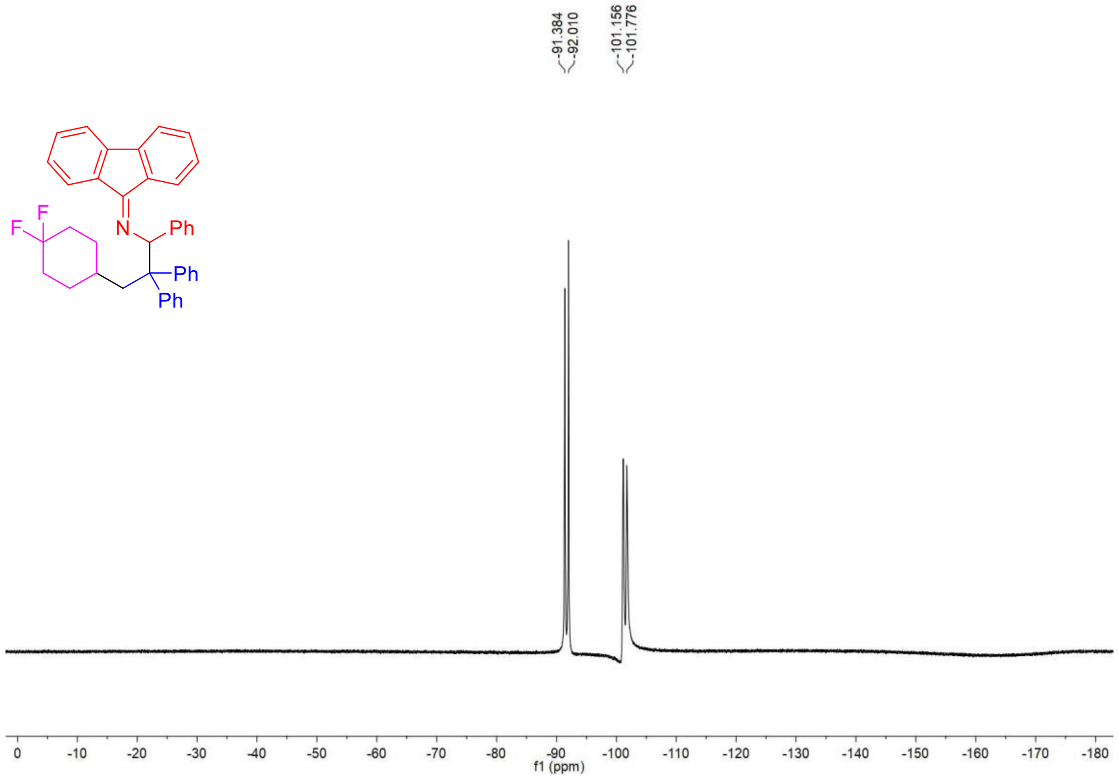

Figure S36. ^{1}H NMR spectra (400 MHz, Chloroform-*d*) of *N*-(3-Cycloheptyl-1,2,2-triphenylpropyl)-9*H*-fluoren-9-imine (4ao).

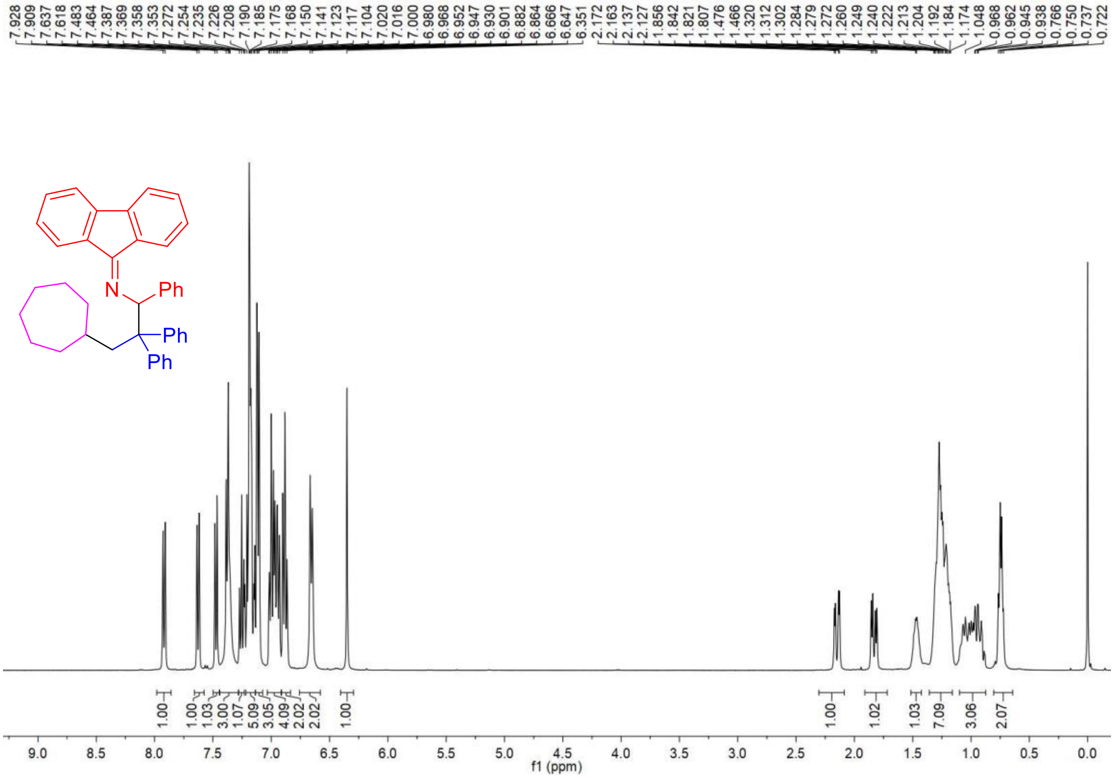

Figure S37. ^{13}C{^{1}H} NMR spectra (100 MHz, Chloroform-*d*) of *N*-(3-Cycloheptyl-1,2,2-triphenylpropyl)-9*H*-fluoren-9-imine (4ao).

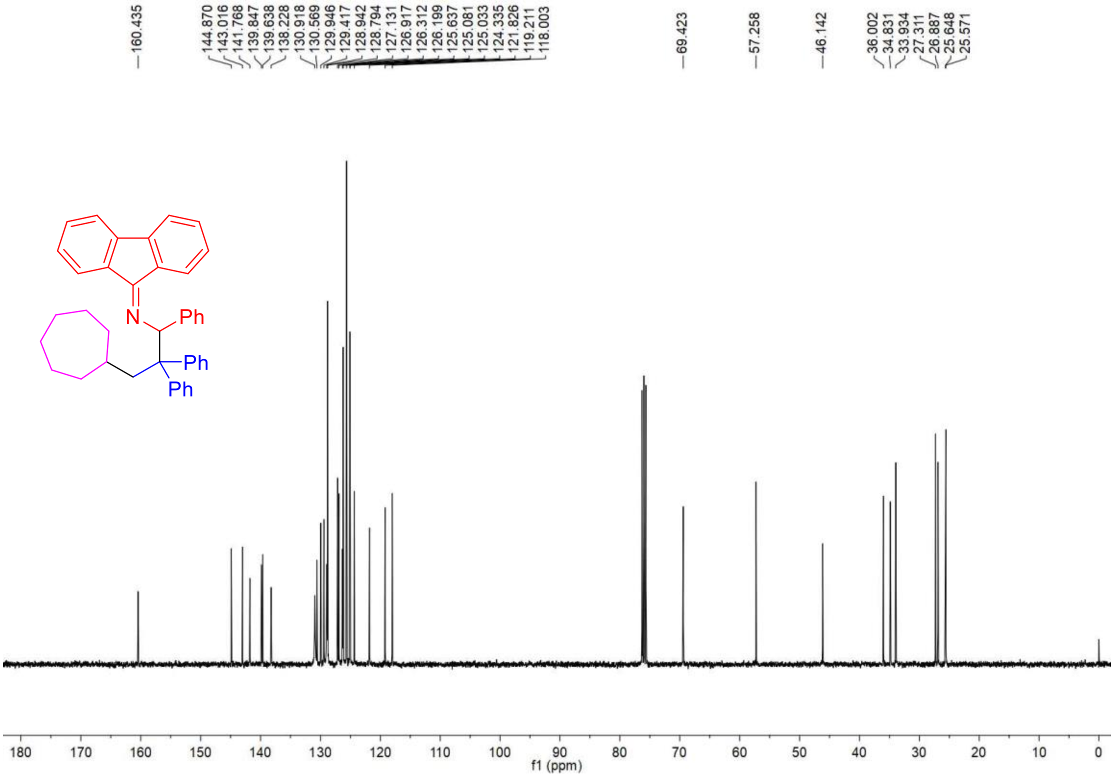

Figure S38. ^{1}**H NMR spectra (400 MHz, Chloroform-*d*) of *N*-(4,4-Dimethyl-1,2,2-triphenylpentyl)-9*H*-fluoren-9-imine (4ap).**

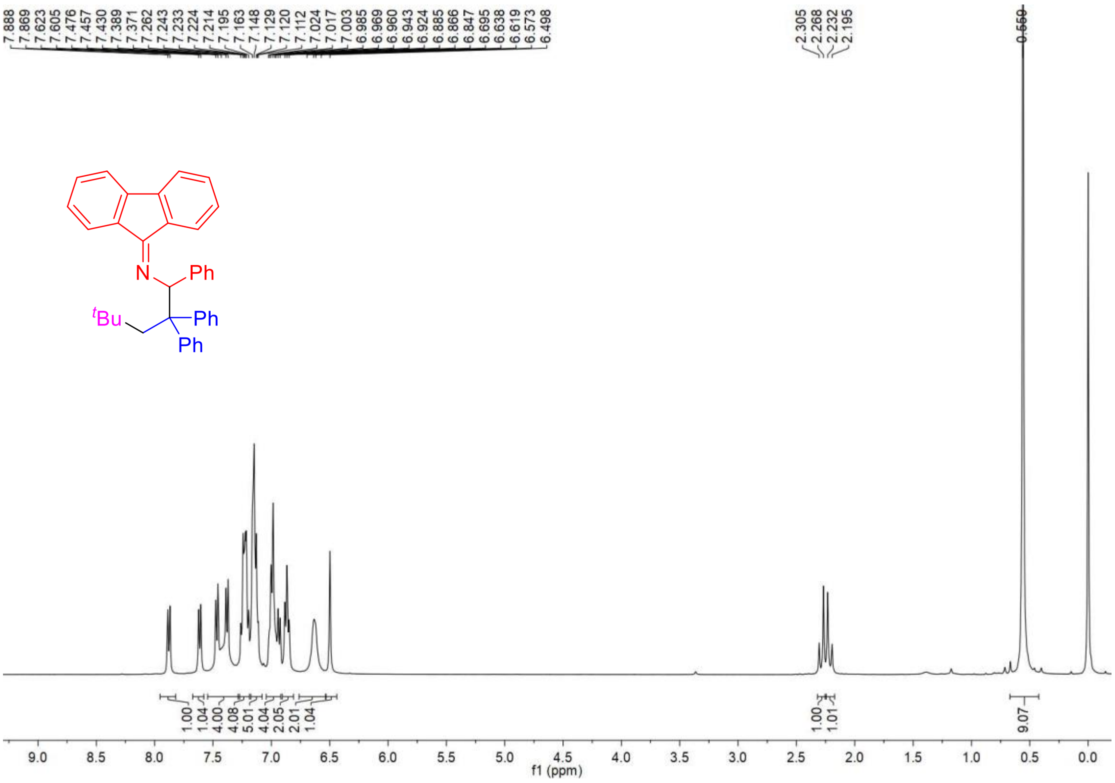

Figure S39. ^{13}**C{^{1}H} NMR spectra (100 MHz, Chloroform-*d*) of *N*-(4,4-Dimethyl-1,2,2-triphenylpentyl)-9*H*-fluoren-9-imine (4ap).**

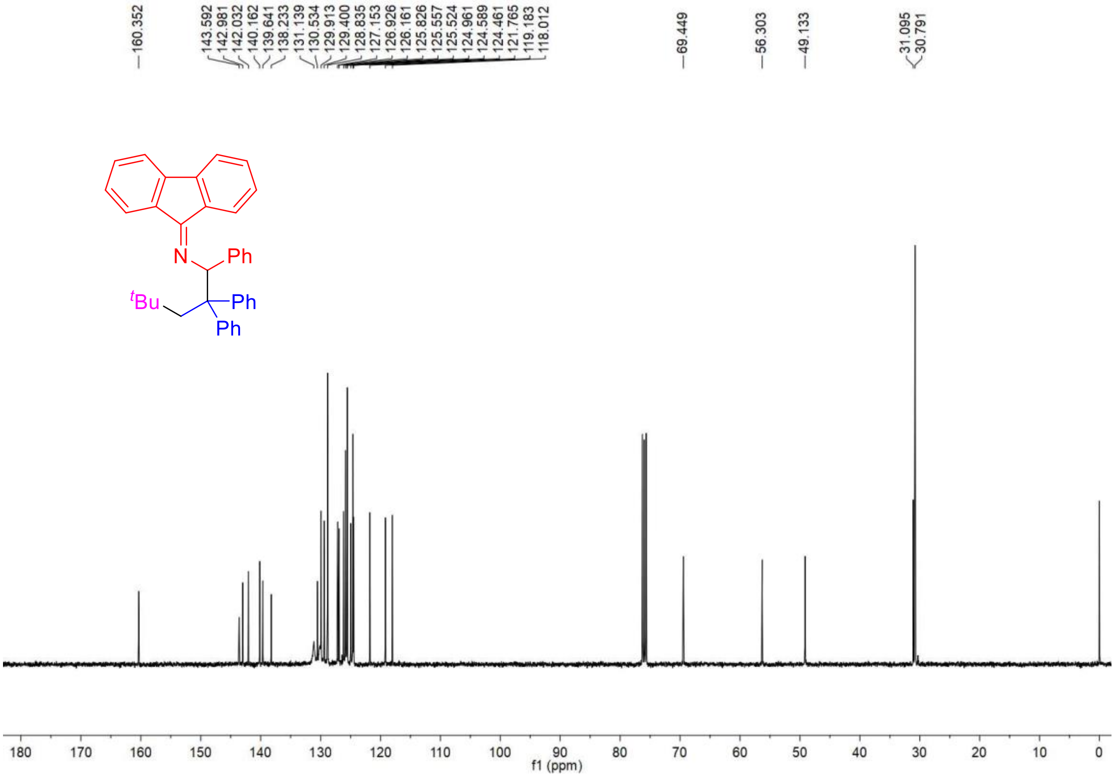

Figure S40. [1]**H NMR spectra (400 MHz, Chloroform-*d*) of *N*-(5,5,5-Trifluoro-4,4-dimethyl-1,2,2-triphenylpentyl)-9*H*-fluoren-9-imine (4aq).**

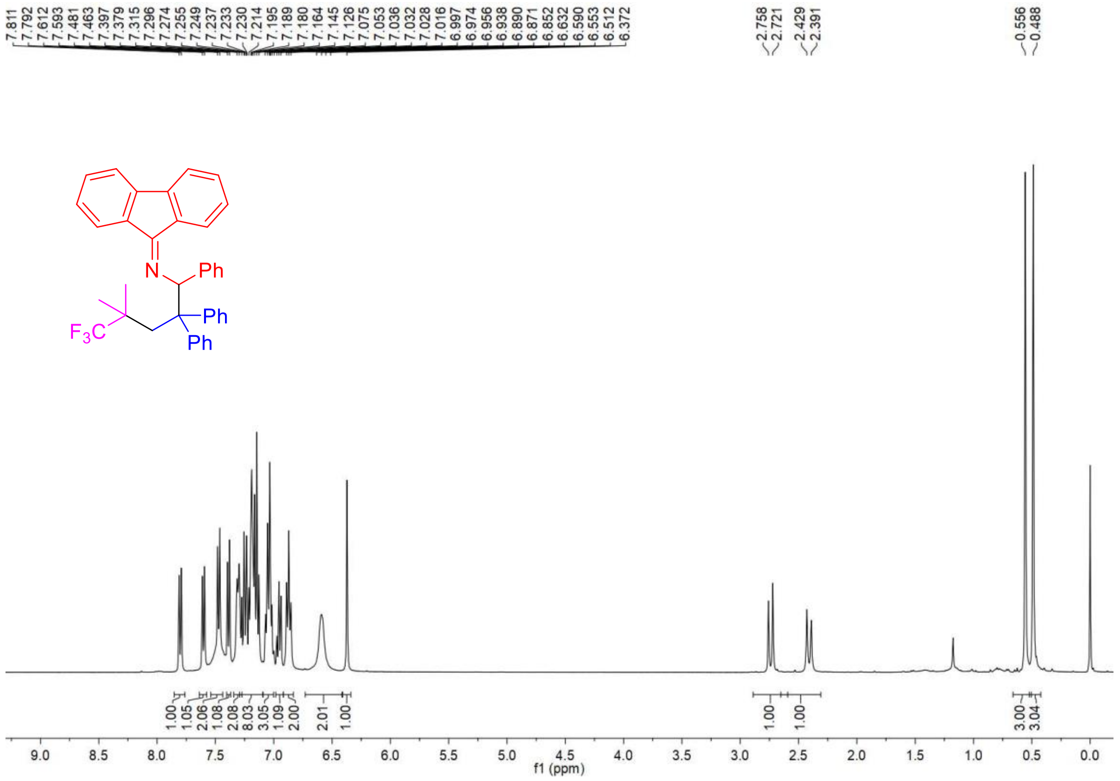

Figure S41. [13]**C{**[1]**H} NMR spectra (100 MHz, Chloroform-*d*) of *N*-(5,5,5-Trifluoro-4,4-dimethyl-1,2,2-triphenylpentyl)-9*H*-fluoren-9-imine (4aq).**

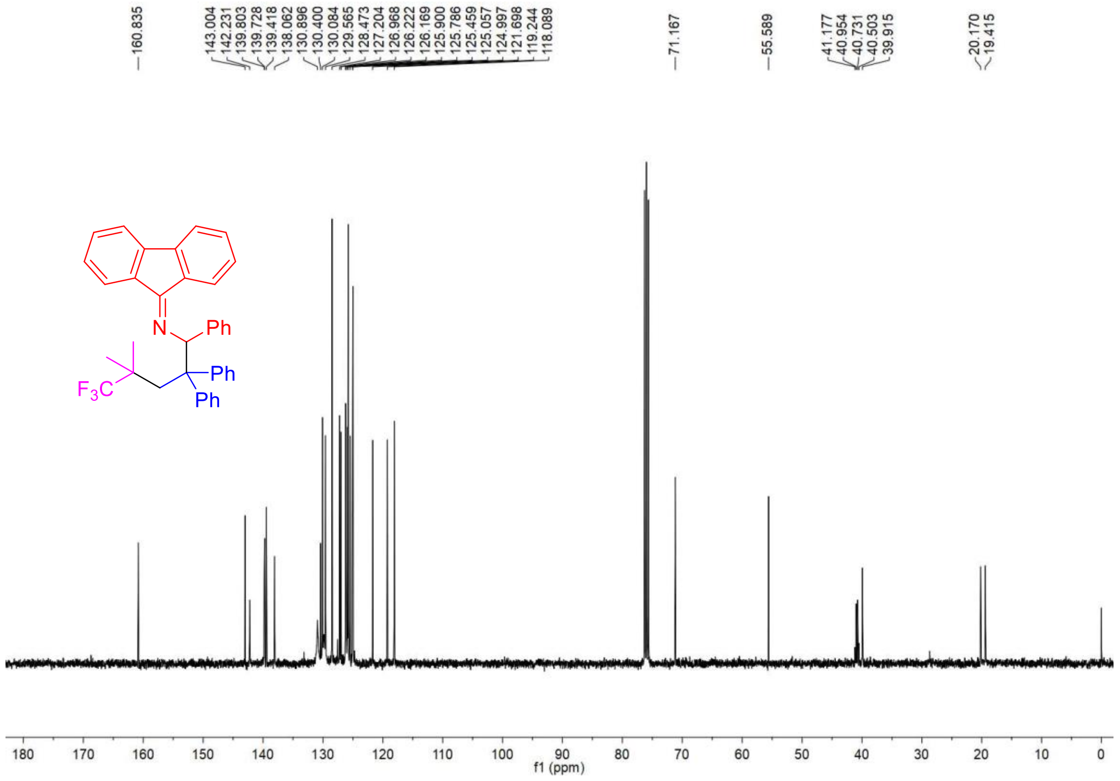

Figure S42. **^{19}F NMR spectra (376 MHz, Chloroform-*d*) of *N*-(5,5,5-Trifluoro-4,4-dimethyl-1,2,2-triphenylpentyl)-9*H*-fluoren-9-imine (4aq).**

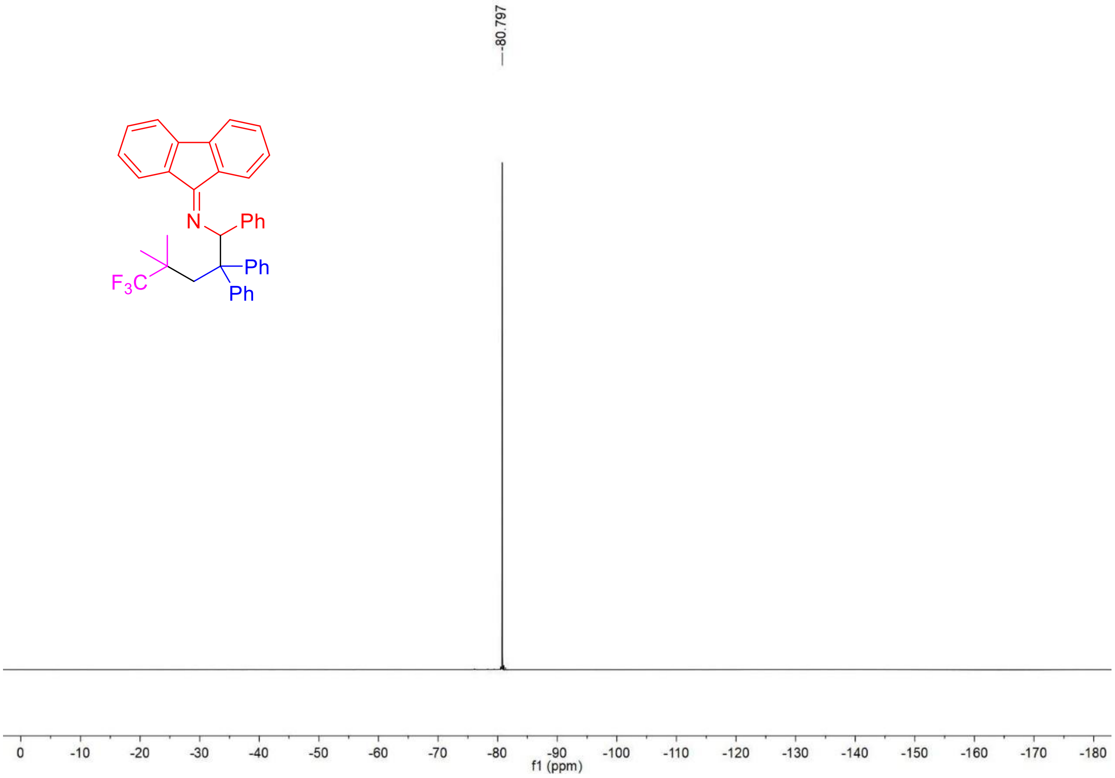

Figure S43. **^{1}H NMR spectra (400 MHz, Chloroform-*d*) of *N*-(3-(1-Methylcyclopropyl)-1,2,2-triphenylpropyl)-9*H*-fluoren-9-imine (4ar).**

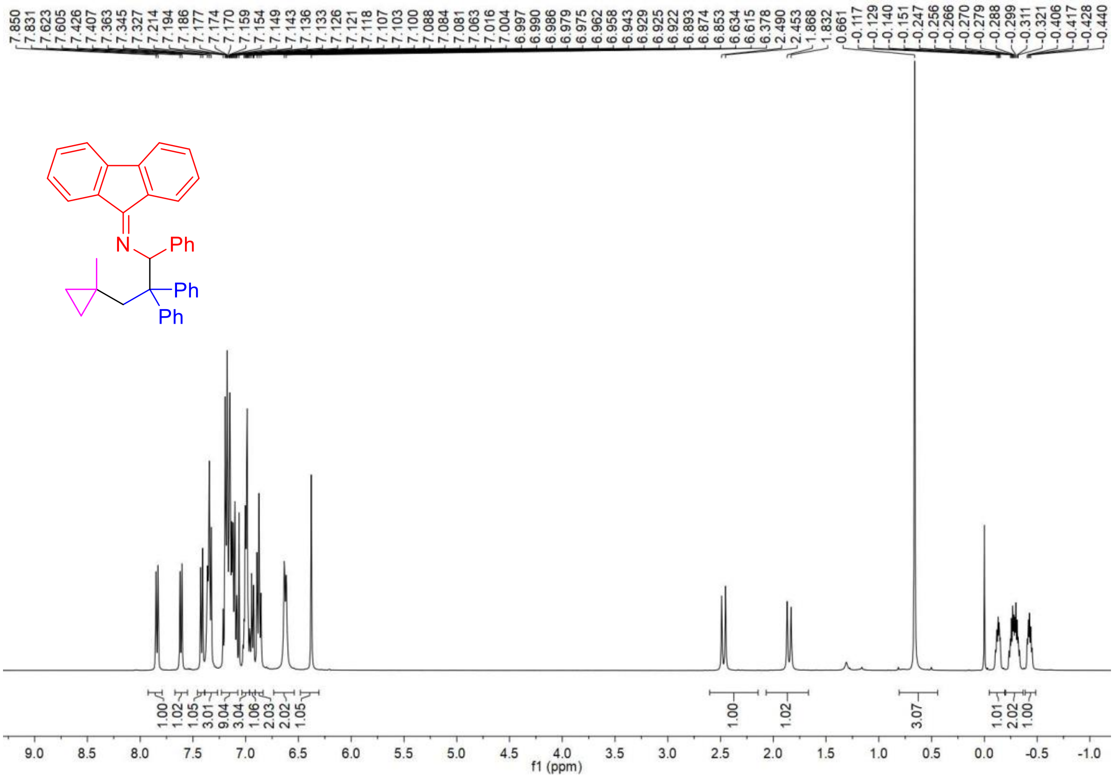

Figure S44. **^{13}C{^{1}H} NMR spectra (100 MHz, Chloroform-*d*) of *N*-(3-(1-Methylcyclopropyl)-1,2,2-triphenylpropyl)-9*H*-fluoren-9-imine(4ar).**

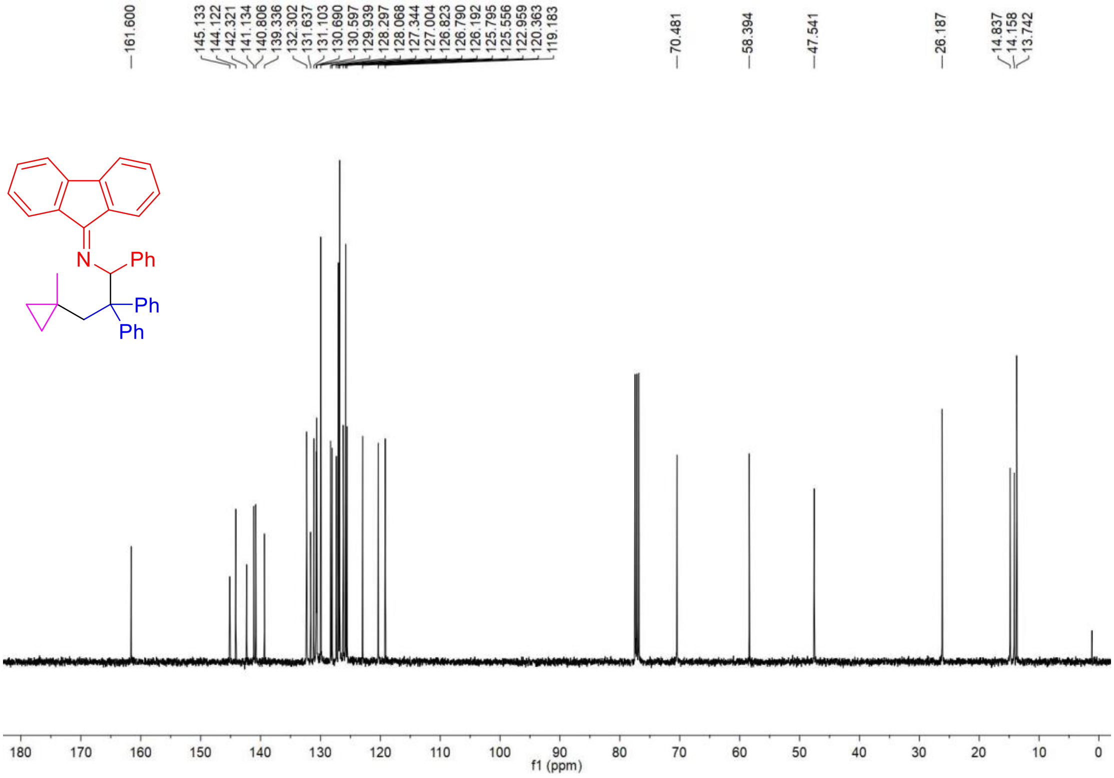

Figure S45. ^{1}**H NMR spectra (400 MHz, Chloroform-*d*) of *N*-(3-(1-Methylcyclobutyl)-1,2,2-triphenylpropyl)-9*H*-fluoren-9-imine (4as).**

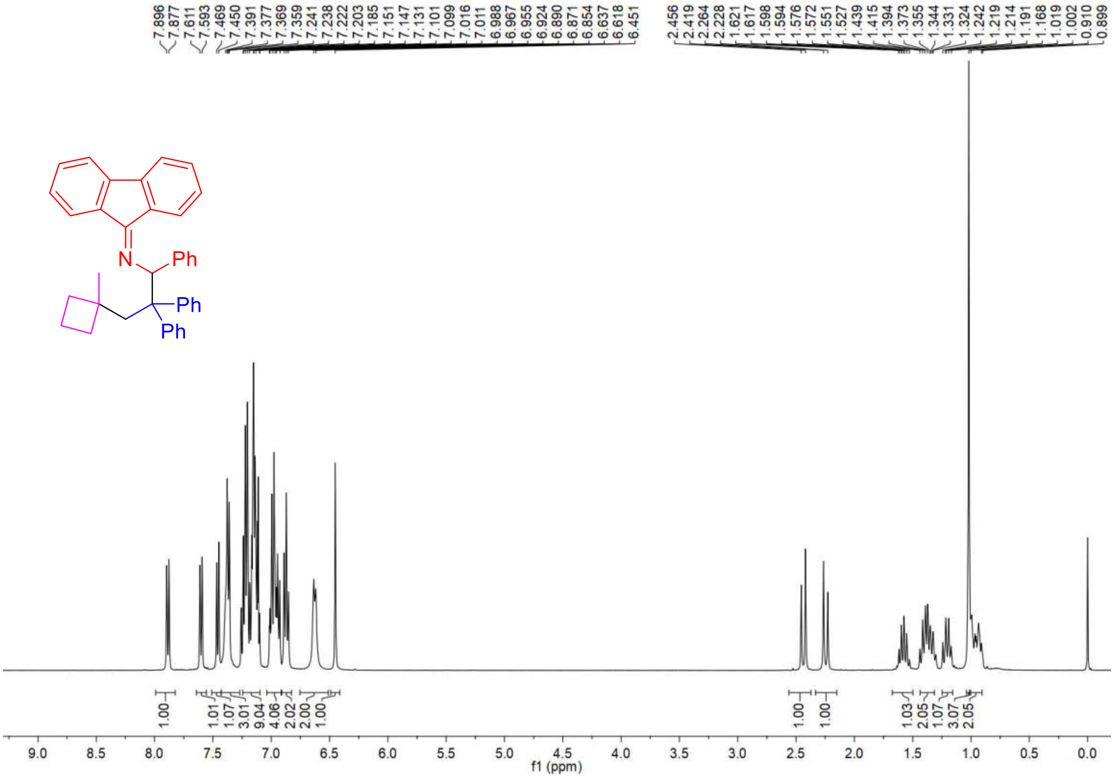

Figure S46. ^{13}**C{**^{1}**H} NMR spectra (100 MHz, Chloroform-*d*) of *N*-(3-(1-Methylcyclobutyl)-1,2,2-triphenylpropyl)-9*H*-fluoren-9-imine (4as).**

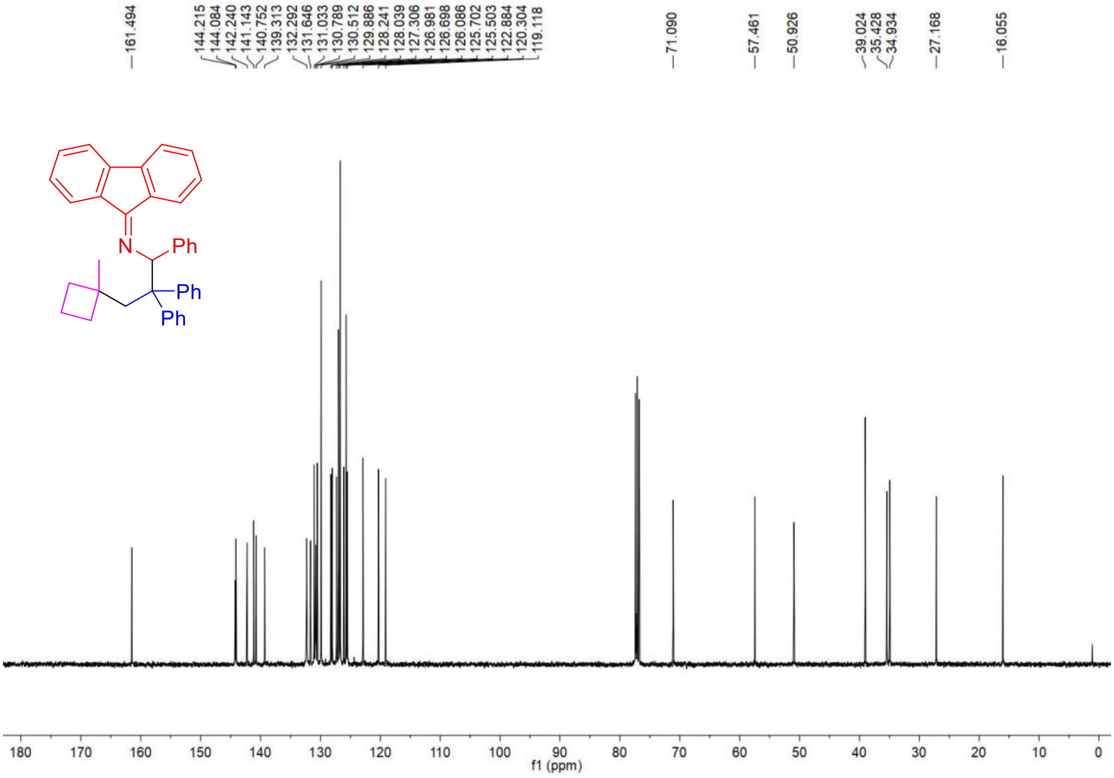

Figure S47. ^{1}H **NMR spectra (400 MHz, Chloroform-*d*) of *N*-(1,2,2-Triphenyl-3-(1-(trifluoromethyl)cyclobutyl)propyl)-9*H*-fluoren-9-imine (4at).**

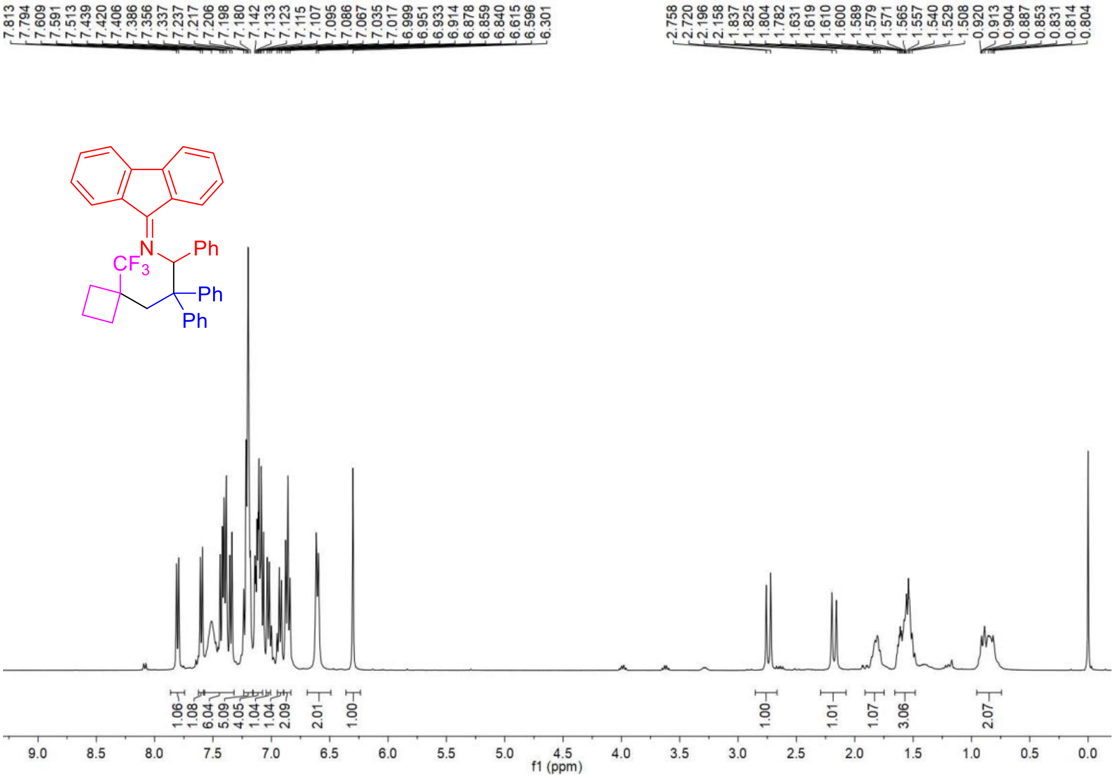

Figure S48. $^{13}C\{^{1}H\}$ **NMR spectra (100 MHz, Chloroform-*d*) of *N*-(1,2,2-Triphenyl-3-(1-(trifluoromethyl)cyclobutyl)propyl)-9*H*-fluoren-9-imine (4at).**

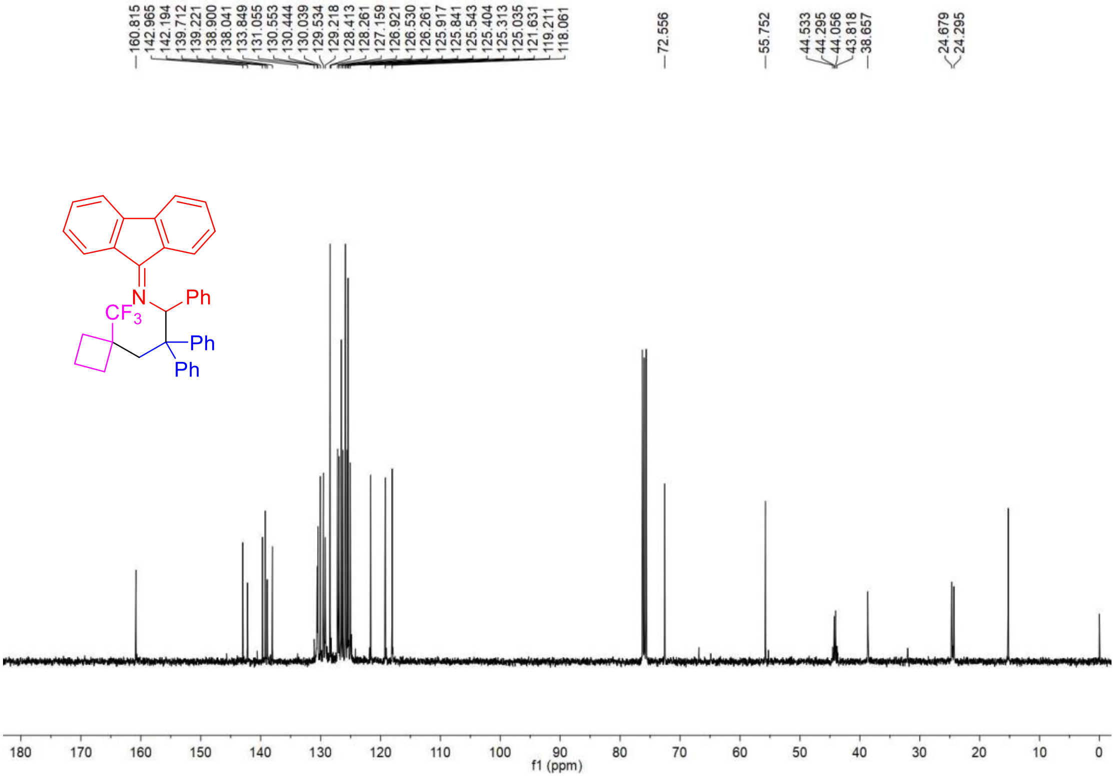

Figure S49. ^{19}**F NMR spectra (376 MHz, Chloroform-*d*) of *N*-(1,2,2-Triphenyl-3-(1-(trifluoromethyl)cyclobutyl)propyl)-9*H*-fluoren-9-imine (4at).**

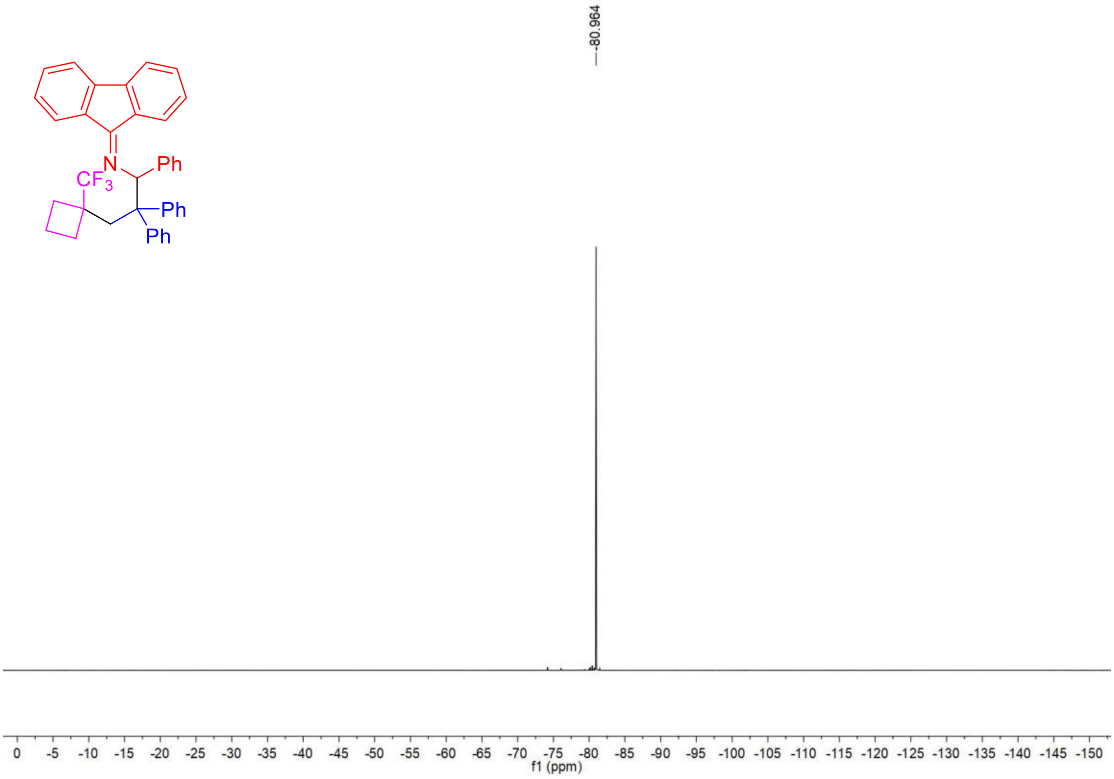

Figure S50. **^{1}H NMR spectra (400 MHz, Chloroform-*d*) of *N*-(3-((3r,5r,7r)-Adamantan-1-yl)-1,2-diphenylpropyl)-9*H*-fluoren-9-imine (4au').**

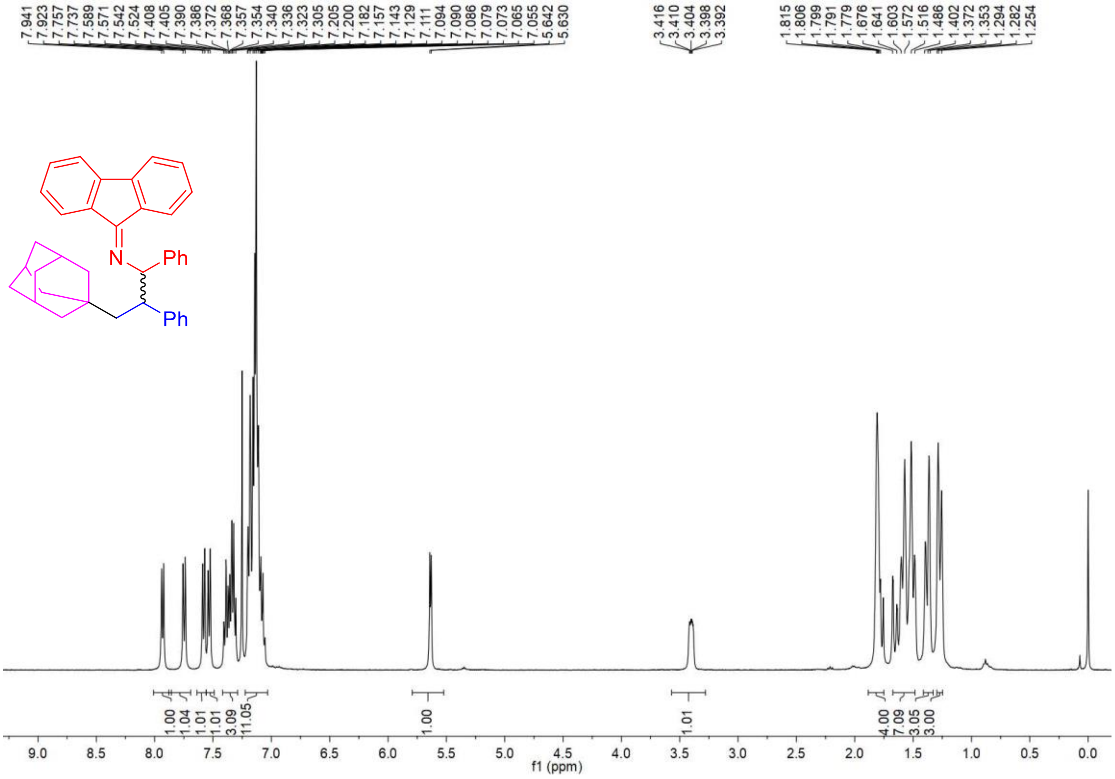

Figure S51. **^{13}C{^{1}H} NMR spectra (100 MHz, Chloroform-*d*) of *N*-(3-((3r,5r,7r)-Adamantan-1-yl)-1,2-diphenylpropyl)-9*H*-fluoren-9-imine (4au').**

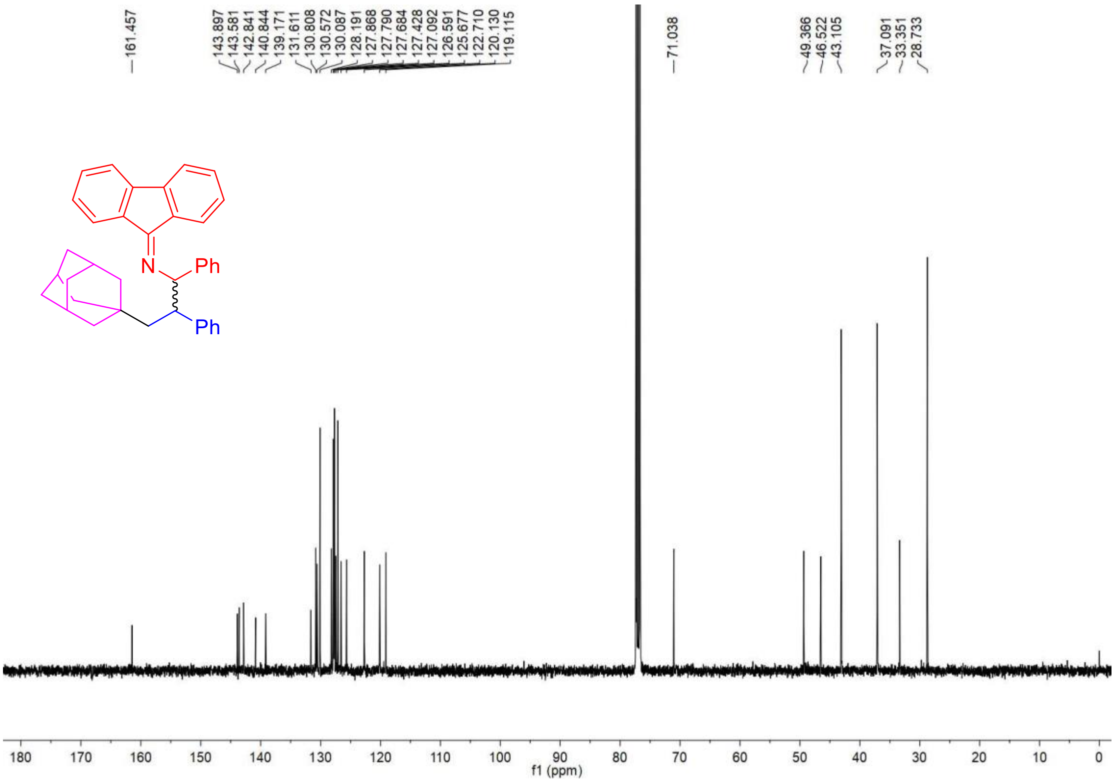

Figure S52. **^{1}H NMR spectra (400 MHz, Chloroform-*d*) of *N*-(3-((3r,5r,7r)-Adamantan-1-yl)-1,2-diphenylpropyl)-9*H*-fluoren-9-imine (4au'').**

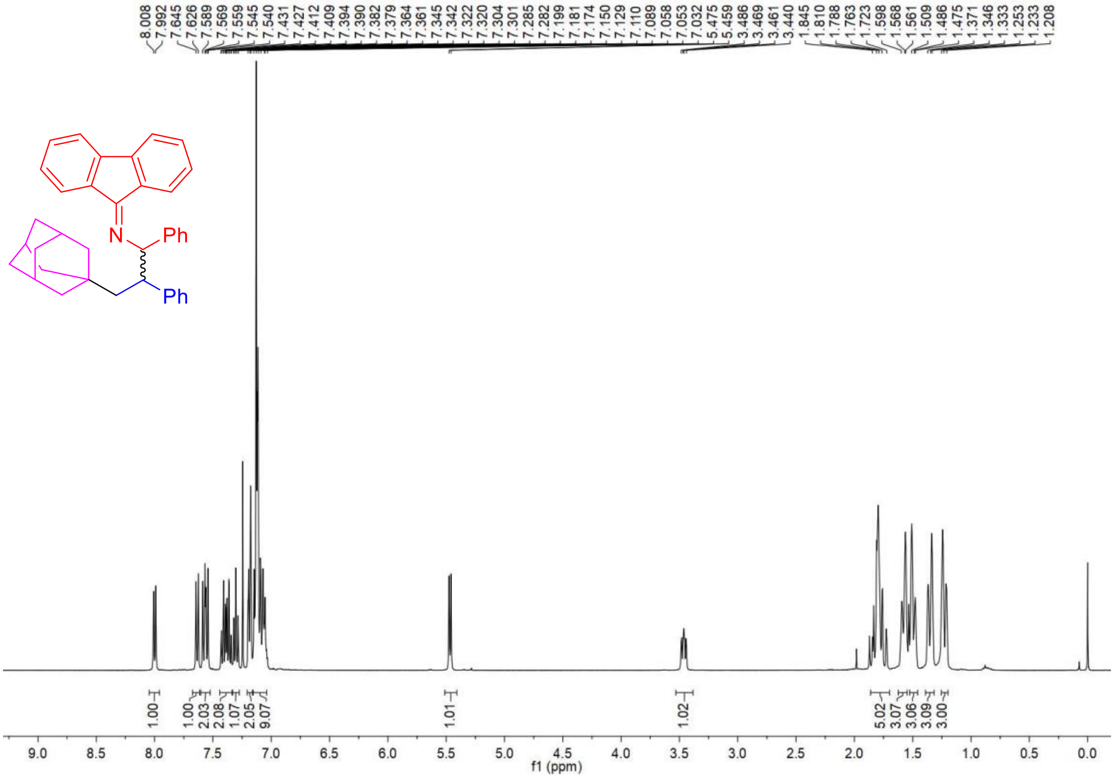

Figure S53. **^{13}C{^{1}H} NMR spectra (100 MHz, Chloroform-*d*) of *N*-(3-((3r,5r,7r)-Adamantan-1-yl)-1,2-diphenylpropyl)-9*H*-fluoren-9-imine (4au'').**

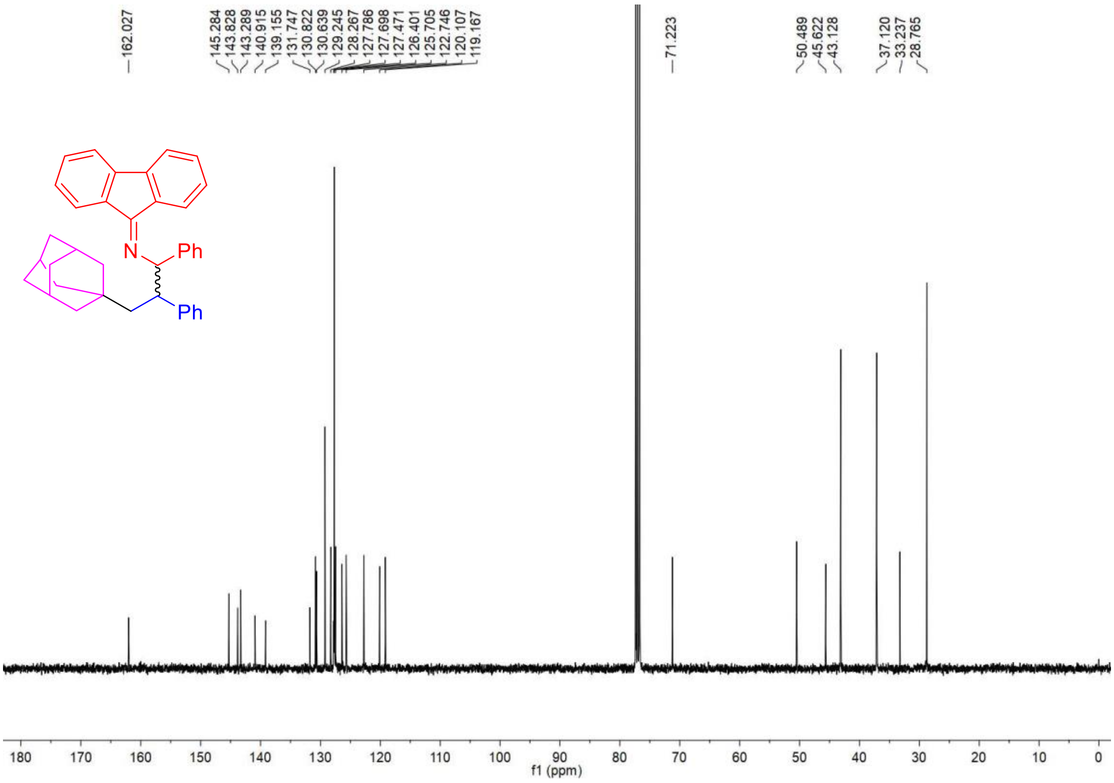

Figure S54. ^{1}**H NMR spectra (400 MHz, Chloroform-*d*) of *tert*-Butyl 4-(3-((9*H*-fluoren-9-ylidene)amino)-2,3-diphenylpropyl)piperi-dine-1-carboxylate (4mu').**

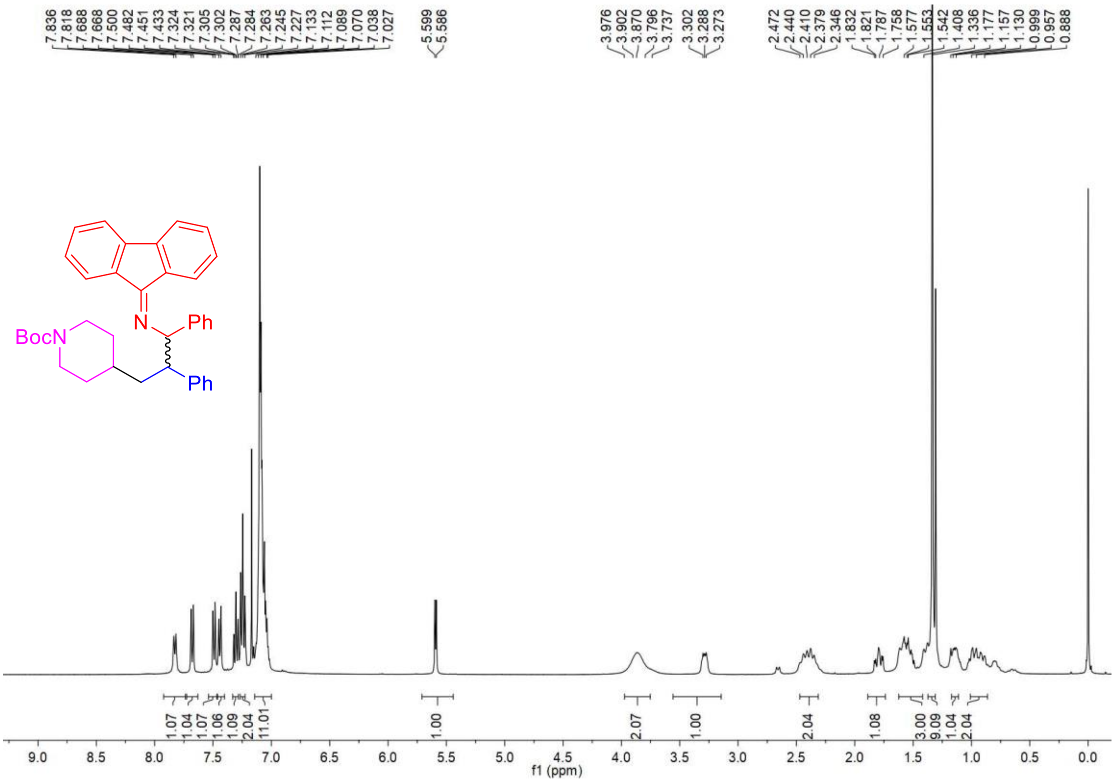

Figure S55. ^{13}**C{**^{1}**H} NMR spectra (100 MHz, Chloroform-*d*) of *tert*-Butyl 4-(3-((9*H*-fluoren-9-ylidene)amino)-2,3-diphenylpropyl)piperi-dine-1-carboxylate (4mu').**

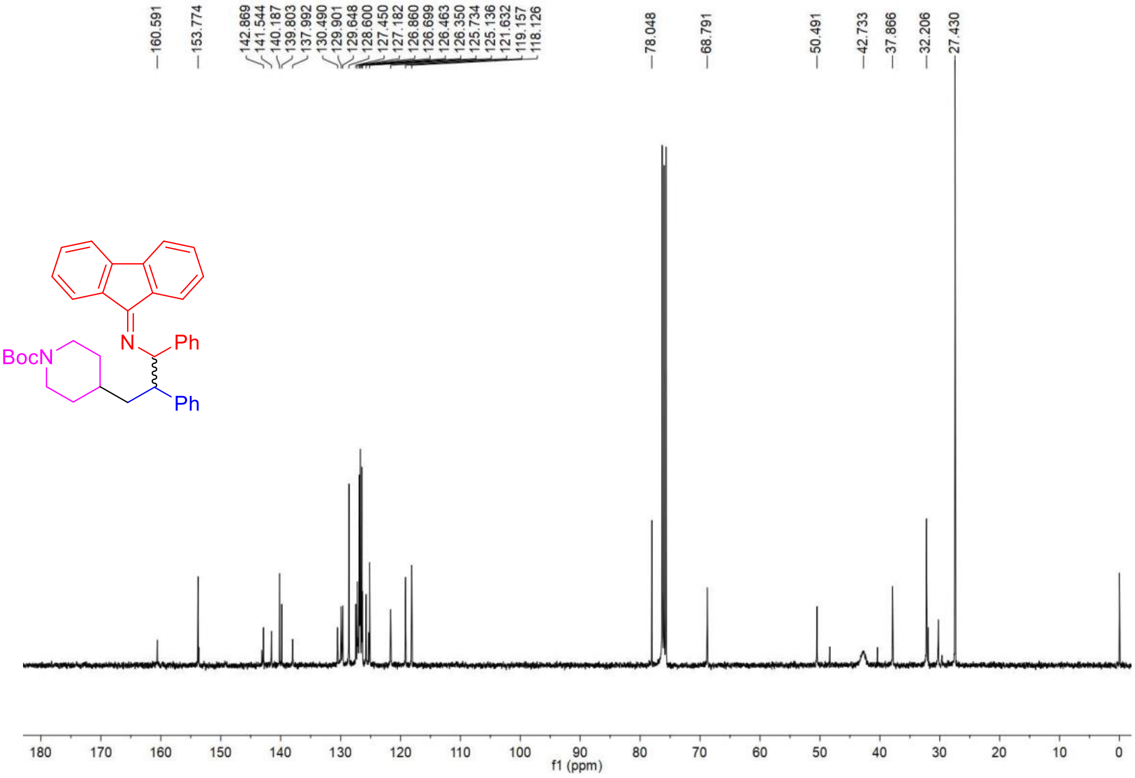

Figure S56. ^{1}**H NMR spectra (400 MHz, Chloroform-*d*) of *tert*-Butyl 4-(3-((9*H*-fluoren-9-ylidene)amino)-2,3-diphenylpropyl)piperi-dine-1-carboxylate (4mu’’).**

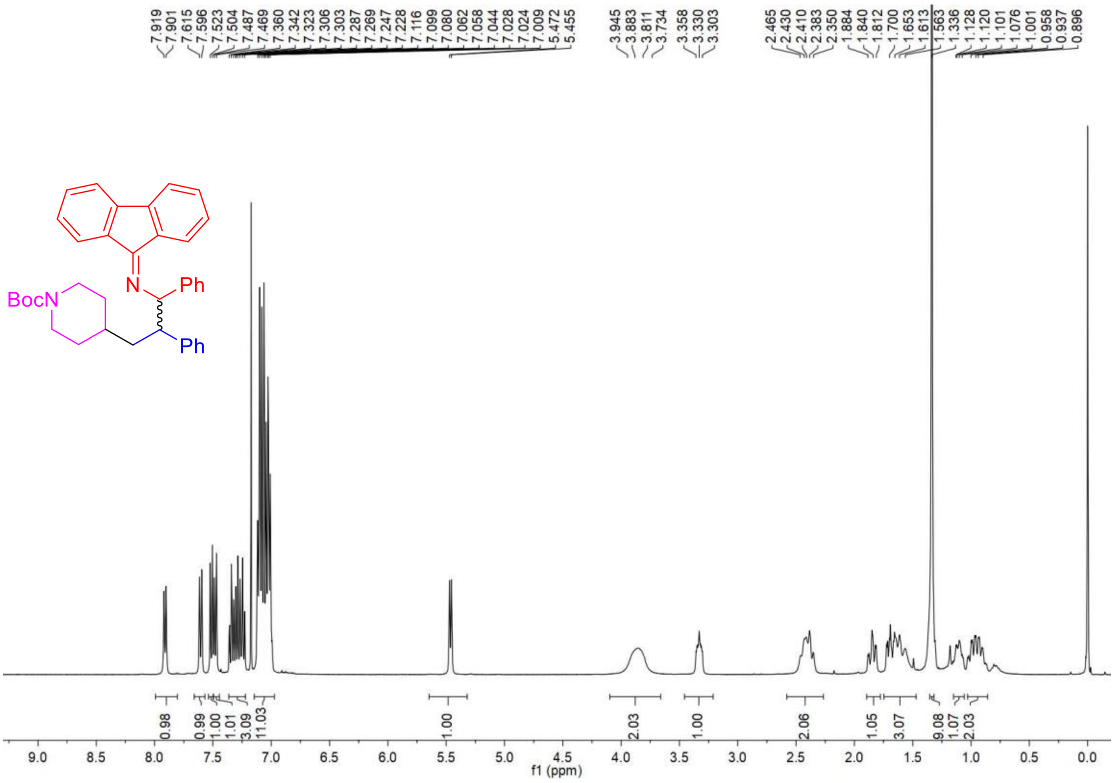

Figure S57. ^{13}**C{**^{1}**H} NMR spectra (100 MHz, Chloroform-*d*) of *tert*-Butyl 4-(3-((9*H*-fluoren-9-ylidene)amino)-2,3-diphenylpropyl)piperi-dine-1-carboxylate (4mu’’).**

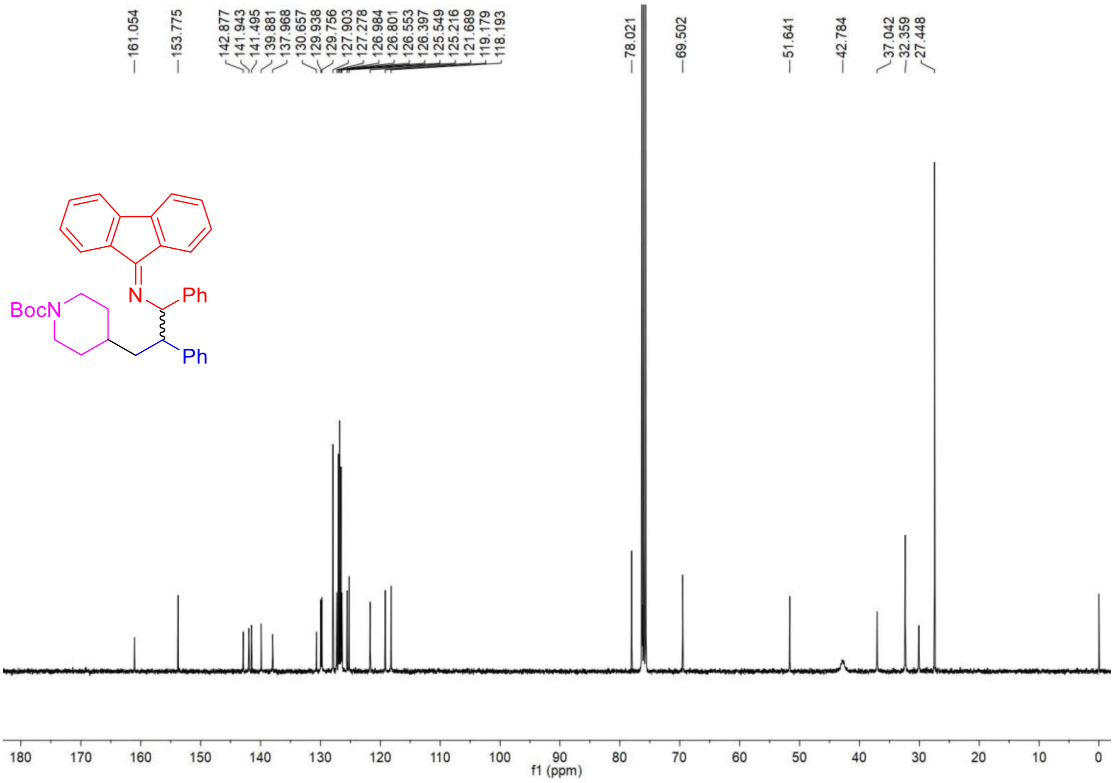

Figure S58. ^{1}**H NMR spectra (400 MHz, Chloroform-*d*) of *tert*-Butyl 4-(3-((9*H*-fluoren-9-ylidene)amino)-2-methyl-2,3-diphenylpro-pyl)piperidine-1-carboxylate (4mv'**(major)**).**

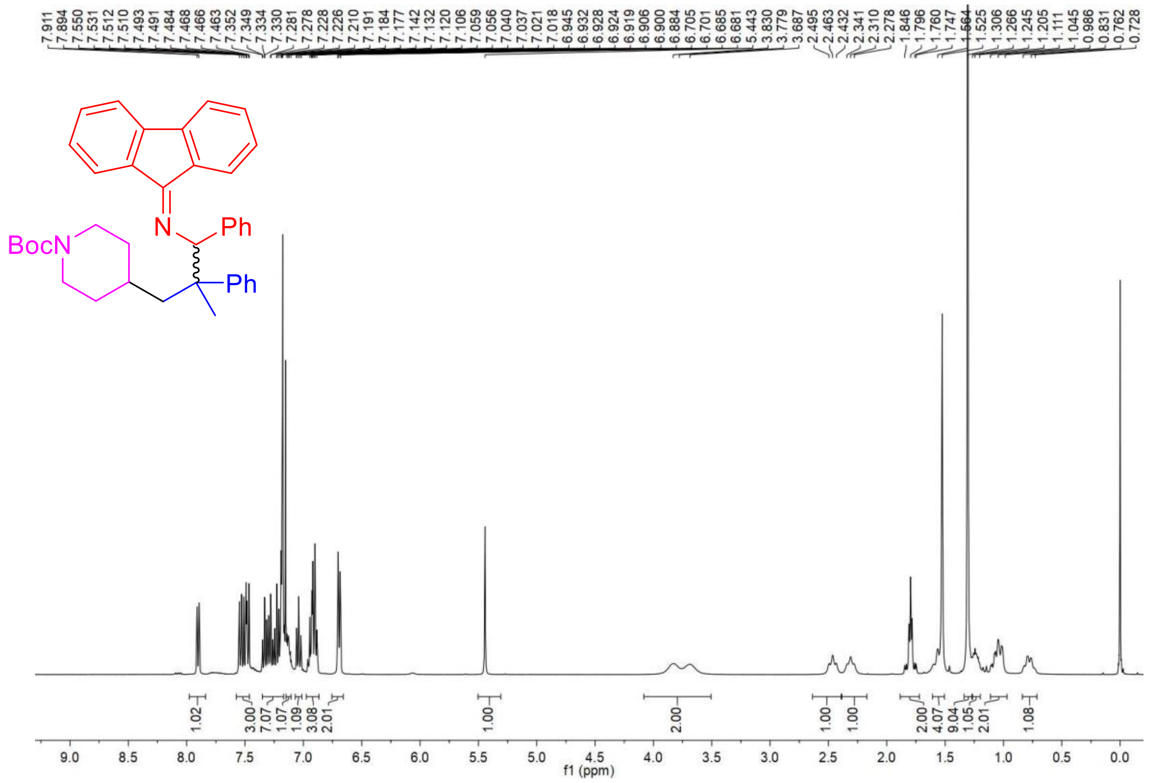

Figure S59. ^{13}**C{**^{1}**H} NMR spectra (100 MHz, Chloroform-*d*) of *tert*-Butyl 4-(3-((9*H*-fluoren-9-ylidene)amino)-2-methyl-2,3-diphenylpro-pyl)piperidine-1-carboxylate (4mv'**(major)**).**

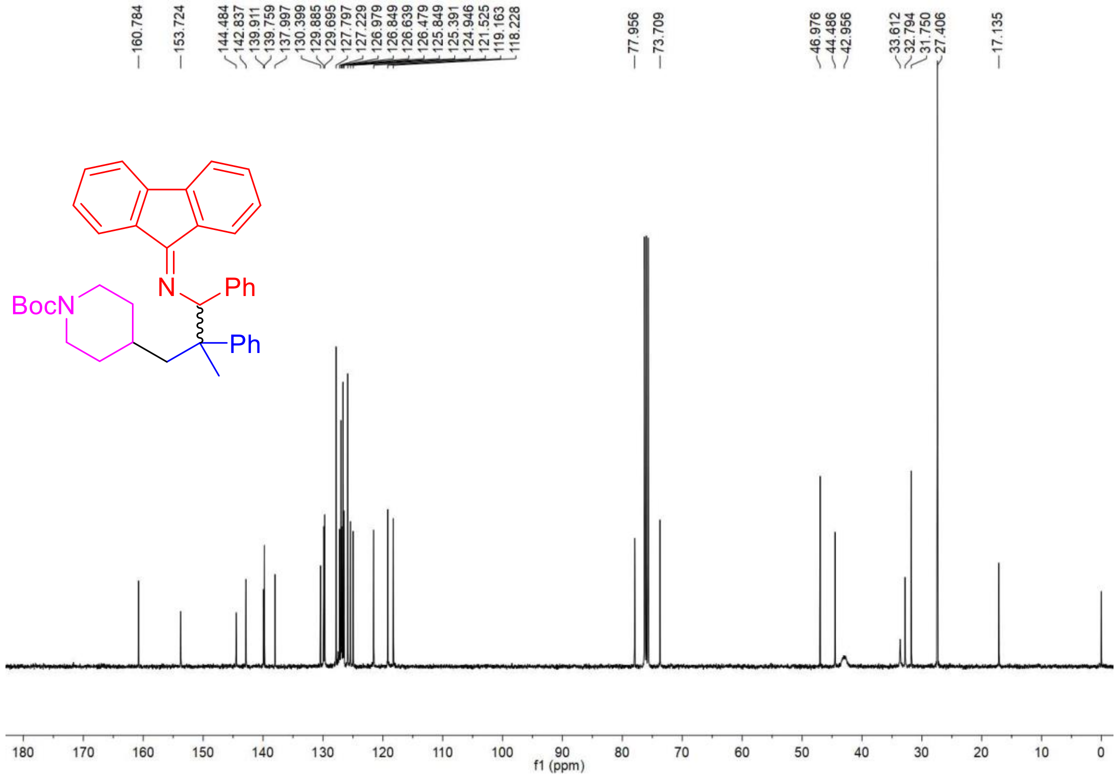

Figure S60. **^{1}H NMR spectra (400 MHz, Chloroform-*d*) of *tert*-Butyl 4-(3-((9*H*-fluoren-9-ylidene)amino)-2-methyl-2,3-diphenylpro-pyl)piperidine-1-carboxylate (4mv'')**(minor)**).**

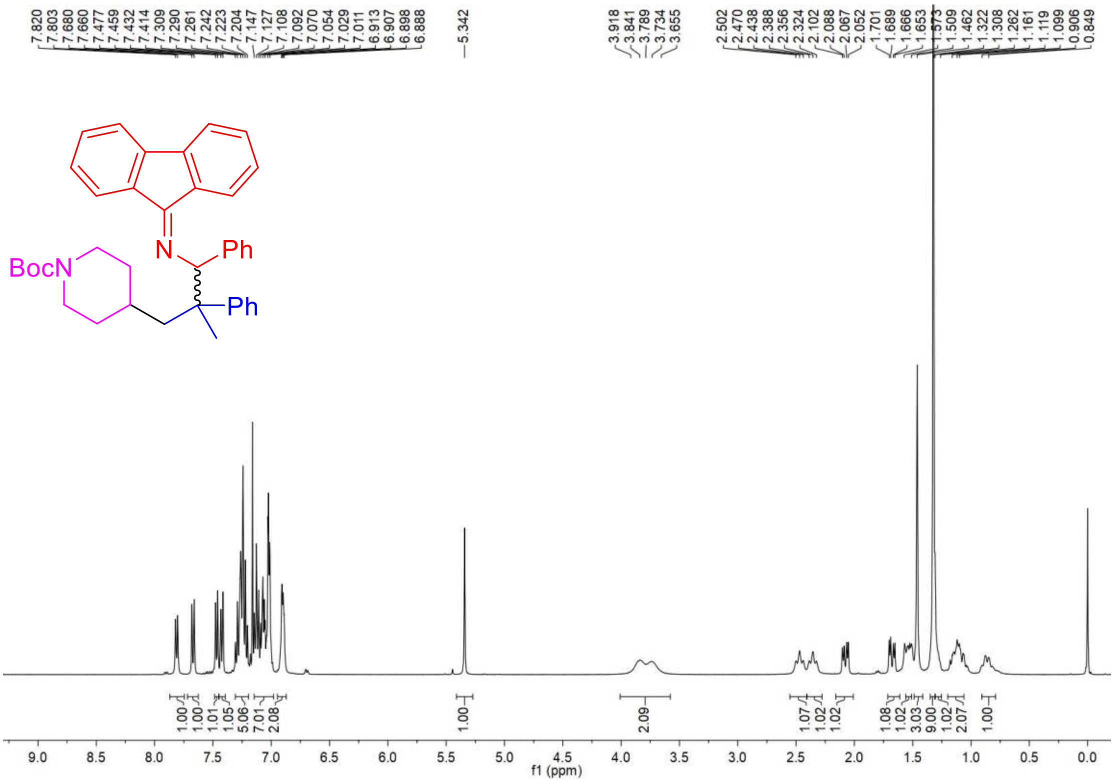

Figure S61. **^{13}C{^{1}H} NMR spectra (100 MHz, Chloroform-*d*) of *tert*-Butyl 4-(3-((9*H*-fluoren-9-ylidene)amino)-2-methyl-2,3-diphenylpro-pyl)piperidine-1-carboxylate (4mv'')**(minor)**).**

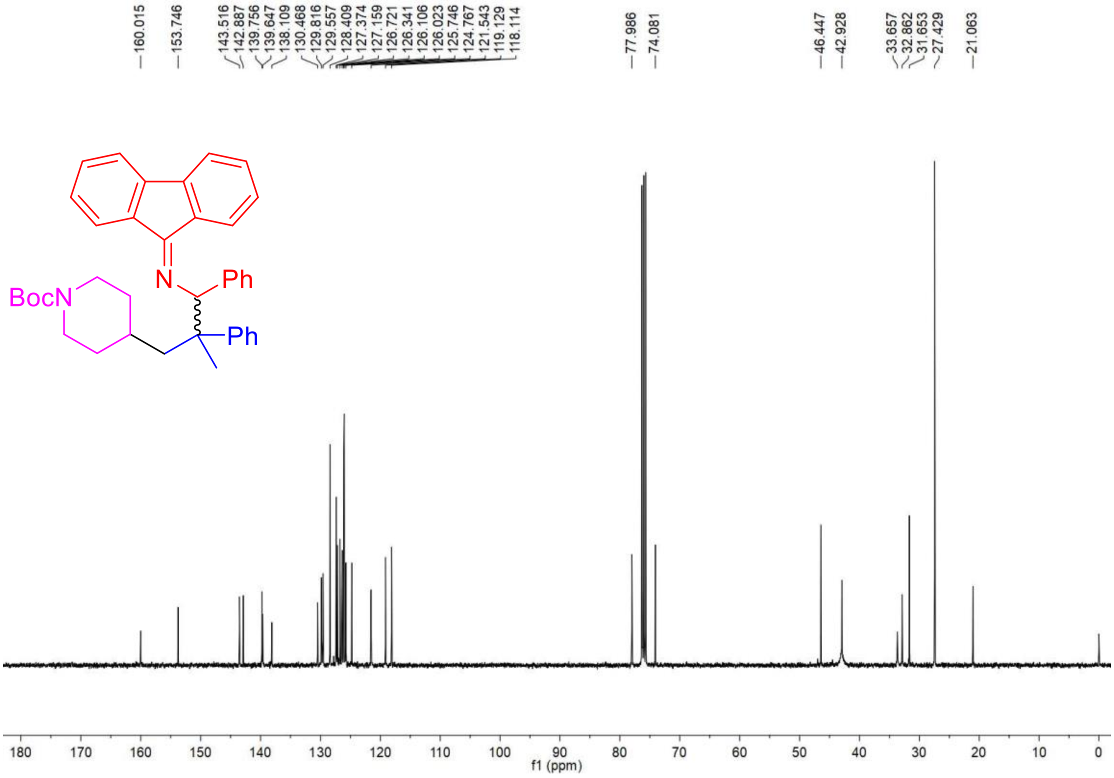

Figure S62. ^{1}H **NMR spectra (600 MHz, Chloroform-*d*) of *N*-((1-(((3r,5r,7r)-Adamantan-1-yl)methyl)-4-phenylcyclohexyl)(phenyl)methyl)-9*H*-fluoren-9-imine (4aw).**

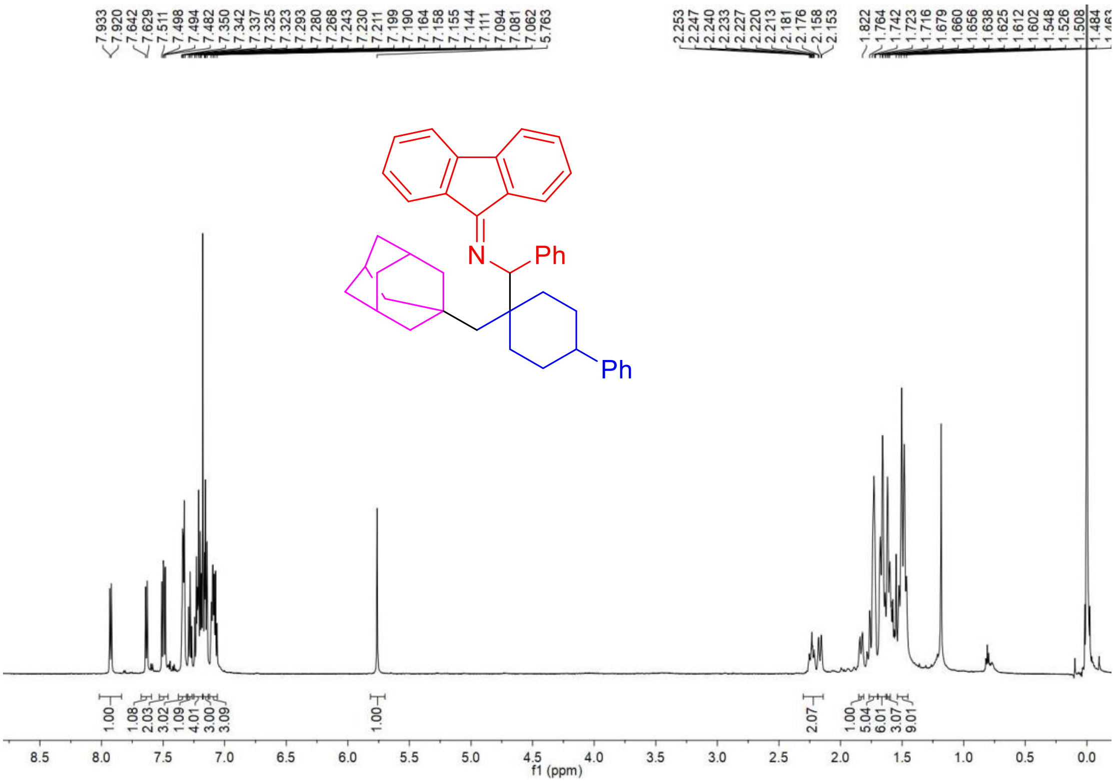

Figure S63. $^{13}C\{^{1}H\}$ **NMR spectra (150 MHz, Chloroform-*d*) of *N*-((1-(((3r,5r,7r)-Adamantan-1-yl)methyl)-4-phenylcyclohexyl)(phenyl)methyl)-9*H*-fluoren-9-imine (4aw).**

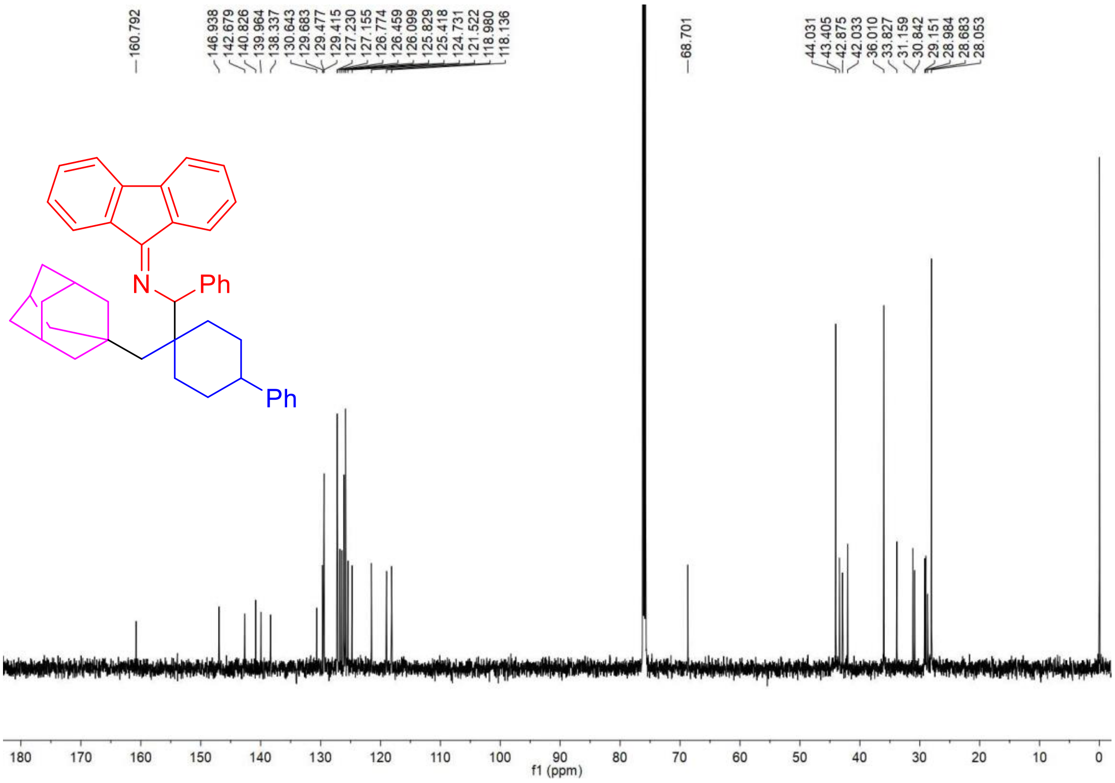

Figure S64. ^{1}**H NMR spectra (400 MHz, Chloroform-*d*) of *N*-(3-Methyl-1,2,2-triphenylbutyl)-9H-fluoren-9-imine (4ux).**

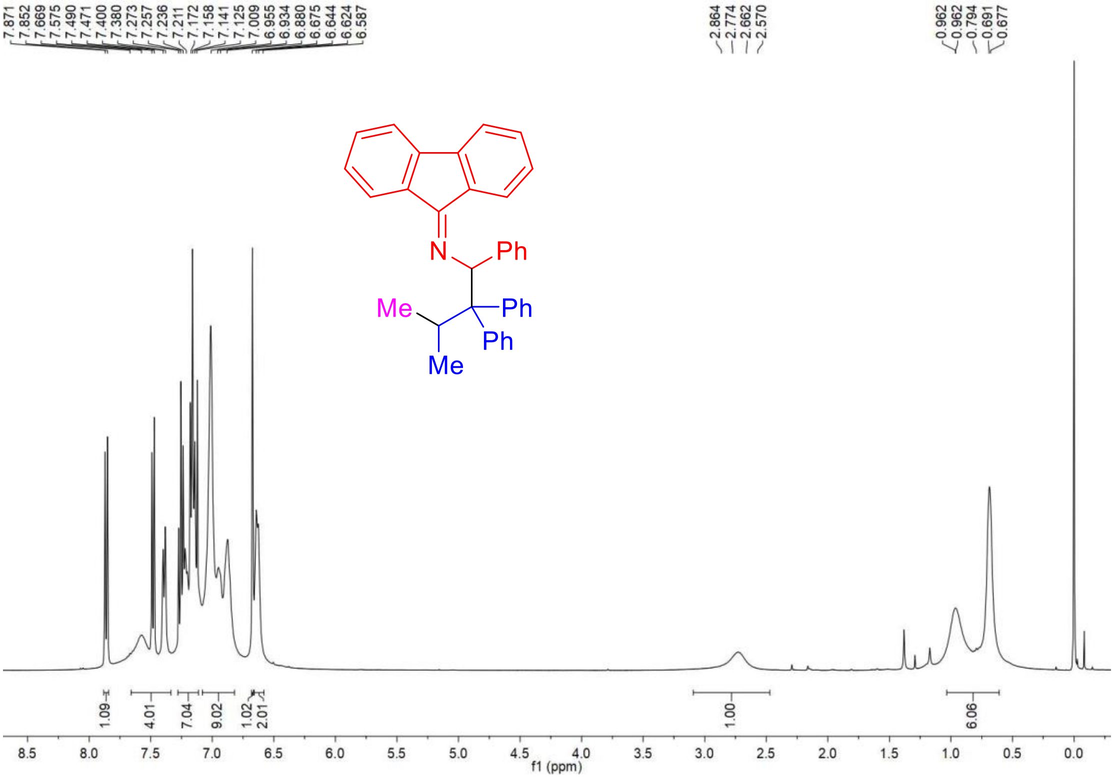

Figure S65. ^{13}**C{**^{1}**H} NMR spectra (100 MHz, Chloroform-*d*) of *N*-(3-Methyl-1,2,2-triphenylbutyl)-9H-fluoren-9-imine (4ux).**

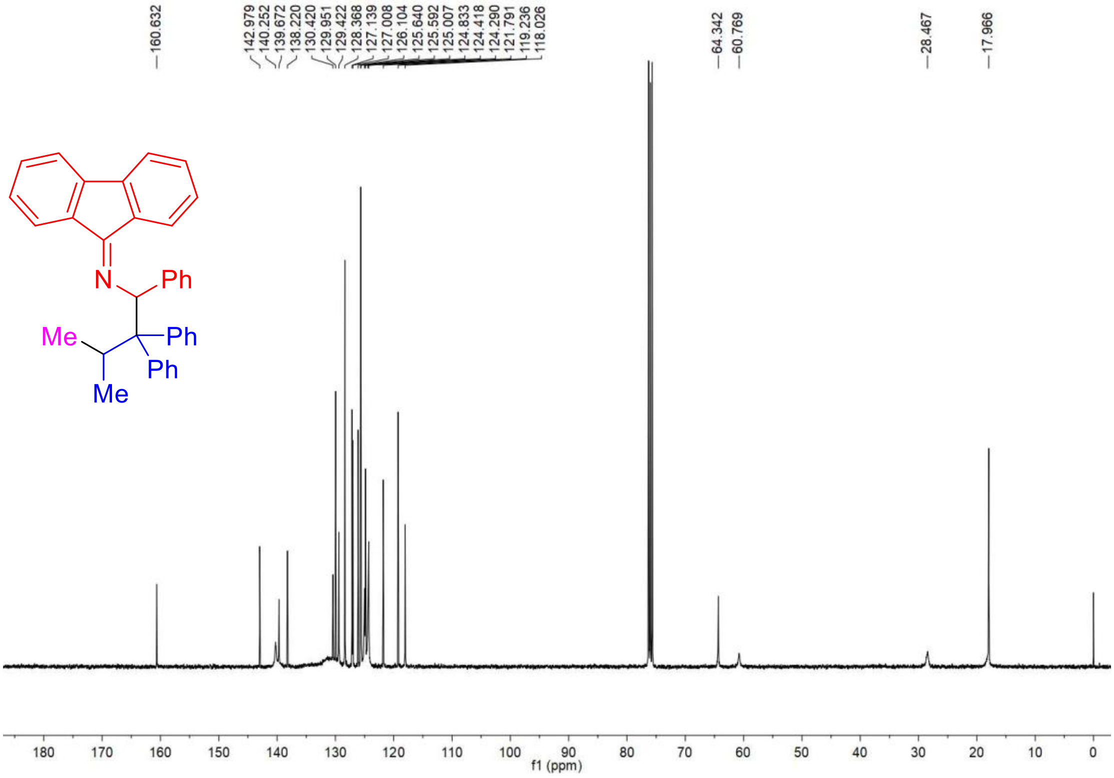

Figure S66. ^{1}**H NMR spectra (600 MHz, Chloroform-*d*) of 3-Methyl-1,2,2-triphenylpentan-1-amine (4vx,** dr = 2.3:1**).**

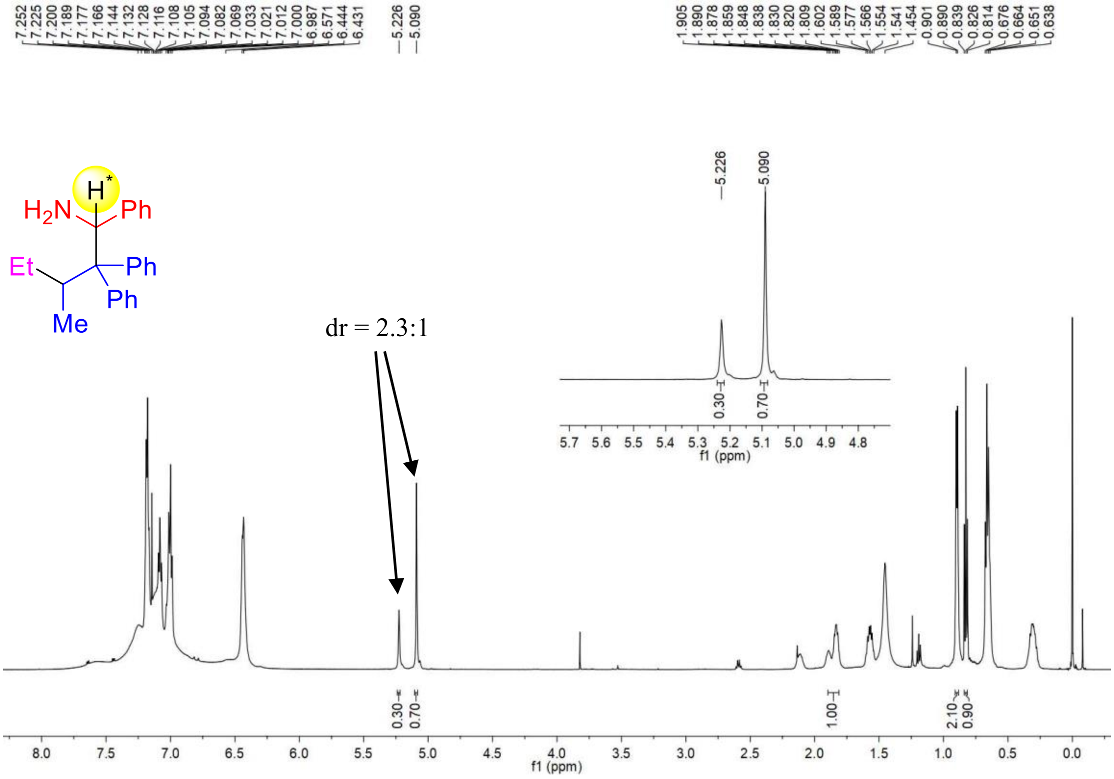

Figure S67. ^{13}**C{**^{1}**H} NMR spectra (150 MHz, Chloroform-*d*) of 3-Methyl-1,2,2-triphenylpentan-1-amine (4vx,** dr = 2.3:1**).**

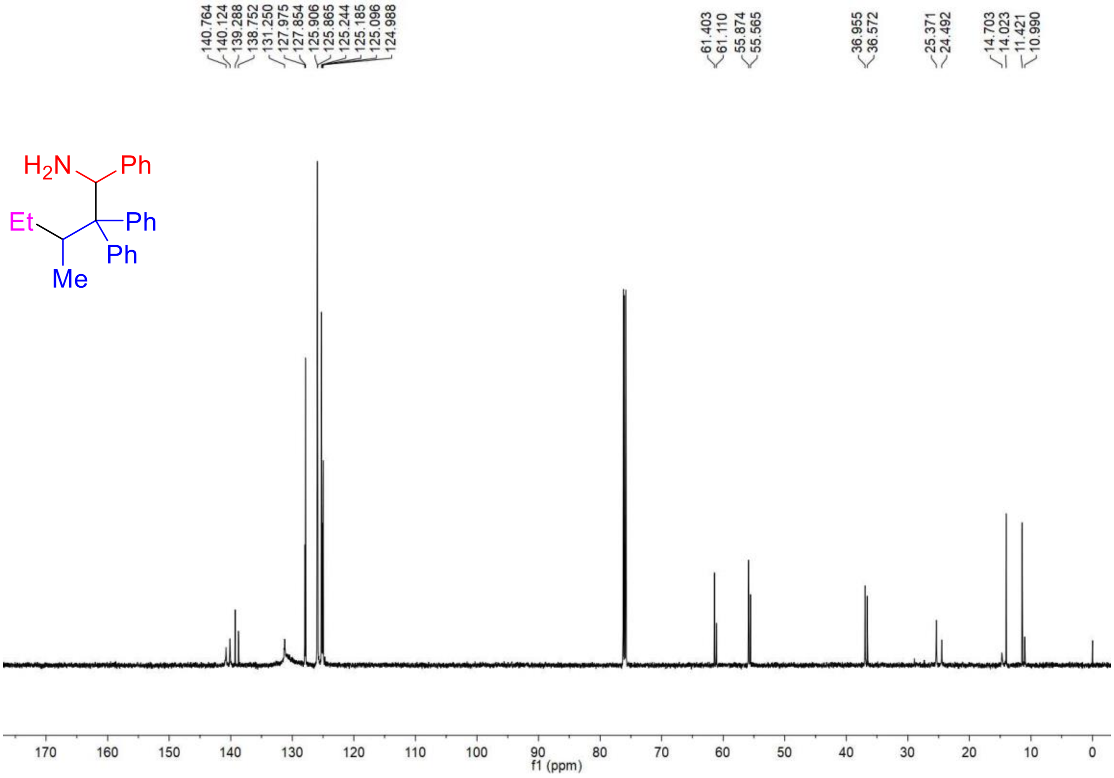

Figure S68. ^{1}H NMR spectra (400 MHz, Chloroform-*d*) of *tert*-Butyl 4-(3-((9*H*-fluoren-9-ylidene)amino)-3-(4-fluorophenyl)-2,2-diphenylpropyl)piperidine-1-carboxylate (4bm).

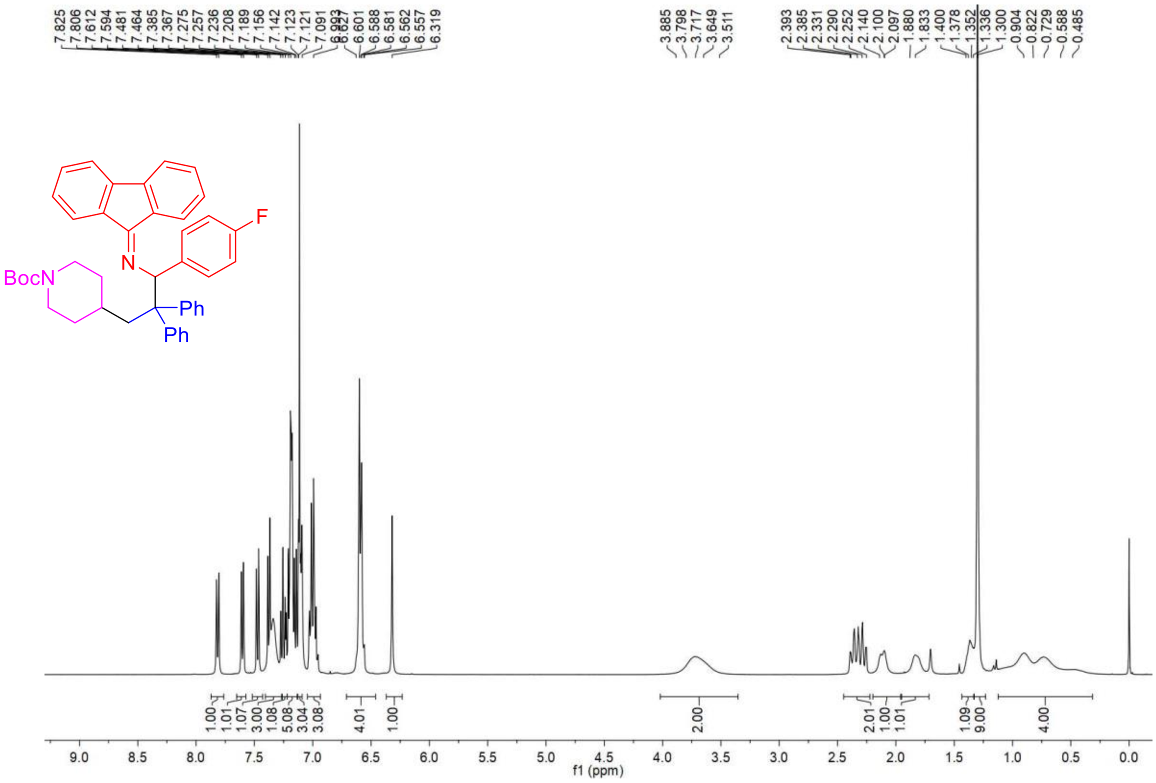

Figure S69. $^{13}C\{^{1}H\}$ NMR spectra (100 MHz, Chloroform-*d*) of *tert*-Butyl 4-(3-((9*H*-fluoren-9-ylidene)amino)-3-(4-fluorophenyl)-2,2-diphenylpropyl)piperidine-1-carboxylate (4bm).

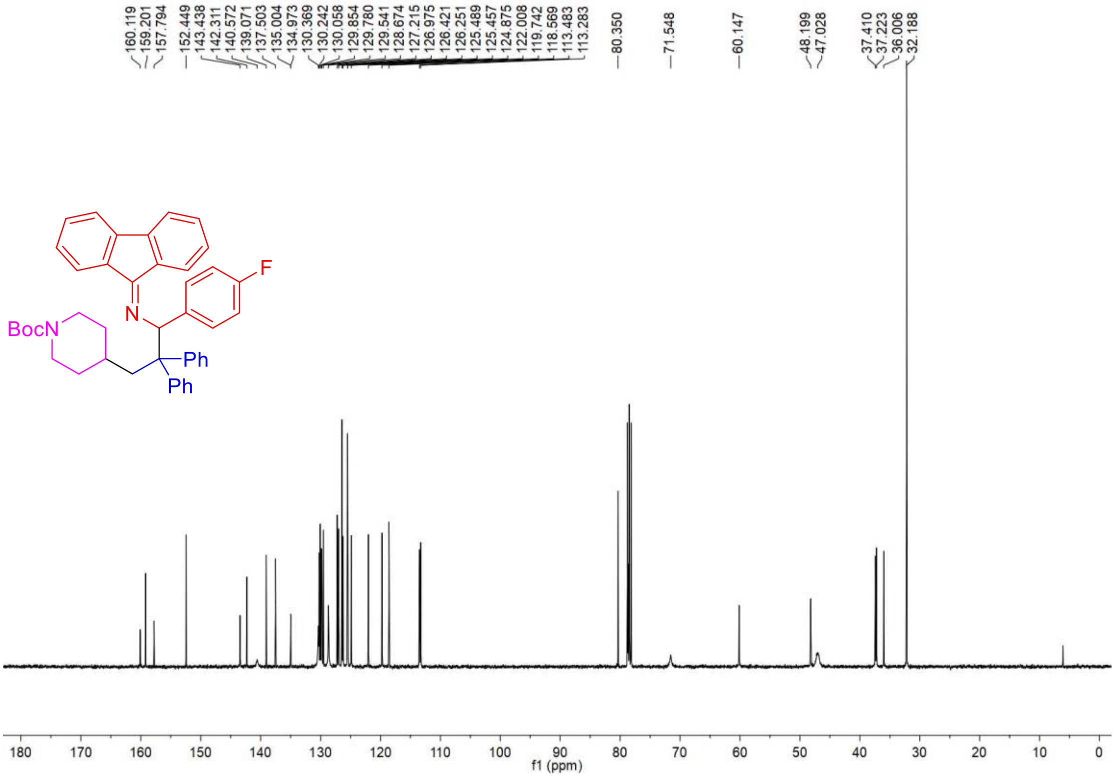

Figure S70. **^{19}F NMR spectra (376 MHz, Chloroform-*d*) of *tert*-Butyl 4-(3-((9*H*-fluoren-9-ylidene)amino)-3-(4-fluorophenyl)-2,2-diphenylpropyl)piperidine-1-carboxylate (4bm).**

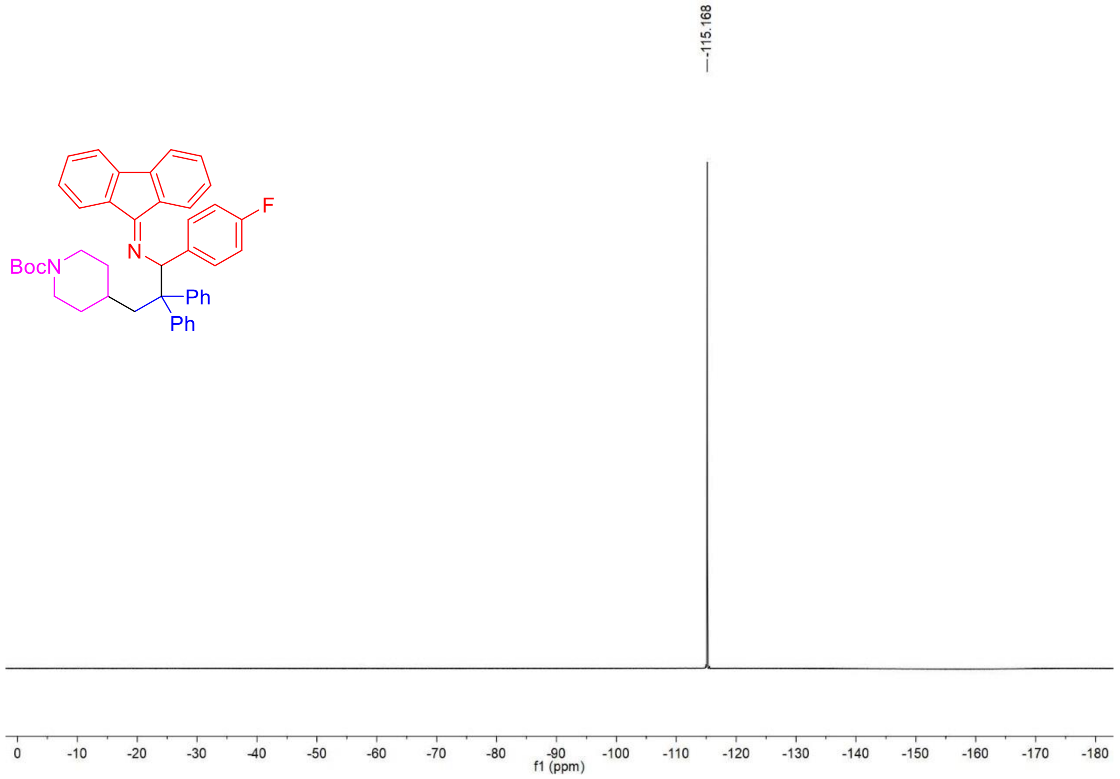

Figure S71. **^{1}H NMR spectra (400 MHz, Chloroform-*d*) of *tert*-Butyl 4-(3-((9*H*-fluoren-9-ylidene)amino)-3-(4-chlorophenyl)-2, 2-diphenylpropyl)piperidine-1-carboxylate (4cm).**

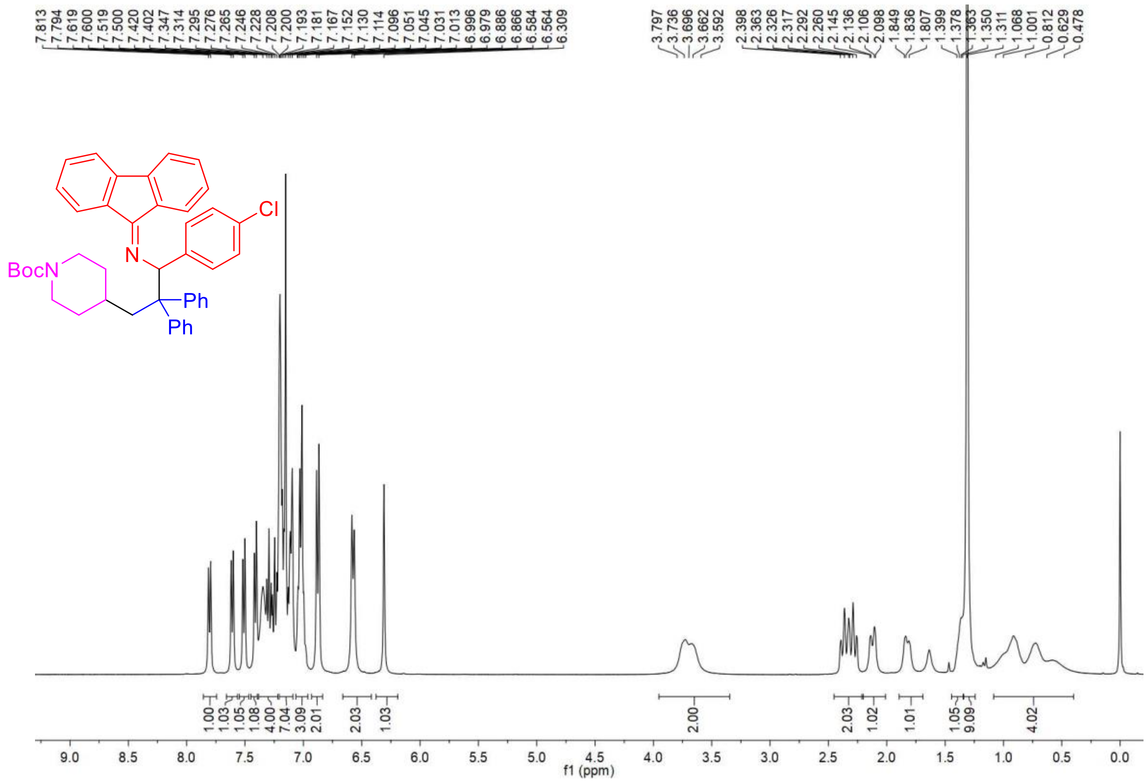

Figure S72. **^{13}C{^{1}H} NMR spectra (100 MHz, Chloroform-*d*) of *tert*-Butyl 4-(3-((9*H*-fluoren-9-ylidene)amino)-3-(4-chlorophenyl)-2, 2-diphenylpropyl)piperidine-1-carboxylate (4cm).**

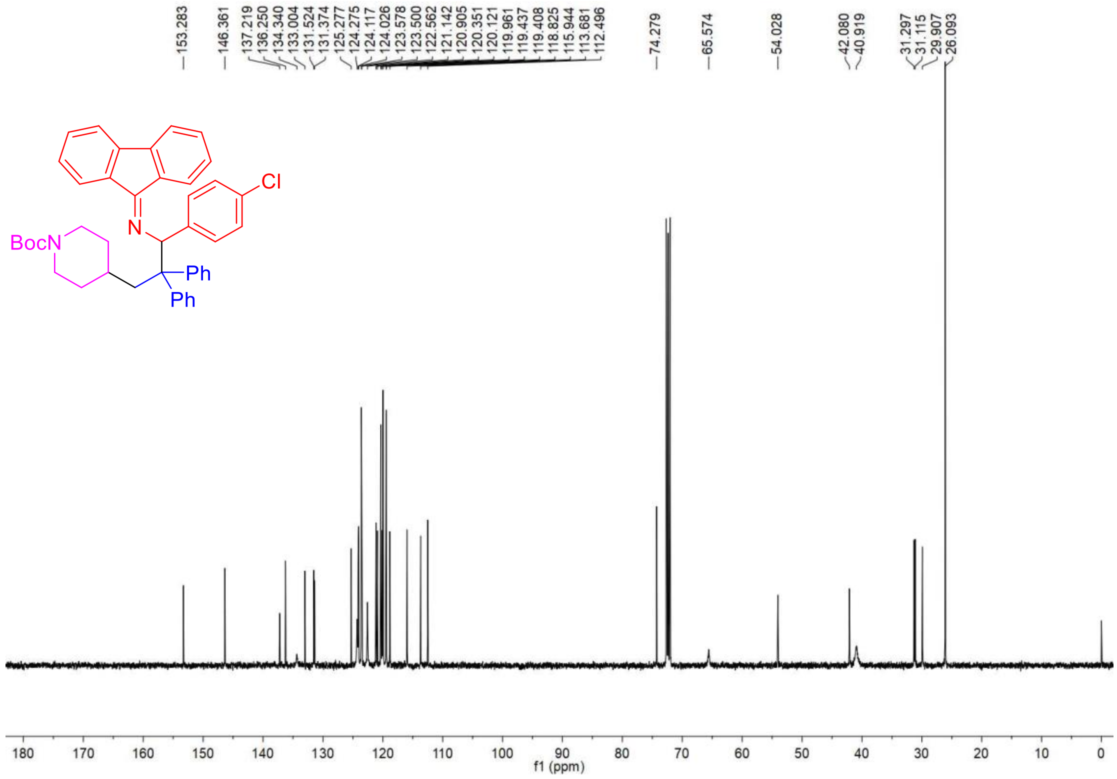

Figure S73. ^{1}H NMR spectra (400 MHz, Chloroform-*d*) of *tert*-Butyl 4-(3-((9*H*-fluoren-9-ylidene)amino)-2,2-diphenyl-3-(*p*-tolyl) propyl)piperidine-1-carbox-ylate (4dm).

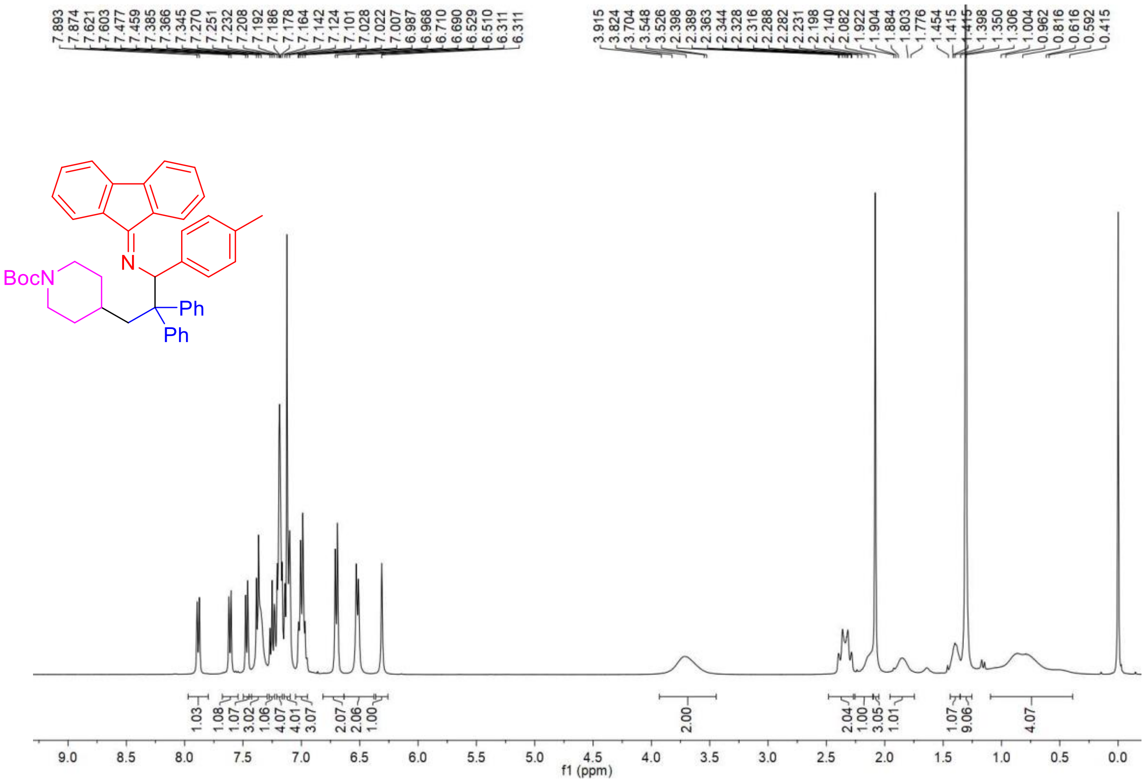

Figure S74. ^{13}C{^{1}H} NMR spectra (100 MHz, Chloroform-*d*) of *tert*-Butyl 4-(3-((9*H*-fluoren-9-ylidene)amino)-2,2-diphenyl-3-(*p*-tolyl) propyl)piperidine-1-carbox-ylate (4dm).

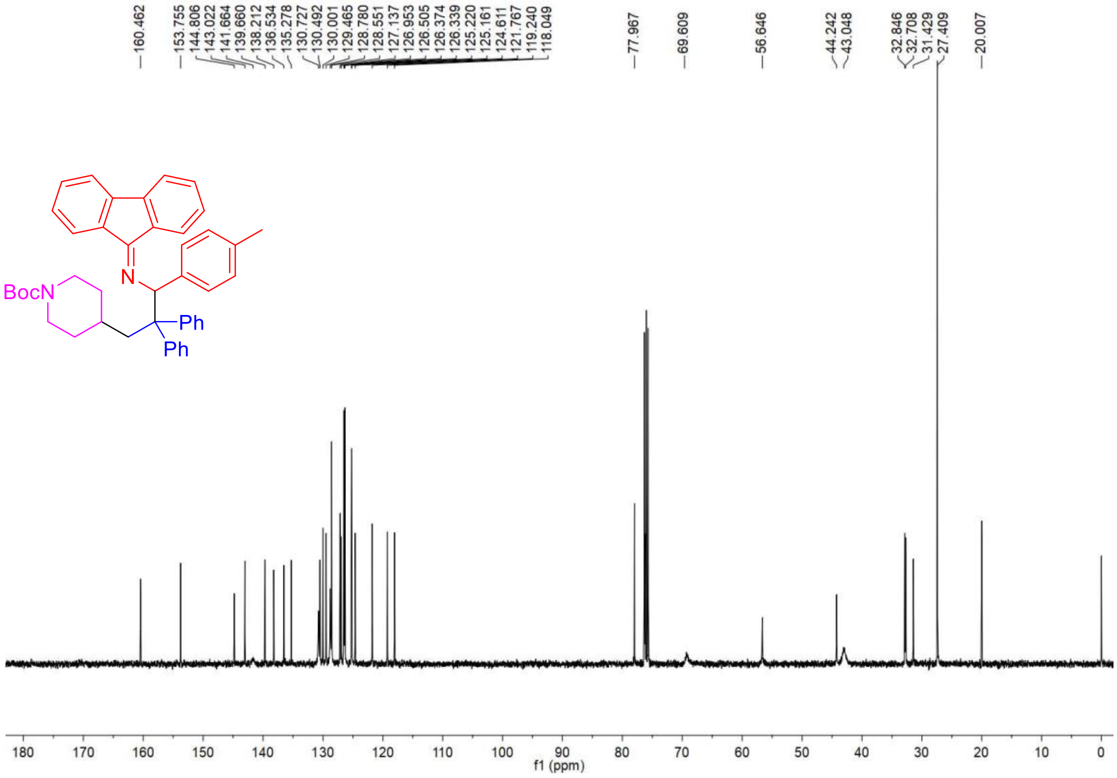

Figure S75. ^{1}H NMR spectra (400 MHz, Chloroform-*d*) of *tert*-Butyl 4-(3-((9*H*-fluoren-9-ylidene)amino)-3-(4-(*tert*-butyl) phenyl)-2,2-diphenylpropyl)piperi-dine-1-carboxylate (4em).

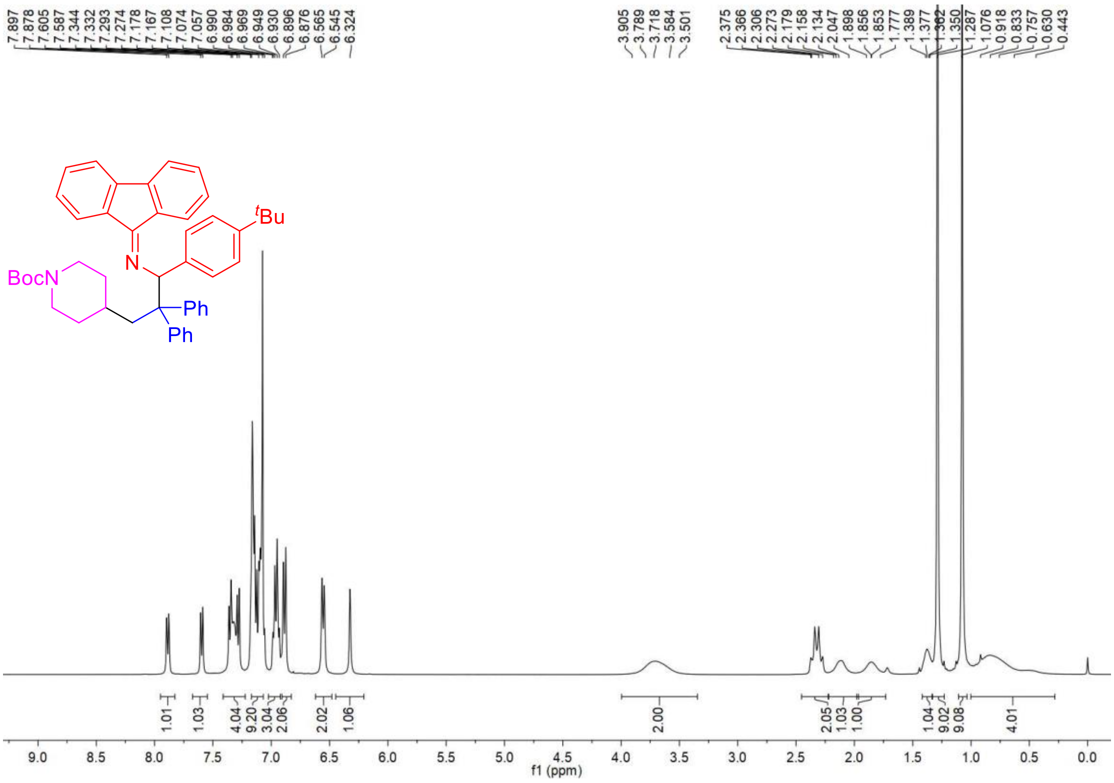

Figure S76. $^{13}C\{^{1}H\}$ NMR spectra (100 MHz, Chloroform-*d*) of *tert*-Butyl 4-(3-((9*H*-fluoren-9-ylidene)amino)-3-(4-(*tert*-butyl) phenyl)-2,2-diphenylpropyl)piperi-dine-1-carboxylate (4em).

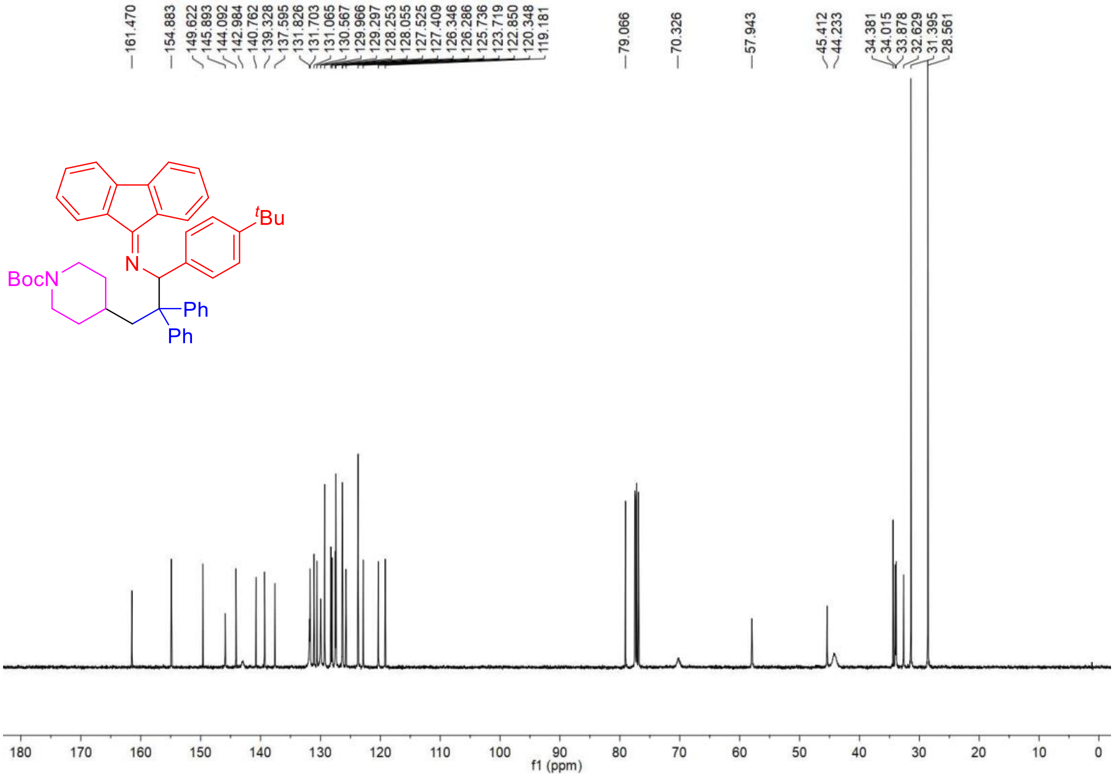

Figure S77. ^{1}H **NMR spectra (400 MHz, Chloroform-*d*) of *tert*-Butyl 4-(3-((9*H*-fluoren-9-ylidene)amino)-3-(3,4-dimethoxy-phenyl)-2,2-diphenylpropyl)piperi-dine-1-carboxylate (4fm).**

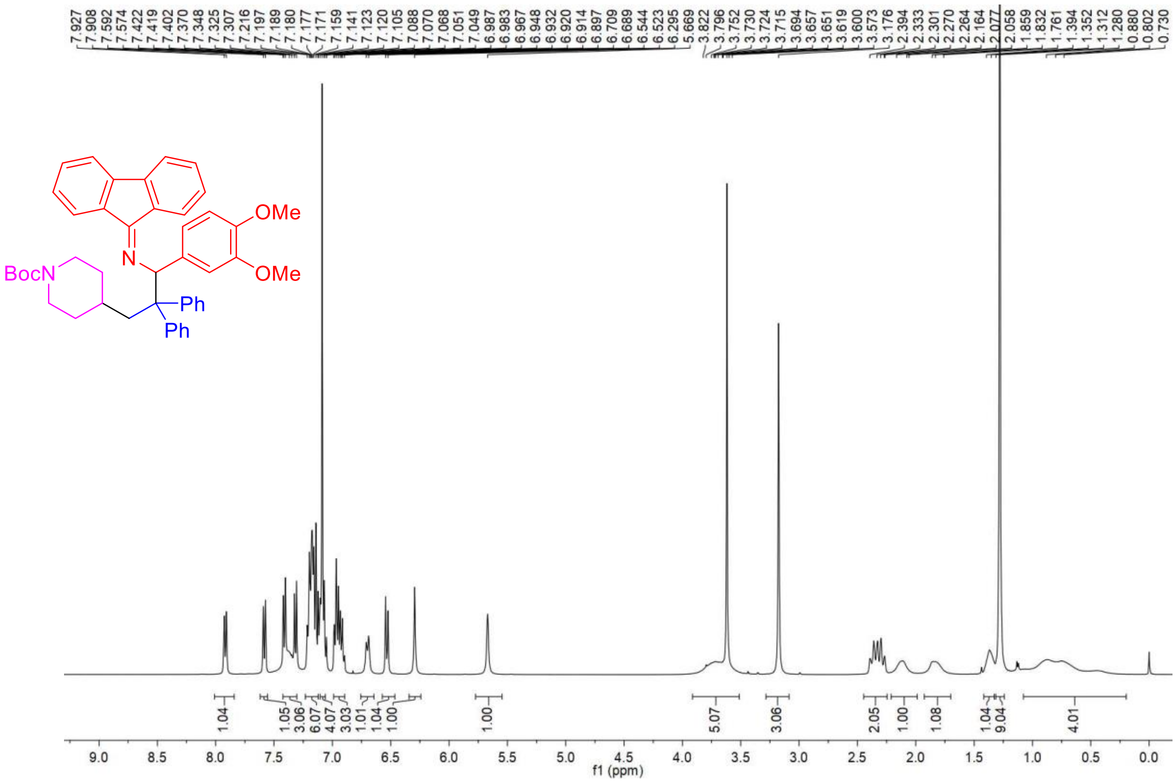

Figure S78. ^{13}C{^{1}H} **NMR spectra (100 MHz, Chloroform-*d*) of *tert*-Butyl 4-(3-((9*H*-fluoren-9-ylidene)amino)-3-(3,4-dimethoxy-phenyl)-2,2-diphenylpropyl)piperi-dine-1-carboxylate (4fm).**

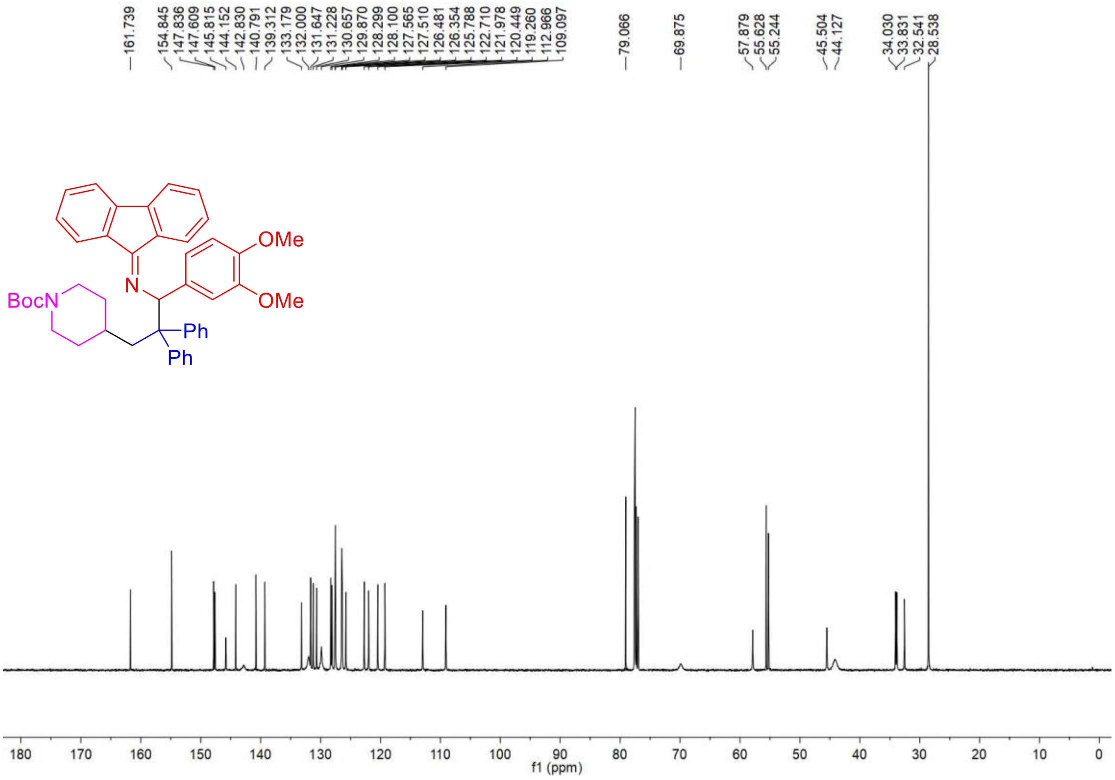

Figure S79. **^{1}H NMR spectra (400 MHz, Chloroform-*d*) of *tert*-Butyl 4-(3-((9*H*-fluoren-9-ylidene)amino)-3-([1,1'-biphenyl]-4- yl)-2,2-diphenylpropyl)piperidine-1-carboxylate (4gm).**

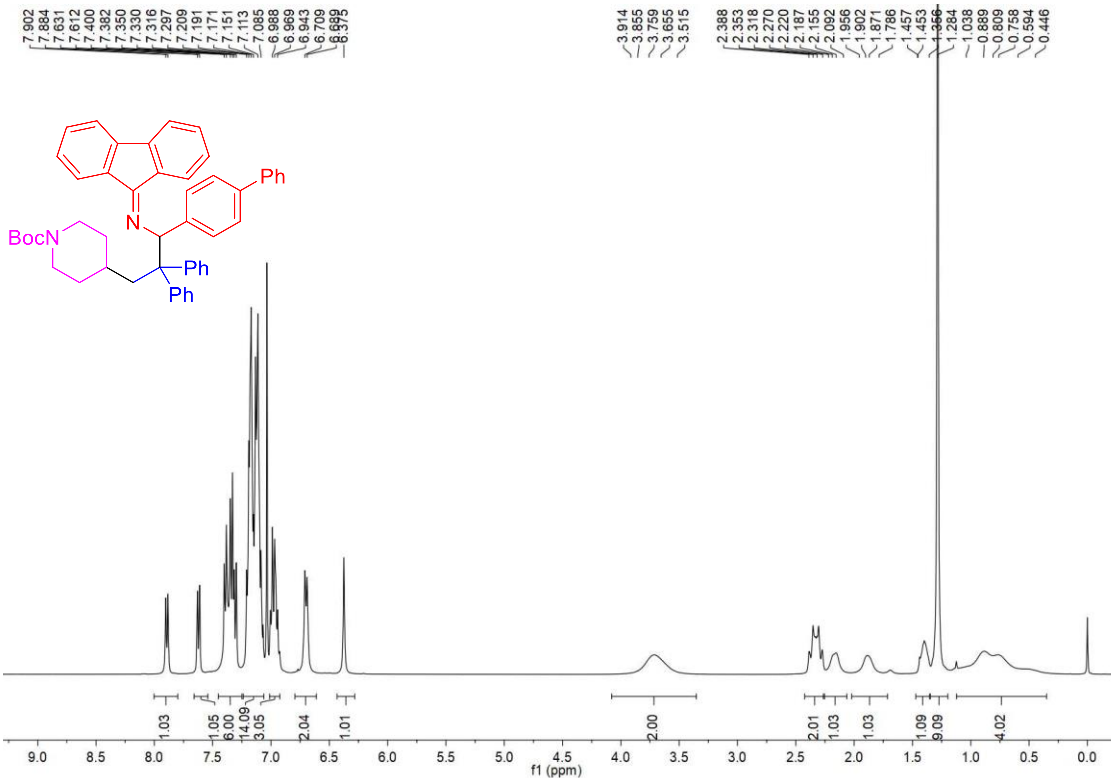

Figure S80. **^{13}C{^{1}H} NMR spectra (100 MHz, Chloroform-*d*) of *tert*-Butyl 4-(3-((9*H*-fluoren-9-ylidene)amino)-3-([1,1'-biphenyl]-4- yl)-2,2-diphenylpropyl)piperidine-1-carboxylate (4gm).**

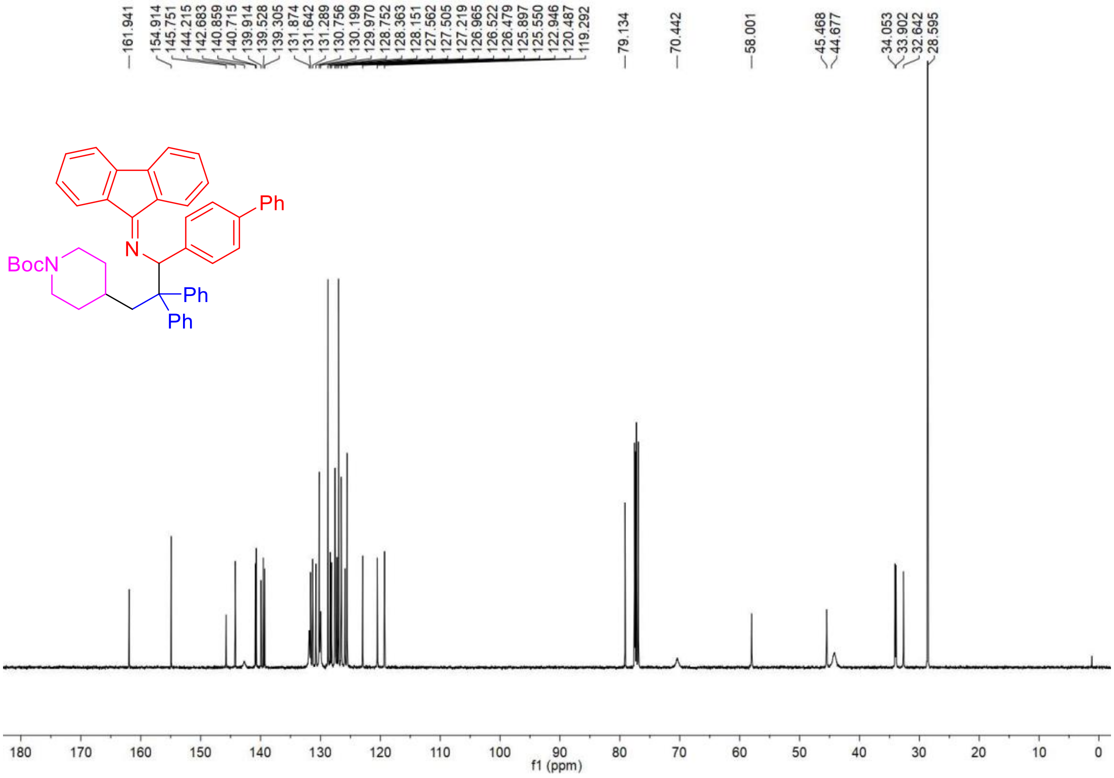

Figure S81. ^{1}**H NMR spectra (400 MHz, Chloroform-*d*) of *tert*-Butyl 4-(3-((9*H*-fluoren-9-ylidene)amino)-2,2-diphenyl-3-(*o*-tolyl)propyl)piperidine-1-carboxylate (4hm).**

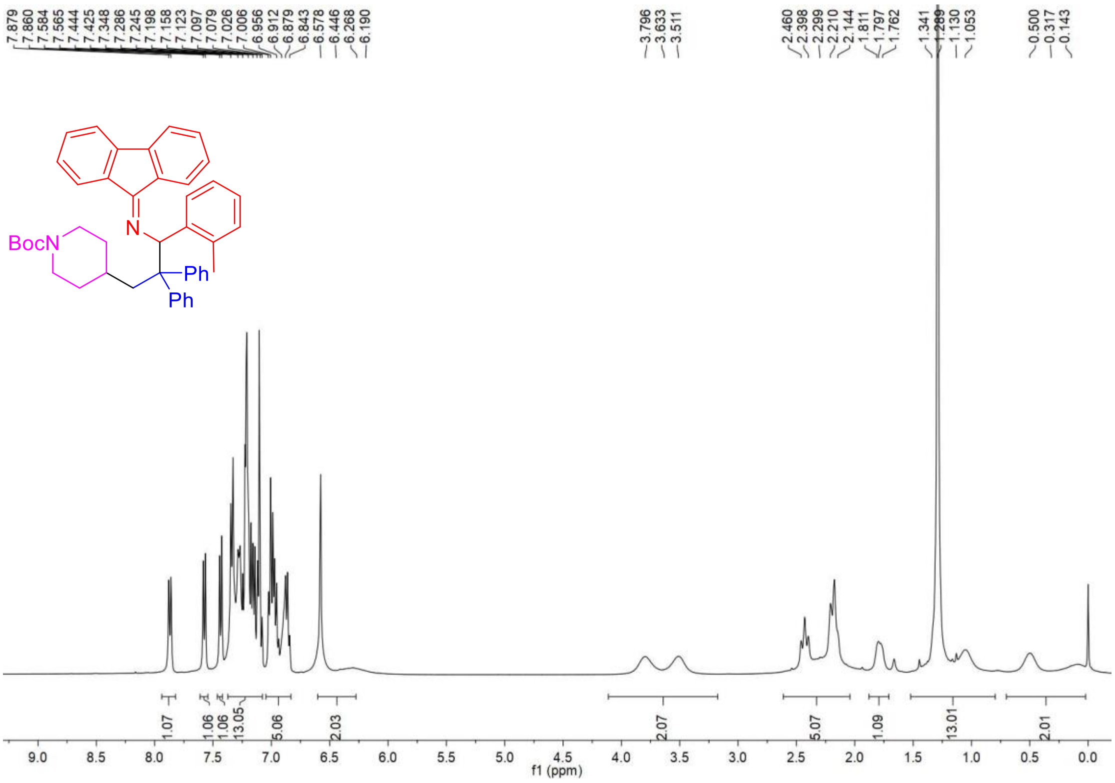

Figure S82. ^{13}**C{**^{1}**H} NMR spectra (100 MHz, Chloroform-*d*) of *tert*-Butyl 4-(3-((9*H*-fluoren-9-ylidene)amino)-2,2-diphenyl-3-(*o*-tolyl)propyl)piperidine-1-carboxylate (4hm).**

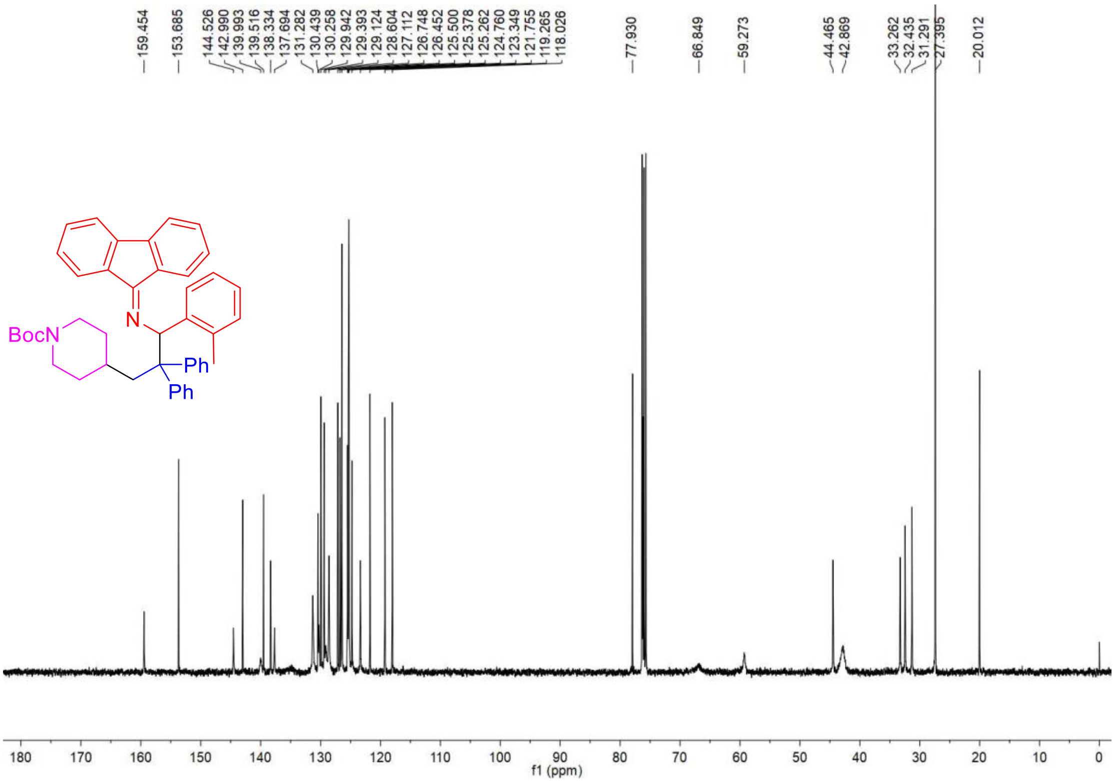

Figure S83. **^{1}H NMR spectra (400 MHz, Chloroform-*d*) of *tert*-Butyl 4-(3-((9*H*-fluoren-9-ylidene)amino)-3-(naphthalen-1- yl)-2,2-diphenylpropyl)piperidine-1-carboxylate (4im).**

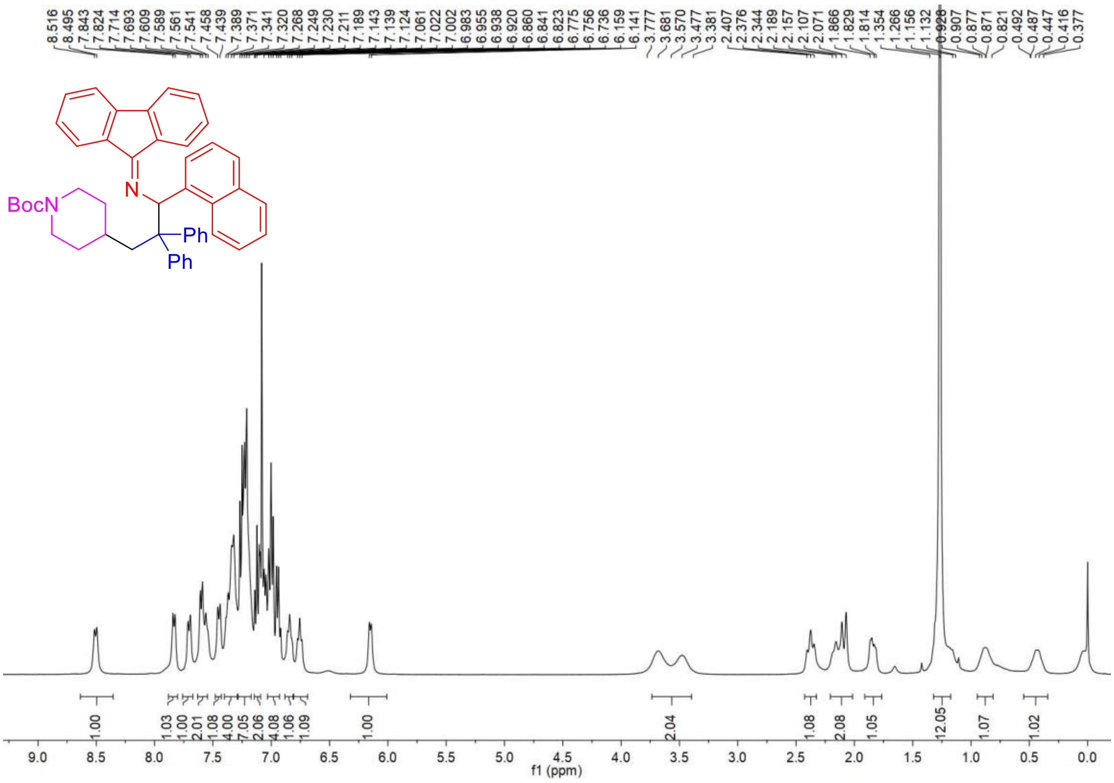

Figure S84. **^{13}C{^{1}H} NMR spectra (100 MHz, Chloroform-*d*) of *tert*-Butyl 4-(3-((9*H*-fluoren-9-ylidene)amino)-3-(naphthalen-1- yl)-2,2-diphenylpropyl)piperidine-1-carboxylate (4im).**

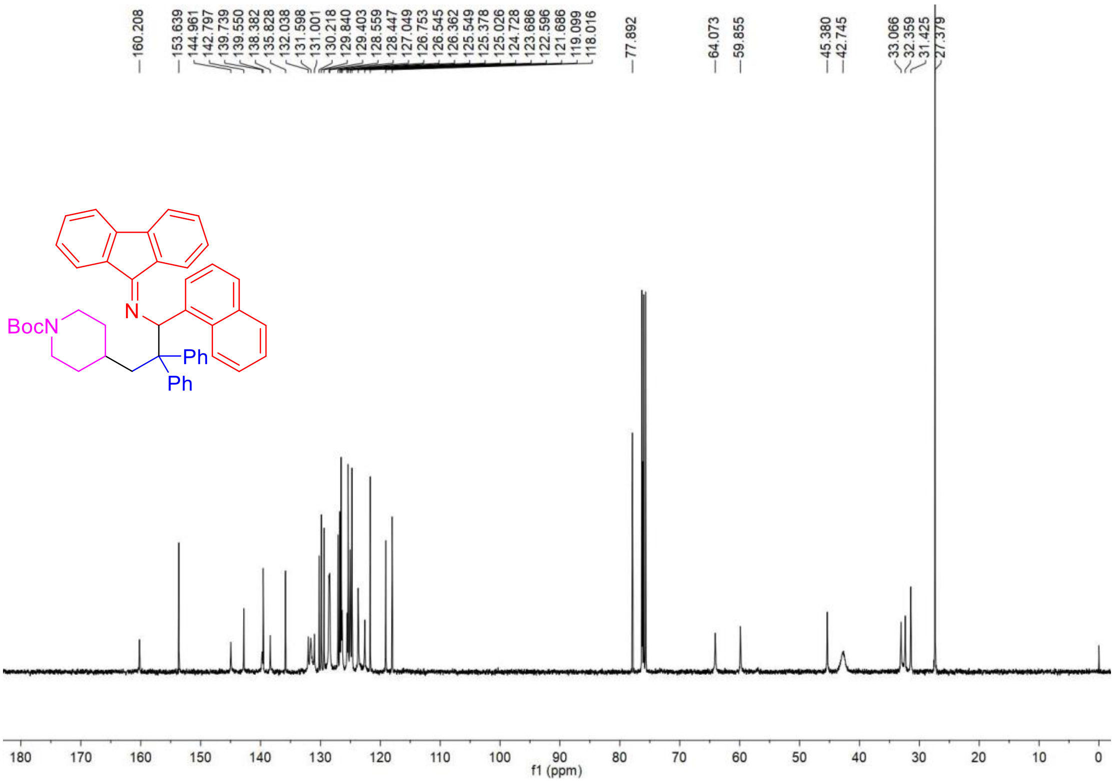

Figure S85. **^{1}H NMR spectra (400 MHz, Chloroform-*d*) of *tert*-Butyl 4-(3-((9*H*-fluoren-9-ylidene)amino)-3-(2,3-dihydrobenzo-furan-6-yl)-2,2-diphenylpropyl)piperidine-1-carboxylate (4jm).**

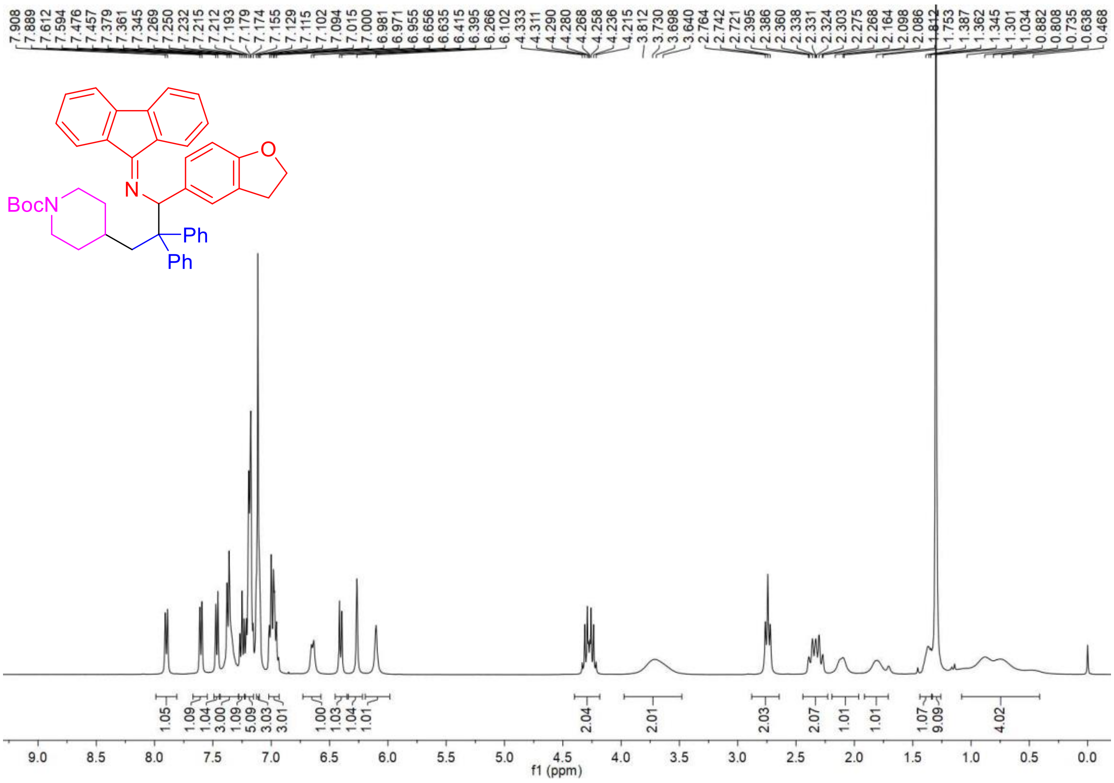

Figure S86. **^{13}C{^{1}H} NMR spectra (100 MHz, Chloroform-*d*) of *tert*-Butyl 4-(3-((9*H*-fluoren-9-ylidene)amino)-3-(2,3-dihydrobenzo-furan-6-yl)-2,2-diphenylpropyl)piperidine-1-carboxylate (4jm).**

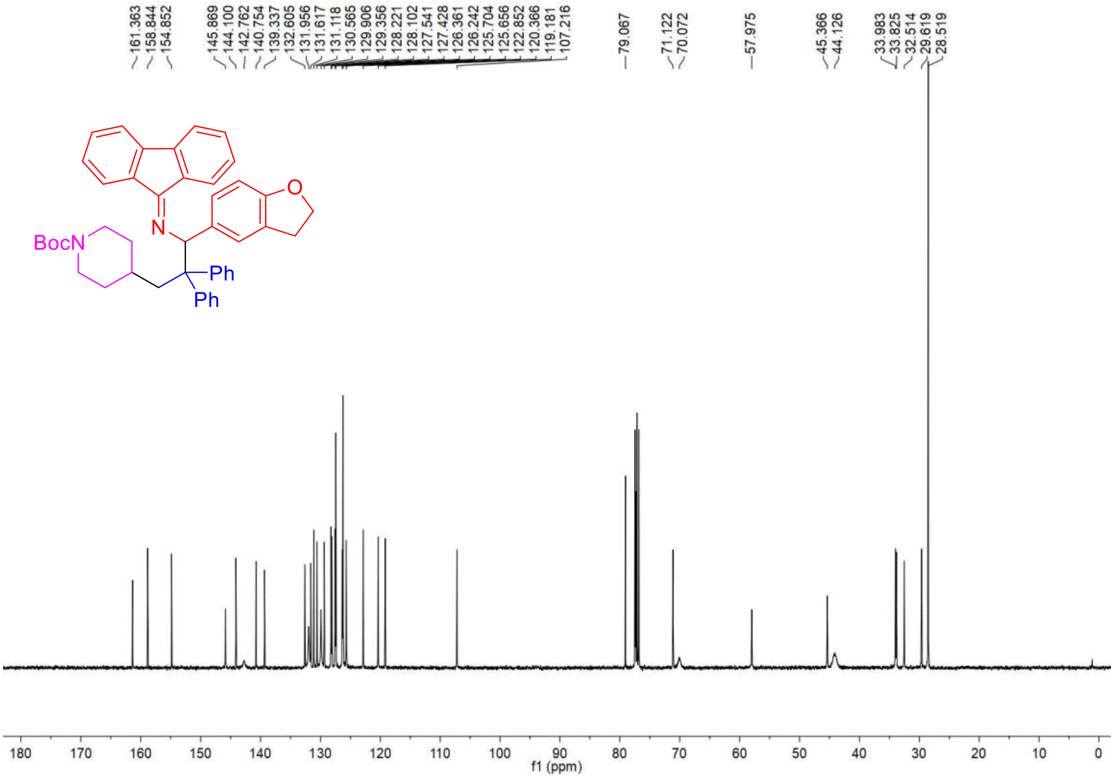

Figure S87. ^{1}H **NMR spectra (400 MHz, Chloroform-*d*) of *tert*-Butyl 4-(3-((9*H*-fluoren-9-ylidene)amino)-3-(furan-3-yl)-2,2- diphenylpropyl)piperidine-1-car-boxylate (4km).**

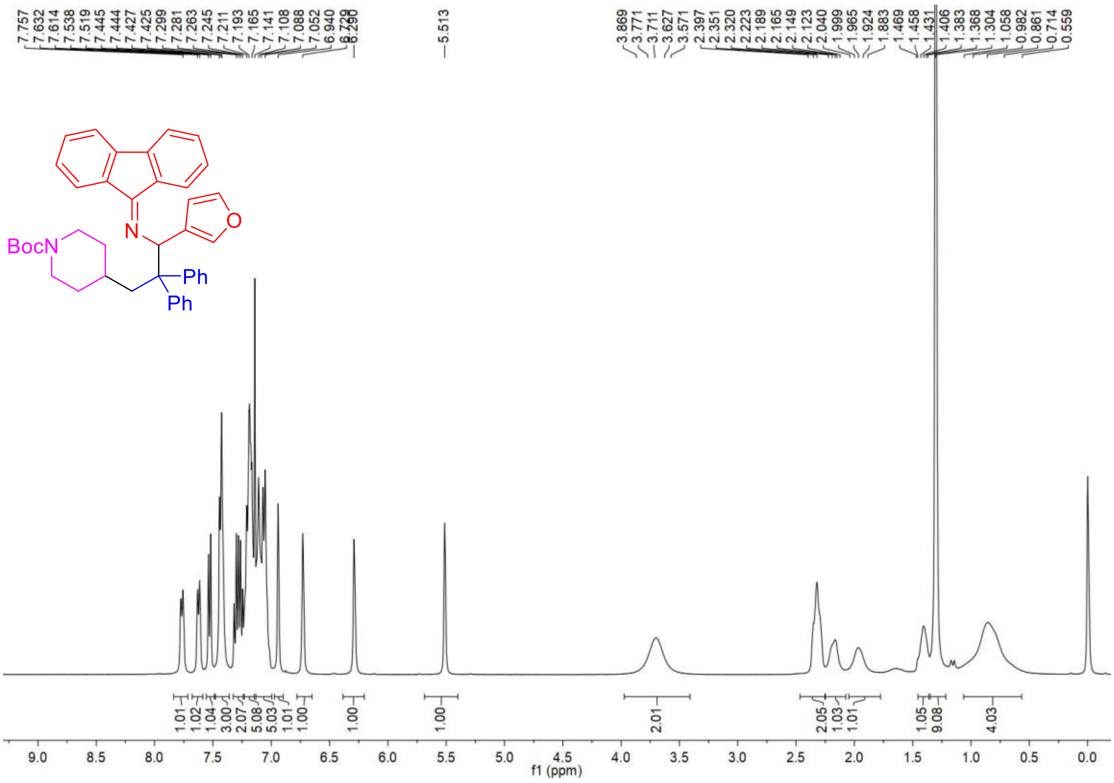

Figure S88. ^{13}C{^{1}H} **NMR spectra (100 MHz, Chloroform-*d*) of *tert*-Butyl 4-(3-((9*H*-fluoren-9-ylidene)amino)-3-(furan-3-yl)-2,2- diphenylpropyl)piperidine-1-car-boxylate (4km).**

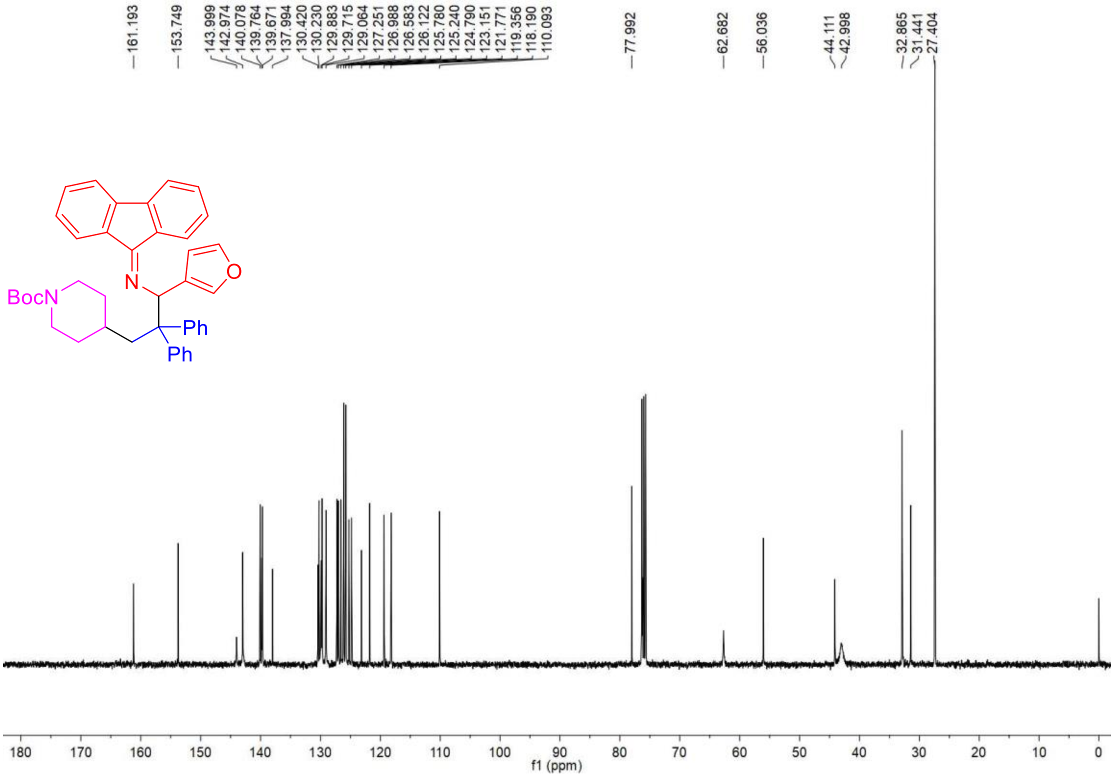

Figure S89. ^{1}**H NMR spectra (400 MHz, Chloroform-*d*) of *tert*-Butyl 4-(3-((9*H*-fluoren-9-ylidene)amino)-3-(2,6-dime-thoxypyridin-3-yl)-2,2-diphenylpropyl)piperidine-1-carboxylate (4lm).**

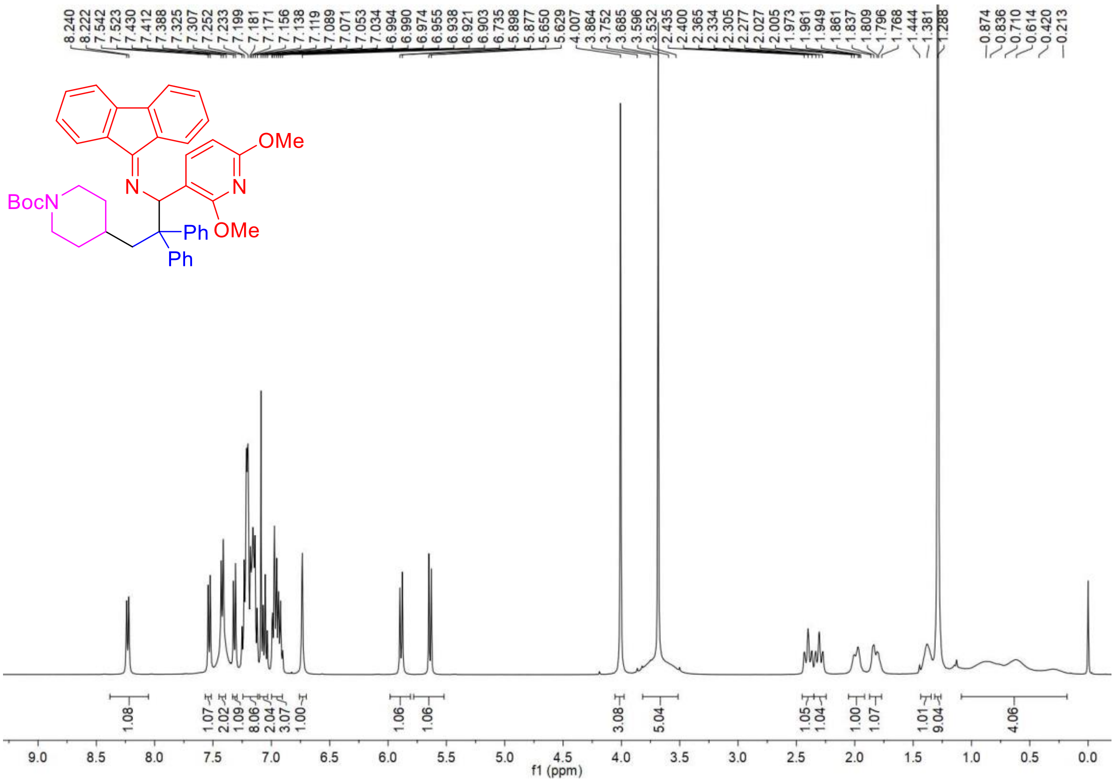

Figure S90. ^{13}**C{**^{1}**H} NMR spectra (100 MHz, Chloroform-*d*) of *tert*-Butyl 4-(3-((9*H*-fluoren-9-ylidene)amino)-3-(2,6-dime-thoxypyridin-3-yl)-2,2-diphenylpropyl)piperidine-1-carboxylate (4lm).**

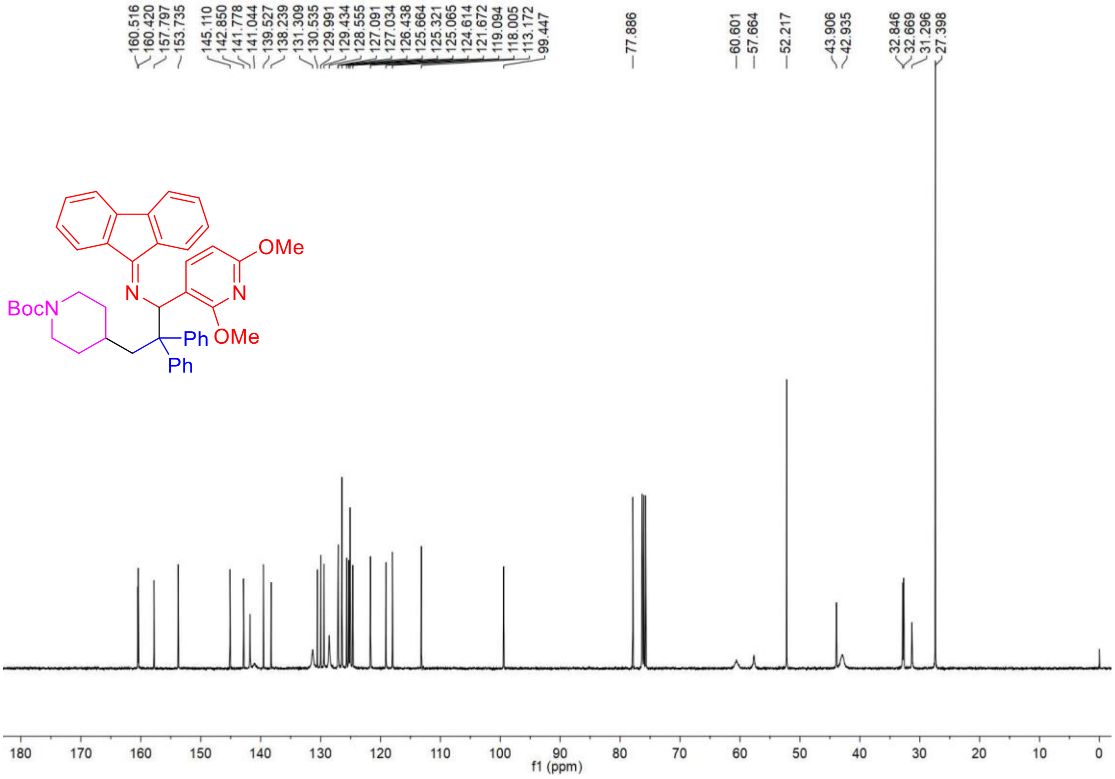

Figure S91. **^{1}H NMR spectra (400 MHz, Chloroform-*d*) of *tert*-Butyl 4-(3-((9*H*-fluoren-9-ylidene)amino)-2,2-diphenylbutyl)piperidine-1-carboxylate (4mm).**

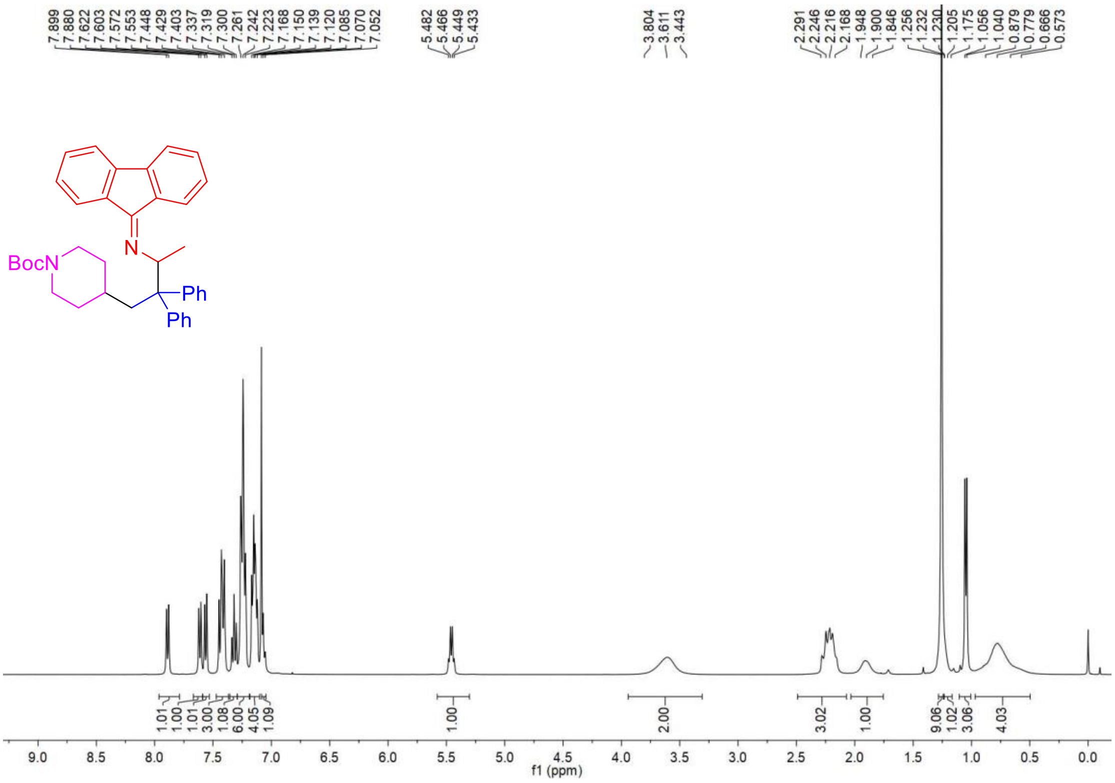

Figure S92. **^{13}C{^{1}H} NMR spectra (100 MHz, Chloroform-*d*) of *tert*-Butyl 4-(3-((9*H*-fluoren-9-ylidene)amino)-2,2-diphenylbutyl)piperidine-1-carboxylate (4mm).**

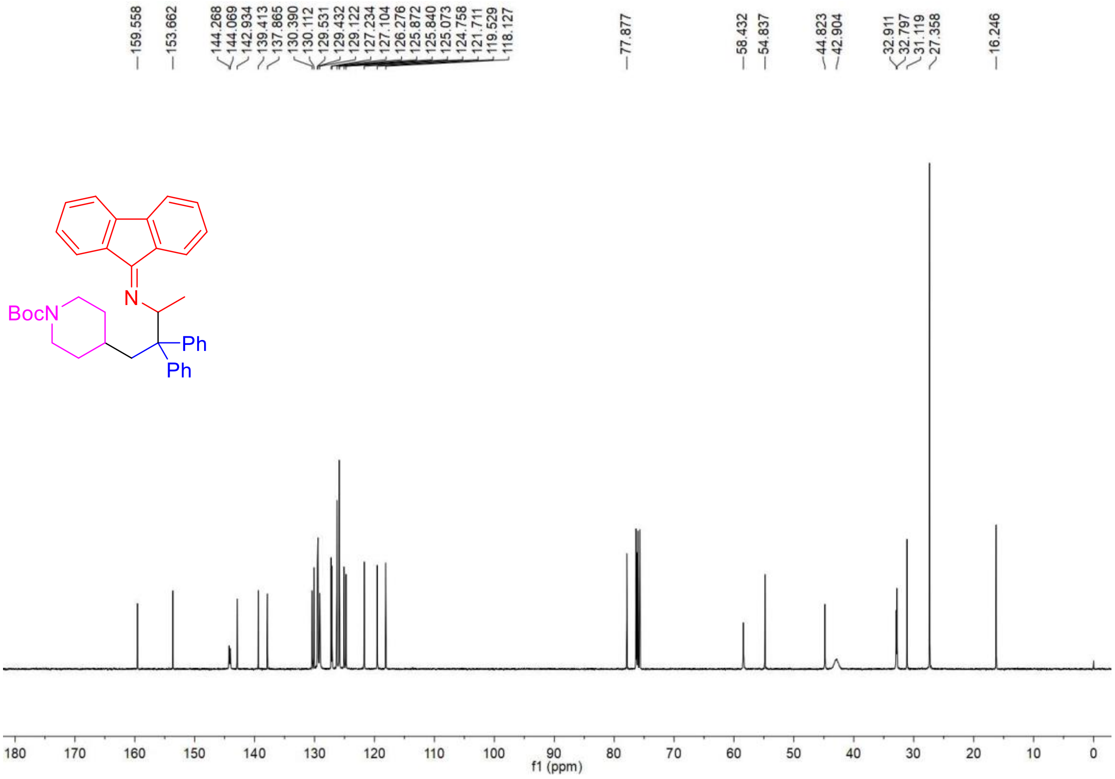

Figure S93. **^{1}H NMR spectra (400 MHz, Chloroform-*d*) of *tert*-Butyl 4-(3-((9*H*-fluoren-9-ylidene)amino)-5-methyl-2,2-diphenylhexyl)piperidine-1-carboxylate (4nm).**

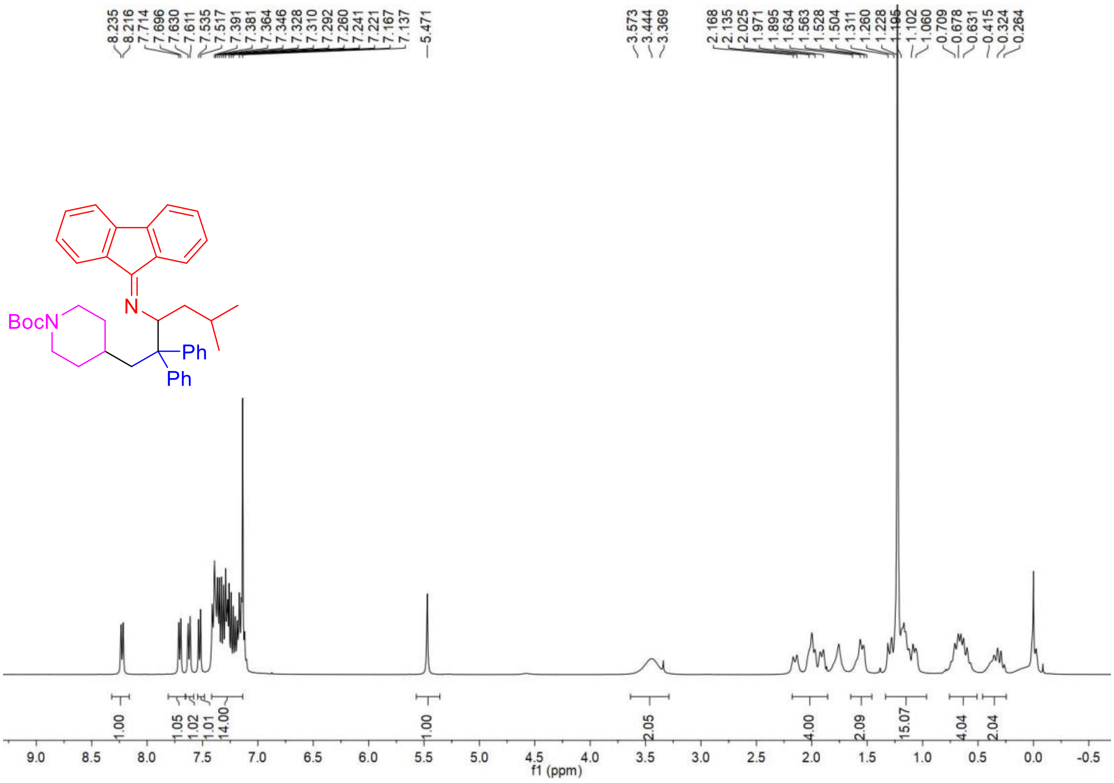

Figure S94. **^{13}C{^{1}H} NMR spectra (100 MHz, Chloroform-*d*) of *tert*-Butyl 4-(3-((9*H*-fluoren-9-ylidene)amino)-5-methyl-2,2-diphenylhexyl)piperidine-1-carboxylate (4nm).**

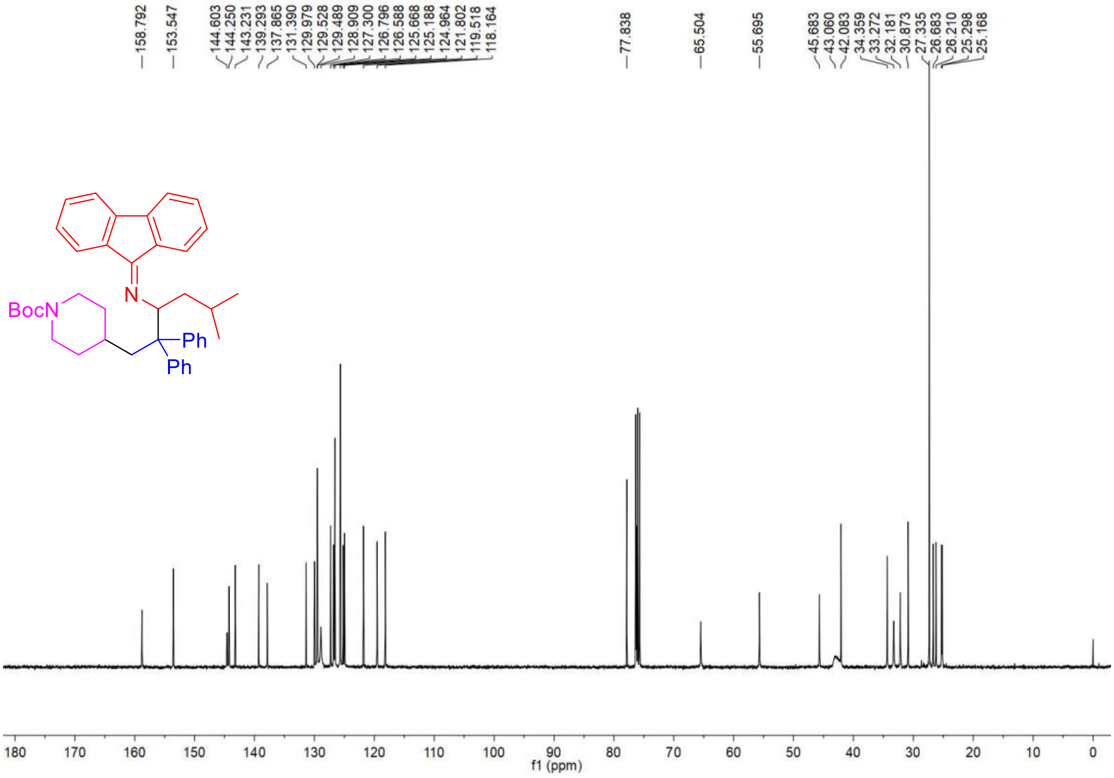

Figure S95. **^{1}H NMR spectra (400 MHz, Chloroform-*d*) of *tert*-Butyl 4-(3-((9*H*-fluoren-9-ylidene)amino)-4-methyl-2,2-diphenylpentyl)piperidine-1-carboxylate (4om).**

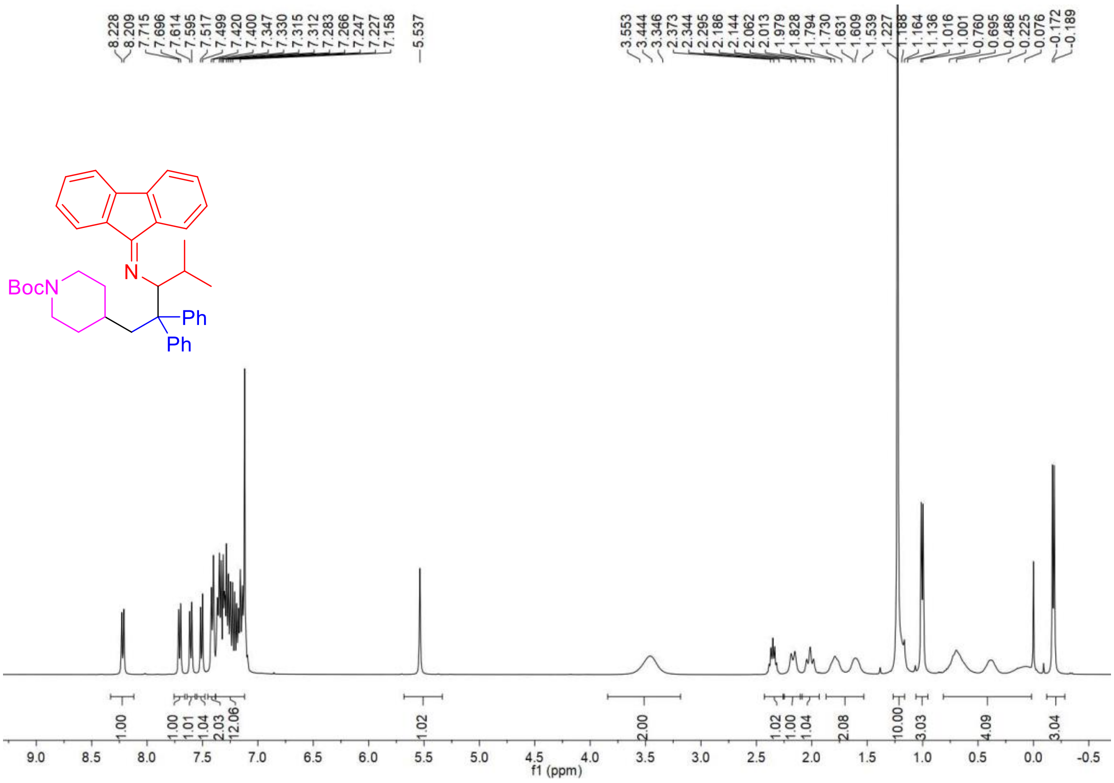

Figure S96. **^{13}C{^{1}H} NMR spectra (100 MHz, Chloroform-*d*) of *tert*-Butyl 4-(3-((9*H*-fluoren-9-ylidene)amino)-4-methyl-2,2-diphenylpentyl)piperidine-1-carboxylate (4om).**

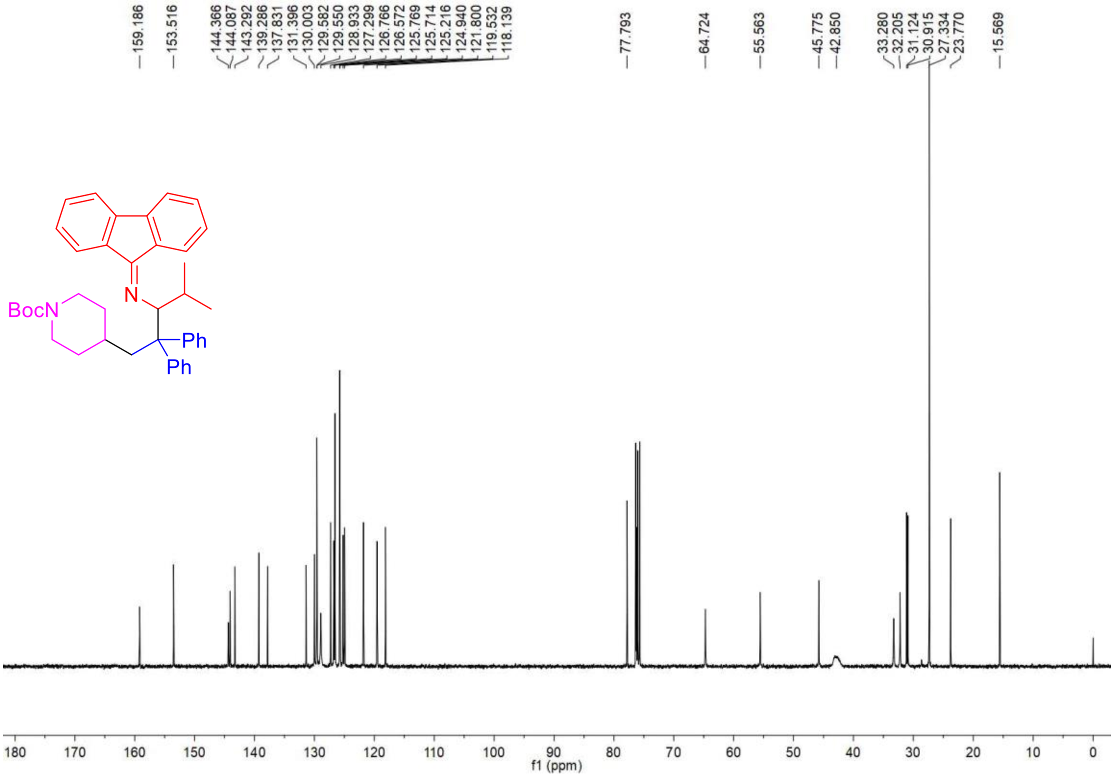

Figure S97. **^{1}H NMR spectra (400 MHz, Chloroform-*d*) of *tert*-Butyl 4-(3-((9*H*-fluoren-9-ylidene)amino)-3-cyclobutyl-2,2-diphenylpropyl)piperidine-1-carboxylate (4pm).**

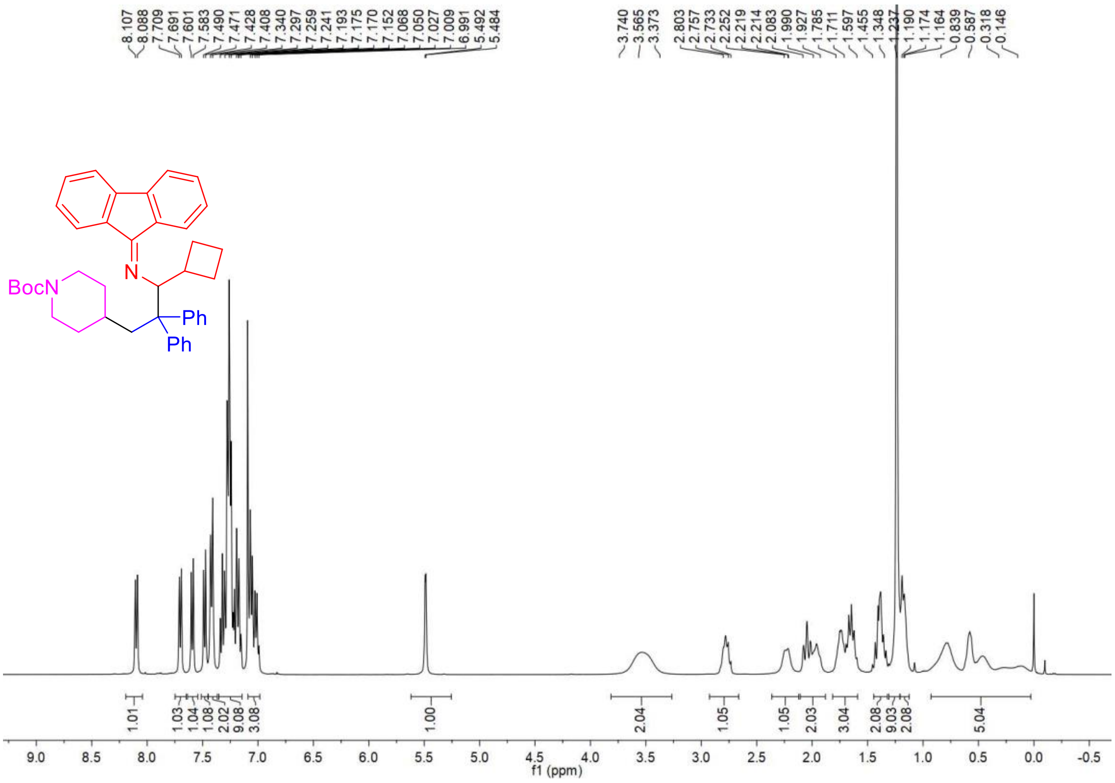

Figure S98. **$^{13}C\{^{1}H\}$ NMR spectra (100 MHz, Chloroform-*d*) of *tert*-Butyl 4-(3-((9*H*-fluoren-9-ylidene)amino)-3-cyclobutyl-2,2-diphenylpropyl)piperidine-1-carboxylate (4pm).**

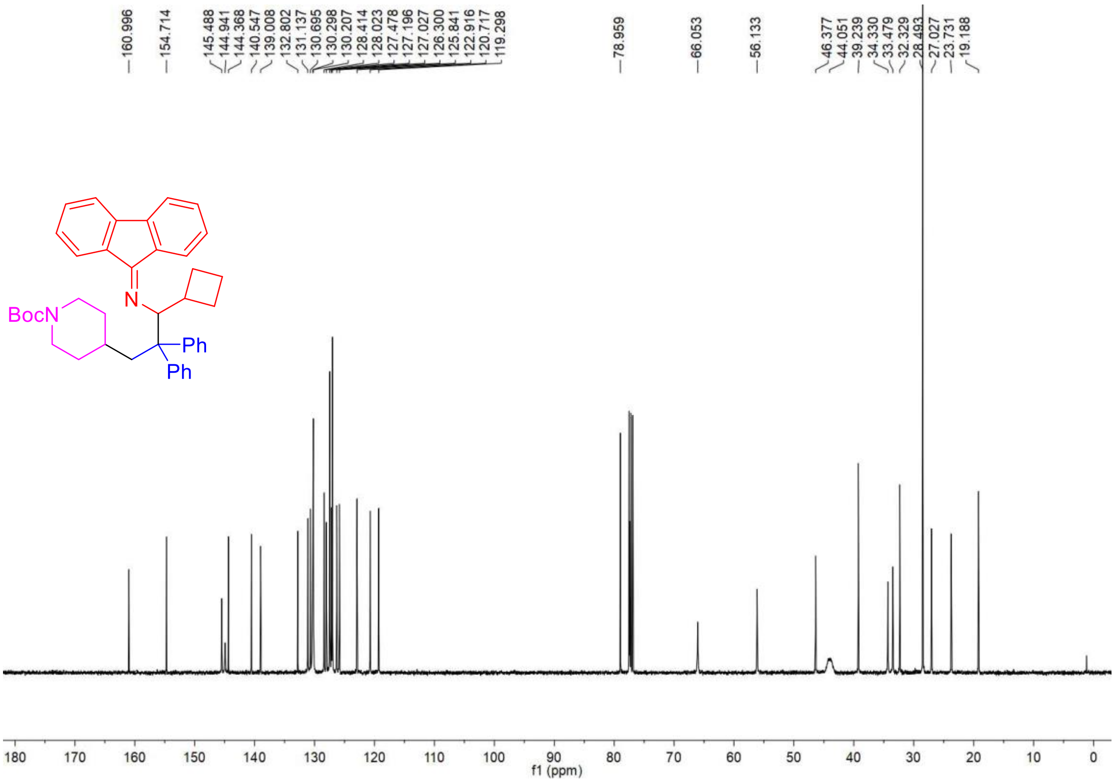

Figure S99. ^{1}**H NMR spectra (400 MHz, Chloroform-*d*) of *tert*-Butyl 4-(3-((9*H*-fluoren-9-ylidene)amino)-3-cyclopentyl-2,2-diphenylpropyl)piperidine-1-carboxylate (4qm).**

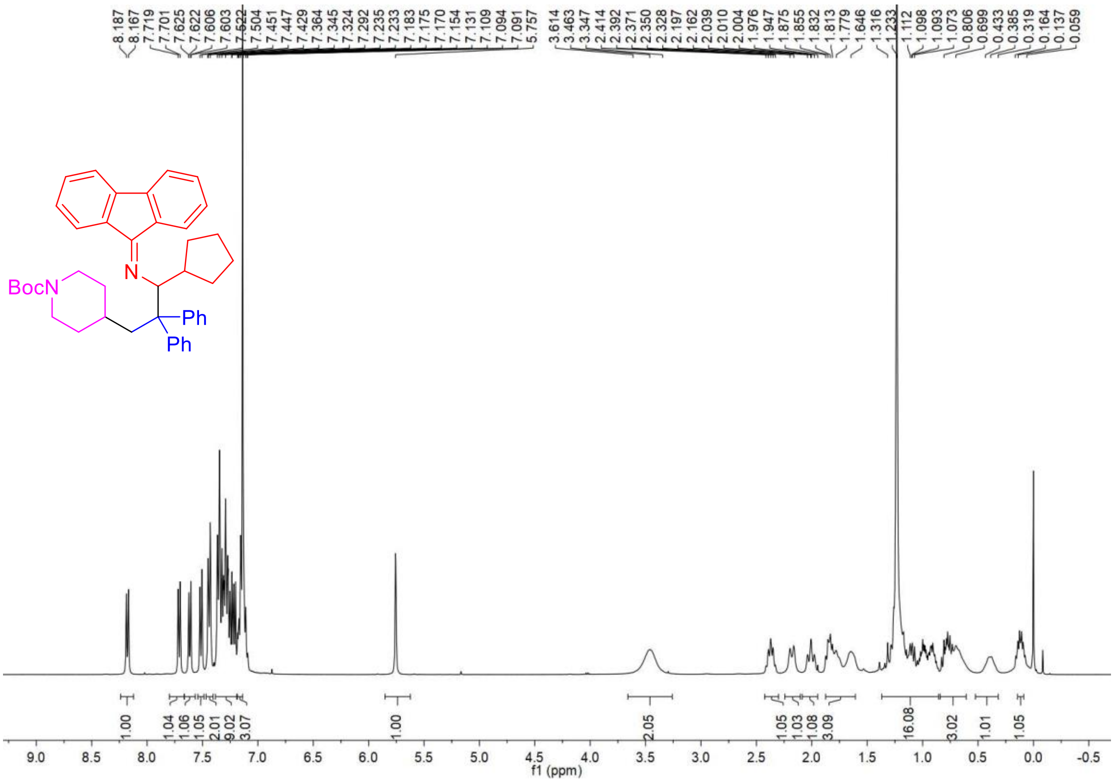

Figure S100. ^{13}**C{^{1}H} NMR spectra (100 MHz, Chloroform-*d*) of *tert*-Butyl 4-(3-((9*H*-fluoren-9-ylidene)amino)-3-cyclopentyl-2,2-diphenylpropyl)piperidine-1-carboxylate (4qm).**

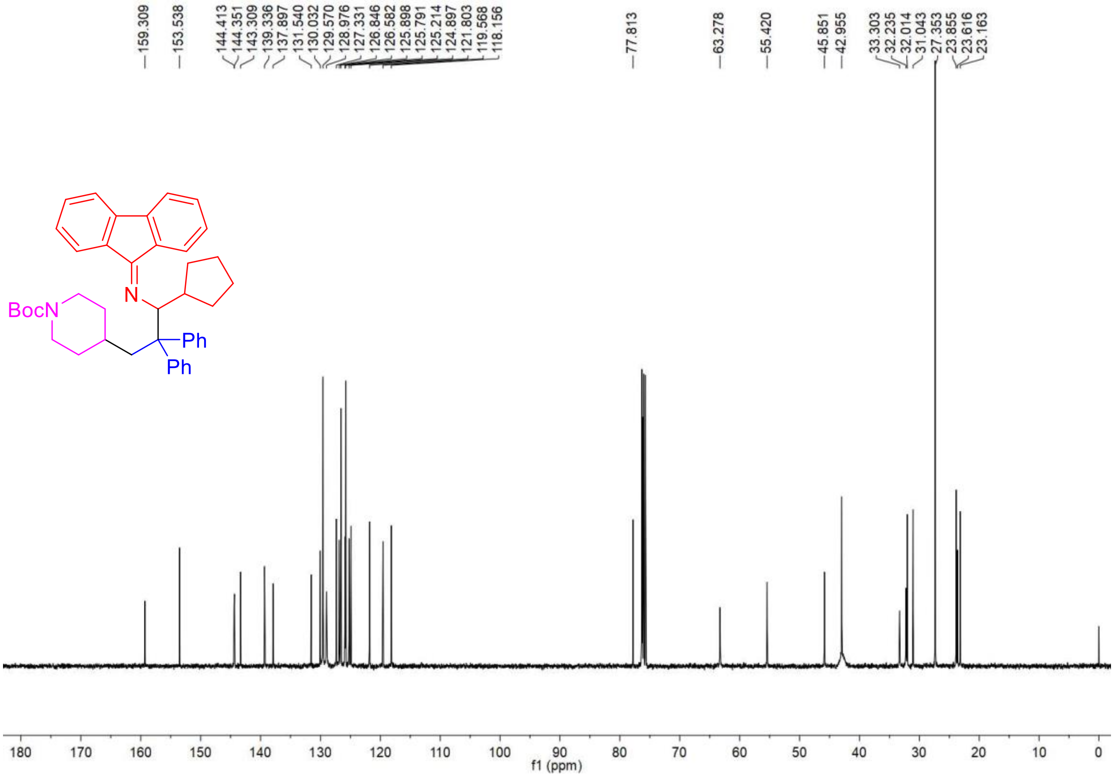

Figure S101. ^{1}H **NMR spectra (400 MHz, Chloroform-*d*) of *tert*-Butyl 4-(3-((9*H*-fluoren-9-ylidene)amino)-3-cyclohexyl-2,2-diphenylpropyl)piperidine-1-carboxylate (4rm).**

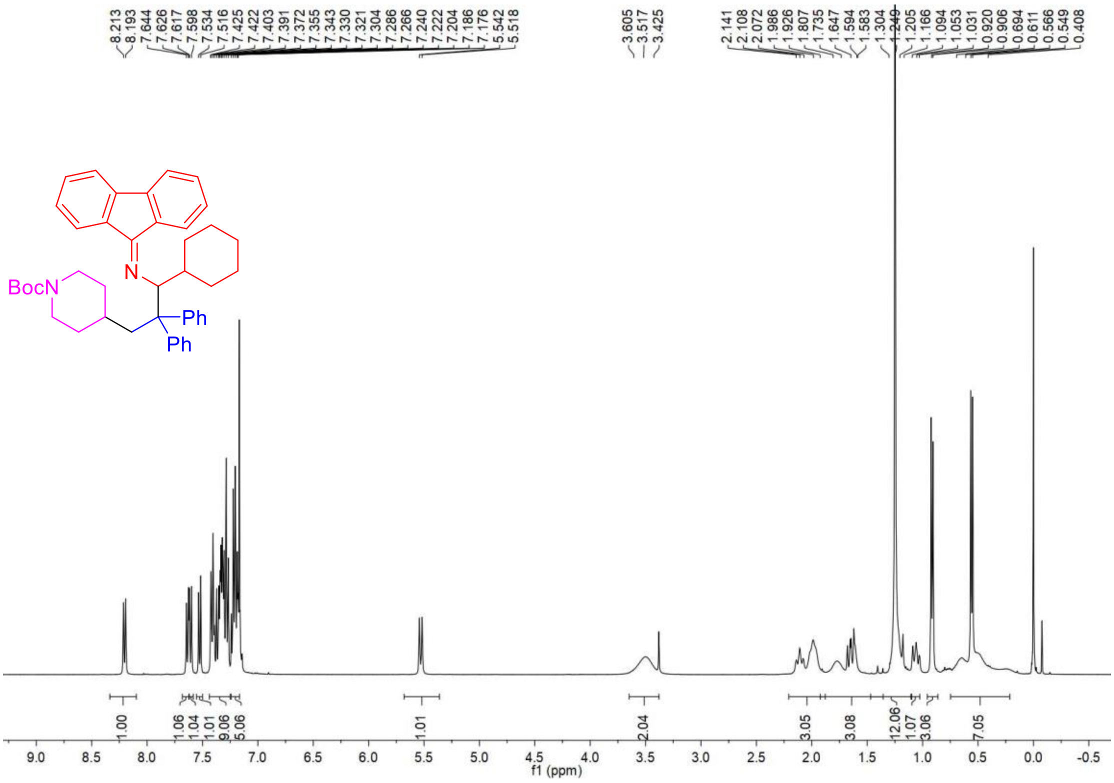

Figure S102. $^{13}C\{^{1}H\}$ **NMR spectra (100 MHz, Chloroform-*d*) of *tert*-Butyl 4-(3-((9*H*-fluoren-9-ylidene)amino)-3-cyclohexyl-2,2-diphenylpropyl)piperidine-1-carboxylate (4rm).**

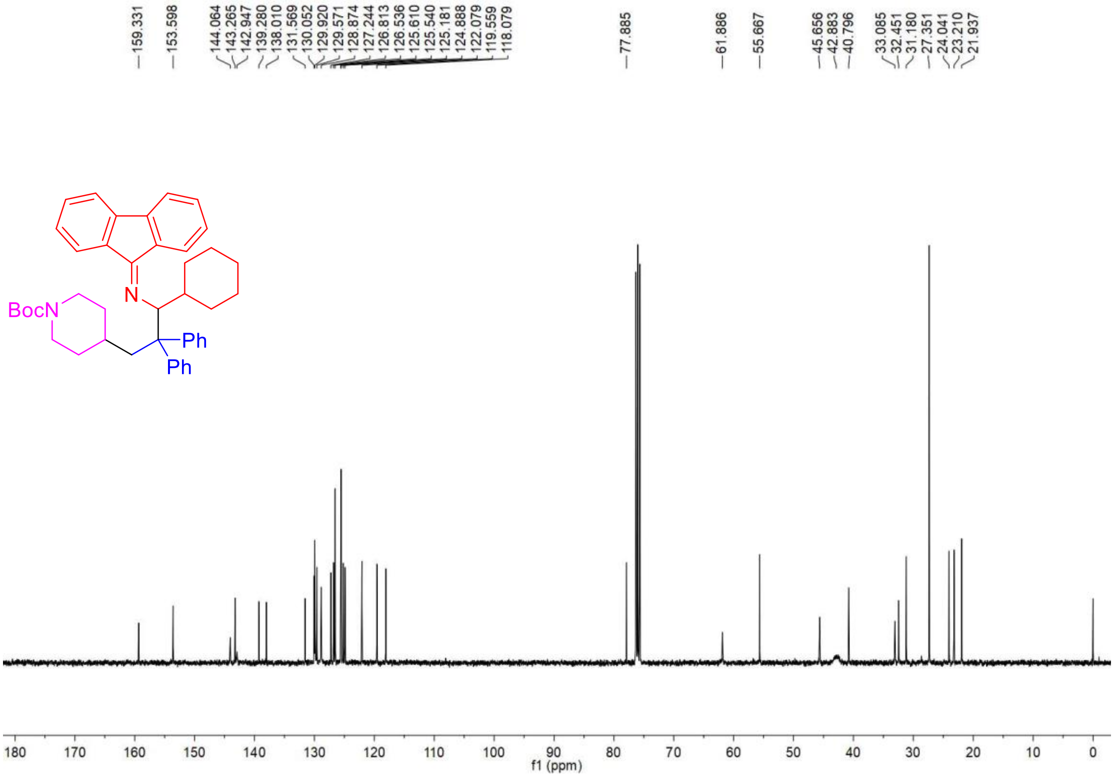

Figure S103. ^{1}**H NMR spectra (400 MHz, Chloroform-*d*) of 3-Cyclohexyl-1,2,2-triphenylpropan-1-amine (5al).**

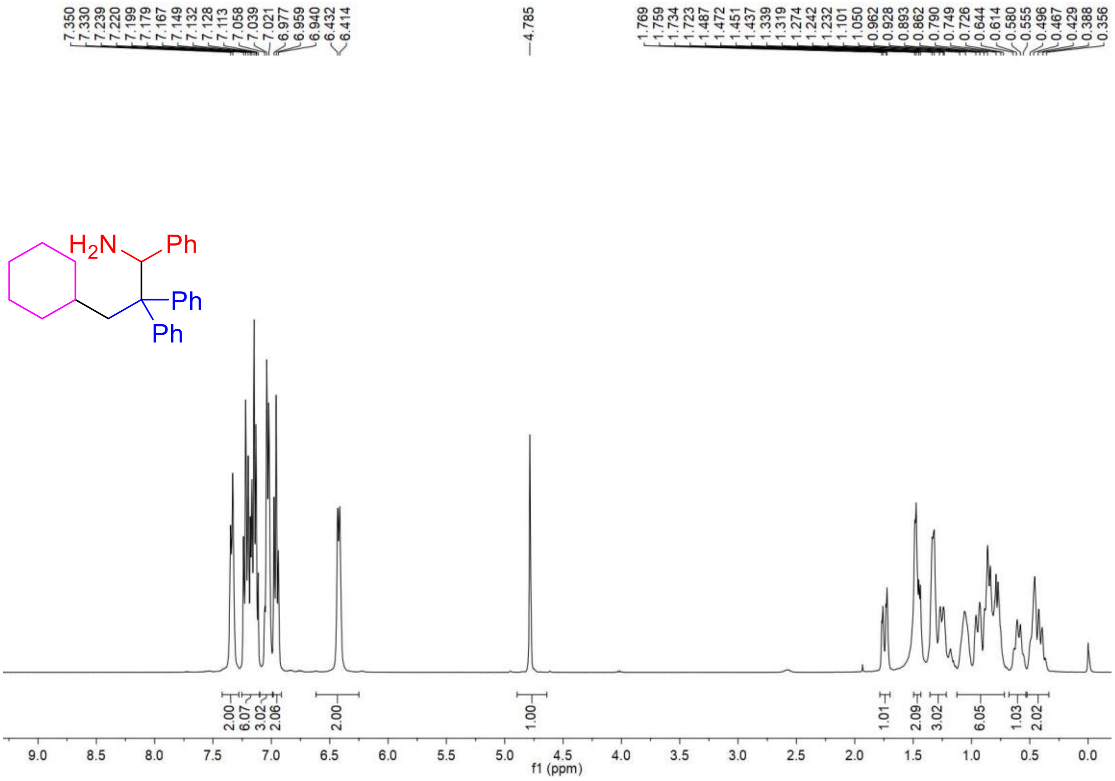

Figure S104. ^{13}**C{**^{1}**H} NMR spectra (100 MHz, Chloroform-*d*) of 3-Cyclohexyl-1,2,2-triphenylpropan-1-amine (5al).**

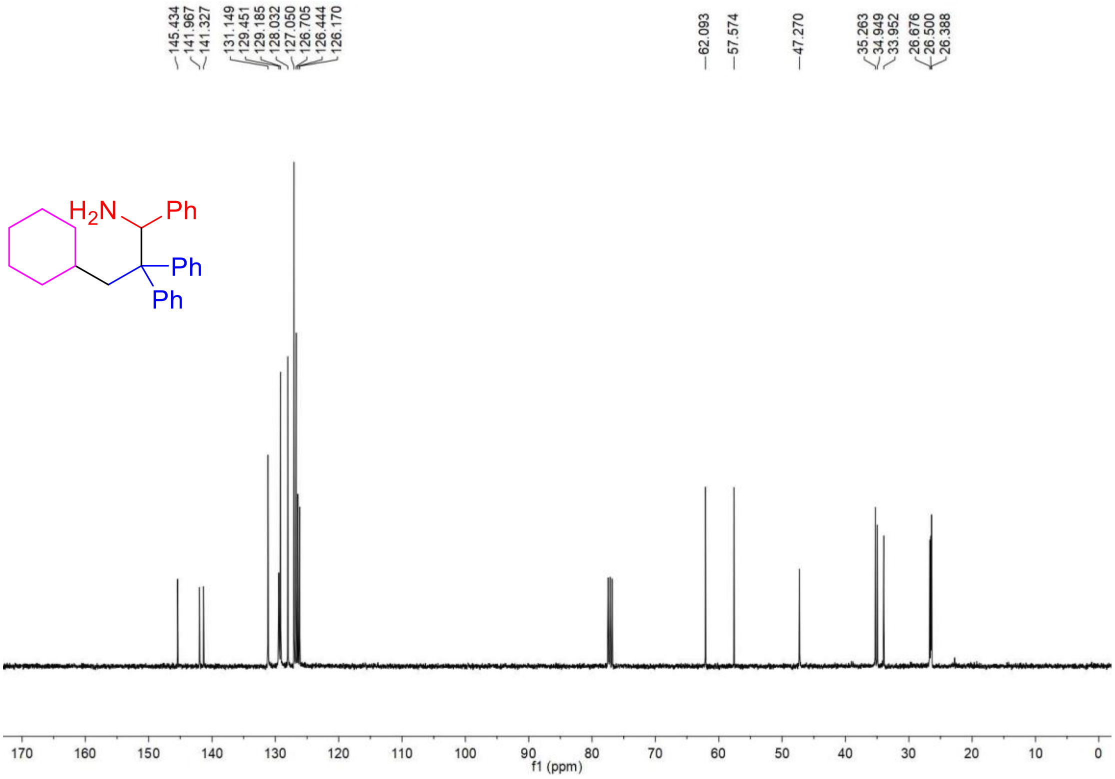

Supplement: SC-013-D2SC00500J-s001 [file SC-013-D2SC00500J-s001.pdf]
